# A new synthetic approach to the 3,4-dihydro-1*H*-[1,4]oxazino[4,3-*a*]indole system from ethyl 1*H*-indole-2-carboxylates and activated glycerol carbonate†

**DOI:** 10.1039/d5ra02996a

**Published:** 2025-06-05

**Authors:** Inesa Zagorskytė, Eglė Arbačiauskienė, Greta Račkauskienė, Sergey Belyakov, Aurimas Bieliauskas, Patrick Rollin, Algirdas Šačkus

**Affiliations:** a Department of Organic Chemistry, Kaunas University of Technology Radvilėnų pl. 19, Kaunas LT-50254 Kaunas Lithuania egle.arbaciauskiene@ktu.lt; b Institute of Synthetic Chemistry, Kaunas University of Technology K. Baršausko g. 59 Kaunas LT-51423 Lithuania algirdas.sackus@ktu.lt; c Latvian Institute of Organic Synthesis Aizkraukles 21 LV-1006 Riga Latvia; d Université d’Orléans et CNRS, ICOA, UMR 7311 BP 6759 F-45067 Orléans France

## Abstract

An efficient synthesis of a small library of potentially bioactive 3,4-dihydro-1*H*-[1,4]oxazino[4,3-*a*]indoles is described through the reaction of ethyl 1*H*-indole-2-carboxylates and activated glycerol carbonate. The reactivity of the C-10 position of the system was utilized to access 10-halogenated, formylated, and (hetero)arylated derivatives, while the 3-hydroxymethyl appendage was further converted into various 3-*O*-, 3-*S*-, or 3-*N*-derivatives. The structures of the synthesized compounds were elucidated using ^1^H-, ^13^C-, and ^15^N-NMR, IR spectroscopy, high-resolution mass spectrometry, and X-ray crystallography analyses. The photophysical properties of the selected compounds were investigated using spectroscopic techniques, including UV-vis and fluorescence spectroscopy.

## Abstract 2

An efficient synthesis of a small library of potentially bioactive 3,4-dihydro-1*H*-[1,4]oxazino[4,3-*a*]indoles is described through the reaction of ethyl 1*H*-indole-2-carboxylates and activated glycerol carbonate.

## Graphical abstract

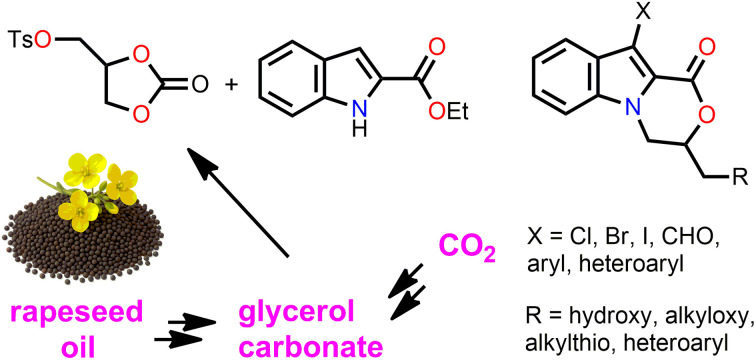

## Introduction

Fused indoles are an important class of heterocyclic compounds, as they are widely distributed in various natural products, pharmaceuticals, agrochemicals, and functional materials.^1–6^ For example, fused indole compounds exhibit a wide variety of biological properties, including insecticidal, anti-fungal, anti-HIV, anti-cancer, anti-diabetic, tobacco mosaic anti-virus, anti-inflammation, and other properties.^7–13^ Some of the fused indole compounds are present in marketed drugs, such as reserpine, pericine, uleine, vincamine, and yohimbine, as well as other indole alkaloids.^14–18^ In material sciences, extensive research on fused indole derivatives has been conducted over the past three decades to develop various organic dyes for developing dye-sensitized solar cells (DSSCs).^19–22^

**Fig. 1:**
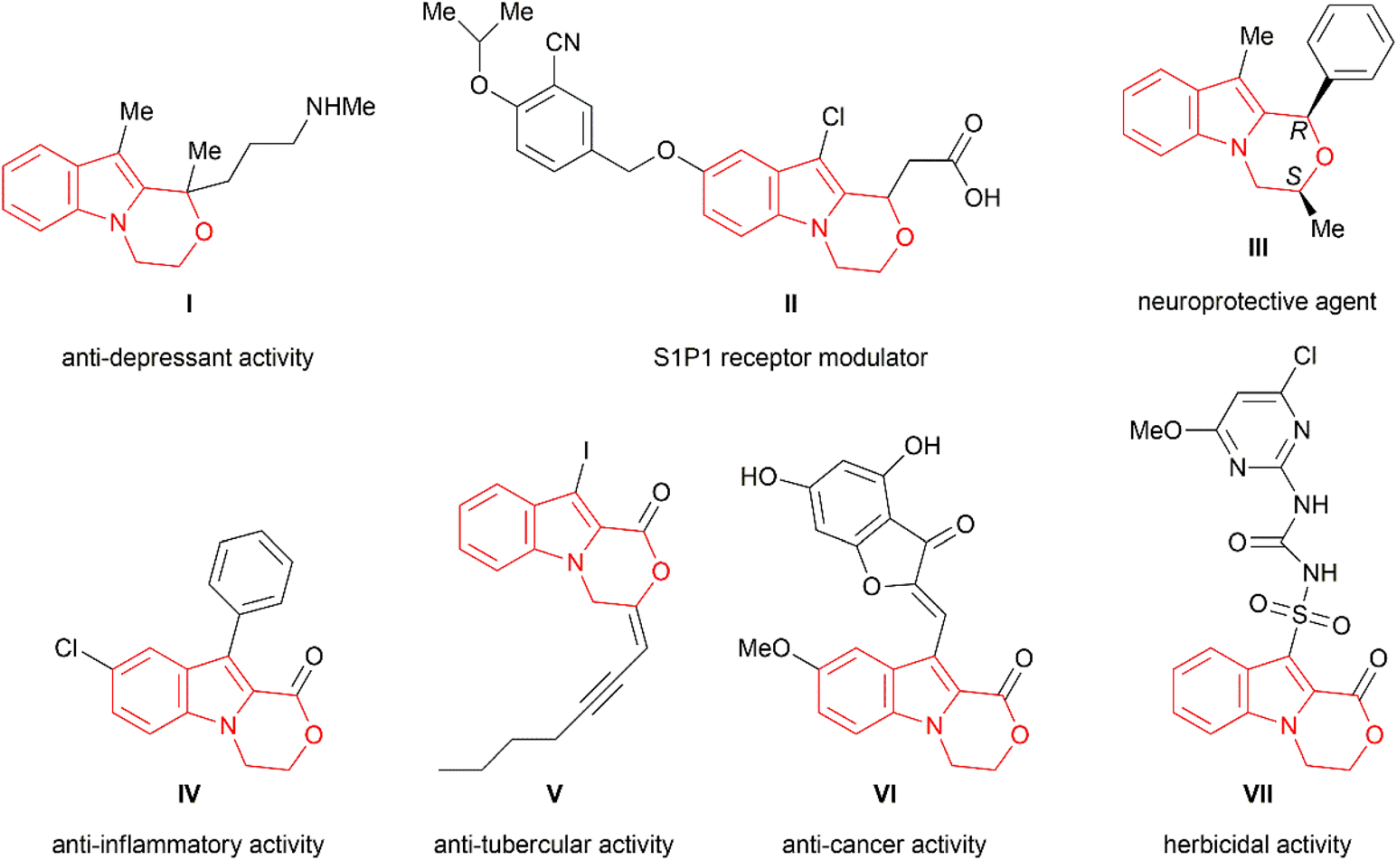
Some examples of oxazino[4,3-*a*]indole-based biologically active molecules.

Among the other fused indole systems, the oxazino[4,3-*a*]indole core has led to diverse structures with biological activities.^23,24^ In particular, oxazino[4,3-*a*]indole I is known to have an antidepressant effect^25^ (Fig. 1), while oxazino[4,3-*a*]indole II is a selective potent modulator of the S1P1 receptor, which may be a potential therapeutic agent for the effective treatment of autoimmune diseases.^26^ Their derivatives exhibited an anti-atherosclerotic effect in a mouse model through JAK/STAT phosphorylation down-regulation.^27^ Chiral oxazino[4,3-*a*]indole derivative III acts as a potential and selective neuroprotective agent against Aβ_25–35_-induced neuronal damage.^28^ Oxazino[4,3-*a*]indole lactone IV was found to be an anti-inflammatory agent,^29^ and lactone V displayed anti-tuberculary activity,^30^ while lactone VI is known to possess potential anti-cancer activity.^31^ Furthermore, oxazinoindolone-sulfonylurea VII exhibits herbicidal activity and is used as a general or selective post- and pre-emergent herbicide or plant growth regulator.^32^

Numerous methods for forming oxazino[4,3-*a*]indole ring systems have been developed.^23,24^ Most synthetic methods to access oxazino[4,3-*a*]indoles require a 1*H*-indole as precursor, a suitable alkylating agent, and intramolecular cyclization conditions. For example, a patent was granted for the synthesis of 10-phenyl-3,4-dihydro-1*H*-[1,4]oxazino[4,3-*a*]indol-1-one from 3-phenyl-1*H*-indole-2-carboxylic acid with BrCH_2_CH_2_Cl in the presence of NaH in DMF.^29^ Bandini *et al.* synthesized (4-ethoxy-4-oxobut-2-en-1-yl)-1*H*-indole-2-carboxylate which underwent *t*BuOK-induced cyclization to provide the desired chiral oxazino[4,3-*a*]indole derivative, ethyl (1-oxo-3,4-dihydro-1*H*-[1,4]oxazino[4,3-*a*]indol-4-yl)acetate.^33^ Chen *et al.* prepared chiral 1-phenyl-3,4-dihydro-1*H*-[1,4]oxazino[4,3-*a*]indole in two steps from 3-methyl-1*H*-indole with (*R*)- or (*S*)-methyloxirane, employing an intermolecular oxa-Pictet–Spengler reaction involving benzaldehyde.^28^

In recent decades, silver and gold salts have been applied as versatile catalysts to elaborate fused indole ring systems through intramolecular cyclization of 1*H*-indole substrates.^34–36^ For example, Taskaya *et al.* reported an efficient synthesis of oxazino[4,3-*a*]indole derivatives in good yields employing silver triflate (AgOTf) and gold trichloride (AuCl_3_), as promoters for the cyclization of easily accessible 1-propargyl-1*H*-indole-2- carboxylic acid.^37^ Maaliki *et al.* reported the iodocyclization of 1-propargyl-1*H*-indole-2-carboxylic acid in the presence of silver nitrate (AgNO_3_), diiodine, and sodium carbonate in tetrahydrofuran, which led to the formation of 10-iodo-3-(iodomethylidene)-3,4-dihydro-1*H*-[1,4]oxazino[4,3-*a*]indol-1-one,^30^ while Pedrazzari *et al.* reported the synthesis of 3-ethenyl-3,4-dihydro-1*H*-[1,4]oxazino[4,3-*a*]indol-1-ones through intramolecular cyclization of 1-allenyl-1*H*-indole-2-carboxylic acids by ImPyAuCl complexes.^38^ More recently, Michelet and coll. reported the preparation of chiral functionalized oxazino[4,3-*a*]indol-1-ones *via* a gold-mediated cycloisomerization/alkoxylation of (3-phenylprop-2-yn-1-yl)-1*H*-indole-2-carbaldehydes.^39^

We recently synthesized functionalized fused pyrazole compounds containing a pyrazolo[5,1-*c*][1,4]oxazine ring starting from tosylated glycerol carbonate (TGC) and 1*H*-pyrazole-5(3)-carboxylates and employing an alkylation-ring cleavage-cyclization sequence.^40^ Known as a versatile reagent for the synthesis of complex organic compounds and materials, TGC can be readily obtained from overproduced glycerol waste *via* glycerol carbonate.^41–46^ Herein, we report a novel synthetic route to prepare functionalized fused indole derivatives containing an oxazino[4,3-*a*]indole ring, starting from ethyl 1*H*-indole-2-carboxylates and activated glycerol carbonates, such as tosyl glycerol carbonate (TGC), and mesyl glycerol carbonate (MGC), followed by intramolecular cyclization. The obtained oxazino[4,3-*a*]indole system was further functionalized at positions 3 and 10 to afford a diversified library of compounds.

## Results and discussion

### Synthesis

**Scheme 1:**
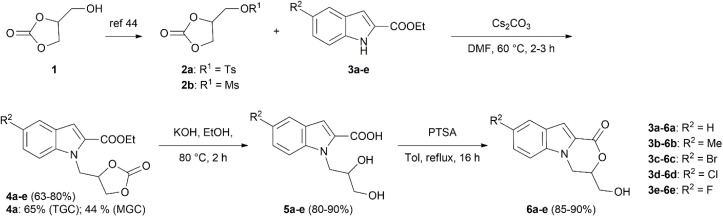
Synthesis of 3-(hydroxymethyl)-3,4-dihydro-1*H*-[1,4]oxazino[4,3-*a*]indol-1-ones 6a–e.

The synthesis of 3,4-dihydro-1*H*-[1,4]oxazino[4,3-*a*]indoles 6a–e was carried out as depicted in Scheme 1. First, glycerol carbonate 1 was treated with tosyl- or mesylchlorides following the known procedures,^44^ to afford activated glycerol carbonate derivatives, TGC (2a) and MGC (2b), respectively. Next, NH-indoles 3a–e were alkylated in DMF using TGC (2a) in the presence of Cs_2_CO_3_ as a base. Alkylation experiments were conducted using 1*H*-indole-2-carboxylate 3a as a model compound, and the effect of temperature on the reaction outcome was investigated. Stirring the reaction mixture at 80 °C for 2 hours provided *N*-glycerylated indole 4a in 47% yield, while some of 3a remained unreacted. Proceeding with the reaction for 16 hours led to a significant decrease in the yield of 4a to only 10%, presumably due to decomposition of the product. Stirring the reaction mixture at 60 °C for 3 hours enabled full conversion of 3a, thereby improving the yield of 4a to 65%. In contrast, the efforts to obtain glycerylated indole 4a from 3a and MGC (2b) resulted in 44% yield only, thus indicating a comparatively higher indole *N*-glycerination activity of TGC.

Using TGC and applying the same alkylation reaction conditions (Cs_2_CO_3_, DMF, 60 °C) to ethyl 5-substituted 1*H*-indole-2-carboxylates 3b–e, provided targeted *N*-glycerylated indoles 4b–e in 63–80% yields.

Furthermore, a two-step oxazino[4,3-*a*]indol-1-one synthesis procedure, similar to our previously reported strategy, was employed in the synthesis of structurally related pyrazolo[5,1-*c*][1,4]oxazines.^40^ Ethyl 1-[(2-oxo-1,3-dioxolan-4-yl)methyl]-1*H*-indole-2-carboxylates 4a–e were reacted with KOH in ethanol for 2 hours, resulting in both decarboxylative ring cleavage and ester hydrolysis to form 1-(2,3-dihydroxypropyl)-1*H*-indole-2-carboxylic acids 5a–e with very good yields of 80–90%. Subsequently, intramolecular Fischer–Speier esterification^47^ was performed by refluxing 5a–e in dry toluene in the presence of a catalytic amount of *p*-toluenesulfonic acid, providing 6a–e in excellent yields of 85–90% (Scheme 1). Comparing to other synthetic methods to access oxazino[4,3-*a*]indoles the suggested route is more sustainable as the green chemical glycerol carbonate is employed for TGC synthesis.^48,49^

**Scheme 2:**
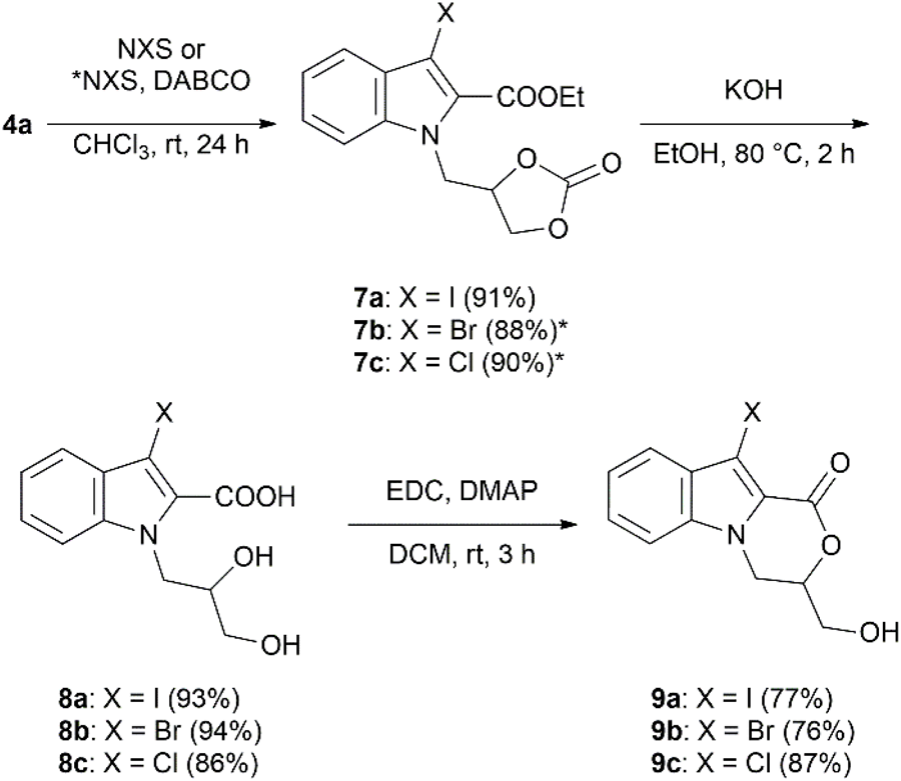
Synthesis of compounds 9a–c.

Halogenated oxazino-indoles can serve as valuable intermediates for expanding structural diversity *via* transition metal-catalyzed cross-coupling processes, such as Suzuki–Miyaura, Stille, Sonogashira, Negishi, Heck, and Fukuyama reactions.^50–59^ In addition, halogen substituents may improve the bioactivity of oxazino[4,3-*a*]indole derivatives.^26,30^ Therefore, the structural diversity of 3,4-dihydro-1*H*-[1,4]oxazino[4,3-*a*]indoles was expanded through their 10-halogenated counterparts. Iodination of 4a was carried out at room temperature using *N*-iodosuccinimide,^60,61^ and iodinated *N*-glycerylindole 7a was obtained with an excellent yield of 91% (Scheme 2). For the halogenation of 4a with *N*-bromosuccinimide or *N*-chlorosuccinimide, a catalytic amount of DABCO was added to increase the reaction efficiency, as reported by Xu *et al.*^62^ As a result, brominated indole 7b and its chlorinated analogue 7c were isolated with excellent yields of 88% and 90%, respectively. Halogenated 1-(2,3-dihydroxypropyl)-1*H*-indole-2-carboxylic acids 8a–c were further prepared following the same procedure as described for the synthesis of 5a–e; however, considering the low solubility of 8a–c in non-polar solvents and the oxazino[4,3-*a*]indole ring's susceptibility to cleavage, Steglich esterification conditions^63,64^ were applied instead of *p*-toluenesulfonic acid induced intramolecular Fischer–Speier esterification, as a milder alternative approach. 10-Halogenated 3,4-dihydro-1*H*-[1,4]oxazino[4,3-*a*]indoles 9a–c were obtained with good to very good yields of 76–87% after stirring the reaction mixture in DCM at room temperature for 3 hours in the presence of carboxylic acid activating EDC and DMAP as a base (Scheme 2).

Further development of 3-(hydroxymethyl)-3,4-dihydro-1*H*-[1,4]oxazino[4,3-*a*]indol-1-ones aimed to broaden the scope of structural diversity within the system by introducing varied 3-*O*-, 3-*S*-, or 3-*N*-substituents.

**Scheme 3:**
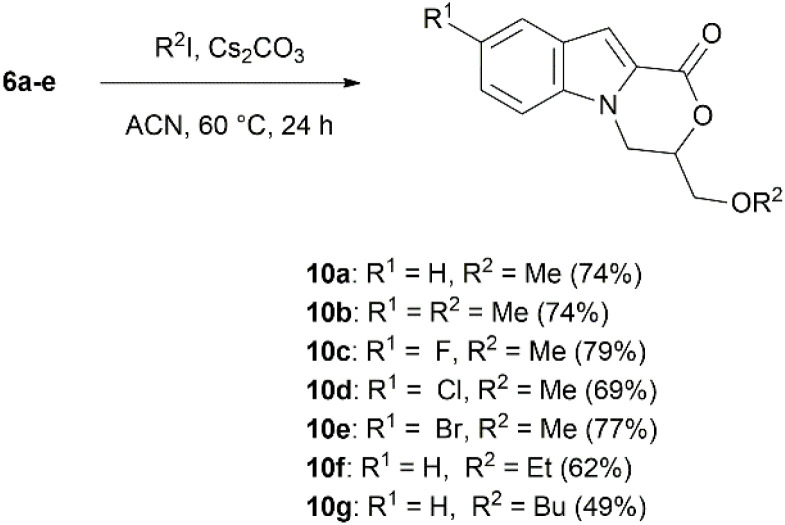
Synthesis of compounds 10a–g.

*O*-Alkylation experiments of 3-(hydroxymethyl)-3,4-dihydro-1*H*-[1,4]oxazino[4,3-*a*]indol-1-ones were performed with various alkyl halides (Scheme 3). Deprotonation of primary alcohols usually requires a strong base, such as NaH with a pKa of 37.^65,66^ Therefore, alcohol 6a was deprotonated using NaH, and the methylation reaction was tested with MeI in DMF or THF at ambient or elevated temperature. However, opening of the lactone ring and the formation of complex reaction mixture was observed. *O*-Methylation of 6a in DMF using K_2_CO_3_ (ref. 67) gave no positive result either, whereas switching the solvent to acetonitrile (ACN) or acetone afforded traces of targeted 10a. As Cs_2_CO_3_ has a considerably higher solubility in aprotic solvents compared to K_2_CO_3_,^68^*O*-alkylation of 6a–e was performed in the presence of Cs_2_CO_3_ in ACN, using different alkyl iodides to give 3-(alkoxymethyl)-3,4-dihydro-1*H*-[1,4]oxazino[4,3-*a*]indol-1-ones 10a–g with 49–79% yields (Scheme 3). It was observed that, with an increase in the alkyl halide chain length, the yield of *O*-alkylation decreased: 3-(ethoxymethyl)- and 3-(butoxymethyl)-3,4-dihydro-1*H*-[1,4]oxazino[4,3-*a*]indol-1-ones 10f and 10g were obtained in 62% and 49% yields, respectively.

**Scheme 4:**
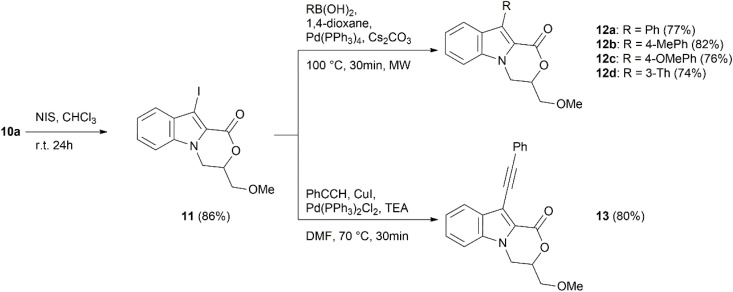
Synthesis of compounds 12 and 13.

In addition, the 10-(hetero)arylated or ethynylated analogs of 3-(alkoxymethyl)-3,4-dihydro-1*H*-[1,4]oxazino[4,3-*a*]indol-1-ones 10a–g were synthesized. First, oxazino[4,3-*a*]indol-1-one 10a was iodinated using NIS in CHCl_3_ to yield the 10-iodo compound 11 in 86% yield. The latter further underwent a palladium-catalyzed Suzuki cross-coupling reaction under anhydrous conditions with phenyl, 4-methylphenyl, 4-methoxyphenyl, and thien-3-yl boronic acids, providing the corresponding cross-coupled products 12a–d with yields of 74–82% (Scheme 4). Interestingly, a decomposition of the oxazine ring was observed when aqueous Suzuki reaction conditions (Pd(PPh_3_)_4_, Cs_2_CO_3_, DMSO, H_2_O) were applied. In addition, the Sonogashira reaction conditions (Pd(PPh_3_)_2_Cl_2_, CuI, TEA, and DMF) were employed for the synthesis of 10-(phenylethynyl)-3,4-dihydro-1*H*-[1,4]oxazino[4,3-*a*]indol-1-one 13, which was obtained in 80% yield.

**Scheme 5:**
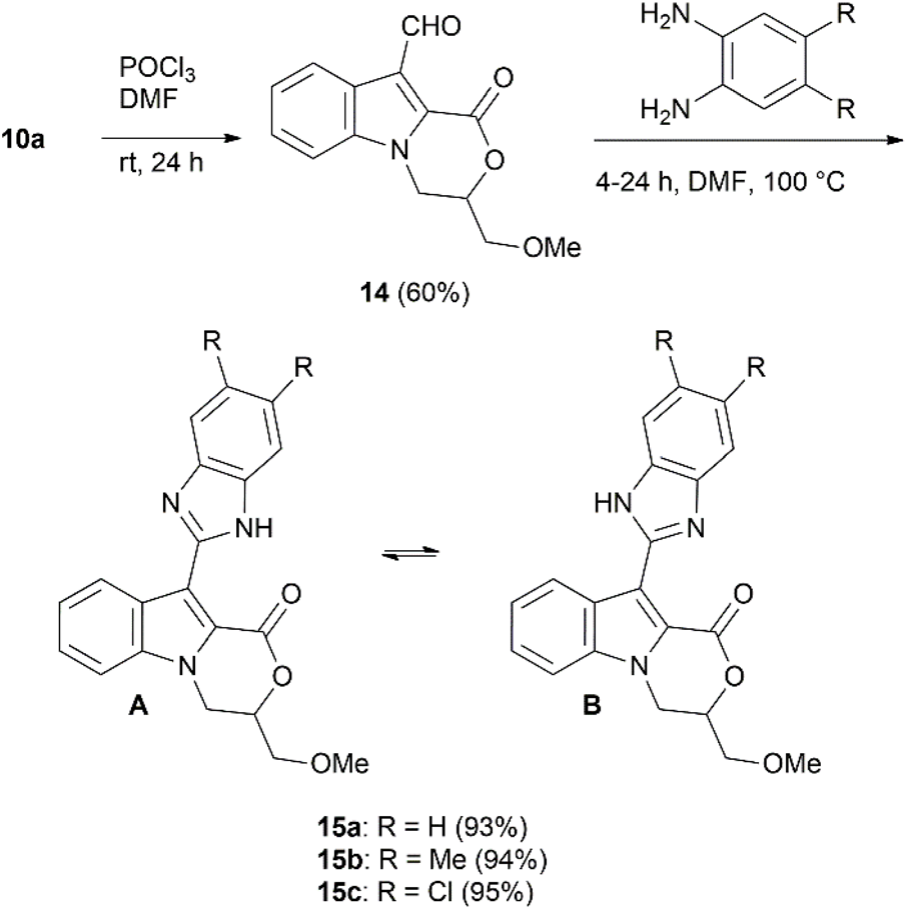
Synthesis of compounds 15a–c.

The C-10 nucleophilicity of 3,4-dihydro-1*H*-[1,4]oxazino[4,3-*a*]indol-1-one 10a was further highlighted by performing a formylation reaction under Vilsmeier–Haack conditions, similar to those reported by Pfizer chemists in their investigation of gamma-secretase modulators.^69^ The obtained carbaldehyde 14 was further reacted with benzene-1,2-diamine and its 4,5-dimethyl or 4,5-dichloro analogues to provide tautomeric 10-(1*H*-benzo[*d*]imidazole-2-yl)-3,4-dihydro-1*H*-[1,4]oxazino[4,3-*a*]indol-1-ones 15a–c with excellent 93–95% yields (Scheme 5).^70^

**Scheme 6:**
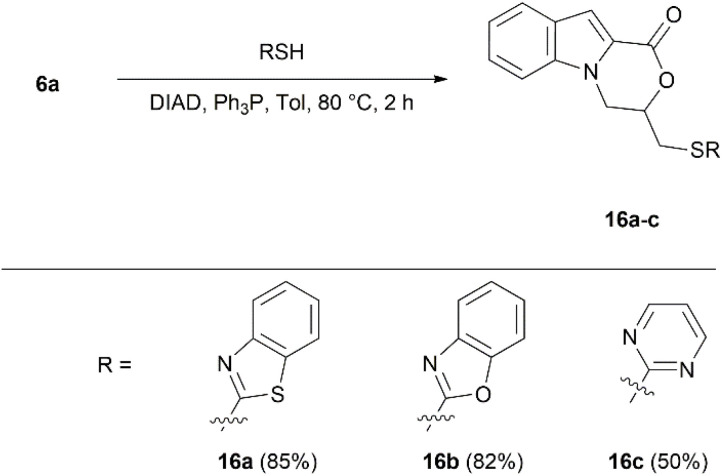
Synthesis of compounds 16a–c.

Further synthesis of 3-(heteroarylthio)methyl-substituted 3,4-dihydro-1*H*-[1,4]oxazino[4,3-*a*]indol-1-ones was inspired by the work of Rollin and coll., who obtained aza-heterocyclic thiosugar hybrids through Mitsunobu reaction conditions.^71^ Applying to 6a standard Mitsunobu protocols,^72^ in several solvents, including toluene, 1,4-dioxane, and THF, failed to deliver the targeted sulfides at room temperature. However, increasing the reaction temperature to 80 °C in toluene led to the formation of Mitsunobu reaction products 16a–c. 3-[(Benzo[*d*]thiazol-2-ylthio)methyl]-, 3-benzo[*d*]oxazol-2-ylthio)methyl]-, and 3-[(pyrimidin-2-ylthio)methyl]-3,4-dihydro-1*H*-[1,4]oxazino[4,3-*a*]indol-1-ones were formed with 85%, 82%, and 50% yields, respectively (Scheme 6).

**Scheme 7:**
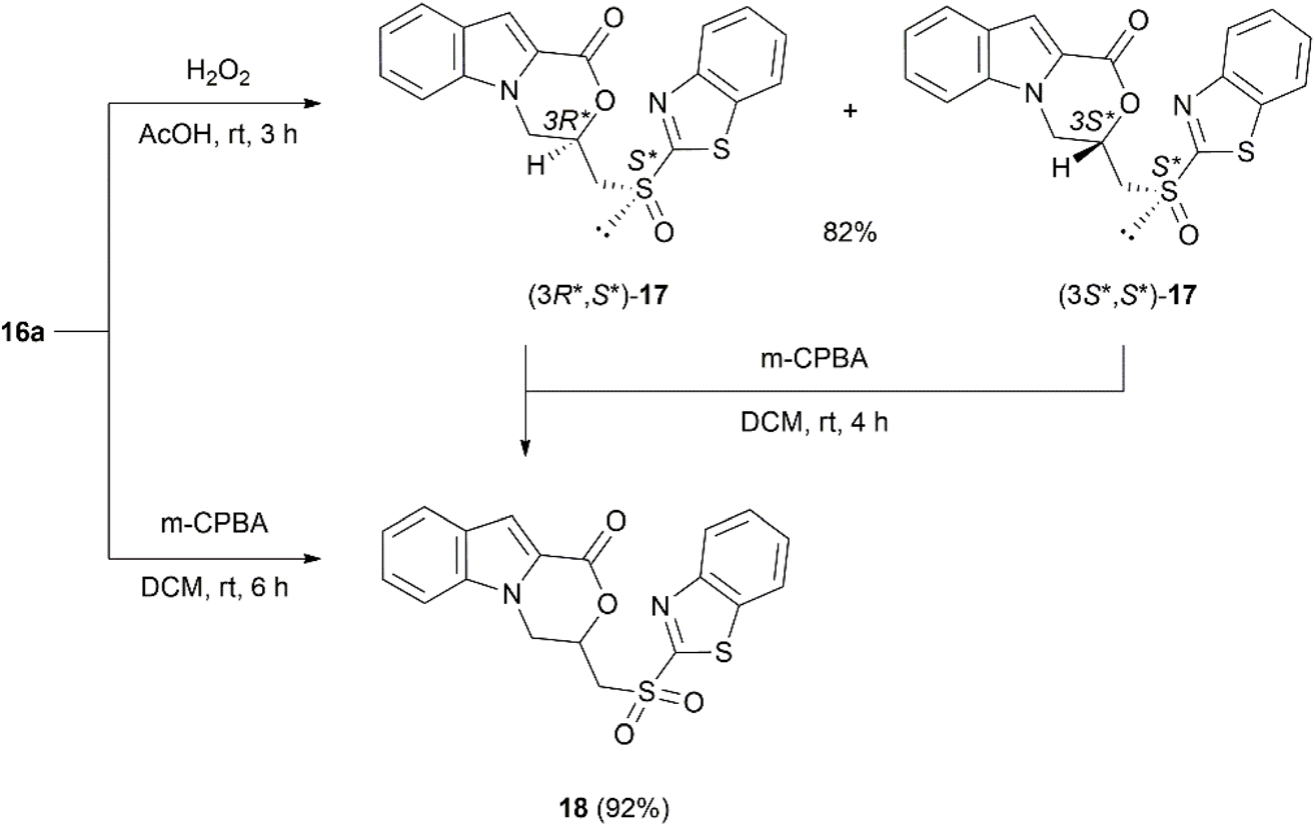
Synthesis of compound diastereomers (*R*,*S*)- and (*S*,*S*)- and (*S*,*R*)- and (*R*,*R*)-17 and compound 18.

The introduction of a sulfone or sulfoxide moiety into heterocyclic systems is sometimes known to significantly enhance biological activity.^73–76^ Therefore, several oxidation experiments were carried out with sulfide 16a. We first attempted to oxidize 3-[(benzo[*d*]thiazol-2-ylthio)methyl]-3,4-dihydro-1*H*-[1,4]oxazino[4,3-*a*]indol-1-one (16a) under mild conditions using hydrogen peroxide in glacial acetic acid.^77^ A full conversion into sulfoxide 17 was achieved after 48 hours; however, further oxidation led to traces of sulfone and the subsequent decomposition of compound 17. When Amberlyst 15 was added to generate peracetic acid *in situ*, the oxidation was accelerated, as reported by Tumula *et al.*^78^ The sulfoxide 17 formed in only 3 hours in 82% yield as an inseparable diastereomeric mixture (Scheme 7). A stronger oxidizing agent was required to reach the full oxidation to sulfone 18. Following the procedure of Ratovelomanana-Vidal *et al.* for the synthesis of benzothiazolylsulfones, 16a was reacted with *m*-chloroperoxybenzoic acid in DCM at room temperature for 6 hours, providing 3-[(benzo[*d*]thiazol-2-ylsulfonyl)methyl]-3,4- dihydro-1*H*-[1,4]oxazino[4,3-*a*]indol-1-one (18) with an excellent 92% yield (Scheme 7).^79^ Sulfoxide 17 contains two stereogenic centers (carbon C-3 and exocyclic sulfur), leading to a pair of diastereomers. In other respects, sulfone 18 has a stereogenic carbon in 3-position, which implies the existence of a mixture of enantiomers, (3*S*)- and (3*R*)-18.

**Scheme 8:**
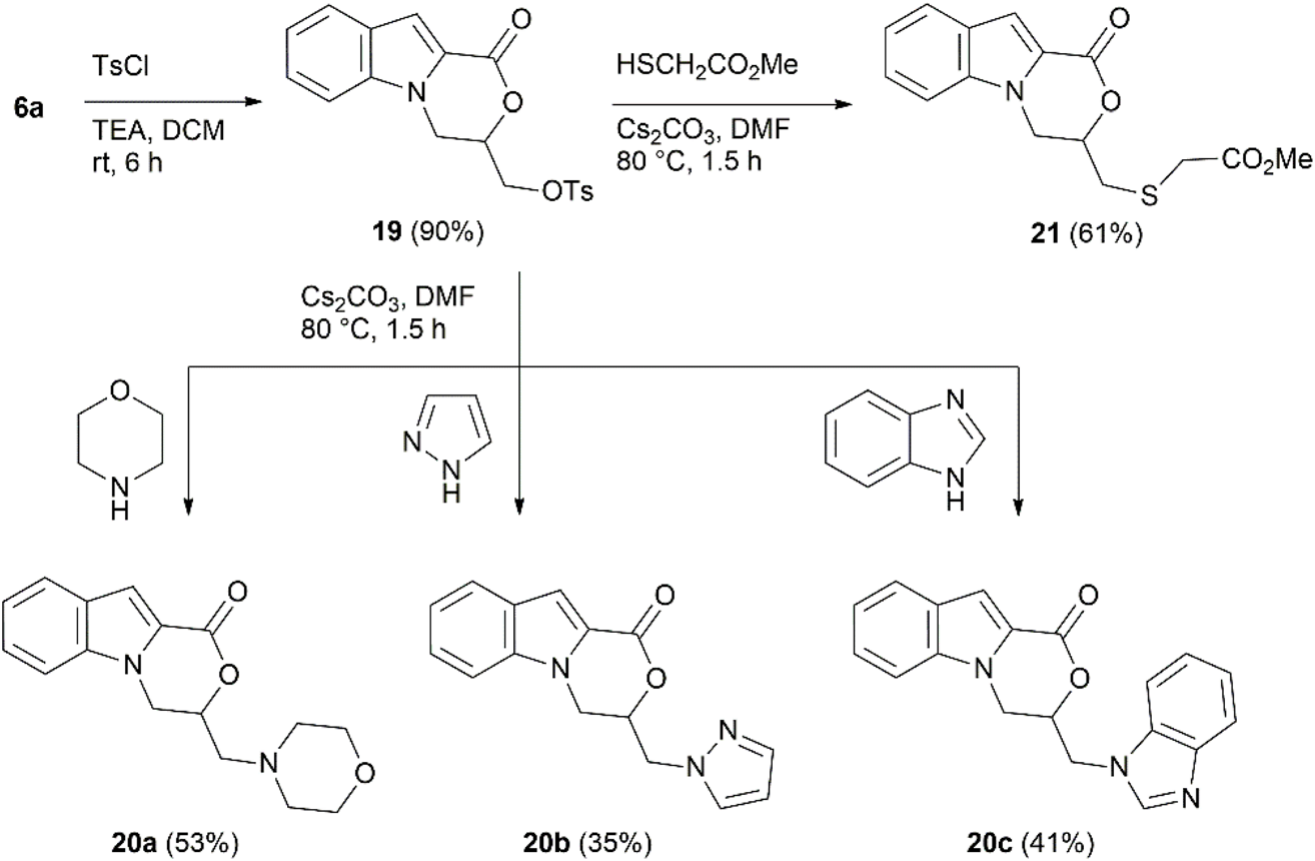
Synthesis of compounds 20 and 21.

After the successful 3-(heteroarylthio)methyl-substitution of indolo-oxazine 6a, similar Mitsunobu conditions were tested using both aliphatic as well as aromatic NH-heterocycles, but no formation of 3-(*N*-alkyl/*N*-arylmethyl)-3,4-dihydro-1*H*-[1,4]oxazino[4,3-*a*]indol-1-ones was observed. Therefore, we proceeded with an alternative method, starting with *O*-tosylation of 6a by TsCl in the presence of TEA in DCM.^80^ Tosylate 19 formed in 90% yield was subsequently used for the *N*-alkylation of morpholine, 1*H*-pyrazole, and 1*H*-benzimidazole in the presence of Cs_2_CO_3_ in DMF to afford 3-(morpholinomethyl)-, 3-[(1*H*-pyrazol-1-yl)methyl]-, and 3-[(1*H*-benzo[*d*]imidazole-1-yl)methyl]-3,4-dihydro-1*H*-[1,4]oxazino[4,3-*a*]indol-1-ones 20a–c with yields ranging from 35 to 53%. Similarly, *S*-alkylation of methyl thioglycolate provided the corresponding sulfide 21 in 61% yield (Scheme 8).

### NMR and IR spectroscopic investigations

**Fig. 2:**
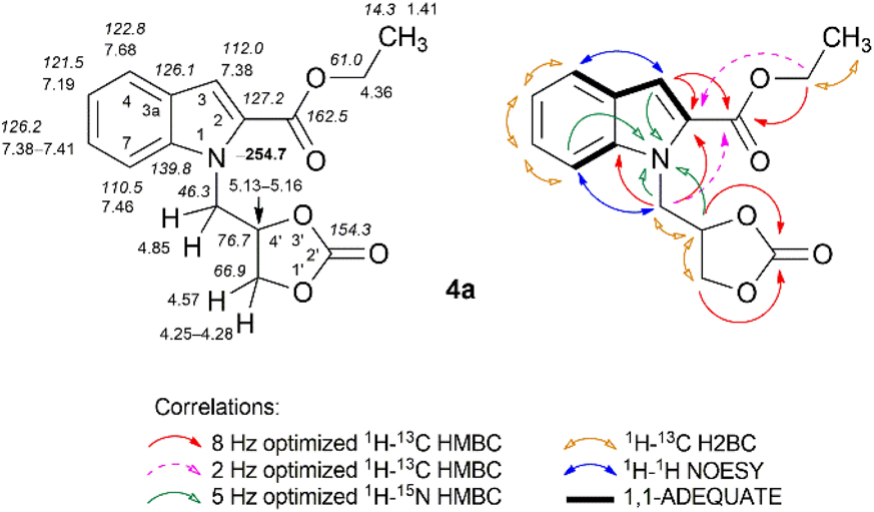
Relevant ^1^H–^13^C HMBC, ^1^H–^13^C H2BC, ^1^H–^15^N HMBC, ^1^H–^1^H NOESY, and 1,1-ADEQUATE correlations, as well as ^1^H NMR (italic), ^13^C NMR, and ^15^N NMR (bold) chemical shifts of compound 4a (CDCl_3_).

**Fig. 3:**
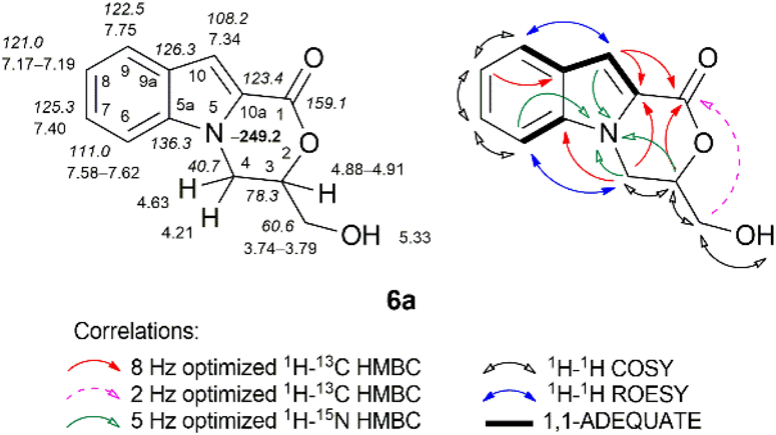
Relevant ^1^H–^13^C HMBC, ^1^H–^15^N HMBC, ^1^H–^1^H COSY, ^1^H–^1^H ROESY and 1,1-ADEQUATE correlations, as well as ^1^H NMR (italic), ^13^C NMR, and ^15^N NMR (bold) chemical shifts of compound 6a (DMSO-*d*_*6*_).

**Fig. 4:**
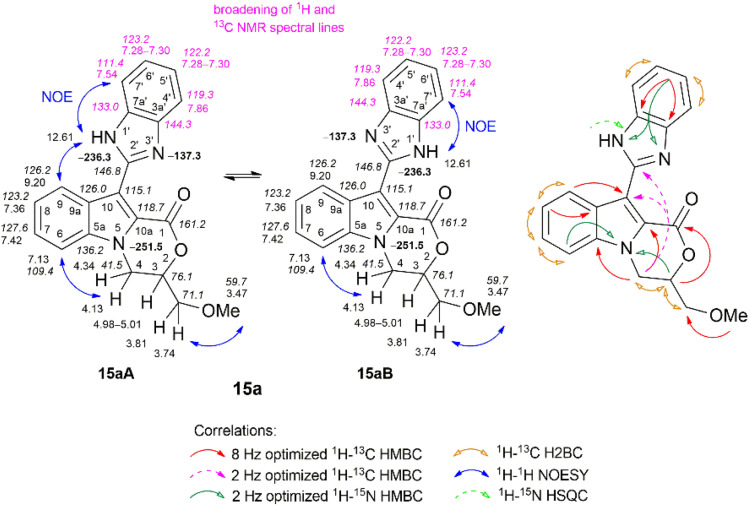
Relevant ^1^H–^13^C HMBC, ^1^H–^13^C H2BC, ^1^H–^15^N HMBC, ^1^H–^15^N HSQC and ^1^H–^1^H NOESY correlations, as well as ^1^H NMR (italic), ^13^C NMR, and ^15^N NMR (bold) chemical shifts of compound 15a (tautomers 15aA and 15aB) (CDCl_3_).

The structures of all the compounds were unambiguously confirmed *via* multinuclear NMR spectroscopy, infrared spectroscopy (IR), mass spectrometry (MS), and high-resolution mass spectrometry (HRMS). The NMR spectroscopic data for all compounds investigated in this study are presented in the Experimental section and ESI.† The combined application of standard NMR spectroscopic techniques such as ^1^H–^13^C HMBC, ^1^H–^13^C HSQC, ^1^H–^13^C H2BC, ^1^H–^15^N HMBC, ^1^H–^15^N HSQC, ^1^H–^1^H COSY, ^1^H–^1^H TOCSY, ^1^H–^1^H NOESY, ^1^H–^1^H ROESY, ^1^H–^1^H EXSY, and 1,1-ADEQUATE experiments confirmed an unequivocal assignment of the signals. The corresponding NMR data for the representative compounds 4a, 6a, 15a, 16b, and 20c are displayed in Fig. 2–4, 6, and 7, respectively.

Compound 4a bears a (2-oxo-1,3-dioxolan-4-yl)methyl moiety substituted at N-1 of the indole ring. The ^1^H NMR spectrum of compound 4a showed a characteristic 3-H proton singlet at *δ* 7.38 ppm. The ^1^H–^1^H NOESY spectrum of 4a exhibited distinct NOEs between the well-resolved indole 3-H proton and the neighboring indole 4-H proton (*δ* 7.68 ppm). In contrast, the NCH_2_ protons (doublet, *δ* 4.85 ppm) displayed correlation with the indole 7-H proton (*δ* 7.46 ppm), confirming their proximity in space. This finding, together with data from the 1,1-ADEQUATE and ^1^H–^13^C H2BC experiments, allowed us to unambiguously assign indole C-2 (*δ* 127.2 ppm), C-3a (*δ* 126.1 ppm), and C-7a (139.8 ppm) quaternary carbon signals. The 2 Hz optimized ^1^H–^13^C HMBC spectrum revealed correlations of the NCH_2_ protons with the C=O ester carbon at *δ* 162.5 ppm. Furthermore, the unambiguous formation of the *N*-glycerylated indole 4a was confirmed using the ^1^H–^15^N HMBC spectrum, in which clear long-range correlations were observed between the 3-H and 7-H protons of indole and the 4′-H proton of 2-oxo-1,3-dioxolane with indole N-1 nitrogen (*δ* –254.7 ppm) (Fig. 2). The IR spectrum of compound 4a showed characteristic absorption bands at 1707 and 1796 cm^−1^ (C=O stretching vibrations) for the ester functional group and the 2-oxo-1,3-dioxolane moiety, respectively.^81,82^ The unambiguous formation of the 3,4-dihydro-1*H*-[1,4]oxazino[4,3-*a*]indol-1-one ring system was readily established *via* analogous NMR spectroscopy experiments as described above (Fig. 3), supplemented with ^1^H–^1^H TOCSY, ^1^H–^1^H ROESY, and ^1^H–^1^H COSY spectral data.

Specifically, in the case of compound 6a, the ^1^H–^1^H COSY spectrum indicated the presence of a 3-hydroxymethyl appendage connected to newly formed oxazino[4,3-*a*]indol-1-one ring system, as it showed COSY cross peaks between the hydroxyl proton (triplet, *δ* 5.33 ppm) and methylene protons (*δ* 3.74–3.79 ppm), which further correlated with an adjacent methine 3-H proton (*δ* 4.88–4.91 ppm). Furthermore, the spectral data from the ^1^H–^1^H TOCSY spectrum clearly showed a spin system of six protons, which were upfield and belonged to the aliphatic part of the newly formed oxazino[4,3-*a*]indol-1-one ring ^1^H spin system, including the aforementioned hydroxyl proton. The ^1^H–^1^H ROESY spectrum of 6a exhibited distinct ROEs between the methylene 4-H protons (*δ* 4.21 and 4.63 ppm) and the neighboring indole 6-H proton (*δ* 7.58–7.62 ppm), connecting the aliphatic and aromatic ^1^H spin systems of oxazino[4,3-*a*]indol-1-one. The aforementioned oxazine methine 3-H proton, together with the well-resolved indole 10-H proton (singlet, *δ* 7.34 ppm), exhibited long-range correlations with the carbonyl carbon C-1 (*δ* 159.1 ppm) in the ^1^H–^13^C HMBC spectrum. The ^1^H–^15^N HMBC experiment revealed the expected long-range correlation between the indole H-6 and H-10 protons and the oxazine 3-H and 4-H protons with nitrogen N-5, which resonated at *δ* −249.2 ppm.

The IR spectrum of compound 6a exhibited characteristic absorption bands at 3403 cm^−1^ (O–H stretching vibrations) for the 3-hydroxymethyl moiety and at 1694 cm^−1^ (C=O stretching vibrations) for the morpholin-2-one moiety.

Advanced NMR spectrometry methods were extensively employed to determine the structure of oxazino[4,3-*a*]indole-benzimidazole 15a (Fig. 4). Notably, NMR spectroscopy also enables a wide range of experiments to investigate various dynamic properties of heterocyclic compounds.^83–85^ For example, Su *et al.*^86^ and Nieto *et al.*^87^ investigated the application of ^1^H and ^13^C NMR methods to study the prototropic tautomerism of omeprazole compounds, respectively.

In these cases, the 1D ^1^H and ^13^C NMR spectra resonances were analyzed, including the determination of the NMR spectral line broadening, and were used to identify tautomeric benzimidazole compounds.

Compound 15a bears a benzimidazol-2-yl moiety substituted at C-10 of the oxazino[4,3-*a*]indole ring system. In the ^1^H NMR spectrum of 15a in CDCl_3_, all protons of the oxazino[4,3-*a*]indole moiety, including the 9-H and CH_3_ protons, which are essential for linking various structural fragments, exhibited narrow 1D NMR spectral lines, due to a fast exchange on the NMR time scale (Fig. 4, ESI and S117†). However, the broadening of the protons 4′-H, 5′-H, 6′-H, and 7′-H was observed in the ^1^H NMR spectrum of the benzimidazole moiety. Additionally, in the ^13^C NMR spectrum of compound 15a, broadening of the carbon peaks in the benzimidazole moiety (C-2′, C-3a′, C-4′, C-5′, C-6′, C-7, and C-7a′) was observed, as well. Therefore, the broadening of the corresponding NMR spectral lines reflects dynamic structural transformations in the molecule 15a in solution in CDCl_3_ due to the rapid interconversion of 15aA and 15aB tautomers.^88^

**Fig. 5:**
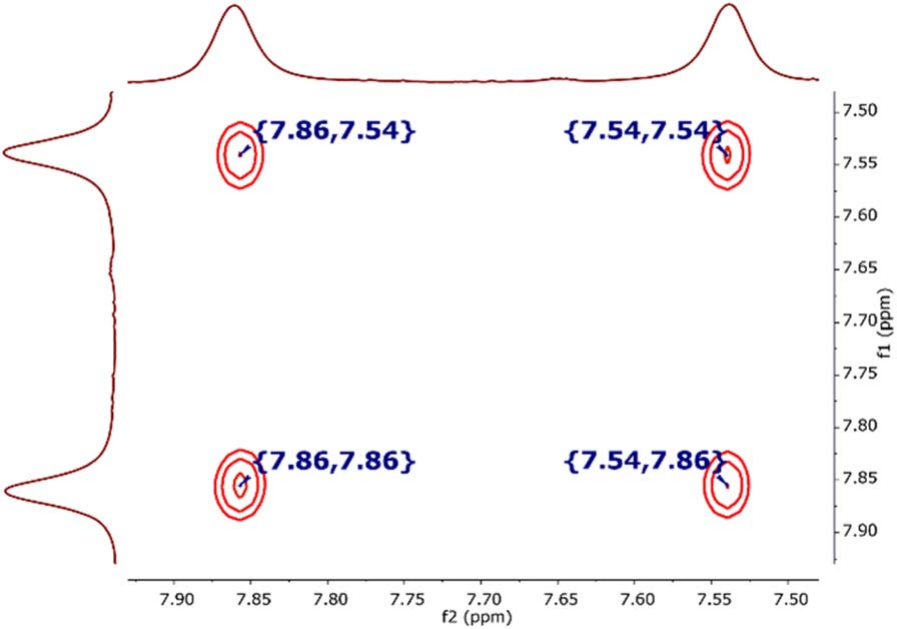
^1^H–^1^H EXSY spectrum (700 MHz, 25  °C, CDCl_3_) for the 4′-H and 7′-H regions of tautomers 15aA and 15aB in CDCl_3_.

Ley *et al.* have demonstrated that selective chemical exchange NMR experiments are highly effective in distinguishing between equilibrating rotamers and non-equilibrating diastereomers.^89^ In such instances, a 1D selective NOESY experiment was suitable for determining the rotamers of Boc-amino acids.^90^ In our case, we decided to use the 1D selective NOESY experiment to determine whether two tautomers exist for compound 15a. When the proton 4-H′ signal, which resonated at *δ* 7.86 ppm, was irradiated, two negative signals of the same phase were observed at *δ* 7.86 ppm (4′-H) and *δ* 7.54 ppm (7′-H) for tautomers 15aA and 15aB (ESI, Fig. S117†). This observation indicated a chemical exchange process in the respective structures. Additionally, we employed 2D EXSY NMR exchange spectroscopy, a unique method that enables the detection of chemical exchange phenomena in real time as an exchange signal.^91,92^ Therefore, the equilibrium between tautomers 15aA and 15aB of compound 15a in CDCl_3_ was confirmed by observing diagonal cross peaks at *δ* 7.86 and *δ* 7.54 ppm in the 2D EXSY spectrum (Fig. 5).

The distinction between the problematic C-10 and C-2′ quaternary carbons was achieved through a comparison of the long-range 2 Hz and 8 Hz optimized ^1^H–^13^C HMBC spectra, where correlations with the NCH_2_ methylene protons (*δ* 4.13 ppm) were easily observed. The 2 Hz optimized ^1^H–^15^N HMBC experiment revealed a correlation between the N-1′ and N-3′ nitrogen (*δ* –137.3 ppm) atoms and the 5′-H and 6′-H protons (*δ* 7.28–7.30 ppm). In contrast, the ^1^H–^15^N HSQC spectral data showed a characteristic proton H–N (*δ* 12.61 ppm) coupling with the N-1′ nitrogen (*δ* −236.3 ppm), allowing unambiguous identification of the benzimidazole moiety. Finally, the NOESY spectra provided additional information about connectivity based on through-space correlations; for instance, a clear NOE was observed between the 9-H and H–N protons, and a subsequent NOE correlation was observed between the H–N and 7′-H (*δ* 7.54 ppm) protons, while in the case of 4′-H (*δ* 7.86 ppm), a sole NOE with 5′-H was observed. The remaining methine carbons in the benzimidazol-2-yl moiety were easily assigned on the base of appropriate correlations in the ^1^H–^13^C HSQC, ^1^H–^13^C H2BC, and ^1^H–^13^C HMBC NMR spectra, and this is in good agreement with spectral data reported in the literature.^85,87^

The IR spectrum of compound 15a exhibited characteristic absorption bands at 3214 cm^−1^ (N–H stretching vibrations) and 1696 cm^−1^ (C=O stretching vibrations) for the benzimidazole and morpholin-2-one moieties, respectively.

**Fig. 6:**
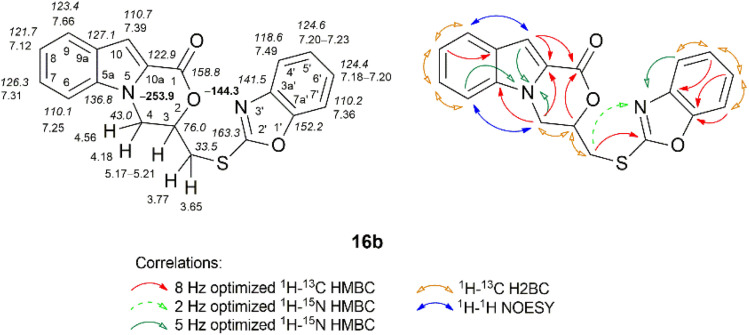
Relevant ^1^H–^13^C HMBC, ^1^H–^13^C H2BC, ^1^H–^15^N HMBC, and ^1^H–^1^H NOESY correlations, as well as ^1^H NMR (italics), ^13^C NMR, and ^15^N NMR (bold) chemical shifts of compound 16b (CDCl_3_).

The key information for the structure elucidation of compound 16b for the benzo[*d*]oxazol-2-yl moiety was obtained from the ^1^H–^13^C HMBC and ^1^H–^15^N HMBC spectra (Fig. 6). Namely, the methylene protons (*δ* 3.65 and 3.77 ppm) of the 8 Hz optimized ^1^H–^13^C HMBC experiment revealed distinct long-range correlations with the quaternary carbon C-2′ (*δ* 163.3 ppm), while the long-range 2 Hz optimized ^1^H–^15^N HMBC experiment showed a correlation of the corresponding protons with the N-3′ nitrogen (*δ* –144.3 ppm). The nitrogen from the oxazino[4,3-*a*]indolone moiety resonated at *δ* –253.9 ppm.

The structure of compound 16b was confirmed by the characteristic absorption band of the carbonyl of the morpholin-2-one moiety at 1716 cm^−1^ (C=O stretching vibrations) in the IR spectrum.

**Fig. 7:**
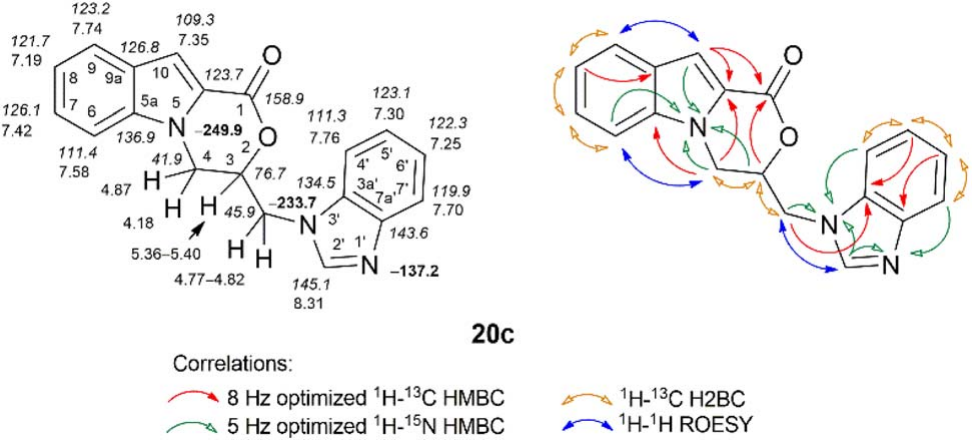
Relevant ^1^H–^13^C HMBC, ^1^H–^13^C H2BC, ^1^H–^15^N HMBC, and ^1^H–^1^H ROESY correlations, as well as ^1^H NMR (italics), ^13^C NMR, and ^15^N NMR (bold) chemical shifts of compound 20c (DMSO-*d*_6_).

Compound 20c has a heterocyclic system in which an oxazino[4,3-*a*]indol-1-one ring is linked by a methylene to a benzo[*d*]imidazole ring at N-3'. The key information required to join different heterocyclic moieties together was obtained through long-range ^1^H–^15^N HMBC correlations and was supported by ^1^H–^1^H ROESY spectral data. The methylene bridge protons (*δ* 4.77–4.82 ppm) revealed ^1^H–^15^N HMBC correlations with the N-3′ nitrogen (*δ* –233.7 ppm) and the through-space ROE correlations with the 2′-H proton (*δ* 8.31 ppm), while the methylene protons (*δ* 4.18 and 4.87 ppm) of oxazino[4,3-*a*]indol-1-one exhibited ^1^H–^15^N HMBC correlations with the N-5 nitrogen (*δ* –249.9 ppm) and a spatial ROE correlation with the 6-H proton (*δ* 7.58 ppm). The N-1′ nitrogen from the benzimidazole moiety resonated at *δ* –137.2 ppm (Fig. 7).

The IR spectrum of compound 20c exhibited an absorption band at 1709 cm^−1^ (C=O stretching vibrations), characteristic of the morpholin-2-one moiety.

### Single-crystal X-ray diffraction analysis

**Fig. 8:**
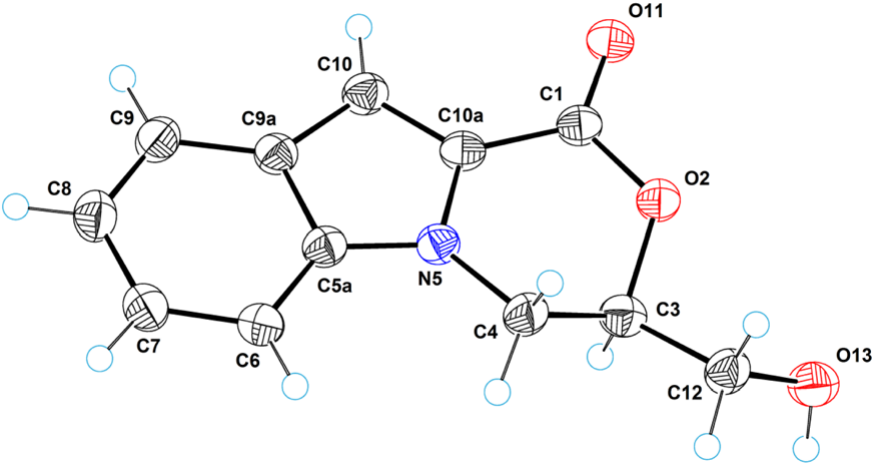
ORTEP diagram for molecule 6a.

An X-ray crystallographic analysis was performed to elucidate the structures of the 3,4-dihydro-1*H*-[1,4]oxazino[4,3-*a*]indol-1-one system containing compounds 6a (ref. 93) and 18 (ref. 94). Fig. 8 gives a perspective view of molecule 6a with thermal ellipsoids and the atom-numbering scheme followed in the text. The tricyclic system is almost planar. The exception is the six-membered oxazine cycle, which has an envelope conformation. The deviation of the C3 atom from the plane of the remaining atoms is equal to 0.615(3) Å. The hydroxymethyl group occupies an equatorial position with respect to the oxazine cycle. A similar situation occurs in the structure of 3-ethenyl-3,4-dihydro-1*H*-[1,4]oxazino[4,3-*a*]indol-1-one,^95^ where the C3 atom has an ethenyl group instead of a hydroxymethyl group.

**Fig. 9:**
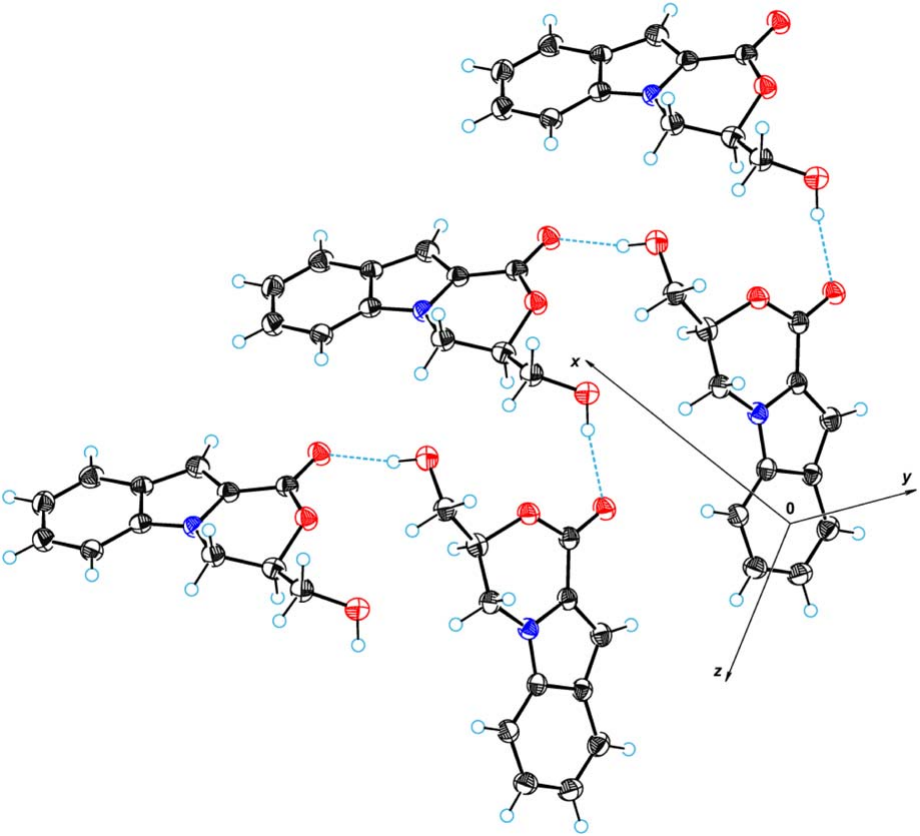
Formation of molecular chains in the crystal structure of 18.

In the crystal structure of 6a, there are strong intermolecular hydrogen bonds of OH⋯O type between the hydroxy group and carbonyl oxygen atom O11. The length of these bonds is 2.866(2) Å (H13⋯O11 = 1.97(2) Å, O13–H13⋯O11 = 169(1)°). Through these hydrogen bonds, the molecular chains are formed in the crystal structure along the crystallographic direction [0 1 1]. Fig. 9 shows a fragment of the molecular chain in the crystal structure. The structure contains a stereogenic carbon atom (C3); however, the crystal structure belongs to the crystallographic rhombic pyramidal class (space group is *Pna*2_1_). This means that the crystals contain *S-* and *R-*enantiomers in a 1 : 1 ratio, *i.e.*, the compound represents a true racemate.

**Fig. 10:**
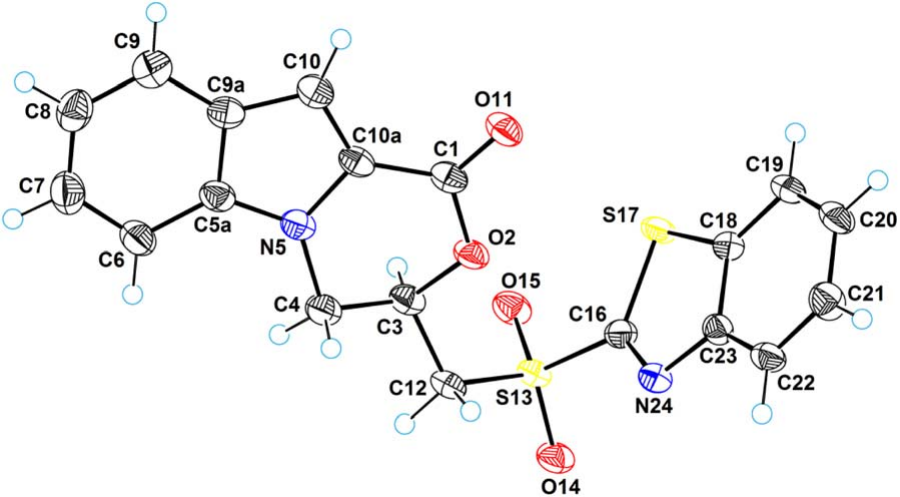
ORTEP diagram for molecule 18.

Fig. 10 illustrates a perspective view of molecule 18. The molecular structure of compound 18 differs from that of 6a in that the position of the hydroxy group is replaced by a benzothiazolylsulfonyl fragment. The value of the C3–C12–S13–C16 torsion angle is 77.5(2)°. There are no intramolecular interactions between the tricyclic system and the benzothiazolylsulfonyl fragment; a fairly high dihedral rotation angle of 66.8(3)° is observed between them. As with 6a, the crystals of 18 are a true racemate (space group is *P*1̄).

### Optical investigations

1*H*-Indole and its derivatives are known for their fluorescence properties.^96–99^ In particular, these compounds have been considered potential fluorometric and colorimetric probes for various biological and analytical applications.^100–104^ For example, Pereira *et al.* prepared and studied methyl 3-aryl-1*H*-indole-2-carboxylates as fluorescent probes for the detection of fluoride ions.^105^ Lu *et al.* designed and synthesized a novel fluorescent probe based on indole-fused 1,8-naphthalimide, which was applied to visualize and discriminate GSH/H2S and Cys/Hcy in living cells.^106^

**Fig. 11:**
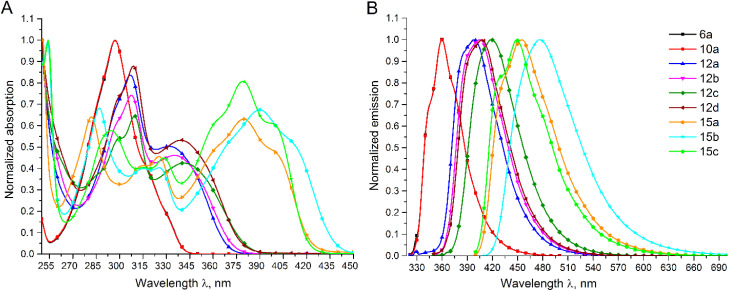
(a) UV-vis absorption spectra of compounds 6a, 10a, 12a–d, and 15a–c in THF; (b) fluorescence emission spectra of compounds 6a, 10a, 12a–d, and 15a–c in THF.

Although many fluorescent indole compounds have been reported, no fluorescent compounds based on the oxazino[4,3-*a*]indole skeleton have been found in the literature. Therefore, we investigated the absorption and fluorescence properties of oxazino[4,3-*a*]indoles 6a, 10a, 12a–d, and 15a–c in THF. The maximum absorption (*λ*_abs_) and emission wavelengths (*λ*_em_), yields (*Φ*_F_) of these oxazino[4,3-*a*]indole derivatives are presented in Table 1. The absorption and normalized fluorescence spectra molar absorption coefficients (*ε*), and fluorescence quantum of 6a, 10a, 12a–d, and 15a–c are presented in Fig. 11a and b.

**Table 1:** Absorption (*λ*_abs_), extinction coefficient (ε), emission (*λ*_em_), Stokes shifts and quantum yield (*Φ*_f_) parameters for compounds 6a, 10a, 12a–d, and 15a–c in THF

Compound	*λ*_abs_, nm	*ε* × 10^3^, dm^3^ mol^−1^ cm^−1^	*λ*_em_, nm	Stokes shift, nm	*Φ*_f_, %
6a	297	0.84	360^a^	63	16
10a	297	1.19	360^a^	63	31
	332	0.37			
12a	307	0.62	398^a^	66	40
	336	0.43			
12b	308	0.69	405^b^	69	44
	341	0.31			
12c	310	0.47	418^b^	77	43
	339	0.38			
12d	309	0.63	408^b^	69	19
	379	0.70			
	325	0.51			
	282	0.71			
15a	250	1.11	455^c^	76	65
	390	0.92			
	325	0.55			
	315	0.54			
	287	0.93			
15b	254	1.36	477^c^	87	75
	379	0.67			
	328	0.37			
15c	294	0.48	449^c^	70	67

a *λ*_ex_ = 300 nm.

b *λ*_ex_ = 330 nm.

c *λ*_ex_ = 380 nm.

In comparison to the oxazino[4,3-*a*]indoles 6a and 10a showing absorption maximum (*λ*_abs_) at 297 nm, 10-substituted oxazino[4,3-*a*]indoles 12a–d and 15a–c exhibited bathochromic shifts to a near ultraviolet band with an *λ*_abs_ in a range of 332–341 nm for 10-(hetero)aryloxazino[4,3-*a*]indoles 12a–d and in a range of 379–390 nm for (benzo[*d*]imidazolyl)oxazino[4,3-*a*]indoles 15a–c (Table 1 and Fig. 11a).

The fluorescence spectra of 10-(hetero)aryloxazino[4,3-*a*]indoles 12a, 12b, 12d displayed similar emission maxima around 400 nm, and Stokes shifts around 70 nm; however, the 10-(*p*-methoxyphenyl)-substituted compound 12c exhibited a significant bathochromic shift of *λ*_em_ at 418 nm as well as a larger Stokes shift of 77 nm. (Benzo[*d*]imidazolyl)oxazino[4,3-*a*]indoles exhibited strong fluorescence with 15a*λ*_em_ at 455 nm and Stokes shifts of 76 nm, while the 5,6-chlorinated analogue 15c exhibited a slight shift in *λ*_em_ of 449 nm and a Stokes shift of 70 nm. However, (benzo[*d*]imidazolyl)oxazino[4,3-*a*]indole 15b, possessing a 5,6-dimethyl substituent, showed a significant bathochromic shift to *λ*_em_ at 477 nm and the largest Stokes shift of 87 nm (Table 1 and Fig. 11b).

The fluorescence quantum yields (*Φ*_F_) of oxazino[4,3-*a*]indoles 6a, 10a, 12a–d, and 15a–c in THF were measured using the integrated sphere method. The structure of the compounds influenced the fluorescence quantum yields. Firstly, for the parent oxazino[4,3-*a*]indole 6a, *Φ*_F_ was estimated to be 16%, while for its methoxy analogue 10a, *Φ*_F_ increased to 31%. The fluorescence quantum yields for 10-(phenyl)oxazino[4,3-*a*]indoles 12a–c remained at 40–44%, but for the 10-(thiophenyl)oxazino[4,3-*a*]indole 12d, the *Φ*_F_ dropped to 19%. (Benzo[*d*]imidazolyl)oxazino[4,3-*a*]indoles 15a–c exhibited the highest fluorescence quantum yields for the compounds 15a and 15c, with *Φ*_F_ values of 65% and 67%, respectively. A more significant increase in the *Φ*_F_ value of 75% was observed for compound 15b.

## Conclusions

To summarize, we have developed a novel synthetic route to create diverse 3,4-dihydro-1*H*-[1,4]oxazino[4,3-*a*]indoles *via* the alkylation of NH-indole-2-carboxylate with tosylated glycerol-1,2-carbonate, followed by hydrolysis and acid-induced cyclization of the intermediate *N*-glycerylated indole-2-carboxylates. Halogenation of the *N*-glycerylindoles led to the formation of 10-iodo-, 10-bromo-, and 10-chloro-3,4-dihydro-1*H*-[1,4]oxazino[4,3-*a*]indole derivatives and further 10-(het)arylation through Pd-catalyzed cross-coupling reactions. In addition, the C-10 nucleophilicity of 3,4-dihydro-1*H*-[1,4]oxazino[4,3-*a*]indol-1-one was implemented in a formylation reaction under Vilsmeier–Haack conditions, and the obtained carbaldehyde was further converted into 10-(1*H*-benzo[*d*]imidazole-2-yl)-3,4-dihydro-1*H*-[1,4]oxazino[4,3-*a*]indol-1-ones. Moreover, the scope of the structural variability was broadened by introducing 3-*O*-, 3-*S*-, or 3-*N*-substituents onto the 3,4-dihydro-1*H*-[1,4]oxazino[4,3-*a*]indol-1-one core. Furthermore, the oxidation of the obtained 3-[(heteroarylthio)methyl]-3,4-dihydro-1*H*-[1,4]oxazino[4,3-*a*]indol-1-ones was investigated, selectively providing the related sulfoxide or sulfone. The structures of the synthesized compounds were confirmed *via* detailed NMR spectroscopy, high-resolution mass spectrometry investigations, and X-ray single-crystal analysis. The selected 3,4-dihydro-1*H*-[1,4]oxazino[4,3-*a*]indoles were characterized by their good quantum yields and significant Stokes shifts.

## Experimental section

### General

All starting materials were purchased from commercial suppliers and were used without further purification unless indicated otherwise. The reaction progress was monitored using thin-layer chromatography (TLC) on pre-coated ALUGRAM®Xtra SIL G/UV_254_ plates. The purification of the reaction mixtures was performed using flash chromatography on a glass column, stationary phase - silica gel (high-purity grade 9385, pore size 60 Å, particle size 70–230 mesh). The ^1^H, ^13^C, ^15^N and ^19^F NMR spectra were recorded in CDCl_3_, CD_3_CN or DMSO-*d*_6_ at 25 °C on a Bruker Avance III 700 spectrometer or a Bruker Avance III 400 spectrometer. All chemical shifts (*δ*) were expressed in ppm, using tetramethylsilane (TMS) as the internal standard. ^15^N chemical shifts were recalculated using a reference of neat external nitromethane standard (coaxial capillary). ^19^F NMR spectra (376 MHz, absolute referencing *via* Ξ ratio) were obtained on a Bruker Avance III 400 using a directly detecting BBO probe. The full and unambiguous assignments of the ^1^H, ^13^C, and ^15^N NMR resonances were achieved using a combination of standard NMR spectroscopic techniques. The following abbreviations were used in reporting the NMR data: *BIM*, benzoimidazole; *BTh*, benzothiazole; *Bu*, butyl; *BZX*, benzoxazole; *Et*, ethyl; *Me*, methyl; *MPh*, morpholine; *Ph*, phenyl; *Py*, pyrazole; *Pyr*, pyrimidine; *Th*, thiophene. The IR spectra were recorded on a Bruker TENSOR 27 spectrometer using pressured KBr pellets. HRMS spectra were recorded on a Bruker maXis quadrupole time-of-flight UHR-Q-TOF mass spectrometer or a Bruker MicrOTOF-Q III mass spectrometer equipped with an electrospray ionization source (ESI). The UV-vis spectra were recorded on a Shimadzu 2600 UV/vis (Shimadzu Corporation, Japan). The fluorescence spectra were recorded on an FL920 fluorescence spectrometer from Edinburgh Instruments (Edinburgh Analytical Instruments Limited, Edinburgh, UK). The PL quantum yields were measured from dilute solutions *via* an absolute method using the Edinburgh Instruments integrating sphere excited with a Xe lamp. X-ray diffraction data were collected at low temperature (160 K) on a Rigaku, XtaLAB Synergy, Dualflex, diffractometer using CuKα radiation (*λ* = 1.54184 Å). The crystal structure was solved by direct methods^107^ and refined by full-matrix least squares with the help of a software package.^108^ Crystal data for 6a: orthorhombic; *a* = 30.9767(6), *b* = 6.3602(2), *c* = 4.9575(1) Å, V = 976.72(4) Å^3^, *Z* = 4, *μ* = 0.889 mm^−1^, *D*_calc_ = 1.4771 g cm^−3^; space group is *Pna*2_1_. Crystal data for 18: *a* = 6.8591(1), *b* = 11.0368(4), *c* = 11.7919(4) Å, *α* = 93.795(3), *β* = 92.677(2), *γ* = 105.124(2)°, *V* = 857.97(5) Å3, *Z* = 2, *μ* = 3.082 mm^−1^, *D*_calc_ = 1.542 g cm^−3^; space group is P′1. For further details, see crystallographic data for 6a and 18 deposited at the Cambridge Crystallographic Data Centre as Supplementary Publication Numbers CCDC 2428119 (for 6a) and CCDC 2428115 (for 18). Copies of the data can be obtained free of charge. The ^1^H and ^13^C NMR spectra, as well as the HRMS data of new compounds, are provided in Fig. S1–S154 (ESI materials†).

### Synthetic procedures

#### General synthetic procedure for 4a–e

A mixture of appropriate ethyl 1*H*-indole-2-carboxylate 3a–e (2.5 mmol) and Cs_2_CO_3_ (1222 mg 3.75 mmol) in anhydrous DMF was stirred at room temperature for 30 min. Then, tosylated glycerol-1,2-carbonate (TGC) (1020 mg 0.75 mmol) was added, and the temperature was raised to 60 °C for 2–3 h. After completion of the reaction (TLC monitoring), the mixture was diluted with water (50 mL) and extracted with ethyl acetate (3 × 50 mL). The combined organic layers were dried over Na_2_SO_4_ and concentrated under vacuum. The obtained residue was purified *via* column chromatography on silica gel (eluent ethyl acetate/hexane, 2 : 5 v/v) to provide products 4a–e.

#### Ethyl 1-[(2-oxo-1,3-dioxolan-4-yl)methyl]-1*H*-indole-2-carboxylate (4a)

Compound 4a was obtained as a white solid, yield 472 mg (65%), mp 113.6–114.1 °C. *R*_f_ = 0.290 (ethyl acetate/hexane, 1 : 2 v/v). ^1^H NMR (700 MHz, CDCl_3_): *δ* = 1.41 (t, *J* = 7.2 Hz, 3H, *Et* CH_3_), 4.25–4.28 (m, 1H, OCH_2_), 4.36 (q, *J* = 7.1 Hz, 2H, *Et* CH_2_), 4.57 (t, *J* = 8.5 Hz, 1H, OCH_2_), 4.85 (d, *J* = 5.1 Hz, 2H, NCH_2_), 5.13–5.16 (m, 1H, OCH), 7.19 (t, *J* = 7.5 Hz, 1H, 5-H), 7.38 (s, 1H, 3-H), 7.38–7.41 (m, 1H, 6-H), 7.46 (d, *J* = 8.5 Hz, 1H, 7-H), 7.68 (d, *J* = 8.0 Hz, 1H, 4-H) ppm. ^13^C NMR (176 MHz, CDCl_3_): *δ* = 14.3 (*Et* CH_3_), 46.3 (NCH_2_), 61.0 (*Et* CH_2_), 66.9 (OCH_2_), 76.7 (OCH), 110.5 (C-7), 112.0 (C-3), 121.5 (C-5), 122.8 (C-4), 126.1 (C-3a), 126.2 (C-6), 127.2 (C-2), 139.8 (C-7a), 154.3 (C=O) 162.5 (COO*Et* C=O) ppm. ^15^N NMR (71 MHz, CDCl_3_): *δ* = −254.7 (N-1) ppm. IR (KBr): 2991 (C–H_aliph_), 1796, 1707 (C=O), 1523, 1479, 1457, 1394, 1306, 1274, 1253 (C=C, C–N_arom_, CH_2,_ CH_3_), 1227, 1198, 1169, 1076 (C–O–C), 1003, 823, 766, 745, 705 (C=C) cm^−1^. MS *m*/*z* (%): 290 ([M + H]^+^, 100). HRMS (ESI) for C_15_H_15_NNaO_5_ ([M + Na]^+^) calculated 312.0842, found 312.0839.

#### Ethyl 5-methyl-1-[(2-oxo-1,3-dioxolan-4-yl)methyl]-1*H*-indole-2-carboxylate (4b)

Compound 4b was obtained as a white solid, yield 477 mg (63%), mp 134.1–134.6 °C. *R*_f_ = 0.385 (ethyl acetate/hexane, 1 : 2 v/v). ^1^H NMR (700 MHz, CDCl_3_): *δ* = 1.41 (t, *J* = 7.2 Hz, 3H, *Et* CH_3_), 2.43 (s, 3H, 5-CH_3_), 4.25 (dd, *J* = 9.0, 7.0 Hz, 1H, OCH_2_), 4.35 (q, *J* = 7.1 Hz, 2H, *Et* CH_2_), 4.55 (dd, *J* = 8.9, 8.1 Hz, 1H, OCH_2_), 4.82 (d, *J* = 5.1 Hz, 2H, NCH_2_), 5.11–5.15, (m, 1H, OCH), 7.22 (dd, *J* = 8.6, 1.5 Hz, 1H, 6-H), 7.28 (s, 1H, 3-H), 7.35 (d, *J* = 8.6 Hz, 1H, 7-H), 7.44 (s, 1H, 4-H) ppm. ^13^C NMR (176 MHz, CDCl_3_): *δ* = 14.3 (*Et* CH_3_), 21.3 (5-CH_3_), 46.3 (NCH_2_), 60.9 (*Et* CH_2_), 66.9 (OCH_2_), 76.7 (OCH), 110.2 (C-7), 111.5 (C-3), 122.0 (C-4), 126.3 (C-3a), 127.1 (C-2), 128.1 (C-6), 130.9 (C-5), 138.3 (C-7a), 154.3 (C=O), 162.5 (COO*Et* C=O) ppm. ^15^N NMR (71 MHz, CDCl_3_): *δ* = −255.6 (N-1) ppm. IR (KBr): 2984, 2946 (C–H_aliph_), 1783, 1698 (C=O), 1524, 1463, 1411, 1372, 1342, 1300, 1262 (C=C, C–N_arom_, CH_2,_ CH_3_), 1205, 1170, 1155, 1128, 1085 (C–O–C), 792, 767, 741 (C=C) cm^−1^. MS *m*/*z* (%): 304 ([M + H]^+^, 100). HRMS (ESI) for C_16_H_17_NNaO_5_ ([M + Na]^+^) calculated 326.0999, found 326.1003.

#### Ethyl 5-bromo-1-[(2-oxo-1,3-dioxolan-4-yl)methyl]-1*H*-indole-2-carboxylate (4c)

Compound 4c was obtained as a white solid, yield 733 mg (80%), mp 132.9–133.4 °C. *R*_f_ = 0.256 (ethyl acetate/hexane, 1 : 2 v/v). ^1^H NMR (700 MHz, CDCl_3_): *δ* = 1.41 (t, *J* = 7.2 Hz, 3H, *Et* CH_3_), 4.25 (dd, *J* = 8.9, 7.3 Hz, 1H, OCH_2_), 4.37 (q, *J* = 7.1 Hz, 2H, *Et* CH_2_), 4.58–4.61 (m, 1H, OCH_2_), 4.79–4.85 (m, 2H, NCH_2_), 5.11–5.14 (m, 1H, OCH), 7.28 (s, 1H, 3-H), 7.34 (d, *J* = 8.9 Hz, 1H, 7-H), 7.45 (d, *J* = 8.9 Hz, 1H, 6-H), 7.79 (s, 1H, 4-H) ppm. ^13^C NMR (176 MHz, CDCl_3_): *δ* = 14.3 (CH_3_), 46.6 (NCH_2_), 61.3 (*Et* CH_2_), 66.8 (OCH_2_), 76.6 (OCH), 111.0 (C-3), 112.1 (C-7), 114.6 (C-5), 125.0 (C-4), 127.5 (C-3a), 128.1 (C-2), 129.0 (C-6), 138.3 (C-7a), 154.1 (C=O), 162.1 (COO*Et* C=O) ppm. ^15^N NMR (71 MHz, CDCl_3_): *δ* = −253.6 (N-1) ppm. IR (KBr): 2987 (C–H_aliph_), 1780, 1702 (C=O), 1514, 1458, 1417, 1394, 1367, 1300, 1274, (C=C, C–N_arom_, CH_2_, CH_3_), 1251, 1222, 1197, 1173, 1078, 1012 (C–O–C), 866, 807, 763, 737, 706 (C=C, C-Br) cm^−1^. MS *m*/*z* (%): 368; 370 ([M + H]^+^, 100). HRMS (ESI) for C_15_H_14_BrNNaO_5_ ([M + Na]^+^) calculated 389.9948, found 389.9952.

#### Ethyl 5-chloro-1-[(2-oxo-1,3-dioxolan-4-yl)methyl]-1*H*-indole-2-carboxylate (4d)

Compound 4d was obtained as a white solid, yield 555 mg (69%), mp 130.0–130.5 °C *R*_f_ = 0.227 (ethyl acetate/hexane, 1 : 2 v/v). ^1^H NMR (700 MHz, CDCl_3_): *δ* = 1.34 (t, *J* = 7.1 Hz, 3H, *Et* CH_3_), 4.19 (dd, *J* = 8.9, 7.3 Hz, 1H, OCH_2_), 4.30 (q, *J* = 7.1 Hz, 2H, *Et* CH_2_), 4.52–4.55 (m, 1H, OCH_2_), 4.74–4.80 (m, 2H, NCH_2_), 5.05–5.09 (m, 1H, OCH), 7.23 (s, 1H, 3-H), 7.27 (dd, *J* = 8.9, 1.8 Hz, 1H, 6-H), 7.33 (d, *J* = 8.9 Hz, 1H, 7-H), 7.57 (s, 1H, 4-H) ppm. ^13^C NMR (176 MHz, CDCl_3_): *δ* = 14.3 (*Et* CH_3_), 46.6 (NCH_2_), 61.3 (*Et* CH_2_), 66.8 (OCH_2_), 76.7 (OCH), 111.2 (C-3), 111.8 (C-7), 121.9 (C-4), 126.6 (C-6), 126.9 (C-3a), 127.2 (C-5), 128.3 (C-2), 138.1 (C-7a), 154.1 (C=O), 162.2 (COO*Et* C=O) ppm. ^15^N NMR (71 MHz, CDCl_3_): *δ* = −253.9 (N-1) ppm. IR (KBr): 2984, 2950 (C–H_aliph_), 1782, 1700 (C=O), 1516, 1452, 1414, 1397, 1373, 1341, 1275, 1263 (C=C, C–N_arom_, CH_2_, CH_3_), 1250, 1199, 1171, 1133, 1087, 1022 (C–O–C), 879, 797, 766, 738 (C=C, C–Cl) cm^−1^. MS *m*/*z* (%): 324 ([M + H]^+^, 100). HRMS (ESI) for C_15_H_14_ClNNaO_5_ ([M + Na]^+^) calculated 346.0453, found 346.0454.

#### Ethyl 5-fluoro-1-[(2-oxo-1,3-dioxolan-4-yl)methyl]-1*H*-indole-2-carboxylate (4e)

Compound 4e was obtained as a white solid, yield 560 mg (73%), mp 132.3–132.8 °C *R*_f_ = 0.186 (ethyl acetate/hexane, 1 : 2 v/v). ^1^H NMR (700 MHz, CD_3_CN): *δ* = 1.36 (t, *J* = 7.1 Hz, 3H, *Et* CH_3_), 4.29–4.31 (m, 1H, OCH_2_), 4.32–4.37 (m, 2H, *Et* CH_2_), 4.58–4.61 (m, 1H, OCH_2_), 4.82 (dd, *J* = 15.5, 3.2 Hz, 1H, NCH_2_), 4.95 (dd, *J* = 15.5, 8.2 Hz, 1H, NCH_2_), 5.08–5.12 (m, 1H, OCH), 7.19 (td, *J* = 9.2, 2.6 Hz, 1H, 6-H), 7.30 (s, 1H, 3-H), 7.39 (dd, *J* = 9.3, 2.5 Hz, 1H, 4-H), 7.56 (dd, *J* = 9.2, 4.3 Hz, 1H, 7-H) ppm. ^13^C NMR (176 MHz, CD_3_CN): *δ* = 14.1 (*Et* CH_3_), 47.0 (NCH_2_), 61.6 (*Et* CH_2_), 67.5 (OCH_2_), 77.0 (OCH), 107.1 (d, ^2^*J*_C,F_ = 23.5 Hz, C-4), 111.2 (d, ^4^*J*_C,F_ = 5.5 Hz, C-3), 113.0 (d, ^3^*J*_C,F_ = 10.0 Hz, C-7), 114.6 (d, ^2^*J*_C,F_ = 27.3 Hz, C-6), 126.7 (d, ^3^*J*_C,F_ = 10.4 Hz, C-3a), 130.0 (C-2), 136.8 (C-7a), 155.2 (C=O), 158.8 (d, ^1^*J*_C,F_ = 235.4 Hz, C-5), 162.3 (COO*Et* C=O) ppm. ^15^N NMR (71 MHz, CD_3_CN): *δ* = −252.2 (N-1) ppm IR (KBr): 3134 (C–H_arom_), 2986 (C–H_aliph_), 1797, 1696 (C=O), 1534, 1476, 1455, 1394, 1374, 1353, 1310, 1294, 1270 (C=C, C–N_arom_, CH_2_, CH_3_, C–F), 1249, 1205, 1116, 1083, 1045, 1015 (C–O–C, C–F), 953, 875, 802, 759, 726 (C=C) cm^−1^. MS *m*/*z* (%): 308 ([M + H]^+^, 100). HRMS (ESI) for C_15_H_14_FNNaO_5_ ([M + Na]^+^) calculated 330.0748, found 330.0747.

#### General synthetic procedure for 5a–e and 8a–c

A 3 M solution of KOH (1.5 mL, 4.5 mmol) was added to a solution of appropriate ethyl 1-[(2-oxo-1,3-dioxolan-4-yl)methyl]-1*H*-indole-2-carboxylate 4a–e or 7a–c (1.5 mmol) in 3 mL of anhydrous EtOH, and the mixture was stirred at 80 °C for 2 h. After completion of the reaction (as monitored by TLC), the mixture was cooled, and 1 M HCl was added dropwise until the pH reached 3–4. The mixture was then diluted with water (20 mL) and extracted with ethyl acetate (3 × 20 mL). The combined organic layers were dried over Na_2_SO_4_ and, without further purification, concentrated under reduced pressure to provide the desired products 5a–e or 8a–c.

#### 1-(2,3-Dihydroxypropyl)-1*H*-indole-2-carboxylic acid (5a)

Compound 5a was obtained as a white solid, yield 317 mg (90%), mp 170.1–170.6 °C. *R*_f_ = 0.157 (methanol/dichloromethane 1 : 10 v/v). ^1^H NMR (400 MHz, DMSO-*d*_6_): *δ* = 3.19–3.56 (m, 5H, OCH_2_, OH), 3.77–3.83 (m, 1H, OCH), 4.50 (dd, *J* = 14.1, 7.7 Hz, 1H, NCH_2_), 4.62–4.93 (m, 2H, NCH_2_, OH), 7.10 (t, *J* = 7.4 Hz, 1H, Ar), 7.22 (s, 1H, 3-H), 7.29 (t, *J* = 7.7 Hz, 1H, Ar), 7.59 (d, *J* = 8.5 Hz, 1H, Ar), 7.65 (d, *J* = 8.0 Hz, 1H, Ar) ppm. ^13^C NMR (101 MHz, DMSO-*d*_6_): *δ* = 47.6 (NCH_2_), 64.4 (OCH_2_), 71.9 (OCH), 110.2, 112.1, 120.7, 122.5, 124.7, 125.9, 129.1, 139.8, 163.5 (C=O) ppm. IR (KBr): 3387, 3275 (OH), 1675 (C=O), 1521, 1485, 1460, 1430, 1360, 1322, 1274, 1227, 1204 (C=C, C–N_arom_, CH_2_), 1138, 1117 (C–O of secondary alcohol), 1053 (C–O of primary alcohol), 964, 899, 813, 747 (C=C) cm^−1^. MS *m*/*z* (%): 236 ([M + H]^+^, 100); 234 ([M–H]^−^). HRMS (ESI) for C_12_H_13_NNaO_4_ ([M + Na]^+^) calculated 258.0737, found 258.0740.

#### 1-(2,3-Dihydroxypropyl)-5-methyl-1*H*-indole-2-carboxylic acid (5b)

Compound 5b was obtained as a white solid, yield 336 mg (90%), mp 186.1–186.6 °C. *R*_f_ = 0.297 (methanol/dichloromethane 1 : 6 v/v). ^1^H NMR (400 MHz, DMSO-*d*_6_): *δ* = 2.37 (s, 3H, CH_3_), 3.28–3.59 (m, 4H, OCH_2_, OH), 3.76–3.81 (m, 1H, OCH), 4.46 (dd, *J* = 14.1, 7.6 Hz, 1H, NCH_2_), 4.58–4.99 (m, 2H, NCH_2_, OH), 7.12–7.13 (m, 2H, Ar), 7.41 (s, 1H, Ar), 7.47 (d, *J* = 8.6 Hz, 1H, Ar) ppm. ^13^C NMR (101 MHz, DMSO-*d*_6_): *δ* = 21.4 (CH_3_), 47.6 (NCH_2_), 64.4 (OCH_2_), 71.9 (OCH), 109.7, 111.9, 121.6, 126.0, 126.6, 129.0, 129.3, 138.4, 163.5 (C=O) ppm. IR (KBr): 3389, 3305 (OH), 2930 (C–H_aliph_), 1675 (C=O), 1527, 1464, 1423, 1361, 1347, 1304, 1274 (C=C, C–N_arom_, CH_2,_ CH_3_), 1171, 1129 (C–O of secondary alcohol), 1051 (C–O of primary alcohol), 965, 921, 865, 819 (C=C) cm^−1^. MS *m*/*z* (%): 250 ([M + H]^+^, 100); 248 ([M–H]^−^). HRMS (ESI) for C_13_H_15_NNaO_4_ ([M + Na]^+^) calculated 272.0893, found 272.0895.

#### 5-Bromo-1-(2,3-dihydroxypropyl)-1*H*-indole-2-carboxylic acid (5c)

Compound 5c was obtained as a white solid, yield 424 mg (90%), mp 186.3–186.8 °C. *R*_f_ = 0.172 (methanol/dichloromethane 1 : 6 v/v). ^1^H NMR (400 MHz, DMSO-*d*_6_): *δ* = 3.33–3.55 (m, 5H, OCH_2_, OH), 3.72–3.83 (m, 1H, OCH), 4.46 (dd, *J* = 14.1, 8.0 Hz, 1H, NCH_2_), 4.63–4.97 (m, 1H, NCH_2_, OH), 7.19 (s, 1H, Ar), 7.40 (d, *J* = 8.9 Hz, 1H, Ar), 7.58 (d, *J* = 9.0 Hz, 1H, Ar), 7.87 (s, 1H, Ar) ppm. ^13^C NMR (101 MHz, DMSO-*d*_6_): *δ* = 47.9 (NCH_2_), 64.3 (OCH_2_), 71.8 (OCH), 109.5, 113.0, 114.5, 124.5, 127.1, 127.5, 130.4, 138.5, 163.2 (C=O) ppm. IR (KBr): 3333 (OH), 3133 (C–H_arom_), 2947 (C–H_aliph_), 1688 (C=O), 1516, 1452, 1422, 1350, 1283, 1248 (C=C, C–N_arom_, CH_2_), 1190, 1110 (C–O of secondary alcohol), 1045 (C–O of primary alcohol), 936, 884, 961, 836, 763, 764, 729 (C=C, C–Br) cm^−1^. MS *m*/*z* (%): 312; 314 ([M–H]^−^). HRMS (ESI) for C_12_H_12_BrNNaO_4_ ([M + Na]^+^) calculated 335.9842, found 335.9844.

#### 5-Chloro-1-(2,3-dihydroxypropyl)-1*H*-indole-2-carboxylic acid (5d)

Compound 5d was obtained as a white solid, yield 323 mg (80%), mp 177.2–177.7 °C. *R*_f_ = 0.216 (methanol/dichloromethane 1 : 6 v/v). ^1^H NMR (400 MHz, DMSO-*d*_6_): *δ* = 3.35–3.66 (m, 3H, OCH_2_, OH), 3.74–3.85 (m, 1H, OCH), 4.48 (dd, *J* = 14.1, 8.0 Hz, 1H, NCH_2_), 4.64–5.00 (m, 2H, NCH_2_, OH), 7.20 (s, 1H, Ar), 7.30 (d, *J* = 8.9 Hz, 1H, Ar), 7.63 (d, *J* = 9.0 Hz, 1H, Ar), 7.73 (s, 1H, Ar) ppm. ^13^C NMR (101 MHz, DMSO-*d*_6_): *δ* = 47.9 (NCH_2_), 64.3 (OCH_2_), 71.8 (OCH), 109.6, 114.1, 121.4, 124.7, 125.1, 126.8, 130.5, 138.3, 163.2 (C=O) ppm. IR (KBr): 3348 (OH), 2948, 2912 (C–H_aliph_), 1687 (C=O), 1517, 1454, 1424, 1351, 1283, 1249 (C=C, N–C_arom_, CH_2_), 1190, 1108 (C–O of secondary alcohol), 1046 (C–O of primary alcohol), 937, 860, 837, 795 (C=C, C–Cl) cm^−1^. MS *m*/*z* (%): 268 ([M–H]^−^). HRMS (ESI) for C_12_H_12_ClNNaO_4_ ([M + Na]^+^) calculated 292.0347, found 292.0350.

#### 1-(2,3-Dihydroxypropyl)-5-fluoro-1*H*-indole-2-carboxylic acid (5e)

Compound 5e was obtained as a white solid, yield 312 mg (82%), mp 171.8–172.3 °C. *R*_f_ = 0.312 (methanol/dichloromethane 1 : 6 v/v). ^1^H NMR (400 MHz, DMSO-*d*_6_): *δ* = 3.34–3.61 (m, 3H, OCH_2_, OH), 3.73–3.85 (m, 1H, OCH), 4.49 (dd, *J* = 14.1, 7.9 Hz, 1H, NCH_2_), 4.64–5.02 (m, 2H, NCH_2_, OH), 7.16 (dd, *J* = 9.2, 1.9 Hz, 1H, Ar), 7.20 (s, 1H, Ar), 7.42 (dd, *J* = 9.4, 1.8 Hz, 1H, Ar), 7.62 (dd, *J* = 9.1, 4.3 Hz, 1H, Ar) ppm. ^13^C NMR (101 MHz, DMSO-*d*_6_): *δ* = 47.9 (NCH_2_), 64.3 (OCH_2_), 71.9 (OCH), 106.4 (d, ^2^*J*_C,F_ = 23.0 Hz), 109.9 (d, ^4^*J*_C,F_ = 5.2 Hz), 113.5 (d, ^2^*J*_C,F_ = 26.7 Hz), 113.7 (d, ^3^*J*_C,F_ = 9.6 Hz), 125.8 (d, ^3^*J*_C,F_ = 10.4 Hz), 130.7, 136.6, 157.8 (d, ^1^*J*_C,F_ = 233.7 Hz), 163.3 (C=O) ppm. IR (KBr): 3358 (OH), 2966 (C–H_aliph_), 1688 (C=O), 1525, 1462, 1417, 1352, 1253, 1190, 1170 (C=C, C–N_arom_, CH_2,_ C–F), 1106 (C–O of secondary alcohol), 1048 (C–O of primary alcohol), 966, 927, 849, 797, 760, 723 (C=C) cm^−1^. MS *m*/*z* (%): 252 ([M–H]^−^) HRMS (ESI) for C_12_H_12_FNNaO_4_ ([M + Na]^+^) calculated 276.0643, found 276.0646.

#### 1-(2,3-Dihydroxypropyl)-3-iodo-1*H*-indole-2-carboxylic acid (8a)

Compound 8a was obtained as a white solid, yield 504 mg (93%), mp 154.4–154.9 °C. *R*_f_ = 0.156 (methanol/dichloromethane 1 : 6 v/v). ^1^H NMR (400 MHz, DMSO-*d*_6_): *δ* = 3.22–3.58 (m, 4H, OCH_2_, OH), 3.63–3.79 (m, 1H, OCH), 4.44–4.58 (m, 1H, NCH_2_), 4.64–4.98 (m, 2H, NCH_2_, OH), 7.13–7.28 (m, 1H, Ar), 7.30–7.40 (m, 1H, Ar), 7.43 (d, *J* = 5.5 Hz, 1H, Ar), 7.59 (d, *J* = 6.1 Hz, 1H, Ar) ppm. ^13^C NMR (101 MHz, DMSO-*d*_6_): *δ* = 48.6 (NCH_2_), 64.3 (OCH_2_), 66.6 (C-3), 71.7 (OCH), 112.5, 121.6, 123.1, 125.6, 130.0, 131.2, 139.0, 163.0 (C=O) ppm. IR (KBr): 3351 (OH), 2923 (C–H_aliph_), 1671 (C=O), 1496, 1479, 1454, 1406, 1349, 1243 (C=C, C–N_arom_, CH_2_), 1194, 1107 (C–O of secondary alcohol), 1027 C–O of primary alcohol), 941, 862, 733 (C=C), 556 (C–I) cm^−1^. MS *m*/*z* (%): 360 ([M–H]^−^). HRMS (ESI) for C_12_H_13_INO_4_ ([M + H]^+^) calculated 361.9884, found 361.9891.

#### 3-Bromo-1-(2,3-dihydroxypropyl)-1*H*-indole-2-carboxylic acid (8b)

Compound 8b was obtained as a white solid, yield 443 mg (94%), mp 161.2–161.7 °C. *R*_f_ = 0.110 (methanol/dichloromethane 1 : 6 v/v). ^1^H NMR (700 MHz, DMSO-*d*_6_): *δ* = 3.30–3.36 (m, 3H, OCH_2_), 3.70–3.74 (m, 1H, OCH), 4.50 (dd, *J* = 14.4, 8.2 Hz, 1H, NCH_2_), 4.65 (dd, *J* = 14.4, 3.9 Hz, 1H, NCH_2_), 4.76 (br s, 1H, OH) 7.22 (t, *J* = 7.7 Hz, 1H, Ar), 7.38 (t, *J* = 8.2 Hz, 1H, Ar), 7.54 (d, *J* = 8.0 Hz, 1H, Ar), 7.63 (d, *J* = 8.5 Hz, 1H, Ar) ppm. ^13^C NMR (176 MHz, DMSO-*d*_6_): *δ* = 47.8 (NCH_2_), 63.7 (OCH_2_), 71.1 (OCH), 96.0, 112.0, 120.0, 121.1, 125.2, 125.7, 127.2, 137.5, 162.1 (C=O) ppm. IR (KBr): 3350 (OH), 2966 (C–H_aliph_), 1686 (C=O), 1505, 1410, 1351, 1326, 1252 (C=C, C–N_arom_, CH_2_), 1179, 1097 (C–O of secondary alcohol), 1045 (C–O of primary alcohol), 969, 945, 860 (C=C), 743 (C–Br) cm^−1^. MS *m*/*z* (%): 312, 314 ([M–H]^−^). HRMS (ESI) for C_12_H_12_BrNNaO_4_ ([M + Na]^+^) calculated 335.9842, found 335.9842.

#### 3-Chloro-1-(2,3-dihydroxypropyl)-1*H*-indole-2-carboxylic acid (8c)

Compound 8c was obtained as a white solid, yield 347 mg (86%), mp 158.6–159.1 °C. *R*_f_ = 0.203 (methanol/dichloromethane 1 : 6 v/v). ^1^H NMR (400 MHz, DMSO-*d*_6_): *δ* = 3.30–3.38 (m, 2H, OCH_2_), 3.68–3.78 (m, 1H, OCH), 4.50 (dd, *J* = 14.3, 8.1 Hz, 1H, NCH_2_), 4.64 (dd, *J* = 14.3, 3.5 Hz, 1H, NCH_2_), 7.22 (t, *J* = 7.5 Hz, 1H, Ar), 7.38 (t, *J* = 7.6 Hz, 1H, Ar), 7.60–7.65 (m, 2H, Ar) ppm. ^13^C NMR (101 MHz, DMSO-*d*_6_): *δ* = 48.2 (NCH_2_), 64.3 (OCH_2_), 71.7 (OCH), 110.1, 112.5, 119.4, 121.5, 124.5, 125.8, 126.1, 137.3, 162.5 (C=O) ppm. IR (KBr): 3405 (OH), 2967 (C–H_aliph_), 1688 (C=O), 1509, 1458, 1417, 1351, 1329, 1250 (C=C, C–N_arom_, CH_2_), 1181, 1096 (C–O of secondary alcohol), 1046 (C–O of primary alcohol), 955, 918, 875, 833, 743, 669, 628 (C=C, C–Cl) cm^−1^. MS *m*/*z* (%): 268 ([M–H]^−^). HRMS (ESI) for C_12_H_12_ClNNaO_4_ ([M + Na]^+^) calculated 292.0347, found 292.0345.

#### General synthetic procedure for 6a–e

Anhydrous *p*-toluenesulfonic acid (4 mg, 0.02 mmol) was added to a solution of appropriate 1-(2,3-dihydroxypropyl)-2-carboxylic acid 5a–e (1 mmol) in anhydrous toluene, and the mixture was refluxed for 16 h. After completion of the reaction (TLC monitoring), the mixture was concentrated under reduced pressure and the obtained residue was purified *via* column chromatography on silica gel (methanol/dichloromethane, 3 : 100 v/v) to provide the desired products 6a–e.

#### 3-(Hydroxymethyl)-3,4-dihydro-1*H*-[1,4]oxazino[4,3-*a*]indol-1-one (6a)

Compound 6a was obtained as a white solid, yield 195 mg (90%), mp 165.3–165.8 °C. *R*_f_ = 0.312 (ethyl acetate/hexane, 2 : 1 v/v). ^1^H NMR (700 MHz, DMSO-*d*_6_): *δ* = 3.74–3.79 (m, 2H, OCH_2_), 4.21 (dd, *J* = 12.9, 9.9 Hz, 1H, NCH_2_), 4.63 (dd, *J* = 13.0, 3.5 Hz, 1H, NCH_2_), 4.88–4.91 (m, 1H, OCH), 5.33 (t, *J* = 5.7 Hz, 1H, OH), 7.17–7.19 (m, 1H, 8-H), 7.34 (s, 1H, 10-H), 7.40 (t, *J* = 7.7 Hz, 1H, 7-H), 7.58–7.62 (m, 1H, 6-H), 7.75 (d, *J* = 8.1 Hz, 1H, 9-H) ppm. ^13^C NMR (176 MHz, DMSO-*d*_6_): *δ* = 40.7 (NCH_2_), 60.6 (OCH_2_), 78.3 (OCH), 108.2 (C-10), 111.0 (C-6), 121.0 (C-8), 122.5 (C-9), 123.4 (C-10a), 125.3 (C-7), 126.3 (C-9a), 136.3 (C-5a), 159.1 (C=O) ppm. ^15^N NMR (71 MHz, DMSO-*d*_6_): −249.2 (N-5) ppm. IR (KBr): 3403 (OH), 3049 (C–H_arom_), 2936 (C–H_aliph_), 1694 (C=O), 1537, 1467, 1413, 1381, 1348, 1321 (C=C, C–N_arom,_ CH_2_), 1256, 1205, 1168, 1139, 1099, 1081 (C–O–C), 1049 (C–O of primary alcohol), 958, 892, 807, 736 (C=C) cm^−1^. MS *m*/*z* (%): 218 ([M + H]^+^, 100). HRMS (ESI) for C_12_H_12_NO_3_ ([M + H]^+^) calculated 218.0812, found 218.0815.

#### 3-(Hydroxymethyl)-8-methyl-3,4-dihydro-1*H*-[1,4]oxazino[4,3-*a*]indol-1-one (6b)

Compound 6b was obtained as a white solid, yield 206 mg (89%), mp 172.8–173.3 °C. *R*_f_ = 0.193 (ethyl acetate/hexane, 2 : 1 v/v). ^1^H NMR (700 MHz, DMSO-*d*_6_): *δ* = 2.40 (s, 3H, CH_3_), 3.72–3.77 (m, 2H, OCH_2_), 4.17 (dd, *J* = 12.9, 9.9 Hz, 1H, NCH_2_), 4.59 (dd, *J* = 12.9, 3.5 Hz, 1H, NCH_2_), 4.86–4.89 (m, 1H, OCH), 5.29 (t, *J* = 5.7 Hz, 1H, OH), 7.22–7.24 (m, 2H, 6-H; 7-H), 7.49–7.51 (m, 2H, 9-H; 10-H) ppm. ^13^C NMR (176 MHz, DMSO-*d*_6_): *δ* = 21.0 (CH_3_), 40.7 (NCH_2_), 60.6 (OCH_2_), 78.3 (OCH), 107.5 (C-6), 110.7 (C-10), 121.5 (C-9), 123.3 (C-10a), 126.5 (C-9a), 127.3 (C-7), 129.8 (C-8), 134.9 (C-5a), 159.1 (C=O) ppm. ^15^N NMR (71 MHz, DMSO-*d*_6_): *δ* = −249.8 (N-5) ppm. IR (KBr): 3411 (OH), 3043 (CH_arom_), 2941 (CH_aliph_), 1695 (C=O), 1544, 1484, 1466, 1436, 1378, 1346, 1300 (C=C, C–N_arom_, CH_2_, CH_3_), 1258, 1206, 1146, 1129, 1096 (C–O–C), 1050 (C–O of primary alcohol), 957, 874, 803, 757, 736 (C=C) cm^−1^. MS *m*/*z* (%): 232 ([M + H]^+^, 100). HRMS (ESI) for C_13_H_13_NNaO_3_ ([M + Na]^+^) calculated 254.0788, found 254.0789.

#### 8-Bromo-3-(hydroxymethyl)-3,4-dihydro-1*H*-[1,4]oxazino[4,3-*a*]indol-1-one (6c)

Compound 6c was obtained as a white solid, yield 258 mg (87%), mp 179.6–180.1 °C. *R*_f_ = 0.203 (ethyl acetate/hexane, 2 : 1 v/v). ^1^H NMR (700 MHz, DMSO-*d*_6_): *δ* = 3.72–3.77 (m, 2H, OCH_2_), 4.22 (dd, *J* = 13.0, 9.9 Hz, 1H, NCH_2_), 4.65 (dd, *J* = 13.0, 3.5 Hz, 1H, NCH_2_), 4.90–4.93 (m, 1H, OCH), 5.31 (t, *J* = 5.7 Hz, 1H, OH), 7.30 (s, 1H, 10-H), 7.51 (dd, *J* = 8.9, 1.9 Hz, 1H, 7-H), 7.62 (d, *J* = 8.9 Hz, 1H, 6-H), 7.97 (d, *J* = 1.8 Hz, 1H, 9-H) ppm. ^13^C NMR (176 MHz, DMSO-*d*_6_): *δ* = 40.9 (NCH_2_), 60.6 (OCH_2_), 78.3 (OCH), 107.4 (C-10), 113.2 (C-8), 113.3 (C-6), 124.6 (C-9; C-10a), 127.8 (C-7; C-9a), 134.9 (C-5a), 158.8 (C=O) ppm. ^15^N NMR (71 MHz, DMSO-*d*_6_): *δ* = −247.5 (N-5) ppm. IR (KBr): 3243 (OH), 2939 (C–H_aliph_), 1732 (C=O), 1536, 1474, 1436, 1415, 1378, 1275 (C=C, C–N_arom_, CH_2_), 1244, 1190, 1163, 1109, 1086 (C–O–C), 1066, 1041 (C–O of primary alcohol), 959, 902, 860, 796, 752, 730, 679 (C=C, C–Br) cm^−1^. MS *m*/*z* (%): 296; 298 ([M + H]^+^, 100). HRMS (ESI) for C_12_H_10_BrNNaO_3_ ([M + Na]^+^) calculated 317.9736, found 317.9734.

#### 8-Chloro-3-(hydroxymethyl)-3,4-dihydro-1*H*-[1,4]oxazino[4,3-*a*]indol-1-one (6d)

Compound 6d was obtained as a white solid, yield 213 mg (85%), mp 181.8–182.3 °C. *R*_f_ = 0.137 (ethyl acetate/hexane, 2 : 1 v/v). ^1^H NMR (700 MHz, DMSO-*d*_6_): *δ* = 3.72–3.77 (m, 2H, OCH_2_), 4.22 (dd, *J* = 13.0, 9.9 Hz, 1H, NCH_2_), 4.65 (dd, *J* = 13.0, 3.5 Hz, 1H, NCH_2_), 4.90–4.93 (m, 1H, OCH), 5.31 (t, *J* = 5.7 Hz, 1H, OH), 7.30 (s, 1H, 10-H), 7.40 (dd, *J* = 8.9, 2.1 Hz, 1H, 7-H), 7.67 (d, *J* = 8.9 Hz, 1H, 6-H), 7.82 (d, *J* = 2.0 Hz, 1H, 9-H) ppm. ^13^C NMR (176 MHz, DMSO-*d*_6_): *δ* = 40.9 (NCH_2_), 60.6 (OCH_2_), 78.4 (OCH), 107.5 (C-10), 112.9 (C-6), 121.5 (C-8; C-9), 124.8 (C-10a), 125.4 (C-7), 127.1 (C-9a), 134.7 (C-5a), 158.8 (C=O) ppm. ^15^N NMR (71 MHz, DMSO-*d*_6_): *δ* = −248.2 (N-5) ppm. IR (KBr): 3253 (OH), 2941 (C–H_aliph_), 1733 (C=O), 1537, 1475, 1436, 1415, 1378, 1346, 1275 (C=C, C–N_arom_, CH_2_), 1245, 1191, 1164, 1086 (C–O–C), 1063, 1041 (C–O of primary alcohol), 960, 910, 861, 796, 752, 730, 692 (C=C, C–Cl) cm^−1^ MS *m*/*z* (%): 252 ([M + H]^+^, 100). HRMS (ESI) for C_12_H_10_ClNNaO_3_ ([M + Na]^+^) calculated 274.0241, found 274.0243.

#### 8-Fluoro-3-(hydroxymethyl)-3,4-dihydro-1*H*-[1,4]oxazino[4,3-*a*]indol-1-one (6e)

Compound 6e was obtained as a white solid, yield 202 mg (86%), mp 181.0–181.5 °C. *R*_f_ = 0.183 (ethyl acetate/hexane, 2 : 1 v/v). ^1^H NMR (700 MHz, DMSO-*d*_6_): *δ* = 3.73–3.78 (m, 2H, OCH_2_), 4.22 (dd, *J* = 13.0, 9.9 Hz, 1H, NCH_2_), 4.65 (dd, *J* = 13.0, 3.5 Hz, 1H, NCH_2_) 4.89–4.92 (m, 1H, OCH), 5.32 (t, *J* = 5.7 Hz, 1H, OH), 7.29 (td, *J* = 9.2, 2.5 Hz, 1H, 7-H), 7.31 (s, 1H, 10-H), 7.52 (dd, *J* = 9.6, 2.5 Hz, 1H, 7-H), 7.67 (dd, *J* = 9.1, 4.4 Hz, 1H, 6-H) ppm. ^13^C NMR (176 MHz, DMSO-*d*_6_): *δ* = 40.9 (NCH_2_), 60.6 (OCH_2_), 78.4 (OCH), 106.5 (d, ^2^*J*_C,F_ = 23.4 Hz, C-9), 107.9 (d, ^4^*J*_C,F_ = 5.6 Hz, C-10), 112.6 (d, ^3^*J*_C,F_ = 9.7 Hz, C-6), 114.4 (d, ^2^*J*_C,F_ = 27.1 Hz, C-7), 124.9 (C-10a), 126.2 (d, ^3^*J*_C,F_ = 10.8 Hz, C-9a), 133.2 (C-5a), 157.5 (d, ^1^*J*_C,F_ = 235.0 Hz, C-8), 158.8 (C=O) ppm. ^15^N NMR (71 MHz, DMSO-*d*_6_): *δ* = −249.1 (N-5) ppm. ^19^F NMR (376 MHz, DMSO-*d*_6_): *δ* = −122.0 (F-8) ppm. IR (KBr): 3401 (OH), 1693 (C=O), 1539, 1466, 1386, 1352, 1284 (C=C, C–N_arom_, CH_2_, C–F), 1241, 1202, 1124 (C–O–C), 1076 (C–O of primary alcohol), 939, 865, 805, 753, 731, 661 (C=C) cm^−1^. MS *m*/*z* (%): 236 ([M + H]^+^, 100). HRMS (ESI) for C_12_H_10_FNNaO_3_ ([M + Na]^+^) calculated 258.0537, found 258.0539.

#### General synthetic procedure for 7a and 11

NIS (146 mg, 0.65 mmol) was added to a solution of ethyl 1-[(2-oxo-1,3-dioxolan-4-yl)methyl]-1*H*-indole-2-carboxylate 4a (144 mg, 0.5 mmol) or 3-(methoxymethyl)-3,4-dihydro-1*H*-[1,4]oxazino[4,3-*a*]indol-1-one 10a (115 mg, 0.5 mmol) in chloroform, and the mixture was stirred at room temperature for 24 h. After completion of the reaction (TLC monitoring), the mixture was diluted with water (10 mL) and extracted with ethyl acetate (3 × 10 mL). The combined organic layers were dried over Na_2_SO_4_ and concentrated under vacuum. The obtained residue was purified *via* column chromatography on silica gel (eluent ethyl acetate/hexane, 1 : 2 v/v) to provide product 7a or 11.

#### Ethyl 3-iodo-1-[(2-oxo-1,3-dioxolan-4-yl)methyl]-1*H*-indole-2-carboxylate (7a)

Compound 7a was obtained as a white solid, yield 189 mg (91%), mp 128.1–128.6 °C. *R*_f_ = 0.461 (acetone/hexane 1 : 2 v/v). ^1^H NMR (400 MHz, CDCl_3_): *δ* = 1.51 (t, *J* = 7.1 Hz, 3H, *Et* CH_3_), 4.23–4.27 (m, 1H, OCH_2_), 4.45 (q, *J* = 7.1 Hz, 2H, *Et* CH_2_), 4.58–4.63 (m, 1H, OCH_2_), 4.82–4.83 (m, 2H, NCH_2_), 5.12–5.18 (m, 1H, OCH), 7.26–7.29 (m, 1H, Ar), 7.41–7.46 (m, 2H, Ar), 7.58 (d, *J* = 8.1 Hz, 1H, Ar) ppm. ^13^C NMR (101 MHz, CDCl_3_): *δ* = 14.2 (*Et* CH_3_), 47.6 (NCH_2_), 61.8 (*Et* CH_2_), 66.9 (OCH_2_), 69.5 (C-3), 76.8 (OCH), 110.6, 122.4, 124.3, 127.3, 127.7, 130.5, 139.0, 154.1 (C=O), 161.7 (COO*Et* C=O) ppm. IR (KBr): 2980 (C–H_aliph_), 1784, 1704 (C=O), 1497, 1478, 1478, 1454, 1379, 1309 (C=C, C–N_arom_, CH_2_, CH_3_), 1249, 1170, 1131, 1080, 1019 (C–O–C), 766, 749 (C=C) cm^−1^. MS *m*/*z* (%): 416 ([M + H]^+^, 100). HRMS (ESI) for C_15_H_14_INNaO_5_ ([M + Na]^+^) calculated 437.9809, found 437.9805.

#### 10-Iodo-3-(methoxymethyl)-3,4-dihydro-1*H*-[1,4]oxazino[4,3-*a*]indol-1-one (11)

Compound 11 was obtained as white solid, yield 154 mg (86%), mp 112.0–112.5 °C. *R*_f_ = 0.231 (acetone/hexane 1 : 3 v/v). ^1^H NMR (400 MHz, CDCl_3_): *δ* = 3.46 (s, 3H, CH_3_), 3.72 (dd, *J* = 10.2, 6.6 Hz, 1H, OCH_2_), 3.82 (dd, *J* = 10.2, 4.3 Hz, 1H, OCH_2_), 4.23 (dd, *J* = 12.8, 9.7 Hz, 1H, NCH_2_), 4.51 (dd, *J* = 12.8, 3.4 Hz, 1H, NCH_2_), 4.85–4.91 (m, 1H, OCH), 7.28–7.34 (m, 2H, Ar), 7.46 (t, *J* = 7.7 Hz, 1H), 7.60 (d, *J* = 8.2 Hz, 1H) ppm. ^13^C NMR (101 MHz, CDCl_3_): *δ* = 42.5 (NCH_2_), 59.7 (OCH_2_), 68.0 (C-10), 71.3 (OCH_3_), 75.5 (OCH), 110.3, 122.3, 122.4, 124.0, 127.4, 130.9, 136.7, 158.1 (C=O) ppm. IR (KBr): 2985, 2880, 2810 (C–H_aliph_), 1716 (C=O), 1511, 1469, 1410, 1383, 1349 1313 (C=C, C–N_arom_, CH_2_, CH_3_), 1242, 1206, 1156, 1108 (C–O–C), 967, 755, 741 (C=C) cm^−1^. MS *m*/*z* (%): 358 ([M + H]^+^, 100). HRMS (ESI) for C_13_H_12_INNaO_3_ ([M + Na]^+^) calculated 379.9754, found 379.9750.

#### Synthetic procedure for 7b

NBS (115 mg, 0.65 mmol) was added to a solution of ethyl 1-[(2-oxo-1,3-dioxolan-4-yl)methyl]-1*H*-indole-2-carboxylate 4a (144 mg, 0.5 mmol) and DABCO (5 mg, 0.05 mmol) in chloroform, and the mixture was stirred at ambient temperature for 24 h. After completion of the reaction (TLC monitoring), the mixture was diluted with water (10 mL) and extracted with ethyl acetate (3 × 10 mL). The combined organic layers were dried over Na_2_SO_4_ and concentrated under vacuum. The obtained residue was purified *via* column chromatography on silica gel (eluent ethyl acetate/hexane, 1 : 2 v/v) to provide product 7b.

#### Ethyl 3-bromo-1-[(2-oxo-1,3-dioxolan-4-yl)methyl]-1*H*-indole-2-carboxylate (7b)

Compound 7b was obtained as a white solid, yield 162 mg (88%), mp 144.8–145.3 °C. *R*_f_ = 0.125 (acetone/hexane 1 : 3 v/v). ^1^H NMR (400 MHz, CDCl_3_): *δ* = 1.40 (t, *J* = 7.1 Hz, 3H, *Et* CH_3_), 4.15–4.19 (m, 1H, OCH_2_), 4.35 (q, *J* = 7.1 Hz, 2H, *Et* CH_2_), 4.50–4.55 (m, 1H, OCH_2_), 4.68–4.75 (m, 2H, NCH_2_), 5.03–5.09 (m, 1H, OCH), 7.18–7.21 (m, 1H, Ar), 7.36–7.37 (m, 2H, Ar), 7.61 (d, *J* = 8.1 Hz, 1H, Ar) ppm. ^13^C NMR (101 MHz, CDCl_3_): *δ* = 14.2 (*Et* CH_3_), 47.3(NCH_2_), 61.7 (*Et* CH_2_), 66.9 (OCH_2_), 76.8 (OCH), 101.1 (C-3), 110.6, 121.8, 122.2, 124.6, 127.0, 127.4, 138.3, 154.2 (C=O), 161.8 (COO*Et* C=O) ppm. IR (KBr): 2985 (C–H_aliph_), 1808, 1697 (C=O), 1508, 1454, 1394, 1312, 1275 (C=C, C–N_arom_, CH_2_, CH_3_), 1249, 1175, 1132, 1111, 1086, 1049, 1022 (C–O–C), 764, 742 (C-Br) cm^−1^. MS *m*/*z* (%): 368; 370 ([M + H]^+^, 100). HRMS (ESI) for C_15_H_14_BrNNaO_5_ ([M + Na]^+^) calculated 389.9948, found 389.9945.

#### Synthetic procedure for 7c

NCS (87 mg, 0.65 mmol) was added to a solution of ethyl 1-[(2-oxo-1,3-dioxolan-4-yl)methyl]-1*H*-indole-2-carboxylate 4a (144 mg, 0.5 mmol) and DABCO (5 mg, 0.05 mmol) in chloroform, and the mixture was stirred at ambient temperature for 24 h. After completion of the reaction (TLC monitoring), the mixture was diluted with water (10 mL) and extracted with ethyl acetate (3 × 10 mL). The combined organic layers were dried over Na_2_SO_4_ and concentrated under vacuum. The obtained residue was purified *via* column chromatography on silica gel (eluent ethyl acetate/hexane, 1 : 2 v/v) to provide product 7c.

#### Ethyl 3-chloro-1-[(2-oxo-1,3-dioxolan-4-yl)methyl]-1*H*-indole-2-carboxylate (7c)

Compound 7c was obtained as a white solid, yield 145 mg (90%), mp 133.3–133.8 °C. *R*_f_ = 0.141 (acetone/hexane 1 : 3 v/v). ^1^H NMR (400 MHz, CDCl_3_): *δ* = 1.46 (t, *J* = 7.1 Hz, 3H, *Et* CH_3_), 4.23–4.27 (m, 1H, OCH_2_), 4.43 (q, *J* = 7.1 Hz, 2H, *Et* CH_2_), 4.58–4.62 (m, 1H, OCH_2_), 4.74–4.82 (m, 2H, NCH_2_), 5.11–5.17 (m, 1H, OCH), 7.24–7.28 (m, 1H, Ar), 7.41–7.46 (m, 2H, Ar), 7.72 (d, *J* = 8.1 Hz, 1H, Ar) ppm. ^13^C NMR (101 MHz, CDCl_3_): *δ* = 14.2 (*Et* CH_2_), 47.1 (NCH_2_), 61.6 (*Et* CH_2_), 66.8 (OCH_2_), 76.8 (OCH), 110.6, 115.0 (C-3), 120.5, 122.0, 122.8, 125.2, 127.4, 137.7, 154.2 (C=O), 161.8 (COO*Et* C=O) ppm. IR (KBr): 2988 (C–H_aliph_), 1812, 1697 (C=O), 1510, 1457, 1403, 1354, 1316, 1282 (C=C, CH_2_, CH_3,_ C–N_arom_), 1251, 1204, 1176, 1086, 1025 (C–O–C), 752 (C–Cl) cm^−1^. MS *m*/*z* (%): 324 ([M + H]^+^, 100). HRMS (ESI) for C_15_H_14_ClNNaO_5_ ([M + Na]^+^) calculated 346.0453, found 346.0458.

#### General synthetic procedure for 9a–c

To an ice-cold solution of the appropriate 1-(2,3-dihydroxypropyl)-2-carboxylic acid 8a–c (1 mmol) in anhydrous DCM, EDC·HCl (192 mg 1.2 mmol) and DMAP (244 mg, 2 mmol) were added, stirring was maintained for 15 min, and then reaction mixture was then to room temperature and stirred for 3 h. After completion of the reaction (TLC monitoring), the mixture was concentrated under reduced pressure and the obtained residue was purified *via* column chromatography on silica gel (methanol/dichloromethane, 3 : 100 v/v) to provide the desired products 9a–c.

#### 3-(Hydroxymethyl)-10-iodo-3,4-dihydro-1*H*-[1,4]oxazino[4,3-*a*]indol-1-one (9a)

Compound 9a was obtained as a white solid, yield 264 mg (77%), mp 159.3–159.8 °C. *R*_f_ = 0.244 (methanol/dichloromethane 3 : 100 v/v). ^1^H NMR (700 MHz, DMSO-*d*_6_): *δ* = 3.74–3.77 (m, 2H, OCH_2_), 4.25 (dd, *J* = 12.9, 9.9 Hz, 1H, NCH_2_), 4.71 (dd, *J* = 12.9, 3.4 Hz, 1H, NCH_2_), 4.89–4.92 (m, 1H, OCH), 5.33 (t, *J* = 5.6 Hz, 1H, OH), 7.28 (t, *J* = 7.5 Hz, 1H, 8-H), 7.47 (t, *J* = 7.2 Hz, 1H, 7-H), 7.50 (d, *J* = 8.1 Hz, 1H, 9-H), 7.63 (d, *J* = 8.4 Hz, 1H, 6-H) ppm. ^13^C NMR (176 MHz, DMSO-*d*_6_): *δ* = 41.5 (NCH_2_), 60.6 (OCH_2_), 66.9 (C-10), 77.9 (OCH), 111.5 (C-6), 121.8 (C-8), 122.5 (C-10a), 122.6 (C-9), 126.5 (C-7), 129.9 (C-9a), 136.3 (C-5a), 158.2 (C=O) ppm. ^15^N NMR (71 MHz, DMSO-*d*_6_): *δ* = −243.8 (N-5) ppm. IR (KBr): 3220 (OH), 2939 (C–H_aliph_), 1721 (C=O), 1516, 1469, 1444, 1407, 1376, 1358, 1315 (C=C, C–N_arom_, CH_2_), 1239, 1201, 1158 (C–O–C), 1046 (C–O of primary alcohol), 965, 744, 737, 667 (C=C) cm^−1^ MS *m*/*z* (%): 344 ([M + H]^+^, 100). HRMS (ESI) for C_12_H_10_INNaO_3_ ([M + H]^+^) calculated 365.9598, found 365.9598.

#### 10-Bromo-3-(hydroxymethyl)-3,4-dihydro-1*H*-[1,4]oxazino[4,3-*a*]indol-1-one (9b)

Compound 9b was obtained as a white solid, yield 225 mg (76%), mp 161.1–161.6 °C. *R*_f_ = 0.297 (methanol/dichloromethane 3 : 100 v/v). ^1^H NMR (400 MHz, DMSO-*d*_6_): *δ* = 3.75–3.77 (m, 2H, OCH_2_), 4.23 (dd, *J* = 12.9, 10.0 Hz, 1H, NCH_2_), 4.69 (dd, *J* = 13.0, 3.3 Hz, 1H, NCH_2_), 4.90–4.95 (m, 1H, OCH), 5.35 (t, *J* = 5.5 Hz, 1H, OH), 7.29 (t, *J* = 7.5 Hz, 1H, 8-H), 7.49 (t, *J* = 7.7 Hz, 1H, 7-H), 7.62 (d, *J* = 8.2 Hz, 1H, 9-H), 7.68 (d, *J* = 8.5 Hz, 1H, 6-H) ppm. ^13^C NMR (101 MHz, DMSO-*d*_6_): *δ* = 41.9 (NCH_2_), 61.1 (OCH_2_), 78.7 (OCH), 97.5 (C-10), 112.1 (C-6), 120.4, 120.9 (C-9), 122.5 (C-8), 126.6, 127.2 (C-7), 135.8 (C-5a), 158.2 (C=O) ppm. IR (KBr): 3225 (OH), 2940 (C–H_aliph_), 1726 (C=O), 1525, 1470, 1446, 1410, 1377, 1360, 1323, 1267 (C–N_arom_, C=C, CH_2_), 1241, 1204, 1163, 1114, 1088 (C–O–C), 1046 (C–O of primary alcohol), 969, 745, 737 (C=C, C-Br) cm^−1^. MS *m*/*z* (%): 296; 298 ([M + H]^+^, 100). HRMS (ESI) for C_12_H_10_BrNNaO_3_ ([M + Na]^+^) calculated 317.9736, found 317.9739.

#### 10-Chloro-3-(hydroxymethyl)-3,4-dihydro-1*H*-[1,4]oxazino[4,3-*a*]indol-1-one (9c)

Compound 9c was obtained as a white solid, yield 218 mg (87%), mp 152.8–153.3 °C. *R*_f_ = 0.083 (methanol/dichloromethane 3 : 100 v/v). ^1^H NMR (700 MHz, DMSO-*d*_6_): *δ* = 3.74–3.79 (m, 2H, OCH_2_), 4.22 (dd, *J* = 12.9, 9.9 Hz, 1H, NCH_2_), 4.68 (dd, *J* = 12.9, 3.4 Hz, 1H, NCH_2_), 4.91–4.94 (m, 1H, OCH), 5.33 (t, *J* = 5.7 Hz, 1H, OH), 7.28–7.30 (m, 1H, 8-H), 7.48–7.50 (m, 1H, 7-H), 7.67–7.70 (m, 2H, 6-H; 9-H) ppm. ^13^C NMR (176 MHz, DMSO-*d*_6_): *δ* = 41.2 (NCH_2_), 60.6 (OCH_2_), 78.2 (OCH), 110.7 (C-10), 111.5 (C-6), 118.3 (C-10a), 119.3 (C-9), 121.8 (C-8), 124.2 (C-9a), 126.7 (C-7), 134.4 (C-5a), 157.4 (C=O) ppm. ^15^N NMR (71 MHz, DMSO-*d*_6_): *δ* = −250.8 (N-5) ppm. IR (KBr): 3234 (OH), 2940 (C–H_aliph_), 1726 (C=O), 1613, 1531, 1449, 1414, 1377, 1364, 1347, 1328, 1267 (C=C, C–N_arom_, CH_2_), 1242, 1211, 1165, 1116, 1087 (C–O–C), 1047 (C–O of primary alcohol), 984, 947 (C=C), 738 (C–Cl) cm^−1^ MS *m*/*z* (%): 252 ([M + H]^+^, 100). HRMS (ESI) for C_12_H_10_ClNNaO_3_ ([M + Na]^+^) calculated 274.0241, found 274.0239.

#### General synthetic procedure for 10a–g

A mixture of the appropriate 3-(hydroxymethyl)-3,4-dihydro-1*H*-[1,4]oxazino[4,3-*a*]indol-1-one 6a–e (0.5 mmol) and Cs_2_CO_3_ (489 mg, 1.5 mmol) in ACN was stirred in ambient temperature for 30 min. The alkyl iodide (2 mmol) was added dropwise, then the temperature was maintained at 60 °C for 24 h. After completion of the reaction (TLC monitoring), the mixture was diluted with water (10 mL) and extracted with ethyl acetate (3 × 10 mL). The combined organic layers were dried over Na_2_SO_4_ and concentrated under vacuum. The obtained residue was purified *via* column chromatography on silica gel (eluent ethyl acetate/hexane v/v) to provide products 10a–g.

#### 3-(Methoxymethyl)-3,4-dihydro-1*H*-[1,4]oxazino[4,3-*a*]indol-1-one (10a)

Compound 10a was obtained as a white solid, yield 86 mg (74%), mp 121.3–121.8 °C. *R*_f_ = 0.514 (ethyl acetate/hexane, 1 : 1 v/v). ^1^H NMR (400 MHz, CDCl_3_): *δ* = 3.46 (s, 3H, CH_3_), 3.72 (dd, *J* = 9.8, 6.9 Hz, 1H, OCH_2_), 3.81 (dd, *J* = 10.2, 4.2 Hz, 1H, OCH_2_), 4.15–4.21 (m, 1H, NCH_2_), 4.44 (dd, *J* = 12.8, 2.8 Hz, 1H, NCH_2_), 4.84–4.92 (m, 1H, OCH), 7.20 (t, *J* = 7.4 Hz, 1H, Ar), 7.33–7.43 (m, 3H, Ar), 7.73 (d, *J* = 8.1 Hz, 1H, Ar) ppm. ^13^C NMR (101 MHz, CDCl_3_): *δ* = 41.8 (NCH_2_), 59.7 (OCH_2_), 71.4 (CH_3_), 76.1 (OCH), 110.0, 110.2, 121.5, 123.2, 123.2, 126.1, 127.0, 136.8, 159.2 (C=O) ppm. IR (KBr): 2916 (C–H_aliph_), 1723 (C=O), 1536, 1467, 1419, 1377, 1350, 1306 (C–N_arom_, C=C, CH_2_, CH_3_), 1241, 1208, 1167, 1138, 1113 (C–O–C), 974, 818, 746 (C=C) cm^−1^ MS *m*/*z* (%): 232 ([M + H]^+^, 100). HRMS (ESI) for C_13_H_13_NNaO_3_ ([M + Na]^+^) calculated 254.0788, found 254.0790.

#### 3-(Methoxymethyl)-8-methyl-3,4-dihydro-1*H*-[1,4]oxazino[4,3-*a*]indol-1-one (10b)

Compound 10b was obtained as a white solid, yield 91 mg (74%), mp 98.5–99.0 °C. *R*_f_ = 0.296 (ethyl acetate/hexane, 1 : 2 v/v). ^1^H NMR (400 MHz, CDCl_3_): *δ* = 2.45 (s, 3H, 8-CH_3_), 3.45 (s, 3H, OCH_3_), 3.71 (dd, *J* = 10.2, 6.5 Hz, 1H, OCH_2_), 3.80 (dd, *J* = 10.2, 4.4 Hz, 1H, OCH_2_), 4.15 (dd, *J* = 12.8, 9.8 Hz, 1H, NCH_2_), 4.40 (dd, *J* = 12.8, 3.5 Hz, 1H, NCH_2_), 4.85–4.91 (m, 1H, OCH), 7.23 (s, 2H, Ar), 7.34 (s, 1H, Ar), 7.50 (s, 1H, Ar) ppm. ^13^C NMR (101 MHz, CDCl_3_): *δ* = 21.4 (C-8, CH_3_) 41.8 (NCH_2_), 59.7 (OCH_2_), 71.4 (OCH_3_), 76.0 (OCH), 109.6, 109.7, 122.4, 123.1, 127.2, 128.1, 130.9, 135.3, 159.3 (C=O) ppm. IR (KBr): 2896, 2815 (C–H_aliph_), 1712 (C=O), 1537, 1469, 1440, 1415, 1385, 1345, 1298 (C–N_arom_, C=C, CH_2,_ CH_3_), 1247, 1210, 1198, 1129, 1081, 1035 (C–O–C), 964, 901, 871, 792, 755, 733 (C=C) cm^−1^ MS *m*/*z* (%): 246 ([M + H]^+^, 100). HRMS (ESI) for C_14_H_15_NNaO_3_ ([M + Na]^+^) calculated 268.0944, found 268.0941.

#### 8-Fluoro-3-(methoxymethyl)-3,4-dihydro-1*H*-[1,4]oxazino[4,3-*a*]indol-1-one (10c)

Compound 10c was obtained as a white solid, yield 98 mg (79%), mp 116.2–116.7 °C. *R*_f_ = 0.442 (ethyl acetate/hexane, 1 : 1 v/v). ^1^H NMR (400 MHz, CDCl_3_): *δ* = 3.46 (s, 3H, CH_3_), 3.72 (dd, *J* = 10.3, 6.4 Hz, 1H, OCH_2_), 3.81 (dd, *J* = 10.3, 4.3 Hz, 1H, OCH_2_), 4.18 (dd, *J* = 12.8, 9.9 Hz, 1H, NCH_2_), 4.42 (dd, *J* = 12.9, 3.5 Hz, 1H, NCH_2_), 4.87–4.93 (m, 1H, OCH), 7.16 (td, *J* = 9.0, 2.3 Hz, 1H, Ar), 7.29 (dd, *J* = 9.2, 4.3 Hz, 1H, Ar), 7.33–7.36 (m, 2H, Ar) ppm. ^13^C NMR (101 MHz, CDCl_3_): *δ* = 41.9 (NCH_2_), 59.7 (OCH_2_), 71.3 (CH_3_), 76.1 (OCH), 107.3 (d, ^2^*J*_C,F_ = 23.5 Hz), 109.7 (d, ^4^*J*_C,F_ = 5.7 Hz), 111.1 (d, ^3^*J*_C,F_ = 9.6 Hz), 115.4 (d, ^2^*J*_C,F_ = 27.4 Hz), 124.5, 127.0 (d, ^3^*J*_C,F_ = 10.4 Hz), 133.5, 158.4 (d, ^1^*J*_C,F_ = 238.3 Hz), 158.9 (C=O) ppm. ^19^F NMR (376 MHz, CDCl_3_): *δ* = −121.5 (F-8) ppm. IR (KBr): 2911 (C–H_aliph_), 1723 (C=O), 1625, 1535, 1468, 1446, 1418, 1307, 1286 (C=C, CH_2_, CH_3_, C–N_arom_), 1239, 1199, 1127, 1102, 1082 (C–O–C, C–F), 972, 958, 942, 866, 808, 791, 743, 690 (C=C) cm^−1^ MS *m*/*z* (%): 250 ([M + H]^+^, 100). HRMS (ESI) for C_13_H_12_FNNaO_3_ ([M + Na]^+^) calculated 272.0693, found 272.0694.

#### 8-Chloro-3-(methoxymethyl)-3,4-dihydro-1*H*-[1,4]oxazino[4,3-*a*]indol-1-one (10d)

Compound 10d was obtained as a white solid, yield 92 mg (69%), mp 148.4–148.9 °C. *R*_f_ = 0.305 (ethyl acetate/hexane, 1 : 1 v/v). ^1^H NMR (400 MHz, CDCl_3_): *δ* = 3.46 (s, 3H, OCH_3_), 3.72 (dd, *J* = 10.2, 6.5 Hz, 1H, OCH_2_), 3.82 (dd, *J* = 10.3, 4.3 Hz, 1H, OCH_2_), 4.20 (dd, *J* = 12.9, 9.8 Hz, 1H NCH_2_), 4.43 (dd, *J* = 12.9, 3.5 Hz, 1H, NCH_2_), 4.88–4.94 (m, 1H, OCH), 7.29 (s, 1H, Ar), 7.34–7.37 (m, 2H, Ar), 7.71 (d, *J* = 1.4 Hz, 1H, Ar) ppm. ^13^C NMR (101 MHz, CDCl_3_): *δ* = 41.9 (NCH_2_), 59.7 (OCH_2_), 71.3 (CH_3_), 76.0 (OCH), 109.4, 111.2, 122.3,124.3, 126.6, 127.2, 127.7, 135.0, 158.8 (C=O) ppm. IR (KBr): 2909, 2875 (C–H_aliph_), 1723 (C=O), 1534, 1470, 1420, 1383, 1354, 1311, 1276 (C=C, CH_2_, CH_3_, C–N_arom_), 1246, 1189, 1159, 1130, 1086, 1059, 1035 (C–O–C), 962, 913, 899, 856, 811, 754, 732, 708, 666, 610 (C=C, C–Cl) cm^−1^. MS *m*/*z* (%): 266 ([M + H]^+^, 100). HRMS (ESI) for C_13_H_12_ClNNaO_3_ ([M + Na]^+^) calculated 288.0398, found 288.0399.0.

#### 8-Bromo-3-(methoxymethyl)-3,4-dihydro-1*H*-[1,4]oxazino[4,3-*a*]indol-1-one (10e)

Compound 10e was obtained as a white solid, yield 120 mg (77%), mp 151.6–152.1 °C. *R*_f_ = 0.262 (ethyl acetate/hexane, 1 : 1 v/v). ^1^H NMR (400 MHz, CDCl_3_): *δ* = 3.45 (s, 3H, CH_3_), 3.71 (dd, *J* = 10.2, 6.4 Hz, 1H, OCH_2_), 3.81 (dd, *J* = 10.3, 4.3 Hz, 1H, OCH_2_), 4.17 (dd, *J* = 12.8, 9.9 Hz, 1H, NCH_2_), 4.41 (dd, *J* = 12.9, 3.4 Hz, 1H, NCH_2_), 4.87–4.92 (m, 1H, OCH), 7.22 (d, *J* = 8.8 Hz, 1H, Ar), 7.31 (s, 1H, Ar), 7.45 (d, *J* = 8.9 Hz, 1H, Ar), 7.85 (s, 1H, Ar) ppm. ^13^C NMR (101 MHz, CDCl_3_): *δ* = 41.9 (NCH_2_), 59.7 (OCH_2_), 71.3 (CH_3_), 76.0 (OCH), 109.2, 111.5, 114.6, 124.1, 125.5, 128.3, 129.0, 135.2, 158.7 (C=O) ppm. IR (KBr): 2874 (C–H_arom_), 1721 (C=O), 1532, 1469, 1383, 1343, 1310, 1275 (C=C, C–N_arom_, CH_2_, CH_3_), 1246, 1188, 1160, 1080, 1036 (C–O–C), 962, 858, 811, 754, 732, 695, 664 (C=C) cm^−1^. MS *m*/*z* (%): 310; 312 ([M + H]^+^, 100). HRMS (ESI) for C_13_H_12_BrNNaO_3_ ([M + Na]^+^) calculated 331.9893, found 331.9894.

#### 3-(Ethoxymethyl)-3,4-dihydro-1*H*-[1,4]oxazino[4,3-*a*]indol-1-one (10f)

Compound 10f was obtained as a white solid, yield 76 mg (62%), mp 113.6–114.1 °C. *R*_f_ = 0.2 (ethyl acetate/hexane, 1 : 3 v/v). ^1^H NMR (700 MHz, CDCl_3_): *δ* = 1.15 (t, *J* = 7.0 Hz, 3H, *OEt* CH_3_), 3.53 (q, *J* = 7.0 Hz, 2H, *OEt* CH_2_), 3.67 (dd, *J* = 10.3, 6.6 Hz, 1H, OCH_2_), 3.77 (dd, *J* = 10.3, 4.3 Hz, 1H, OCH_2_), 4.11 (dd, *J* = 12.7, 9.7 Hz, 1H, NCH_2_), 4.37 (dd, *J* = 12.7, 3.5 Hz, 1H, NCH_2_), 4.79–4.83 (m, 1H, OCH), 7.12 (t, *J* = 7.5 Hz, 1H), 7.27 (d, *J* = 8.4 Hz, 1H), 7.32 (t, *J* = 7.6 Hz, 1H), 7.34 (s, 1H), 7.65 (d, *J* = 8.1 Hz, 1H) ppm. ^13^C NMR (176 MHz, CDCl_3_): *δ* = 15.1 (*OEt* CH_3_), 41.9, 67.5, 69.4, 76.3 (OCH), 110.1, 110.2, 121.5, 123.2, 123.3, 126.1, 127.0, 136.8, 159.3 (C=O) ppm. IR (KBr): 2972, 2869 (C–H_aliph_), 1710 (C=O), 1540, 1473, 1416, 1377, 1349 (C=C, C–N_arom_, CH_2_, CH_3_), 1244, 1205, 1173, 1138, 1090 (C–O–C), 965, 743 (C=C) cm^−1^. MS *m*/*z* (%): 246 ([M + H]^+^, 100). HRMS (ESI) for C_14_H_16_NO_3_ ([M + H]^+^) calculated 246.1125, found 246.1120.

#### 3-(Butoxymethyl)-3,4-dihydro-1*H*-[1,4]oxazino[4,3-*a*]indol-1-one (10g)

Compound 10g was obtained as a white solid, yield 67 mg (49%), mp 62.8–63.3 °C. *R*_f_ = 0.256 (ethyl acetate/hexane, 1 : 6 v/v). ^1^H NMR (700 MHz, CDCl_3_): 0.85 (t, *J* = 7.4 Hz, 3H, *Bu* CH_3_), 1.27–1.32 (m, 2H, *Bu***CH**_2_CH_3_), 1.48–1.52 (m, 2H, *Bu* OCH_2_-**CH**_2_), 3.46 (t, *J* = 6.6 Hz, 2H, *Bu* OCH_2_), 3.66 (dd, *J* = 10.3, 6.7 Hz, 1H, OCH_2_), 3.77 (dd, *J* = 10.3, 4.3 Hz, 1H, OCH_2_), 4.12 (dd, *J* = 12.7, 9.6 Hz, 1H, NCH_2_), 4.37 (dd, *J* = 12.7, 3.5 Hz, 1H, NCH_2_), 4.80–4.83 (m, 1H, OCH), 7.12 (t, *J* = 7.5 Hz, 1H, Ar), 7.28 (d, *J* = 8.4 Hz, 1H, Ar), 7.31–7.33 (m, 1H, Ar) 7.35 (s, 1H, Ar), 7.65 (d, *J* = 8.1 Hz, 1H, Ar) ppm.^13^C NMR (176 MHz, CDCl_3_): *δ* = 13.9 (*Bu* CH_3_), 19.2, 31.6, 41.9, 69.6, 71.9, 76.3 (OCH), 110.1, 110.2, 121.5, 123.2, 123.3, 126.1, 127.0, 136.8, 159.3 (C=O) ppm. IR (KBr): 3050 (C–H_arom_), 2933, 2833 (C–H_aliph_), 1711 (C=O), 1535, 1472, 1421, 1387, 1351, 1306 (C=C, C–N_arom_, CH_2_, CH_3_), 1255, 1203, 1107, 1080 (C–O–C), 997, 961, 809, 759, 736 (C=C) cm^−1^. MS *m*/*z* (%): 274 ([M + H]^+^, 100). HRMS (ESI) for C_16_H_20_NO_3_ ([M + H]^+^) calculated 274.1438, found 274.1433.

#### General synthetic procedure for 12a–d

10-Iodo-3-(methoxymethyl)-3,4-dihydro-1*H*-[1,4]oxazino[4,3-*a*]indol-1-one 11 (89 mg, 0.25 mmol) was placed into a microwave tube with Cs_2_CO_3_ (224 mg, 0.75 mmol), a boronic acid (0.75 mmol), and Pd(PPh_3_)_4_ (23 mg, 0.02 mmol) in anhydrous 1,4-dioxane, and the reaction was stirred at 100 °C under microwave conditions (300 W) for 30 min. After completion of the reaction (TLC monitoring), the mixture was diluted with water (10 mL) and extracted with ethyl acetate (3 × 10 mL). The combined organic layers were dried over Na_2_SO_4_ and concentrated under reduced pressure. The obtained residue was purified *via* column chromatography on silica gel (eluent ethyl acetate/hexane, 1 : 2 v/v) to provide the desired products 12a–d.

#### 3-(Methoxymethyl)-10-phenyl-3,4-dihydro-1*H*-[1,4]oxazino[4,3-*a*]indol-1-one (12a)

Compound 12a was obtained as a white solid, yield 59 mg (77%), mp 145.7–146.2 °C. *R*_f_ = 0.282 (ethyl acetate/hexane 1 : 1 v/v). ^1^H NMR (700 MHz, CDCl_3_): *δ* = 3.46 (s, 3H, CH_3_), 3.73 (dd, *J* = 10.2, 6.7 Hz, 1H, OCH_2_), 3.82 (dd, *J* = 10.2, 4.4 Hz, 1H, OCH_2_), 4.21 (dd, *J* = 12.7, 9.8 Hz, 1H, NCH_2_), 4.50 (dd, *J* = 12.7, 3.4 Hz, 1H, NCH_2_), 4.89–4.92 (m, 1H, OCH), 7.20 (t, *J* = 7.5 Hz, 1H, 8-H), 7.37–7.40 (m, 2H, *Ph* 4-H; 6-H), 7.43–7.45 (m, 1H, 7-H), 7.47 (t, *J* = 7.7 Hz, 2H, *Ph* 3,5-H), 7.63 (d, *J* = 7.6 Hz, 2H, *Ph* 2,6-H), 7.75 (d, *J* = 8.2 Hz, 1H, 9-H) ppm. ^13^C NMR (176 MHz, CDCl_3_): *δ* = 42.1 (NCH_2_), 59.7 (CH_3_), 71.4 (OCH_2_), 75.5 (OCH), 109.9 (C-6), 118.2 (C-10a), 121.6 (C-8), 122.3 (C-9), 126.6 (C-7), 126.7 (C-10), 126.8 (C-9a), 127.7 (*Ph* C-4), 128.1 (*Ph* C-3,5), 130.4 (*Ph* C-2,6), 132.4 (*Ph* C-1), 136.0 (C-5a), 158.6 (C=O) ppm. ^15^N NMR (71 MHz, CDCl_3_): *δ* = −254.1 (N-5) ppm. IR (KBr): 2901 (C–H_aliph_), 1727 (C=O), 1544, 1494, 1467, 1414, 1380, 1335, 1304 (C=C, C–N_arom_, CH_2_, CH_3_), 1232, 1200, 1157, 1114, 1098 (C–O–C), 975, 780, 752, 699 (C=C) cm^−1^. MS *m*/*z* (%): 308 ([M + H]^+^, 100). HRMS (ESI) for C_19_H_17_NNaO_3_ ([M + Na]^+^) calculated 330.1101, found 330.1100.

#### 3-(Methoxymethyl)-10-(*p*-tolyl)-3,4-dihydro-1*H*-[1,4]oxazino[4,3-*a*]indol-1-one (12b)

Compound 12b was obtained as a brown solid, yield 66 mg (82%), mp 166.3–166.8 °C. *R*_f_ = 0.217 (ethyl acetate/hexane 1 : 2 v/v). ^1^H NMR (700 MHz, CDCl_3_): *δ* = 2.34 (s, 3H, *Ph* CH_3_), 3.38 (s, 3H, CH_3_), 3.64 (dd, *J* = 10.2, 6.7 Hz, 1H, OCH_2_), 3.73 (dd, *J* = 10.2, 4.4 Hz, 1H, OCH_2_), 4.12 (dd, *J* = 12.7, 9.8 Hz, 1H, NCH_2_), 4.41 (dd, *J* = 12.7, 3.3 Hz, 1H, NCH_2_), 4.80–4.84 (m, 1H, OCH), 7.11 (t, *J* = 7.5 Hz, 1H, 8-H), 7.20 (d, *J* = 7.8 Hz, 2H, *Ph* 3,5-H), 7.29 (d, *J* = 8.4 Hz, 1H, 6-H), 7.34–7.36 (m, 1H, 7-H), 7.45 (d, *J* = 7.9 Hz, 2H, *Ph* 2,6-H), 7.67 (d, *J* = 8.2 Hz, 1H, 9-H) ppm. ^13^C NMR (176 MHz, CDCl_3_): *δ* = 21.4 (*Ph* CH_3_), 42.1 (NCH_2_), 59.7 (OCH_3_), 71.5 (OCH_2_), 75.5 (OCH), 109.9 (C-6), 118.1 (C-10a), 121.5 (C-8), 122.4 (C-9), 126.6 (C-7), 126.9 (C-9a; C-10), 129.0 (*Ph* C-3,5), 129.4 (*Ph* C-1), 130.3 (*Ph* C-2,6), 136.0 (C-5a), 137.5 (*Ph* C-4), 158.7 (C=O) ppm. IR (KBr): 2987 (C–H_aliph_), 1709 (C=O), 1550, 1503, 1470, 1418, 1385, 1331 (C=C, C–N_arom_, CH_2_, CH_3_), 1242, 1162, 1126, 1087 (C–O–C), 966, 814, 739 (C=C) cm^−1^. MS *m*/*z* (%): 322 ([M + H]^+^, 100). HRMS (ESI) for C_20_H_19_NNaO_3_ ([M + Na]^+^) calculated 344.1257, found 344.1262.

#### 3-(Methoxymethyl)-10-(4-methoxyphenyl)-3,4-dihydro-1*H*-[1,4]oxazino[4,3-*a*]indol-1-one (12c)

Compound 12c was obtained as a brown solid, yield 64 mg (76%), mp 158.3–158.8 °C. *R*_f_ = 0.181 (ethyl acetate/hexane 1 : 2 v/v). ^1^H NMR (700 MHz, CDCl_3_): *δ* = 3.38 (s, 1H, OCH_3_), 3.64 (dd, *J* = 10.2, 6.7 Hz, 1H, OCH_2_), 3.73 (dd, *J* = 10.2, 4.4 Hz, 1H, OCH_2_), 3.78 (s, 1H, *Ph* OCH_3_), 4.11 (dd, *J* = 12.7, 9.8 Hz, 1H, NCH_2_), 4.40 (dd, *J* = 12.7, 3.4 Hz, 1H, NCH_2_), 4.79–4.83 (m, 1H, OCH), 6.93 (d, *J* = 8.7 Hz, 2H, *Ph* 3,5-H), 7.11 (t, *J* = 7.5 Hz, 1H, 8-H), 7.28 (d, *J* = 8.4 Hz, 1H, 6-H), 7.34–7.36 (m, 1H, 7-H), 7.50 (d, *J* = 8.7 Hz, 2H, *Ph* 2,6-H), 7.67 (d, *J* = 8.2 Hz, 1H, 9-H) ppm. ^13^C NMR (176 MHz, CDCl_3_): *δ* = 42.1 (NCH_2_), 55.3 (*Ph* OCH_3_), 59.7 (OCH_3_), 71.5 (OCH_2_), 75.5 (OCH), 109.9 (C-6), 113.7 (*Ph* C-3,5), 117.9 (C-10a), 121.5 (C-8), 122.4 (C-9), 124.7 (*Ph* C-1), 126.6 (C-7), 126.7 (C-10), 126.9 (C-9a), 131.6 (*Ph* C-2,6), 136.0 (C-5a), 158.8 (C=O), 159.2 (*Ph* C-4) ppm. ^15^N NMR (71 MHz, CDCl_3_): *δ* = −254.64 (N-5) ppm. IR (KBr): 2932, 2834 (C–H_aliph_), 1712 (C=O), 1609, 1552, 1503, 1471, 1419, 1381, 1336, 1308, 1283 (C=C, C–N_arom_, CH_2_, CH_3_), 1248, 1164, 1115, 1094 (C–O–C), 1035, 967, 838, 744 (C=C) cm^−1^. MS *m*/*z* (%): 338 ([M + H]^+^, 100). HRMS (ESI) for C_20_H_19_NNaO_4_ ([M + Na]^+^) calculated 360.1206, found 360.1209.

#### 3-(Methoxymethyl)-10-(thiophen-3-yl)-3,4-dihydro-1*H*-[1,4]oxazino[4,3-*a*]indol-1-one (12d)

Compound 12d was obtained as a brown solid, yield 58 mg (74%), mp 122.5–123.0 °C. *R*_f_ = 0.234 (ethyl acetate/hexane 1 : 2 v/v). ^1^H NMR (700 MHz, CDCl_3_): *δ* = 3.37 (s, 3H, CH_3_), 3.63 (dd, *J* = 10.2, 6.6 Hz, 1H, OCH_2_), 3.72 (dd, *J* = 10.2, 4.3 Hz, 1H, OCH_2_), 4.11 (dd, *J* = 12.7, 9.8 Hz, 1H, NCH_2_), 4.39 (dd, *J* = 12.7, 3.4 Hz, 1H, NCH_2_), 4.77–4.80 (m, 1H, OCH), 7.13–7.15 (m, 1H, 8-H), 7.27 (d, *J* = 8.4 Hz, 1H, 6-H), 7.32 (dd, *J* = 4.9, 3.0 Hz, 1H, *Th* 5-H), 7.34–7.36 (m, 1H, 7-H), 7.43 (d, *J* = 4.9 Hz, 1H, *Th* 4-H), 7.61 (d, *J* = 2.9 Hz, 1H, *Th* 2-H), 7.78 (d, *J* = 8.2 Hz, 1H, 9-H) ppm. ^13^C NMR (176 MHz, CDCl_3_): *δ* = 42.1 (NCH_2_), 59.7 (CH_3_), 71.4 (OCH_2_), 75.4 (OCH), 109.9 (C-6), 118.2 (C-10a), 121.3 (C-10), 121.7 (C-8), 122.5 (C-9), 124.6 (*Th* C-5), 125.0 (*Th* C-2), 126.7 (C-7; C-9a), 129.7 (*Th* C-4), 132.3 (*Th* C-3), 136.0 (C-5a), 158.7 (C=O) ppm. ^15^N NMR (71 MHz, CDCl_3_): *δ* = −254.3 (N-5) ppm. IR (KBr): 3107 (C–H_arom_), 2898 (C–H_aliph_), 1717 (C=O), 1557, 1514, 1497, 1468, 1427, 1381, 1303 (C=C, C–N_arom_, CH_2_, CH_3_), 1240, 1204, 1162, 1110, 1097 (C–O–C), 971, 842, 795, 750 (C=C) cm^−1^. MS *m*/*z* (%): 314 ([M + H]^+^, 100). HRMS (ESI) for C_17_H_15_NNaO_3_S ([M + Na]^+^) calculated 336.0665, found 336.0663.

#### Synthetic procedure for 13

10-Iodo-3-(methoxymethyl)-3,4-dihydro-1*H*-[1,4]oxazino[4,3-*a*]indol-1-one 11 (89 mg, 0.25 mmol) was dissolved in dry DMF under argon atmosphere, TEA (0.17 mL, 1.25 mmol), CuI (9 mg 0.05 mmol) and Pd(PPh_3_)_2_Cl_2_ were added, reaction stirred at 70 °C for 30 min. After completion of the reaction (TLC monitoring), the mixture was diluted with water (20 mL) and extracted with ethyl acetate (3 × 20 mL). The combined organic layers were dried over Na_2_SO_4_ and concentrated under vacuum. The obtained residue was purified *via* column chromatography on silica gel (eluent ethyl acetate/hexane, 1 : 2 v/v) to provide product 13.

#### 3-(Methoxymethyl)-10-(phenylethynyl)-3,4-dihydro-1*H*-[1,4]oxazino[4,3-*a*]indol-1-one (13)

Compound 13 was obtained as a yellow solid, yield 66 mg (80%), mp 149.9–150.4 °C. *R*_f_ = 0.262 (acetone/hexane 1 : 3 v/v). ^1^H NMR (700 MHz, CDCl_3_): *δ* = 3.46 (s, 3H, CH_3_), 3.73 (dd, *J* = 10.2, 6.8 Hz, 1H, OCH_2_), 3.83 (dd, *J* = 10.2, 4.3 Hz, 1H, OCH_2_), 4.23 (dd, *J* = 12.8, 9.5 Hz, 1H, NCH_2_), 4.48 (dd, *J* = 12.8, 3.4 Hz, 1H, NCH_2_), 4.87–4.91 (m, 1H, OCH), 7.30 (t, *J* = 7.3 Hz, 1H, 8-H), 7.34–7.38 (m, 4H, 6-H; *Ph* 3,4,5-H) 7.47 (t, *J* = 8.1 Hz, 1H, 7-H), 7.66–7.67 (m, 2H, *Ph* 2,6-H) 7.94 (d, *J* = 8.1 Hz, 1H, 9-H). ^13^C NMR (176 MHz, CDCl_3_): *δ* = 42.1 (NCH_2_), 59.7 (CH_3_), 71.3 (OCH_2_), 75.5 (OCH_3_), 81.3 (**C**≡C–Ph), 97.5 (C≡**C**–Ph), 106.4 (C-10), 110.1 (C-6), 122.1 (C-8), 122.3 (C-9), 123.5 (*Ph* C-1), 123.6 (C-10a), 127.0 (C-7), 128.3 (*Ph* C-3,6), 128.4 (*Ph* C-4), 128.6 (C-9a), 131.9 (*Ph* C-2,6), 135.7 (C-5a), 157.5 (C=O) ppm. ^15^N NMR (71 MHz, CDCl_3_): *δ* = −252.3 (N-5) ppm. IR (KBr): 3051 (C–H_arom_), 2919, 2810 (C–H_aliph_), 1715 (C=O), 1546, 1469, 1422, 1377, 1332, 1305 (C=C, C–N_arom_, CH_2_, CH_3_), 1240, 1196, 1160, 1116, 1066 (C–O–C), 1041, 965, 752, 741 (C=C) cm^−1^. MS *m*/*z* (%): 332 ([M + H]^+^, 100). HRMS (ESI) for C_21_H_17_NNaO_3_ ([M + Na]^+^) calculated 354.1101, found 354.1097.

#### Synthetic procedure for 14

POCl_3_ (0.28 mL, 3 mmol) was slowly added dropwise into ice cold DMF (0.27 mL, 3.5 mmol), and stirred for 15 min. 3-(Methoxymethyl)-3,4-dihydro-1*H*-[1,4]oxazino[4,3-*a*]indol-1-one (231 mg, 1 mmol) 8a was dissolved in 3 mL DMF, then added to the formylating agent and stirred for 24 h at room temperature. After completion of the reaction (TLC monitoring), the mixture was treated with 1 M Na_2_CO_3_ and extracted with dichloromethane. The combined organic layers were dried over Na_2_SO_4_ and concentrated under vacuum. The obtained residue was purified *via* column chromatography on silica gel (eluent ethyl acetate/hexane, 1 : 2 v/v) to provide product 14.

#### 3-(Methoxymethyl)-1-oxo-3,4-dihydro-1*H*-[1,4]oxazino[4,3-*a*]indole-10-carbaldehyde (14)

Compound 14 was obtained as a white solid, yield 155 mg (60%), mp 178.4–178.9 °C. *R*_f_ = 0.139 (ethyl acetate/hexane 1 : 2 v/v). ^1^H NMR (700 MHz, CDCl_3_): *δ* = 3.48 (s, 3H, CH_3_), 3.78 (dd, *J* = 10.4, 6.1 Hz, 1H, OCH_2_), 3.86 (dd, *J* = 10.4, 4.1 Hz, 1H, OCH_2_), 4.29 (dd, *J* = 13.2, 10.0 Hz, 1H, NCH_2_), 4.51 (dd, *J* = 13.2, 3.5 Hz, 1H, NCH_2_), 4.95–4.99 (m, 1H, OCH), 7.37–7.41 (m, 2H, 8-H; 6-H), 7.49 (t, *J* = 7.7 Hz, 1H, 7-H), 8.46 (d, *J* = 8.1 Hz, 1H, 9-H), 10.75 (s, 1H, H-C=O) ppm. ^13^C NMR (176 MHz, CDCl_3_): *δ* = 41.8 (NCH_2_), 59.8 (CH_3_), 71.1 (OCH_2_), 76.0 (OCH), 110.1 (C-6), 121.0 (C-10), 124.2 (C-9), 124.9 (C-8; C-9a), 126.7 (C-10a), 127.4 (C-7), 135.5 (C-5a), 157.6 (C=O), 187.9 (H–C=O) ppm. IR (KBr): 3000 (C–H_arom_), 2858, 2810 (C–H_aliph_), 1731, 1652 (C=O), 1534, 1476, 1450, 1423, 1390, 1307 (C=C, C–N_arom_, CH_2_, CH_3_), 1227, 1163, 1119, 1070 (C–O–C), 1037, 965, 836, 748 (C=C) cm^−1^. MS *m*/*z* (%): 260 ([M + H]^+^, 100). HRMS (ESI) for C_14_H_13_NNaO_4_ ([M + Na]^+^) calculated 282.0737, found 282.0734.

#### General synthetic procedure for 15a–c

3-(Methoxymethyl)-1-oxo-3,4-dihydro-1*H*-[1,4]oxazino[4,3-*a*]indole-10-carbaldehyde (65 mg, 0.25 mmol) 14 was dissolved in DMF, the appropriate *o*-phenylenediamine (0.25 mmol) was added and the mixture was stirred at 100 °C for 4–24 h. After completion of the reaction (TLC monitoring), solvent was evaporated under reduced pressure and concentrated under vacuum for 1 h. Products 15a–c were obtained without further purification.

#### 10-(1H-Benzo[*d*]imidazole-2-yl)-3-(methoxymethyl)-3,4-dihydro-1*H*-[1,4]oxazino[4,3-*a*]indol-1-one (15a)

Compound 15a was obtained as a yellow solid, yield 81 mg (93%), mp 220.5–221.0 °C. *R*_f_ = 0.283 (ethyl acetate/hexane 1 : 1 v/v). ^1^H NMR (700 MHz, CDCl_3_): *δ* = 3.47 (s, 3H, CH_3_), 3.74 (dd, *J* = 10.4, 5.9 Hz, 1H, OCH_2_), 3.81 (dd, *J* = 10.4, 4.2 Hz, 1H, OCH_2_), 4.13 (dd, *J* = 12.7, 10.2 Hz, 1H, NCH_2_), 4.34 (dd, *J* = 12.8, 3.5 Hz, 1H, NCH_2_), 4.98–5.01 (m, 1H, OCH), 7.13 (d, *J* = 8.3 Hz, 1H, 6-H), 7.28–7.30 (m, 2H, *BIM* 5-H; 6-H), 7.36 (t, *J* = 7.5 Hz, 1H, 8-H), 7.42 (t, *J* = 7.6 Hz, 1H, 7-H), 7.54 (br s, 1H, *BIM* 7-H), 7.86 (br s, 1H, *BIM* 4-H) 9.20 (d, *J* = 8.2 Hz, 1H, 9-H), 12.61 (br s, 1H, NH) ppm. ^13^C NMR (176 MHz, CDCl_3_): *δ* = 41.5 (NCH_2_), 59.7 (CH_3_), 71.1 (OCH_2_), 76.1 (OCH), 109.4 (C-6), 111.4 (*BIM* C-7), 115.1 (C-10), 118.7 (C-10a), 119.3 (*BIM* C-4), 122.2 (*BIM* C-5), 123.2 (C-8; *BIM* C-6), 126.0 (C-9a), 126.2 (C-9), 127.6 (C-7), 133.0 (*BIM* C-7a), 136.2 (C-5a), 144.3 (*BIM* C-3a), 146.8 (*BIM* C-2), 161.2 (C=O) ppm. ^15^N NMR (71 MHz, CDCl_3_): *δ* = −251.5 (N-5), −236.3 (*BIM* N-1), −137.3 (*BIM* N-3) ppm. IR (KBr): 3214 (N-H), 2915, 2872, 2807 (C–H_aliph_), 1696 (C=O), 1614, 1562, 1497, 1466, 1451, 1398, 1375, 1348, 1326 1273 (C=C, C–N_arom_, CH_2_, CH_3_), 1242, 1176, 1120, 1090, 1072 (C–O–C), 973, 955, 940, 772, 739 (C=C) cm^−1^. MS *m*/*z* (%): 348 ([M + H]^+^, 100). HRMS (ESI) for C_20_H_18_N_3_O_3_ ([M + H]^+^) calculated 348.1343, found 348.1345.

#### 10-(5,6-Dimethyl-1*H*-benzo[*d*]imidazole-2-yl)-3-(methoxymethyl)-3,4-dihydro-1*H*-[1,4]oxazino[4,3-*a*]indol-1-one (15b)

Compound 15b was obtained as a yellow solid, yield 88 mg (94%), mp 228.6–229.1 °C. *R*_f_ = 0.248 (ethyl acetate/hexane 1 : 1 v/v). ^1^H NMR (700 MHz, CDCl_3_): *δ* = 2.41 (s, 6H *BIM* CH_3_), 3.49 (s, 3H, OCH_3_), 3.79 (dd, *J* = 10.2, 6.2 Hz, 1H, OCH_2_), 3.86 (dd, *J* = 10.3, 4.1 Hz, 1H, OCH_2_), 4.26–4.29 (m, 1H, NCH_2_), 4.49–4.53 (m, 1H, NCH_2_), 4.97–5.00 (m, 1H, OCH), 7.32 (dd, *J* = 8.1, 4.1 Hz, 1H, 6-H), 7.35 (br s, 1H, *BIM* 7-H), 7.40 (t, *J* = 7.6 Hz, 1H, 8-H), 7.51 (t, *J* = 7.6 Hz, 1H, 7-H), 7.65 (br s, 1H, *BIM* 4-H), 9.29 (d, *J* = 7.8 Hz, 1H, 9-H), 12.55 (br s, 1H, NH) ppm. ^13^C NMR (176 MHz, CDCl_3_): *δ* = 20.4 (*BIM* CH_3_), 20.6 (*BIM* CH_3_), 41.6 (NCH_2_), 59.7 (OCH_3_), 71.2 (OCH_2_), 76.0 (OCH), 109.3 (C-6), 111.5 (*BIM* C-7), 115.8 (C-10), 118.4 (C-10a), 119.4 (*BIM* C-4), 123.0 (C-8), 126.1 (C-9a), 126.5 (C-9), 127.7 (C-7), 131.0 (*BIM***C**–CH_3_), 131.7 (*BIM* C-7a), 132.5 (*BIM***C**–CH_3_), 136.4 (C-5a), 143.1 (*BIM* C-3a), 146.0 (*BIM* C-2), 161.3 (C=O) ppm. ^15^N NMR (71 MHz, CDCl_3_): *δ* = −251.4 (N-5), −237.6 (*BIM* N-1), −137.4 (*BIM* N-3) ppm. IR (KBr): 3235 (N–H), 2922, 2900 (C–H_aliph_), 1696 (C=O), 1628, 1563, 1495, 1466, 1398, 1378, 1343, 1304 (C=C, C–N_arom_, CH_2_, CH_3_), 1242, 1229, 1156, 1116 (C–O–C), 994, 956, 976, 867, 839, 749 (C=C) cm^−1^. MS *m*/*z* (%): 376 ([M + H]^+^, 100). HRMS (ESI) for C_22_H_22_N_3_O_3_ ([M + H]^+^) calculated 376.1656, found 376.1653.

#### 10-(5,6-Dichloro-1*H*-benzo[*d*]imidazole-2-yl)-3-(methoxymethyl)-3,4-dihydro-1*H*-[1,4]oxazino[4,3-*a*]indol-1-one (15c)

Compound 15c was obtained as a yellow solid, yield 99 mg (95%), mp 225.1–225.6 °C. *R*_f_ = 0.190 (ethyl acetate/hexane 1 : 1 v/v). ^1^H NMR (700 MHz, CDCl_3_): *δ* = 3.50 (s, 3H, CH_3_), 3.82 (dd, *J* = 10.4, 6.1 Hz, 1H, OCH_2_), 3.89 (dd, *J* = 10.4, 4.2 Hz, 1H, OCH_2_), 4.35 (dd, *J* = 13.0, 9.9 Hz, 1H, NCH_2_), 4.59 (dd, *J* = 13.0, 3.5 Hz, 1H, NCH_2_), 4.99–5.03 (m, 1H, OCH), 7.41 (d, *J* = 8.4 Hz, 1H, 6-H), 7.44–7.46 (m, 1H, 8-H), 7.55–7.58 (m, 1H, 7-H), 7.68 (s, 1H, *BIM* 7-H), 7.96 (s, 1H, *BIM* 4-H), 9.23 (d, *J* = 8.3 Hz, 1H, 9-H), 12.87 (br s, 1H, NH) ppm. ^13^C NMR (176 MHz, CDCl_3_): *δ* = 41.8 (NCH_2_), 59.8 (CH_3_), 71.0 (OCH_2_), 76.3 (OCH), 109.6 (C-6), 112.5 (*BIM* C-7), 114.7 (C-10), 119.1 (C-10a), 120.5 (*BIM* C-4), 123.7 (C-8), 126.2 (C-9a; *BIM* C–Cl), 126.3 (C-9), 126.9 (*BIM* C–Cl), 128.1 (C-7), 132.3 (*BIM* C-7a), 136.5 (C-5a), 143.9 (BIM C-3a), 148.8 (*BIM* C-2), 161.4 (C=O) ppm. ^15^N NMR (71 MHz, CDCl_3_): *δ* = −249.3 (N-5), −237.6 (*BIM* N-1) ppm. IR (KBr): 3156 (N–H), 2979, 2930, 2866 (C–H_aliph_), 1693 (C=O), 1620, 1560, 1496, 1468, 1447, 1422, 1397, 1380, 1333, 1304 (C=C, C–N_arom_, CH_2_, CH_3_), 1250, 1201, 1173, 1159, 1119, 1093 (C–O–C), 976, 951, 860, 834, 813, 750, 698 (C=C, C–Cl) cm^−1^. MS *m*/*z* (%): 416; 418 ([M + H]^+^, 100). HRMS (ESI) for C_20_H_16_Cl_2_N_3_O_3_ ([M + H]^+^) calculated 416.0563, found 416.0573.

#### General synthetic procedure for 16a–c

In a mixture of 3-(hydroxymethyl)-3,4-dihydro-1*H*-[1,4]oxazino[4,3-*a*]indol-1-one 6a (109 mg, 0.5 mmol) and anhydrous toluene, triphenylphosphine (164 mg, 0.625 mmol) and the appropriate heteroaryl thiol (0.5 mmol) were added. Diisopropyl azodicarboxylate (0.12 mL, 0.625 mmol) was then added dropwise in the dark and the temperature was maintained at 80 °C for 2 h. After completion of the reaction (TLC monitoring), the solvent was evaporated under reduced pressure. The mixture was diluted with water (10 mL) and extracted with ethyl acetate (3 × 10 mL). The combined organic layers were dried over Na_2_SO_4_ and concentrated under vacuum. The obtained residue was purified *via* column chromatography on silica gel (eluent ethyl acetate/hexane, 1 : 4 v/v) to provide the desired products 16a–c.

#### 3-[(Benzo[*d*]thiazol-2-ylthio)methyl]-3,4-dihydro-1*H*-[1,4]oxazino[4,3-*a*]indol-1-one (16a)

Compound 16a was obtained as a white solid, yield 156 mg (85%), mp 127.3–127.8 °C. *R*_f_ = 0.305 (ethyl acetate/hexane, 1 : 4 v/v). ^1^H NMR (400 MHz, CDCl_3_): *δ* = 3.76 (dd, *J* = 14.4, 7.1 Hz, 1H, SCH_2_), 3.96 (dd, *J* = 14.3, 5.3 Hz, 1H, SCH_2_), 4.23 (dd, *J* = 12.7, 9.2 Hz, 1H, NCH_2_), 4.60 (dd, *J* = 12.9, 3.1 Hz, 1H, NCH_2_), 5.21–5.27 (m, 1H, OCH), 7.18 (t, *J* = 7.5 Hz, 1H, Ar), 7.26–7.36 (m, 3H, Ar), 7.41 (t, *J* = 7.7 Hz, 1H, Ar), 7.44 (s, 1H, 10-H), 7.73 (t, *J* = 9.1 Hz, 2H, Ar), 7.79 (d, *J* = 8.1 Hz, 1H, Ar) ppm. ^13^C NMR (101 MHz, CDCl_3_): *δ* = 33.9 (SCH_2_), 42.8 (NCH_2_), 76.3 (OCH), 110.0, 110.5, 121.1, 121.5, 121.6, 122.9, 123.2, 124.7, 126.1, 126.2, 127.0, 135.4, 136.8, 152.6, 158.9, 164.6 ppm. IR (KBr): 1727 (C=O), 1536, 1460, 1427, 1405, 1381, 1349, 1320, 1313 (C=C, C–N_arom_, CH_2_), 1246, 1234, 1197, 1079 (C–O–C), 993, 953, 752, 737, 723 (C=C) cm^−1^. MS *m*/*z* (%): 367 ([M + H]^+^, 100). HRMS (ESI) for C_19_H_15_N_2_O_2_S_2_ ([M + H]^+^) calculated 367.0569, found 367.0564.

#### 3-[(Benzo[*d*]oxazol-2-ylthio)methyl]-3,4-dihydro-1*H*-[1,4]oxazino[4,3-*a*]indol-1-one (16b)

Compound 16b was obtained as a light brown solid, yield 144 mg (82%), mp 158.7–159.2 °C. *R*_f_ = 0.141 (ethyl acetate/hexane, 1 : 4 v/v). ^1^H NMR (700 MHz, CDCl_3_): *δ* = 3.65 (dd, *J* = 14.5, 6.6 Hz, 1H, SCH_2_), 3.77 (dd, *J* = 14.5, 5.8 Hz, 1H, SCH_2_), 4.18 (dd, *J* = 12.7, 9.3 Hz, 1H, NCH_2_), 4.56 (dd, *J* = 12.8, 3.4 Hz, 1H, NCH_2_), 5.17–5.21 (m, 1H, OCH), 7.12 (t, *J* = 7.5 Hz, 1H, 8-H), 7.18–7.20 (m, 1H, *BZX* 6-H), 7.20–7.23 (m, 1H, *BZX* 5-H), 7.25 (d, *J* = 8.4 Hz, 1H, 6-H), 7.31 (t, *J* = 7.6 Hz, 1H, 7-H), 7.36 (d, *J* = 7.9 Hz, 1H, *BZX* 7-H), 7.39 (s, 1H, 10-H), 7.49 (d, *J* = 7.8 Hz, 1H, *BZX* 4-H), 7.66 (d, *J* = 8.1 Hz, 1H, 9-H) ppm. ^13^C NMR (176 MHz, CDCl_3_): *δ* = 33.5 (SCH_2_), 43.0 (NCH_2_), 76.0 (OCH), 110.1 (C-6), 110.2 (*BZX* C-7), 110.7 (C-10), 118.6 (*BZX* C-4), 121.7 (C-8), 122.9 (C-10a), 123.4 (C-9), 124.4 (*BZX* C-6), 124.6 (*BZX* C-5), 126.3 (C-7), 127.1 (C-9a), 136.8 (C-5a), 141.5 (*BZX* C-3a), 152.2 (*BZX* C-7a), 158.8 (C=O), 163.3 (*BZX* C-2) ppm. ^15^N NMR (71 MHz, CDCl_3_): *δ* = −253.9 (N-5), −144.3 (*BZX* N-1) ppm. IR (KBr): 3050 (C–H_arom_), 1716 (C=O), 1537, 1499, 1470, 1453, 1413, 1381, 1348, 1322 (C=C, C–N_arom_, CH_2_), 1241, 1203, 1167, 1129, 1090, 1076, 1027 (C–O–C), 959, 927, 807, 729 (C=C) cm^−1^. MS *m*/*z* (%): 351 ([M + H]^+^, 100). HRMS (ESI) for C_19_H_15_N_2_O_3_S ([M + H]^+^) calculated 351.0798, found 351.0797.

#### 3-[(Pyrimidin-2-ylthio)methyl]-3,4-dihydro-1*H*-[1,4]oxazino[4,3-*a*]indol-1-one (16c)

Compound 16c was obtained as a light brown solid, yield 78 mg (50%), mp 156.4–156.9 °C. *R*_f_ = 0.555 (ethyl acetate/hexane 2 : 1). ^1^H NMR (700 MHz, CDCl_3_): *δ* = 3.37 (dd, *J* = 14.3, 8.5 Hz, 1H, SCH_2_), 3.79 (dd, *J* = 14.3, 5.0 Hz, 1H, SCH_2_), 4.14 (dd, *J* = 12.7, 9.0 Hz, 1H, NCH_2_), 4.54 (dd, *J* = 12.7, 3.4 Hz, 1H, NCH_2_), 5.03–5.06 (m, 1H, OCH), 6.94 (t, *J* = 4.8 Hz, 1H, *Pyr* 5-H), 7.12 (t, *J* = 7.5 Hz, 1H, 8-H), 7.23 (d, *J* = 8.4 Hz, 1H, 6-H), 7.30–7.32 (m, 1H, 7-H), 7.37 (s, 1H, 10-H), 7.66 (d, *J* = 8.1 Hz, 1H, 9-H), 8.42 (d, *J* = 4.9 Hz, 2H, *Pyr* 4,6-H) ppm. ^13^C NMR (176 MHz, CDCl_3_): *δ* = 31.9 (SCH_2_), 42.8 (NCH_2_), 76.5 (OCH), 110.0 (C-6), 110.3 (C-10), 117.2 (*Pyr* C-6), 121.5 (C-8), 123.2 (C-10a), 123.3 (C-9), 126.1 (C-7), 127.0 (C-9a), 136.8 (C-5a), 157.6 (*Pyr* C-4,6), 159.2 (C=O), 170.5 (*Pyr* C-2) ppm. ^15^N NMR (71 MHz, CDCl_3_): *δ* = −253.2 (N-5), −98.0 (*Pyr* N-1, N-3) ppm. IR (KBr): 2918 (C–H_aliph_), 1723 (C=O), 1571, 1554, 1537, 1465, 1416, 1383, 1351, 1320 (C=C, C–N_arom_, CH_2_), 1240, 1201, 1180, 1087 (C–O–C), 956, 801, 752 (C=C) cm^−1^. MS *m*/*z* (%): 312 ([M + H]^+^, 100). HRMS (ESI) for C_16_H_13_N_3_NaO_2_S ([M + Na]^+^) calculated 334.0621, found 334.0624.

#### Synthetic procedure for 17

In a solution of 3-[(benzo[*d*]thiazol-2-ylthio)methyl]-3,4-dihydro-1*H*-[1,4]oxazino[4,3-*a*]indol-1-one 16a (183 mg, 0.5 mmol) in acetic acid (3 mL), Amberlyst 15 (31 mg, 0.1 mmol) was added, and the mixture was warmed to 50 °C. Hydrogen peroxide (30% w/w, 0.18 mL, 1.75 mmol) was slowly added dropwise, then stirring was applied for 3 h. After completion of the reaction (TLC monitoring), the mixture was diluted with water (20 mL) and extracted with ethyl acetate (3 × 20 mL). The combined organic layers were dried over Na_2_SO_4_ and concentrated under reduced pressure to provide the desired product 17.

#### 3-[(Benzo[*d*]thiazol-2-ylsulfinyl)methyl]-3,4-dihydro-1*H*-[1,4]oxazino[4,3-*a*]indol-1-one (17)

Compound 17 was obtained as an off-white solid, yield 156 mg (82%), mp 194.8–195.2 °C. *R*_f_ = 0.143 (ethyl acetate/hexane, 1 : 2 v/v). IR (KBr): 1710 (C=O), 1532, 1467, 1421, 1387, 1376, 1346, 1322 (C=C, C–N_arom_, CH_2_, CH_3_), 1239, 1203, 1165, 1139, 1087, 1058, 1026 (C–O–C, S=O), 1003, 959, 808, 757, 739, 725 (C=C) cm^−1^. MS *m*/*z* (%): 383 ([M + H]^+^, 100). HRMS (ESI) for C_19_H_14_N_2_NaO_3_S_2_ ([M + Na]^+^) calculated 405.0338, found 405.0336.

#### Major isomer

^1^H NMR (700 MHz, DMSO-*d*_6_): *δ* = 3.84–3.86 (m, 2H, SCH_2_), 4.37 (dd, *J* = 13.0, 9.4 Hz, 1H, NCH_2_), 4.74–4.75 (m, 1H, NCH_2_), 5.49–5.52 (m, 1H, OCH), 7.18–7.20 (m, 1H, 8-H), 7.39–7.40 (m, 1H, 7-H), 7.44 (s, 1H, 10-H), 7.49 (d, *J* = 8.5 Hz, 1H, 6-H), 7.62–7.67 (m, 2H, *BTh* 5,6-H), 7.77 (d, *J* = 8.1 Hz, 1H, 9-H), 8.14 (d, *J* = 8.1 Hz, 1H, *BTh* 4-H), 8.32 (d, *J* = 8.2 Hz, 1H, *BTh* 7-H) ppm. ^13^C NMR (176 MHz, DMSO-*d*_6_): *δ* = 43.2 (NCH_2_), 58.9 (SCH_2_), 72.3 (OCH), 109.7 (C-10), 111.4 (C-6), 121.7 (C-8), 123.2 (C-9), 123.5 (C-9a), 123.7 (*BTh* C-7), 124.2 (*BTh* C-4), 126.3 (C-7), 126.8 (C-10a), 127.0 (*BTh* C-6), 127.8 (*BTh* C-5), 135.9 (*BTh* C-7a), 136.9 (C-5a), 153.6 (*BTh* C-3a), 158.6 (C=O), 177.8 (*BTh* C-2) ppm. ^15^N NMR (71 MHz DMSO-*d*_6_): *δ* = −249.6 (N-5), −66.3 (*BTh* N-2) ppm.

#### Minor isomer

^1^H NMR (700 MHz, DMSO-*d*_6_): *δ* = 3.81–3.83 (m, 1H, SCH_2_), 4.02 (dd, *J* = 14.3, 3.8 Hz, 1H, SCH_2_), 4.41 (dd, *J* = 13.0, 9.6 Hz, 1H, NCH_2_), 4.76–4.77 (m, 1H, NCH_2_), 5.47–5.48 (m, 1H, OCH), 7.20–7.21 (m, 1H, 8-H), 7.36 (s, 1H, 10-H), 7.41–7.42 (m, 1H, 7-H), 7.55 (d, *J* = 8.4 Hz, 1H, 6-H), 7.57–7.61 (m, 2H, *BTh* 5,6-H), 7.75 (d, *J* = 8.1 Hz, 1H, 9-H), 8.12 (d, *J* = 8.2 Hz, 1H, *BTh* 4-H), 8.30 (d, *J* = 8.2 Hz, 1H, *BTh* 7-H) ppm. ^13^C NMR (176 MHz, DMSO-*d*_6_): *δ* = 43.3 (NCH_2_), 57.4 (SCH_2_), 72.4 (OCH), 109.5 (C-10), 111.5 (C-6), 121.7 (C-8), 123.2 (C-9), 123.5 (C-9a), 123.6 (*BTh* C-7), 124.1 (*BTh* C-4), 126.2 (C-7), 126.8 (*BTh* C-6), 127.0 (C-10a), 127.6 (*BTh* C-5), 136.2 (*BTh* C-7a), 136.9 (C-5a), 153.9 (*BTh* C-3a), 158.4 (C=O), 178.0 (*BTh* C-2) ppm. ^15^N NMR (71 MHz DMSO-*d*_6_): *δ* = −249.1 (N-5), −66.8 (*BTh* N-2) ppm.

#### Synthetic procedure for 18

In a solution of 3-[(benzo[*d*]thiazol-2-ylthio)methyl]-3,4-dihydro-1*H*-[1,4]oxazino[4,3-*a*]indol-1-one 16a (183 mg, 0.5 mmol) in DCM, *m*-chloroperoxybenzoic acid (70%, 1.5 mmol, 370 mg) was added and the mixture was stirred at room temperature for 6 h. After completion of the reaction (TLC monitoring), mixture was diluted with 1 M Na_2_CO_3_ (20 mL) and extracted with DCM (3 × 20 mL), then washed with brine. The combined organic layers were dried over Na_2_SO_4_ and concentrated reduced pressure to provide the desired product 18.

#### 3-[(Benzo[*d*]thiazol-2-ylsulfonyl)methyl]-3,4-dihydro-1*H*-[1,4]oxazino[4,3-*a*]indol-1-one (18)

Compound 18 was obtained as an off-white solid, yield 183 mg (92%), mp 214.2–214.7 °C. *R*_f_ = 0.285 (ethyl acetate/hexane, 1 : 2 v/v). ^1^H NMR (700 MHz, DMSO-*d*_6_): *δ* = 4.36 (dd, *J* = 13.0, 9.2 Hz, 1H, SCH_2_), 4.43 (dd, *J* = 15.4, 3.3 Hz, 1H, NCH_2_), 4.48 (dd, *J* = 15.4, 8.7 Hz, 1H, SCH_2_), 4.71 (dd, *J* = 13.0, 3.5 Hz, 1H, NCH_2_), 5.49–5.53 (m, 1H, OCH) 7.19 (t, *J* = 7.4 Hz, 1H, Ar), 7.34 (s, 1H, 10-H), 7.41 (t, *J* = 7.7 Hz, 1H, Ar), 7.51 (d, *J* = 8.4 Hz, 1H, Ar), 7.71–7.75 (m, 3H, Ar), 8.27 (d, *J* = 7.7 Hz, 1H, Ar), 8.38 (d, *J* = 7.6 Hz, 1H, Ar) ppm. ^13^C NMR (176 MHz, DMSO-*d*_6_): *δ* = 42.9 (NCH_2_), 56.1 (SCH_2_), 72.7 (OCH), 109.6 (CH), 111.4 (CH), 121.7 (CH), 123.2 (CH), 124.0 (CH), 125.4 (CH), 126.3 (CH), 126.8, 128.4 (CH), 128.7 (CH), 136.9, 137.0, 152.6, 158.0, 167.0 ppm. IR (KBr): 1738 (C=O), 1538, 1467, 1374, 1348, 1326, 1309, 1255 (C–N_arom_, C=C, CH_2_, S=O), 1238, 1199, 1148, 1084, 1030 (C–O–C, S=O), 761, 740, 729 (C=C) cm^−1^. MS *m*/*z* (%): 399 ([M + H]^+^, 100). HRMS (ESI) for C_19_H_14_N_2_NaO_4_S_2_ ([M + Na]^+^) calculated 421.0287, found 421.0285.

#### Synthetic procedure for 19

3-(Hydroxymethyl)-3,4-dihydro-1*H*-[1,4]oxazino[4,3-*a*]indol-1-one 6a (434 mg, 2 mmol) was dissolved in DCM, TEA (0.42 mL, 3 mmol) was added, and the mixture was cooled to 0 °C. Tosyl chloride (456 mg, 2.4 mmol) was then added portion wise, and the mixture was stirred at room temperature for 24 h. After completion of the reaction (TLC monitoring), the mixture was diluted with water (20 mL) and extracted with DCM (3 × 20 mL). The combined organic layers were dried over Na_2_SO_4_ and concentrated under vacuum. The obtained residue was purified *via* column chromatography on silica gel (eluent ethyl acetate/hexane, 1 : 3 v/v) to provide product 19.

#### (1-Oxo-3,4-dihydro-1*H*-[1,4]oxazino[4,3-*a*]indol-3-yl)methyl 4-methylbenzenesulfonate (19)

Compound 19 was obtained as a white solid, yield 608 mg (82%), mp 166.4–166.9 °C. *R*_f_ = 0.539 (ethyl acetate/hexane, 1 : 1 v/v). ^1^H NMR (400 MHz, CDCl_3_): *δ* = 2.45 (s, 3H, CH_3_), 4.18 (dd, *J* = 12.2, 10.2 Hz, 1H, NCH_2_), 4.27 (dd, *J* = 10.9, 6.3 Hz, 1H, OCH_2_), 4.39 (dd, *J* = 10.9, 4.2 Hz, 1H, OCH_2_), 4.46 (dd, *J* = 12.9, 3.0 Hz, 1H, NCH_2_), 4.92–5.01 (m, OCH), 7.21 (t, *J* = 7.5 Hz, 1H, 8-H), 7.32–7.36 (m, 3H, *Ph* 3,5-H; 6-H), 7.41–7.44 (m, 2H, 10-H; 7-H), 7.72 (d, *J* = 8.1 Hz, 1H, 9-H), 7.79 (d, *J* = 8.0 Hz, 2H, *Ph* 2,6-H) ppm. ^13^C NMR (101 MHz, CDCl_3_): *δ* = 21.7 (CH_3_), 41.2 (NCH_2_), 67.2 (OCH_2_), 74.2 (OCH), 110.1 (C-6), 110.9 (C-10), 121.8 (C-8), 122.5 (C-10a), 123.3 (C-9), 126.5 (C-7), 127.0 (C-9a), 128.1 (*Ph* C-2,6), 130.2 (*Ph* C-3,5), 131.8(*Ph* C-1), 136.9 (C-5a), 145.8 (*Ph* C-4), 158.2 (C=O) ppm. ^15^N NMR (41 MHz, CDCl_3_): *δ* = −255.3 (N-5) ppm. IR (KBr): 1711 (C=O), 1531, 1377, 1365, 1345 (C–N_arom_, C=C_,_ S=O), 1247, 1190, 1090, 1070, 1032 (C–O–C, S=O), 966, 940, 927, 820, 810, 765, 759, 670 (C=C) cm^−1^. MS *m*/*z* (%): 372 ([M + H]^+^, 100). HRMS (ESI) for C_19_H_18_NO_5_S ([M + H]^+^) calculated 372.0900, found 372.0893.

#### General synthetic procedure for 20a–c

A mixture of the appropriate aza-heterocycle (0.5 mmol), Cs_2_CO_3_ (244 mg, 0.75 mmol) and tosylate 19 (0.5 mmol, 186 mg) in anhydrous DMF was stirred at 80 °C for 1–3 h. After completion of the reaction (TLC monitoring), the mixture was diluted with water (20 mL) and extracted with ethyl acetate (3 × 20 mL). The combined organic layers were dried over Na_2_SO_4_ and concentrated under vacuum. The obtained residue was purified *via* column chromatography on silica gel to provide products 20a–c.

#### 3-(Morpholinomethyl)-3,4-dihydro-1*H*-[1,4]oxazino[4,3-*a*]indol-1-one (20a)

Compound 20a was obtained as a white solid, yield 76 mg (53%), mp 167.2–167.7 °C. *R*_f_ = 0.152 (ethyl acetate/dichloromethane, 1 : 3 v/v). ^1^H NMR (400 MHz, CDCl_3_): *δ* = 2.55–2.65 (m, 4H, *MPh* 2,6-H), 2.78 (dd, *J* = 13.4, 6.9 Hz, 1H, *MPh* NCH_2_), 2.88 (dd, *J* = 13.4, 5.0 Hz, 1H, *MPh* NCH_2_), 3.69–3.77 (m, 4H, *MPh* 3,5-H), 4.16 (dd, *J* = 12.2, 10.3 Hz, 1H, NCH_2_), 4.48 (dd, *J* = 12.9, 2.9 Hz, 1H, NCH_2_), 4.88–4.98 (m, 1H, OCH), 7.21 (t, *J* = 7.3 Hz, 1H, 8-H), 7.36–7.42 (m, 2H, 6-H; 7-H), 7.44 (s, 1H, 10-H), 7.75 (d, *J* = 8.1 Hz, 1H, 9-H) ppm.^13^C NMR (101 MHz, CDCl_3_): *δ* = 43.0 (NCH_2_), 54.5 (*MPh* C-2,6), 59.7 (*MPh* NCH_2_), 66.9 (*MPh* C-3,5), 75.8 (OCH), 110.0 (C-6), 110.2 (C-10), 121.5 (C-8), 123.3 (C-9; C-10a), 126.1 (C-7), 127.0 (C-9a), 136.7 (C-5a), 159.5 (C=O) ppm. ^15^N (41 MHz, CDCl_3_): *δ* = −342.8 (N-1 *MPh*), −252.8 (N-5) ppm. IR (KBr): 2956, 2851 (C–H_aliph_), 1712 (C=O), 1536, 1471, 1457, 1417, 1378, 1353, 1322 (C=C, CH_2_, C–N_arom_), 1246, 1199, 1166, 1150, 1109, 1075 (C–O–C, C–N_aliph_), 957, 859, 808, 741 (C=C) cm^−1^. MS *m*/*z* (%): 287 ([M + H]^+^, 100). HRMS (ESI) for C_16_H_18_N_2_NaO_3_ ([M + Na]^+^) calculated 309.1210, found 309.1210.

#### 3-[(1H-Pyrazol-1-yl)methyl]-3,4-dihydro-1*H*-[1,4]oxazino[4,3-*a*]indol-1-one (20b)

Compound 20b was obtained as a light brown solid, yield 47 mg (35%), mp 172.4–172.9 °C. *R*_f_ = 0.225 (ethyl acetate/hexane, 1 : 1 v/v). ^1^H NMR (400 MHz, CDCl_3_): *δ* = 3.85–3.91 (m, 1H, NCH_2_), 4.37 (d, *J* = 13.0 Hz, 1H, NCH_2_), 4.51–4.63 (m, 2H, *Py* NCH_2_), 5.03–5.06 (m, 1H, OCH), 6.25 (s, 1H, *Py* 4-H), 7.12 (t, *J* = 7.4 Hz, 1H, 8-H), 7.24 (d, *J* = 8.4 Hz, 1H, 6-H), 7.33 (t, *J* = 7.6 Hz, 1H, 7-H), 7.37 (s, 1H, 10-H), 7.51 (d, *J* = 7.2 Hz, 2H, *Py* 3,5-H), 7.65 (d, *J* = 8.1 Hz, 1H, 9-H) ppm. ^13^C NMR (101 MHz, CDCl_3_): *δ* = 42.0 (NCH_2_), 52.7 (*Py* NCH_2_), 76.1 (OCH), 106.6 (*Py* C-4), 110.0 (C-6), 110.7 (C-10), 121.6 (C-8), 122.7 (C-10a), 123.3 (C-9), 126.4 (C-7), 127.0 (C-9a), 131.1 (*Py* C-5), 136.8 (C-5a), 140.4 (*Py* C-3), 158.8 (C=O) ppm. ^15^N (41 MHz, CDCl_3_): *δ* = −254.0 (N-5), −181.2 (N-1 *Py*), −77.4 (N-2 *Py*) ppm. IR (KBr): 3118 (C–H_arom_), 2942 (C–H_aliph_), 1723 (C=O), 1535, 1516, 1466, 1417, 1398, 1377, 1352, 1317, 1282 (C=C, C–N_arom_, CH_2_), 1249, 1204, 1165, 1094, 1040 (C–O–C), 961, 856, 824, 742 (C=C) cm^−1^. MS *m*/*z* (%): 268 ([M + H]^+^, 100). HRMS (ESI) for C_15_H_13_N_3_NaO_2_ ([M + Na]^+^) calculated 290.0900, found 290.0900.

#### 3-[(1H-Benzo[*d*]imidazole-1-yl)methyl]-3,4-dihydro-1*H*-[1,4]oxazino[4,3-*a*]indol-1-one (20c)

Compound 20c was obtained as a white solid, yield 65 mg (41%), mp 237.4–237.9 °C. *R*_f_ = 0.184 (methanol/dichloromethane, 3 : 100 v/v). ^1^H NMR (700 MHz, DMSO-*d*_6_): *δ* = 4.18 (dd, *J* = 12.8, 10.3 Hz, 1H, NCH_2_), 4.77–4.82 (m, 2H, *BIM* NCH_2_) 4.87 (dd, *J* = 12.9, 3.3 Hz, 1H, NCH_2_), 5.36–5.40 (m, 1H, OCH), 7.19 (t, *J* = 7.5 Hz, 1H, 8-H), 7.25 (t, *J* = 7.6 Hz, 1H, *BIM* 6-H), 7.30 (t, *J* = 7.3 Hz, 1H, *BIM* 5-H), 7.35 (s, 1H, 10-H), 7.42 (t, *J* = 7.7 Hz, 1H, 7-H), 7.58 (d, *J* = 8.4 Hz, 1H, 6-H), 7.70 (d, *J* = 8.0 Hz, 1H, *BIM* 7-H), 7.74 (d, *J* = 8.1 Hz, 1H, 9-H), 7.76 (d, *J* = 8.1 Hz, 1H, *BIM* 4-H), 8.31 (s, 1H, *BIM* 2-H) ppm. ^13^C NMR (176 MHz, DMSO-*d*_6_): *δ* = 41.9 (NCH_2_), 45.9 (*BIM* NCH_2_), 76.7 (OCH), 109.3 (C-10), 111.3 (*BIM* C-4), 111.4 (C-6), 119.9 (*BIM* C-7), 121.7 (C-8), 122.3 (*BIM* C-6), 123.1 (*BIM* C-5), 123.2 (C-9), 123.7 (C-10a), 126.1 (C-7), 126.8 (C-9a), 134.5 (*BIM* C-3a), 136.9 (C-5a), 143.6 (*BIM* C-7a), 145.1 (*BIM* C-2), 158.9 (C=O) ppm. ^15^N NMR (71 MHz, DMSO-*d*_6_): *δ* = −249.9 (N-5), −233.7 (*BIM* N-3), −137.2 (*BIM* N-1) ppm. IR (KBr): 3088, 3050 (C–H_arom_), 1709 (C=O), 1613, 1536, 1499, 1465, 1417, 1374, 1352, 1314, 1288, 1269 (C=C, CH_2_, C–N_arom_), 1252, 1201, 1163, 1137, 1083 (C–O–C), 1009, 959, 892, 760, 746, 690 (C=C) cm^−1^. MS *m*/*z* (%): 287 ([M + H]^+^, 100). HRMS (ESI) for C_19_H_15_N_3_NaO_2_ ([M + Na]^+^) calculated 340.1056, found 340.1052.

#### Synthetic procedure for 21

To an ice-cold mixture of tosylate 19 (111 mg, 0.3 mmol) and Cs_2_CO_3_ (146 mg, 0.45 mmol) in anhydrous DMF under argon atmosphere, methyl thioglycolate (27 μl, 0.3 mmol) was added dropwise while stirring for 5 min. The reaction mixture was then heated to 80 °C for 1.5 h. After completion of the reaction (TLC monitoring), the mixture was diluted with water (20 mL) and extracted with ethyl acetate (3 × 20 mL). The combined organic layers were dried over Na_2_SO_4_ and concentrated under vacuum. The obtained residue was purified *via* column chromatography on silica gel (eluent ethyl acetate/hexane, 1 : 3 v/v) to provide product 21.

#### Methyl 2-{[(1-oxo-3,4-dihydro-1*H*-[1,4]oxazino[4,3-*a*]indol-3-yl)methyl]thio}acetate (21)

Compound 21 was obtained as a white solid, yield 56 mg (61%), mp 68.3–68.8 °C. *R*_f_ = 0.432 (ethyl acetate/hexane 1 : 2 v/v). ^1^H NMR (700 MHz, CDCl_3_): *δ* = 3.04 (dd, *J* = 14.4, 7.4 Hz, 1H, CH**CH**_2_S), 3.19 (dd, *J* = 14.4, 5.2 Hz, 1H, CH**CH**_2_S), 3.36–3.43 (m, 2H, S**CH**_2_C=O), 3.74 (s, 3H, CH_3_), 4.20 (dd, *J* = 12.6, 9.5 Hz, 1H, NCH_2_), 4.54 (dd, *J* = 12.7, 3.4 Hz, 1H, NCH_2_), 4.95–4.99 (m, 1H, OCH), 7.21 (t, *J* = 7.5 Hz, 1H, 8-H), 7.36 (d, *J* = 8.4 Hz, 1H, 6-H), 7.40–7.42 (m, 1H, 7-H), 7.43 (s, 1H, 10-H), 7.73 (d, *J* = 8.1 Hz, 1H, 9-H) ppm. ^13^C NMR (176 MHz, CDCl_3_): *δ* = 34.1 (CH**CH**_2_S), 34.3 (S**CH**_2_C=O), 43.0 (NCH_2_), 52.7 (CH_3_), 77.0 (OCH), 110.1 (C-6), 110.4 (C-10), 121.6 (C-8), 123.0 (C-10a), 123.3 (C-9), 126.2 (C-7), 127.0 (C-9a), 136.8 (C-5a), 159.0 (C-1), 170.5 (COO*Me* C=O) ppm. ^15^N NMR (71 MHz, CDCl_3_): *δ* = −253.5 (N-5) ppm. IR (KBr): 2954 (C–H_aliph_), 1739, 1717 (C=O), 1539, 1473, 1427, 1408, 1379, 1353, 1303 (C=C, CH_2_, CH_3_, C–N_arom_), 1250, 1218, 1201, 1153, 1120, 1074 (C–O–C), 1034, 996, 958, 741 (C=C) cm^−1^. MS *m*/*z* (%): 306 ([M + H]^+^, 100). HRMS (ESI) for C_15_H_15_NNaO_4_S ([M + H]^+^) calculated 328.0614, found 328.0616.

## Data availability

The data supporting this article have been included in the ESI.† Crystallographic data have been deposited at the Cambridge Crystallographic Data Centre (https://www.ccdc.cam.ac.uk/services/structures) with CCDC reference number 2428119 for compound 6a and CCDC reference number 2428115 for compound 18.

## Author contributions

Conceptualization, A. Š.; methodology, A. Š., E. A. and P. R.; formal analysis, A. Š. and E. A.; investigation, I. Z., S. B., G. R. and A. B.; resources, A. Š. and E.A.; data curation, A. Š., I. Z., A. B. and E. A.; writing—original draft preparation, A. Š., E. A., I.Z. and A.B.; writing—review and editing, A.Š., P. R. and E. A.; visualization, A. Š., I.Z. and A. B.; supervision, E. A. and A. Š.; funding acquisition, A. Š. and E. A. All authors have read and agreed to the published version of the manuscript.

## Conflicts of interest

There are no conflicts to declare.

## Supplementary Material

RA-015-D5RA02996A-s001

RA-015-D5RA02996A-s002
