# Supplementary material for: A new synthetic approach to the 3,4-dihydro-1H-[1,4]oxazino[4,3-a]indole system from ethyl 1H-indole-2-carboxylates and activated glycerol carbonate
Source: RSC Adv. 2025 Jun 5;15(24):18947–70. doi: 10.1039/d5ra02996a (PMC12138336; doi:10.1039/d5ra02996a)
Supplement: RA-015-D5RA02996A-s001 [file RA-015-D5RA02996A-s001.pdf]

Supplementary Information (SI) for RSC Advances.
This journal is © The Royal Society of Chemistry 2025

Supplementary materials

A new synthetic approach to the 3,4-dihydro-1*H*-[1,4]oxazino[4,3-*a*]indole system from ethyl 1*H*-indole-2-carboxylates and activated glycerol carbonate

Inesa Zagorskytė,[a] Eglė Arbačiauskienė,[a]* Greta Račkauskienė,[b] Sergey Belyakov,[c] Aurimas Bieliauskas,[b] Patrick Rollin,[d] Algirdas Šačkus [a,b]*

[a]Department of Organic Chemistry, Kaunas University of Technology, Radvilėnų pl. 19, Kaunas LT-50254 Kaunas, Lithuania; egle.arbaciauskiene@ktu.lt

[b]Institute of Synthetic Chemistry, Kaunas University of Technology, K. Baršausko g. 59, Kaunas LT-51423, Lithuania; algirdas.sackus@ktu.lt

[c]Latvian Institute of Organic Synthesis, Aizkraukles 21, LV-1006 Riga, Latvia

[c]Université d'Orléans et CNRS, ICOA, UMR 7311, BP 6759, F-45067 Orléans, France

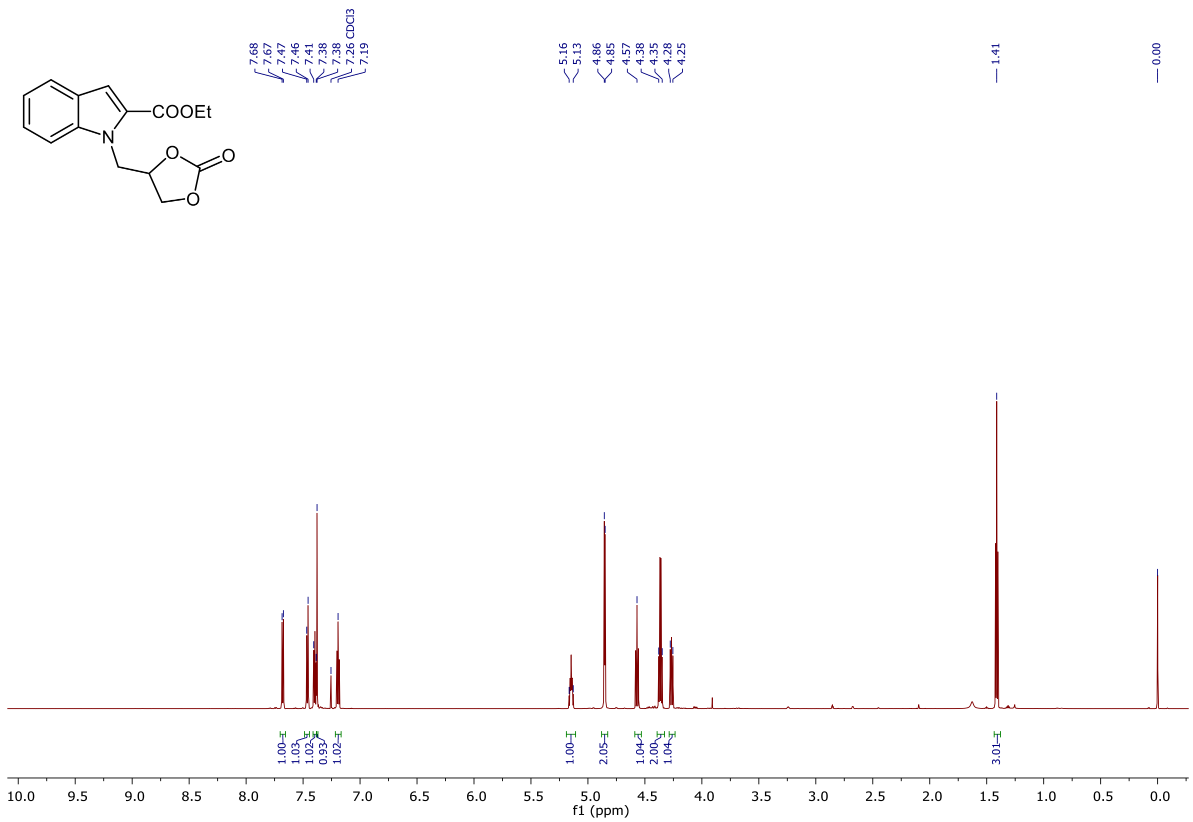

Figure S1. ^{1}H NMR (700 MHz, $CDCl_3$) spectrum of **4a**

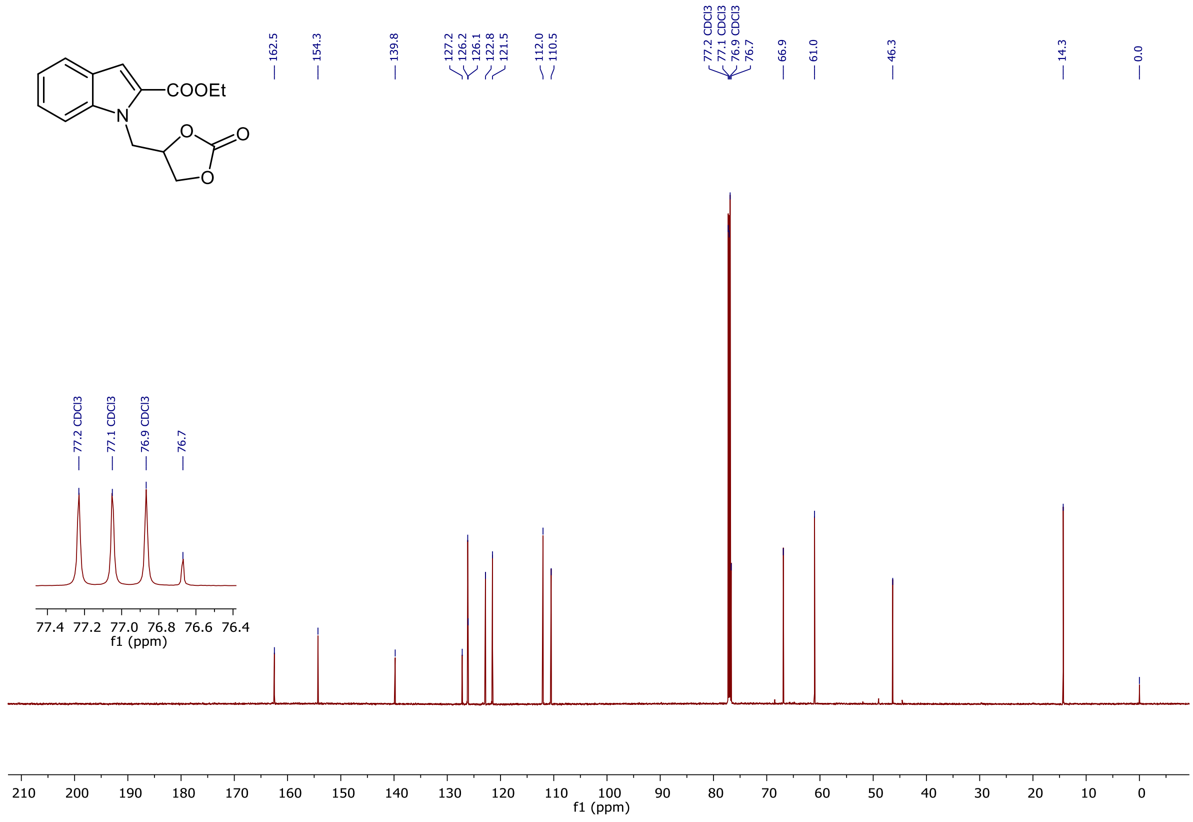

Figure S2. ^{13}C NMR (176 MHz, $CDCl_3$) spectrum of **4a**

Compound Spectrum SmartFormula Report

Analysis Info

Analysis Name	D:\Data\IZ-208.d	Acquisition Date	5/6/2025 10:51:40 PM
Method	DirectInfusion_TuneLow_pos.m	Operator	hplc
Sample Name	IZ-208	Instrument	micrOTOF-Q III 8228888.20448
Comment	AB		

Acquisition Parameter

Source Type	ESI	Ion Polarity	Positive	Set Nebulizer	0.4 Bar
Focus	Not active	Set Capillary	4500 V	Set Dry Heater	180 °C
Scan Begin	50 m/z	Set End Plate Offset	-500 V	Set Dry Gas	4.0 l/min
Scan End	1000 m/z	Set Collision Cell RF	140.0 Vpp	Set Divert Valve	Waste

#	RT [min]	Area	Int. Type	I	S/N	Chromatogram	Max. m/z	FWHM [min]
n.a.	85.8	n.a.	Single spectrum	n.a.	n.a.	n.a.	312.0839	n.a.

+MS, 85.8min #5142

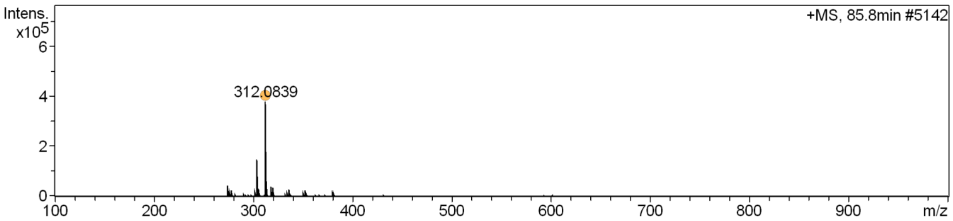

Meas. m/z	#	Ion Formula	m/z	err [ppm]	mSigma	# Sigma	Score	rdb	e⁻ Conf	N-Rule
312.0839	1	C15H15NNaO5	312.0842	0.9	5.4	1	100.00	8.5	even	ok

Figure S3. HRMS (ESI) report of **4a**

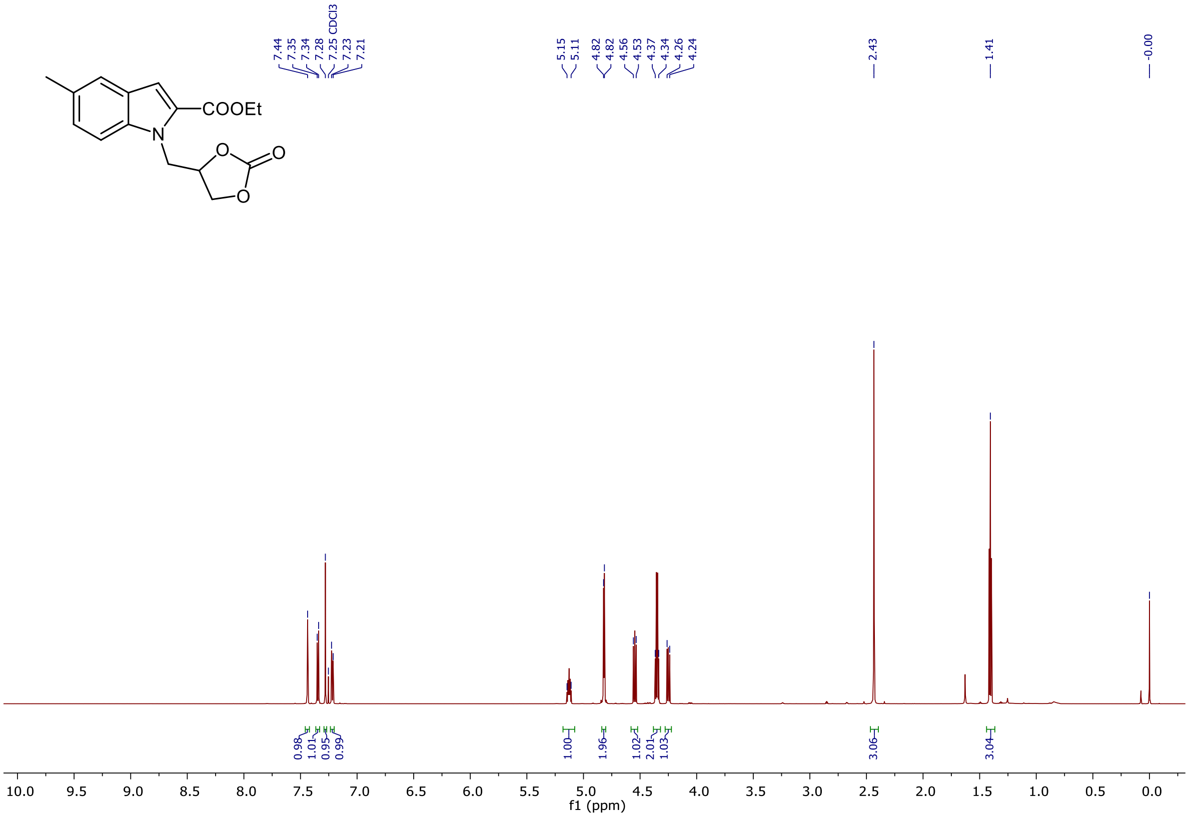

Figure S4. ^{1}H NMR (700 MHz, $CDCl_3$) spectrum of **4b**

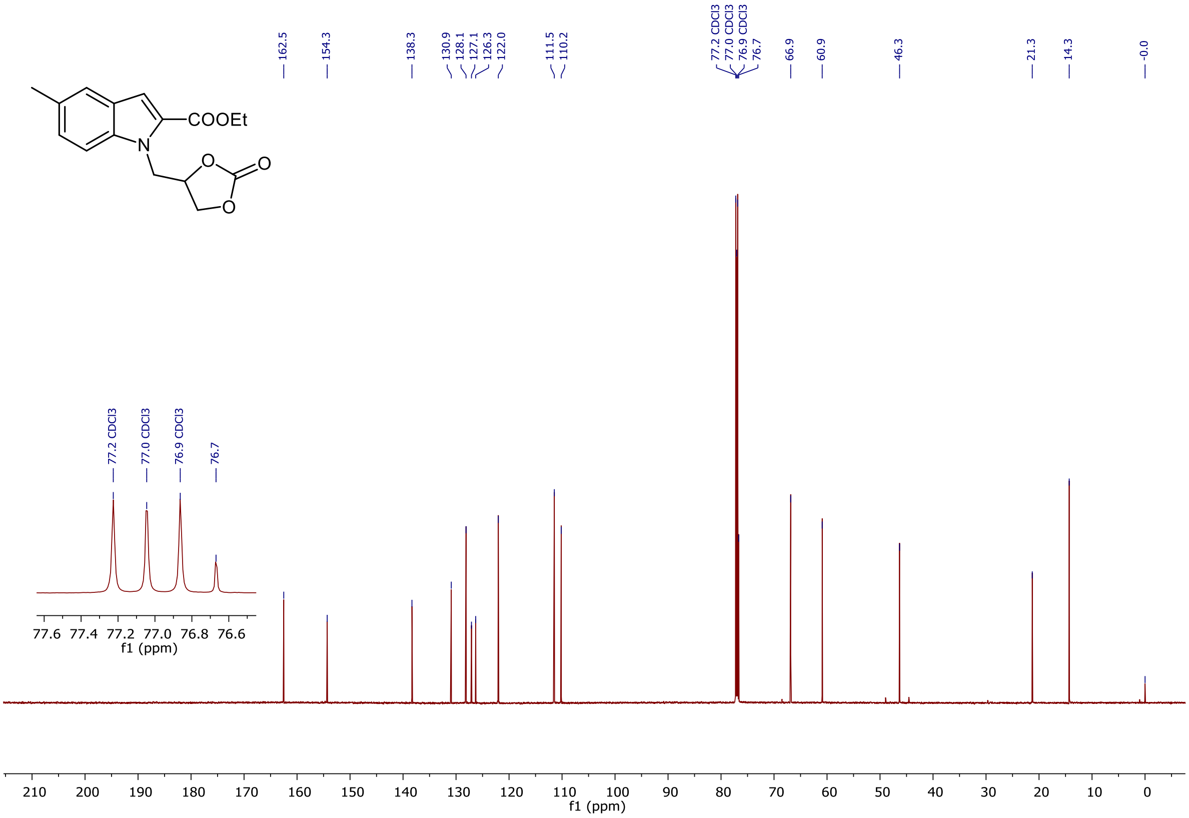

Figure S5. ^{13}C NMR (176 MHz, $CDCl_3$) spectrum of **4b**

Compound Spectrum SmartFormula Report

Analysis Info

Analysis Name	D:\Data\IZ-212.d	Acquisition Date	5/7/2025 12:27:54 AM
Method	DirectInfusion_TuneLow_pos.m	Operator	hplc
Sample Name	IZ-212	Instrument	micrOTOF-Q III 8228888.20448
Comment	AB		

Acquisition Parameter

Source Type	ESI	Ion Polarity	Positive	Set Nebulizer	0.4 Bar
Focus	Not active	Set Capillary	4500 V	Set Dry Heater	180 °C
Scan Begin	50 m/z	Set End Plate Offset	-500 V	Set Dry Gas	4.0 l/min
Scan End	1000 m/z	Set Collision Cell RF	140.0 Vpp	Set Divert Valve	Waste

#	RT [min]	Area	Int. Type	I	S/N	Chromatogram	Max. m/z	FWHM [min]
n.a.	5.7	n.a.	Single spectrum	n.a.	n.a.	n.a.	326.1003	n.a.

+MS, 5.7min #340

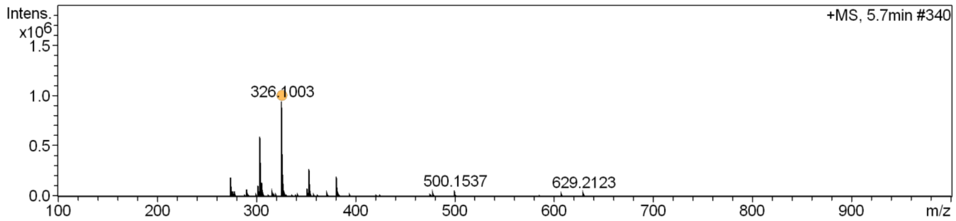

Meas. m/z	#	Ion Formula	m/z	err [ppm]	mSigma	# Sigma	Score	rdb	e⁻ Conf	N-Rule
326.1003	1	C16H17NNaO5	326.0999	-1.4	36.3	2	91.03	8.5	even	ok

Figure S6. HRMS (ESI) report of **4b**

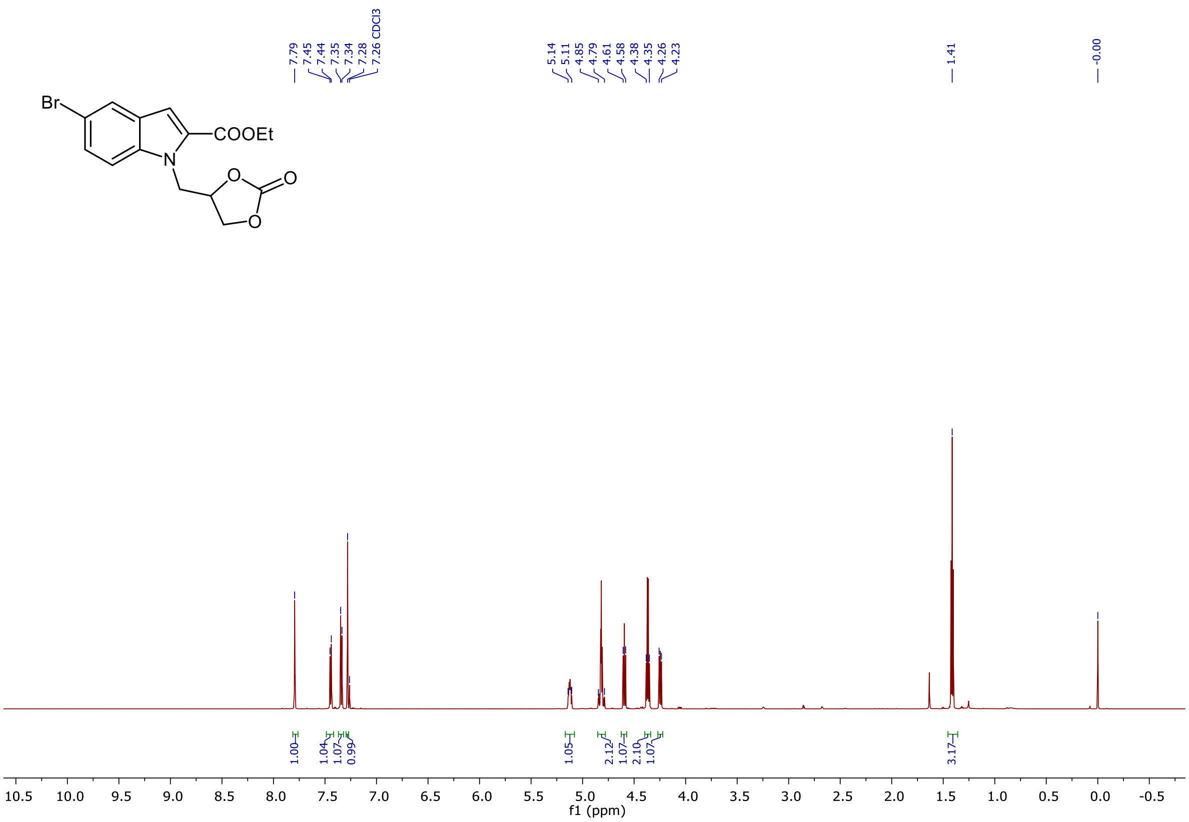

Figure S7. ^{1}H NMR (700 MHz, $CDCl_3$) spectrum of **4c**

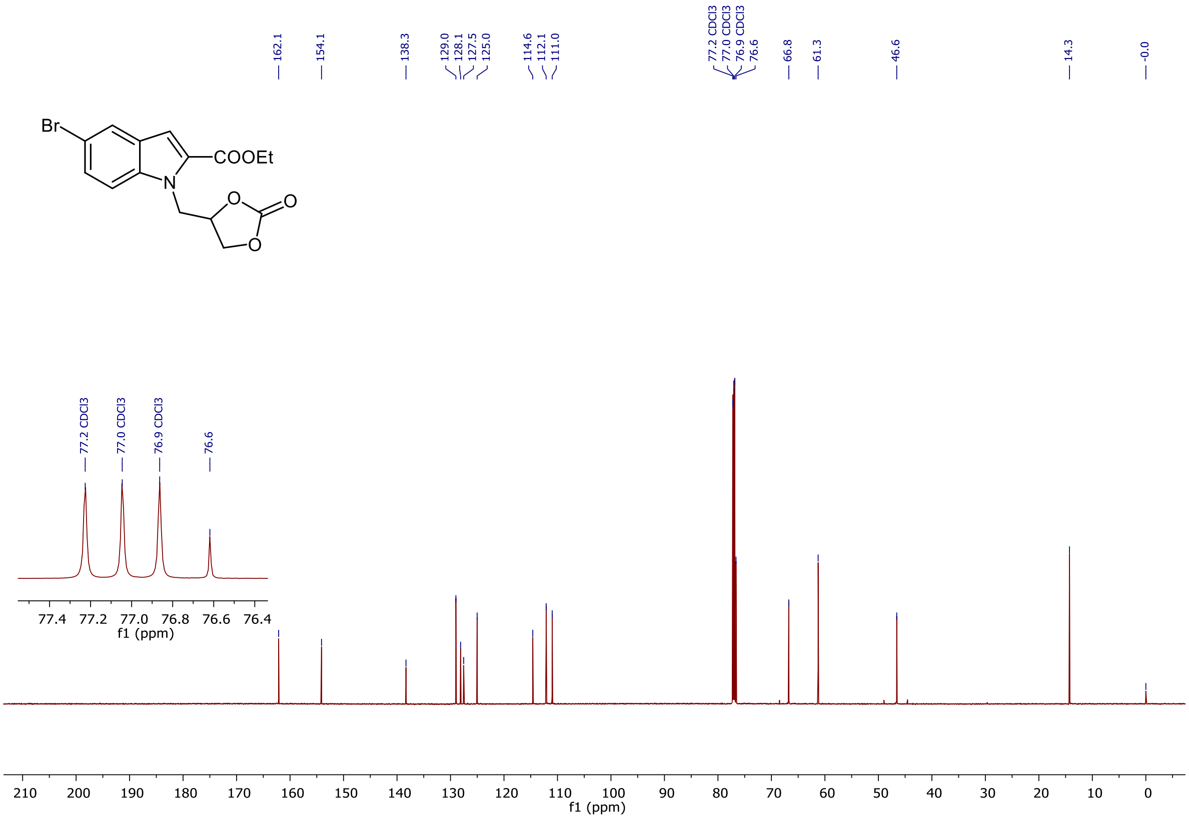

Figure S8. ^{13}C NMR (176 MHz, $CDCl_3$) spectrum of **4c**

Compound Spectrum SmartFormula Report

Analysis Info

Analysis Name	D:\Data\IZ-235_kartoju.d	Acquisition Date	3/17/2025 7:01:45 PM
Method	DirectInfusion_TuneLow_pos.m	Operator	hplc
Sample Name	IZ-235_kartoju	Instrument	micrOTOF-Q III 8228888.20448
Comment	AB		

Acquisition Parameter

Source Type	ESI	Ion Polarity	Positive	Set Nebulizer	0.4 Bar
Focus	Not active	Set Capillary	4500 V	Set Dry Heater	180 °C
Scan Begin	50 m/z	Set End Plate Offset	-500 V	Set Dry Gas	4.0 l/min
Scan End	1000 m/z	Set Collision Cell RF	140.0 Vpp	Set Divert Valve	Waste

#	RT [min]	Area	Int. Type	I	S/N	Chromatogram	Max. m/z	FWHM [min]
n.a.	3.6	n.a.	Single spectrum	n.a.	n.a.	n.a.	389.9952	n.a.

+MS, 3.6min #218

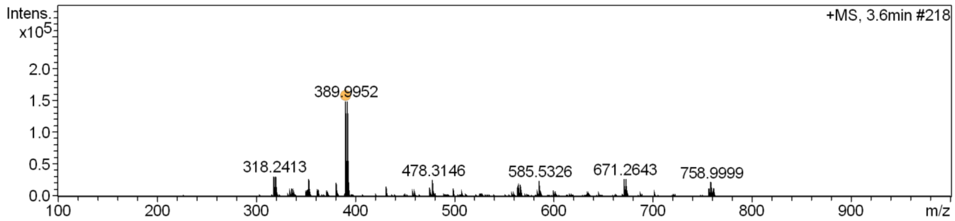

Meas. m/z	#	Ion Formula	m/z	err [ppm]	mSigma	# Sigma	Score	rdb	e⁻ Conf	N-Rule
389.9952	1	C15H14BrNNaO5	389.9948	-1.0	7.2	1	100.00	8.5	even	ok

Figure S9. HRMS (ESI) report of **4c**

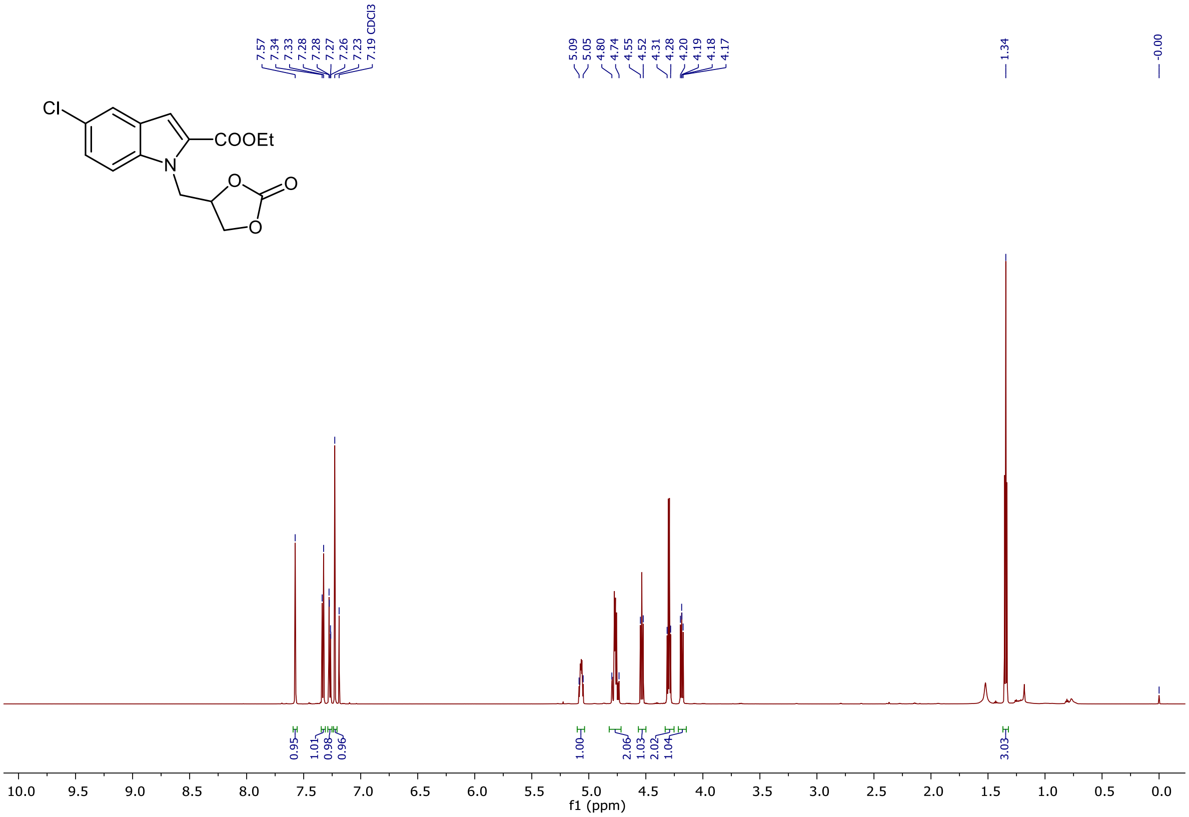

Figure S10. ^{1}H NMR (700 MHz, $CDCl_3$) spectrum of **4d**

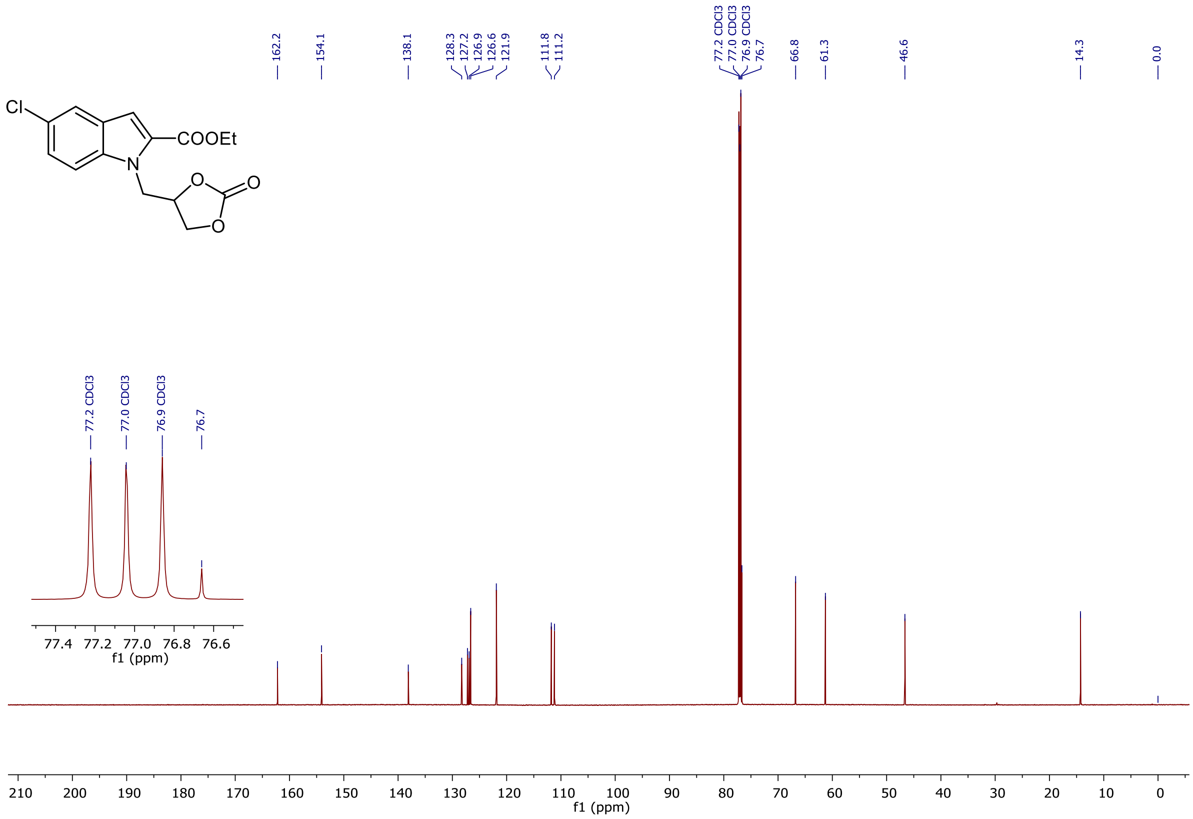

Figure S11. ^{13}C NMR (176 MHz, $CDCl_3$) spectrum of **4d**

Compound Spectrum SmartFormula Report

Analysis Info

Analysis Name	D:\Data\IZ-236.d	Acquisition Date	5/7/2025 4:00:57 AM
Method	DirectInfusion_TuneLow_pos.m	Operator	hplc
Sample Name	IZ-236	Instrument	micrOTOF-Q III 8228888.20448
Comment	AB		

Acquisition Parameter

Source Type	ESI	Ion Polarity	Positive	Set Nebulizer	0.4 Bar
Focus	Not active	Set Capillary	4500 V	Set Dry Heater	180 °C
Scan Begin	50 m/z	Set End Plate Offset	-500 V	Set Dry Gas	4.0 l/min
Scan End	1000 m/z	Set Collision Cell RF	140.0 Vpp	Set Divert Valve	Waste

#	RT [min]	Area	Int. Type	I	S/N	Chromatogram	Max. m/z	FWHM [min]
n.a.	2.8	n.a.	Single spectrum	n.a.	n.a.	n.a.	346.0454	n.a.

+MS, 2.8min #169

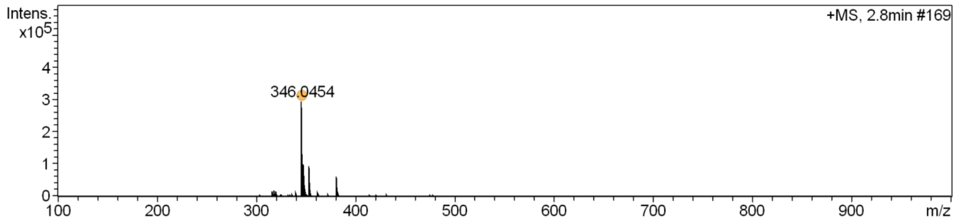

Meas. m/z	#	Ion Formula	m/z	err [ppm]	mSigma	# Sigma	Score	rdb	e⁻ Conf	N-Rule
346.0454	1	C15H14ClNNaO5	346.0453	-0.4	3.8	1	100.00	8.5	even	ok

Figure S12. HRMS (ESI) report of **4d**

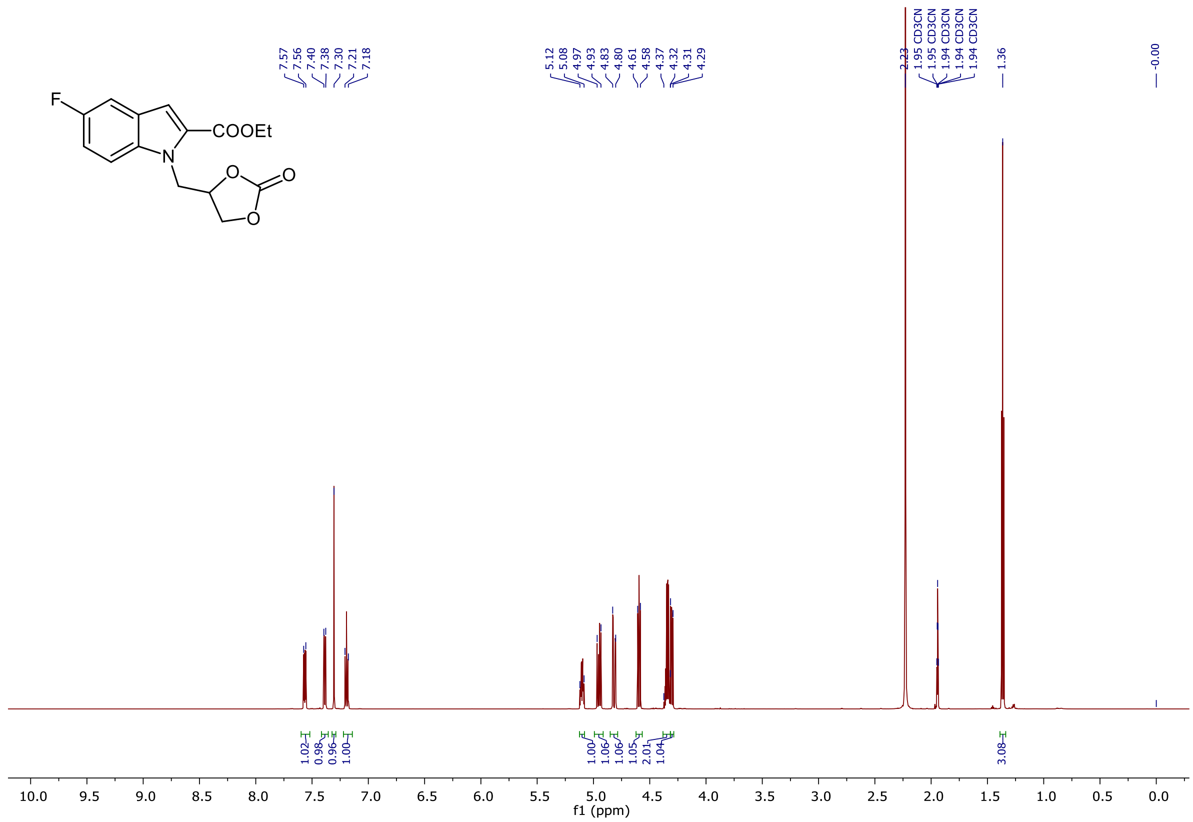

Figure S13. ^{1}H NMR (700 MHz, CD_3CN) spectrum of **4e**

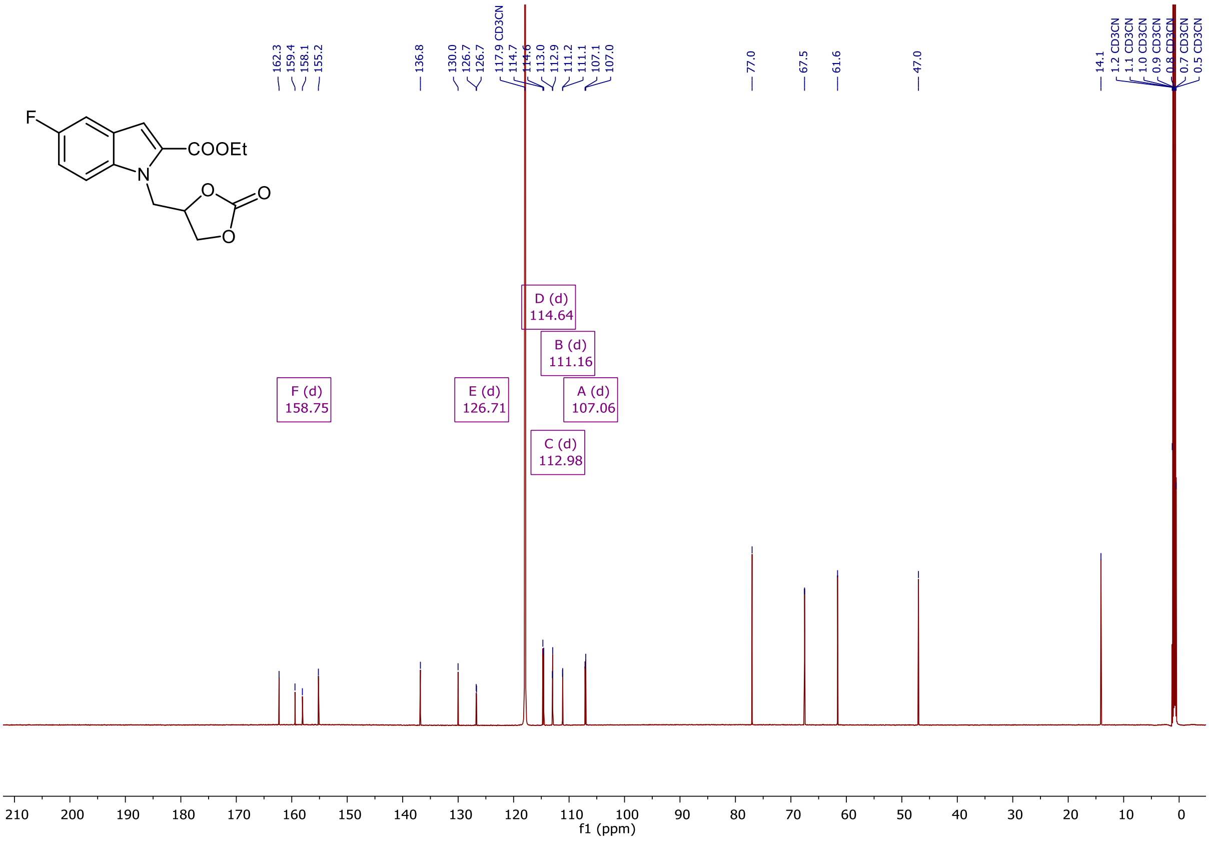

Figure S14. ^{13}C NMR (176 MHz, CD_3CN) spectrum of **4e**

Compound Spectrum SmartFormula Report

Analysis Info

Analysis Name	D:\Data\IZ-237.d	Acquisition Date	12/18/2024 9:22:42 AM
Method	DirectInfusion_TuneLow_pos.m	Operator	hplc
Sample Name	IZ-237	Instrument	micrOTOF-Q III 8228888.20448
Comment	SB		

Acquisition Parameter

Source Type	ESI	Ion Polarity	Positive	Set Nebulizer	0.4 Bar
Focus	Not active	Set Capillary	4500 V	Set Dry Heater	180 °C
Scan Begin	50 m/z	Set End Plate Offset	-500 V	Set Dry Gas	4.0 l/min
Scan End	1000 m/z	Set Collision Cell RF	140.0 Vpp	Set Divert Valve	Waste

#	RT [min]	Area	Int. Type	I	S/N	Chromatogram	Max. m/z	FWHM [min]
n.a.	0.4	n.a.	Single spectrum	n.a.	n.a.	n.a.	304.2630	n.a.
n.a.	6.9	n.a.	Single spectrum	n.a.	n.a.	n.a.	330.0747	n.a.

+MS, 6.9min #414

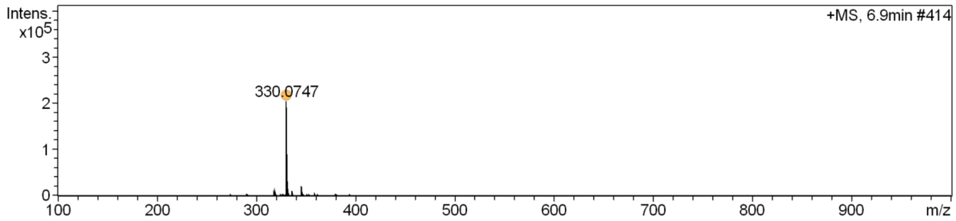

Meas. m/z	#	Ion Formula	m/z	err [ppm]	mSigma	# Sigma	Score	rdb	e⁻ Conf	N-Rule
330.0747	1	C15H14FNNaO5	330.0748	-0.2	3.6	1	100.00	8.5	even	ok

Figure S15. HRMS (ESI) report of **4e**

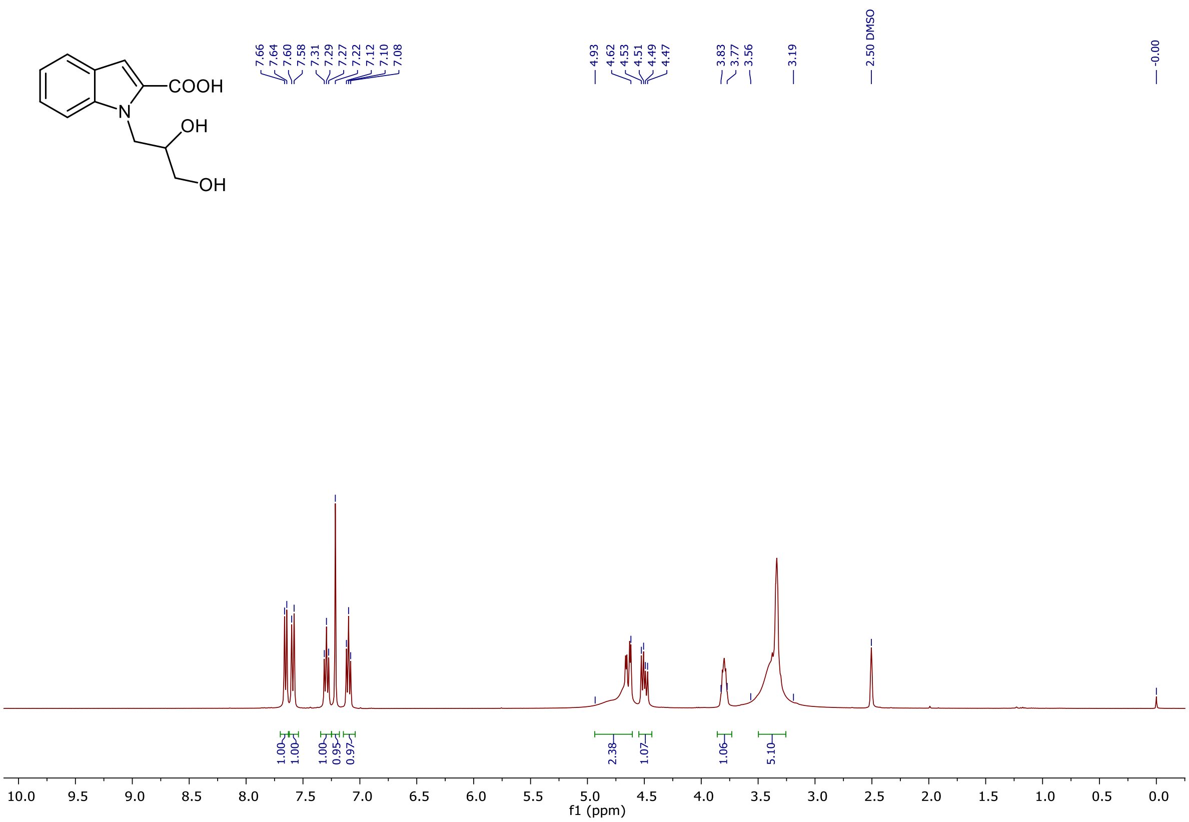

Figure S16. ^{1}H NMR (400 MHz, DMSO-d_6) spectrum of **5a**

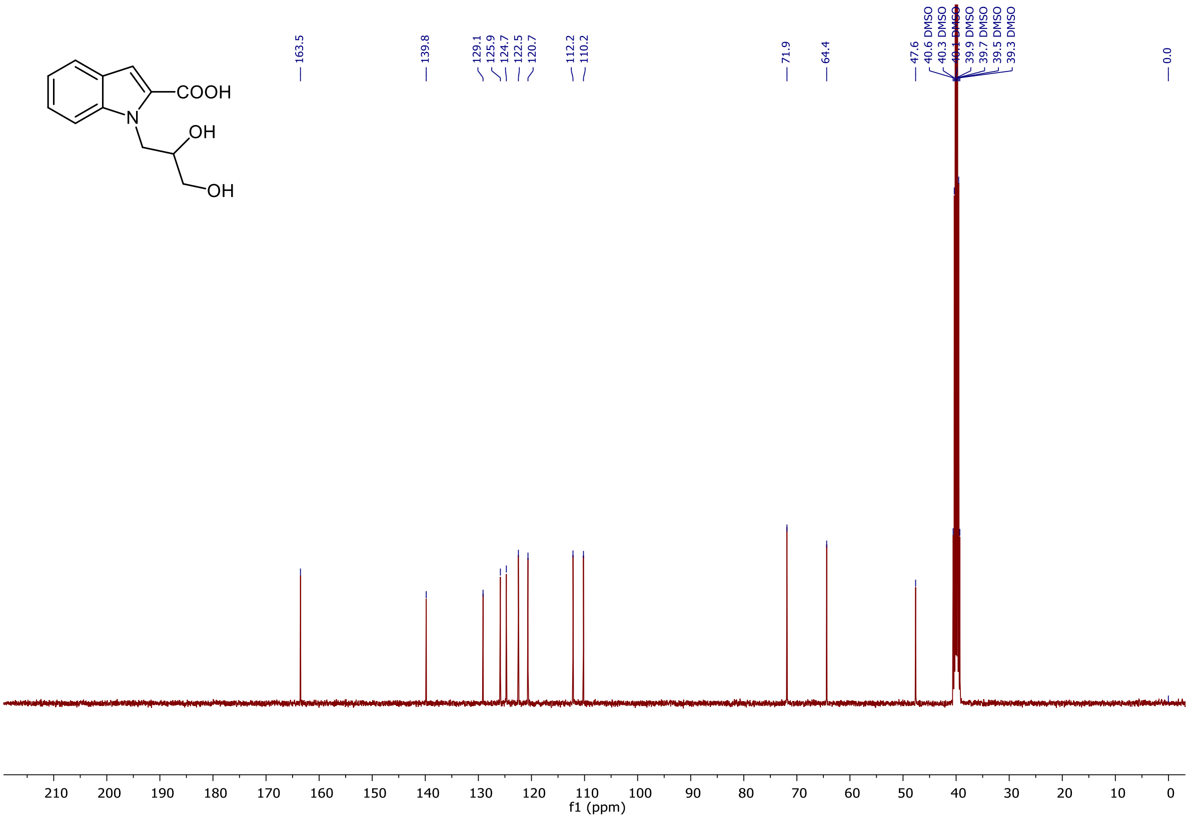

Figure S17. ^{13}C NMR (101 MHz, DMSO-d_6) spectrum of **5a**

Compound Spectrum SmartFormula Report

Analysis Info

Analysis Name	D:\Data\IZ-212_tikrasis_5a.d	Acquisition Date	5/7/2025 12:45:49 AM
Method	DirectInfusion_TuneLow_pos.m	Operator	hplc
Sample Name	IZ-212_tikrasis_5a	Instrument	micrOTOF-Q III 8228888.20448
Comment	AB		

Acquisition Parameter

Source Type	ESI	Ion Polarity	Positive	Set Nebulizer	0.4 Bar
Focus	Not active	Set Capillary	4500 V	Set Dry Heater	180 °C
Scan Begin	50 m/z	Set End Plate Offset	-500 V	Set Dry Gas	4.0 l/min
Scan End	1000 m/z	Set Collision Cell RF	140.0 Vpp	Set Divert Valve	Waste

#	RT [min]	Area	Int. Type	I	S/N	Chromatogram	Max. m/z	FWHM [min]
n.a.	3.0	n.a.	Single spectrum	n.a.	n.a.	n.a.	258.0740	n.a.

+MS, 3.0min #177

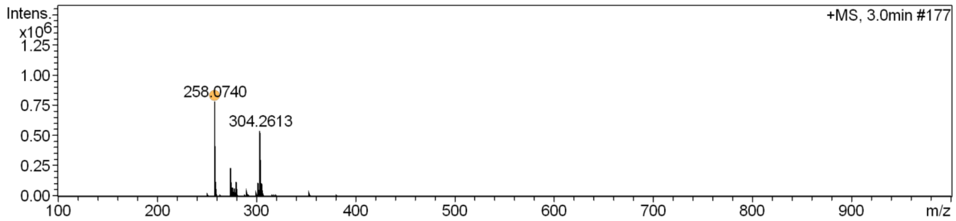

Meas. m/z	#	Ion Formula	m/z	err [ppm]	mSigma	# Sigma	Score	rdb	e⁻ Conf	N-Rule
258.0740	1	C12H13NNaO4	258.0737	1.1	10.1	2	100.00	6.5	even	ok

Figure S18. HRMS (ESI) report of **5a**

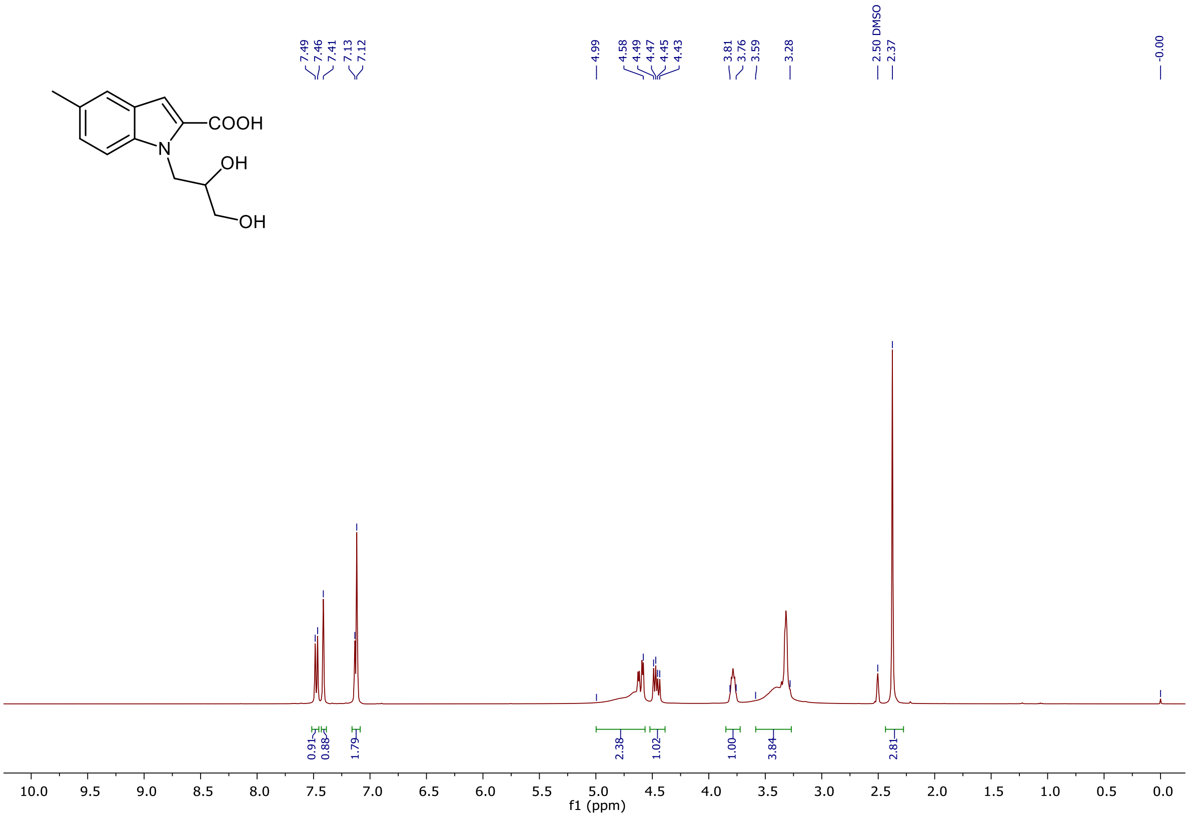

Figure S19. ^{1}H NMR (400 MHz, DMSO-d_6) spectrum of **5b**

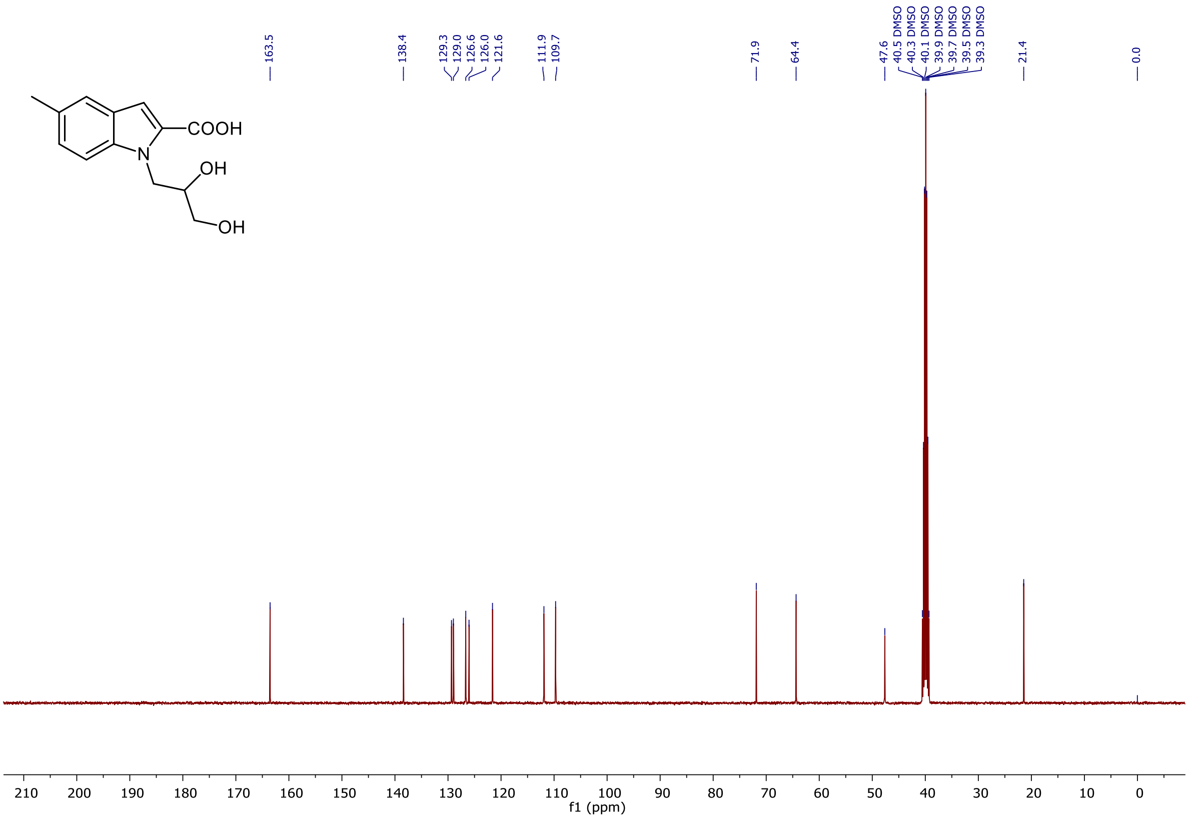

Figure S20. ^{13}C NMR (101 MHz, DMSO-d_6) spectrum of **5b**

Compound Spectrum SmartFormula Report

Analysis Info

Analysis Name	D:\Data\IZ-243.d	Acquisition Date	5/7/2025 2:25:40 AM
Method	DirectInfusion_TuneLow_pos.m	Operator	hplc
Sample Name	IZ-243	Instrument	micrOTOF-Q III 8228888.20448
Comment	AB		

Acquisition Parameter

Source Type	ESI	Ion Polarity	Positive	Set Nebulizer	0.4 Bar
Focus	Not active	Set Capillary	4500 V	Set Dry Heater	180 °C
Scan Begin	50 m/z	Set End Plate Offset	-500 V	Set Dry Gas	4.0 l/min
Scan End	1000 m/z	Set Collision Cell RF	140.0 Vpp	Set Divert Valve	Waste

#	RT [min]	Area	Int. Type	I	S/N	Chromatogram	Max. m/z	FWHM [min]
n.a.	10.7	n.a.	Single spectrum	n.a.	n.a.	n.a.	272.0895	n.a.

+MS, 10.7min #639

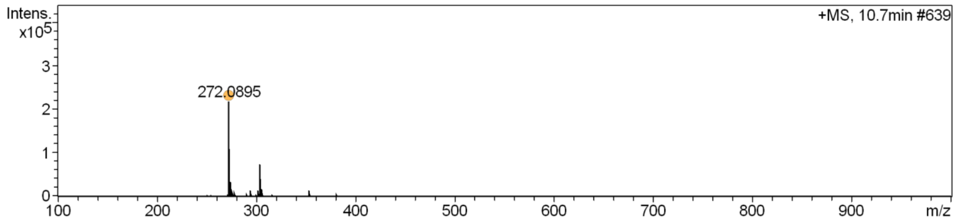

Meas. m/z	#	Ion Formula	m/z	err [ppm]	mSigma	# Sigma	Score	rdb	e^- Conf	N-Rule
272.0895	1	C13H15NNaO4	272.0893	0.5	3.1	1	100.00	6.5	even	ok

Figure S21. HRMS (ESI) report of **5b**

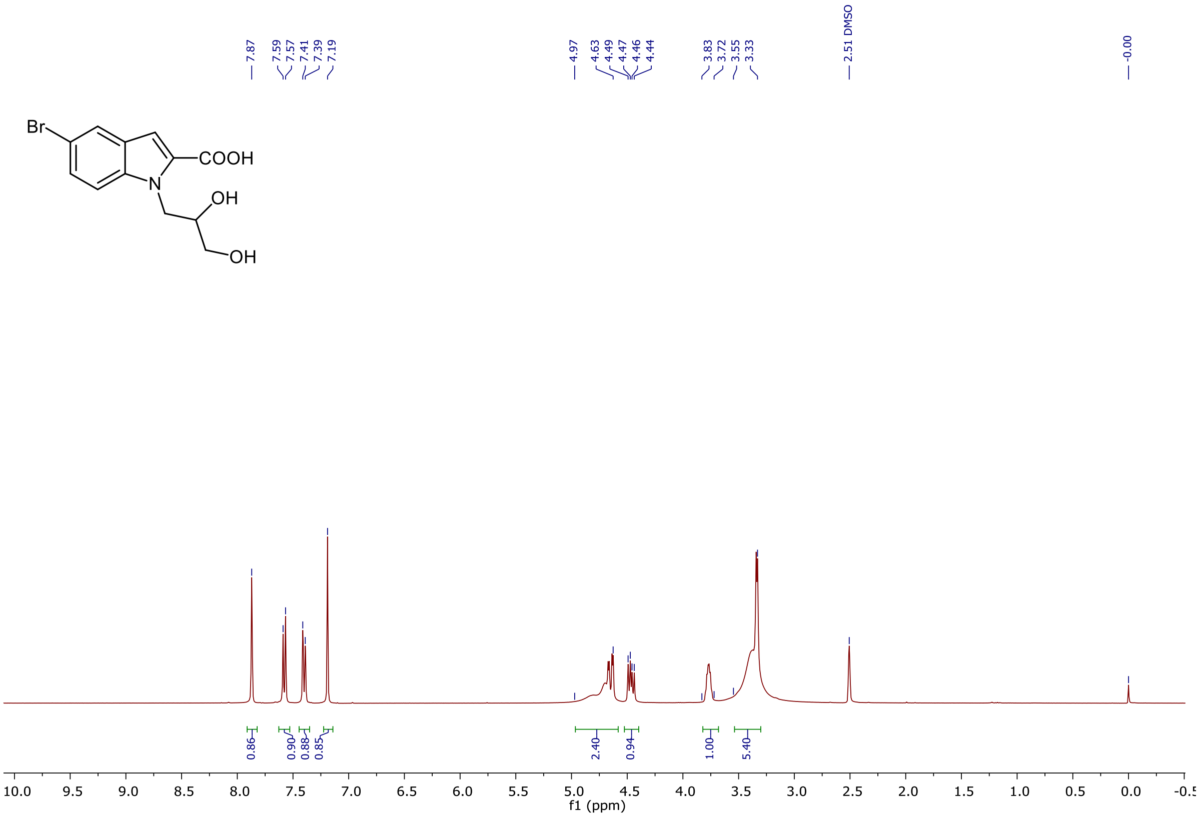

Figure S22. ^{1}H NMR (400 MHz, DMSO-d_6) spectrum of **5c**

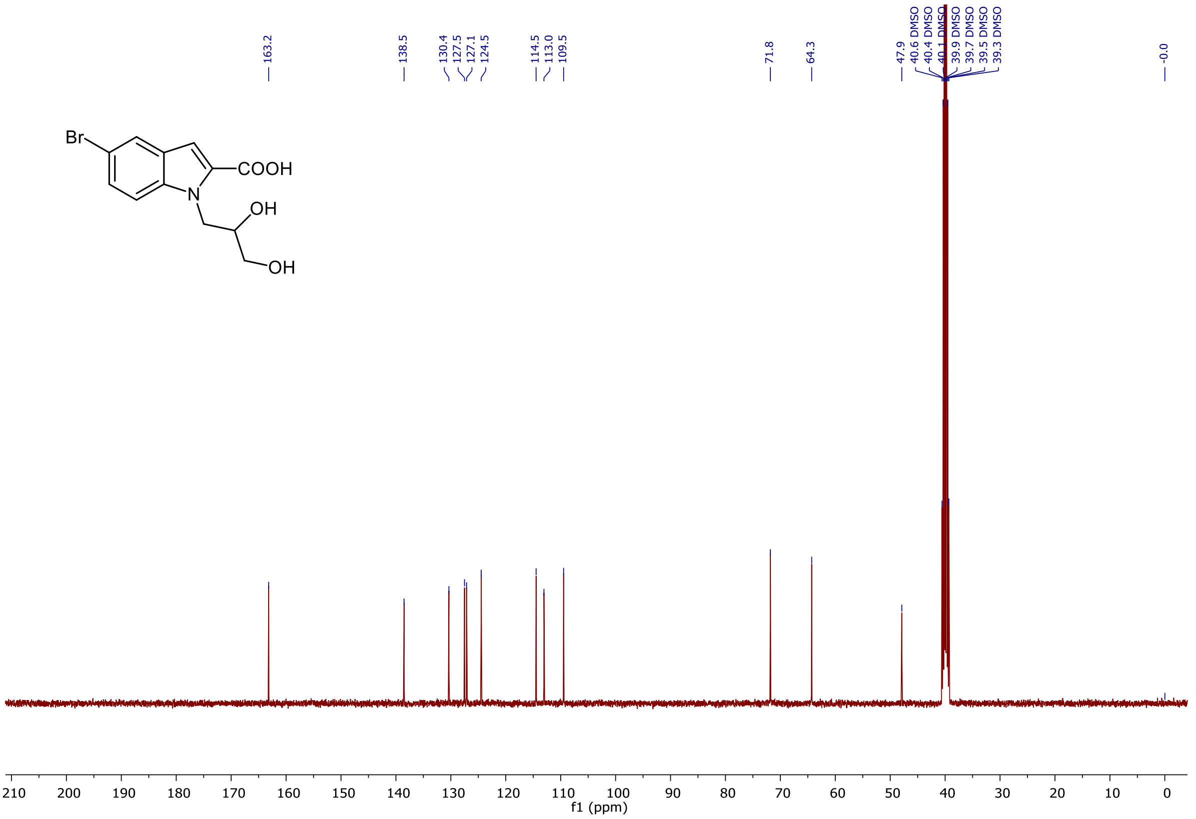

Figure S23. ^{13}C NMR (101 MHz, DMSO-d_6) spectrum of **5c**

Compound Spectrum SmartFormula Report

Analysis Info

Analysis Name	D:\Data\IZ-249.d	Acquisition Date	5/7/2025 1:44:39 AM
Method	DirectInfusion_TuneLow_pos.m	Operator	hplc
Sample Name	IZ-249	Instrument	micrOTOF-Q III 8228888.20448
Comment	AB		

Acquisition Parameter

Source Type	ESI	Ion Polarity	Positive	Set Nebulizer	0.4 Bar
Focus	Not active	Set Capillary	4500 V	Set Dry Heater	180 °C
Scan Begin	50 m/z	Set End Plate Offset	-500 V	Set Dry Gas	4.0 l/min
Scan End	1000 m/z	Set Collision Cell RF	140.0 Vpp	Set Divert Valve	Waste

#	RT [min]	Area	Int. Type	I	S/N	Chromatogram	Max. m/z	FWHM [min]
n.a.	4.7	n.a.	Single spectrum	n.a.	n.a.	n.a.	335.9844	n.a.

+MS, 4.7min #283

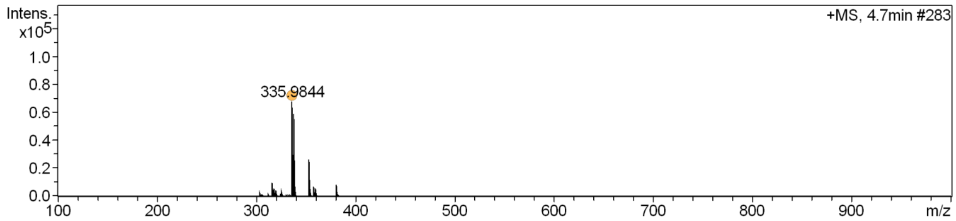

Meas. m/z	#	Ion Formula	m/z	err [ppm]	mSigma	# Sigma	Score	rdb	e⁻ Conf	N-Rule
335.9844	1	C12H12BrNNaO4	335.9842	0.6	57.4	2	100.00	6.5	even	ok

Figure S24. HRMS (ESI) report of **5c**

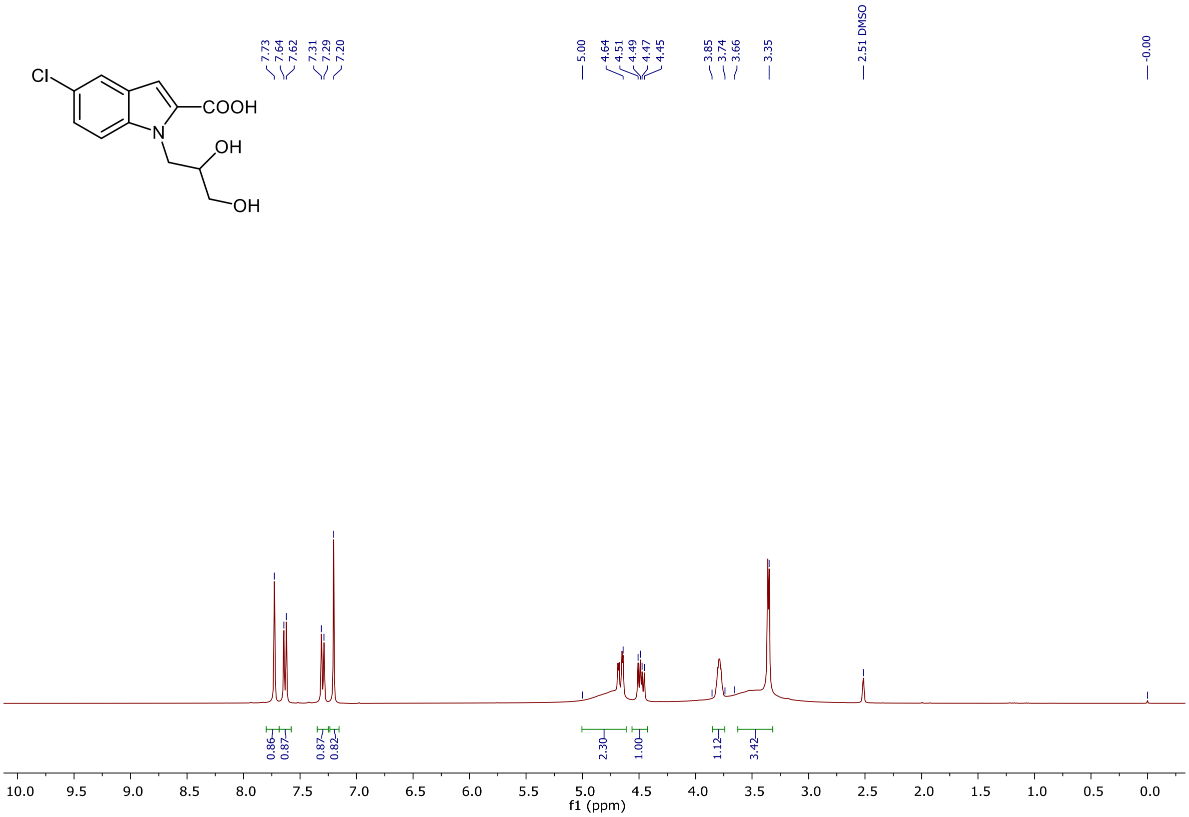

Figure S25. ^{1}H NMR (400 MHz, DMSO-d_6) spectrum of **5d**

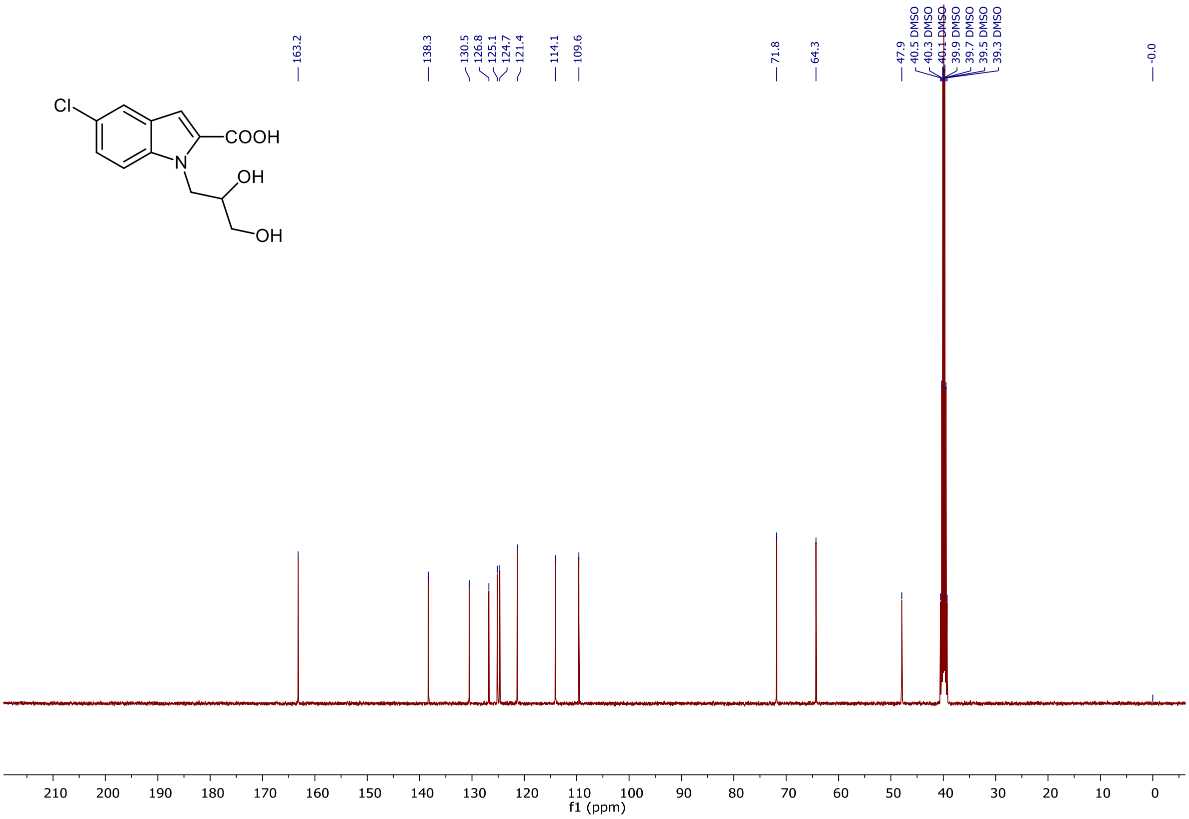

Figure S26. ^{13}C NMR (101 MHz, DMSO-d_6) spectrum of **5d**

Compound Spectrum SmartFormula Report

Analysis Info

Analysis Name	D:\Data\IZ-260.d	Acquisition Date	5/7/2025 3:00:51 AM
Method	DirectInfusion_TuneLow_pos.m	Operator	hplc
Sample Name	IZ-260	Instrument	micrOTOF-Q III 8228888.20448
Comment	AB		

Acquisition Parameter

Source Type	ESI	Ion Polarity	Positive	Set Nebulizer	0.4 Bar
Focus	Not active	Set Capillary	4500 V	Set Dry Heater	180 °C
Scan Begin	50 m/z	Set End Plate Offset	-500 V	Set Dry Gas	4.0 l/min
Scan End	1000 m/z	Set Collision Cell RF	140.0 Vpp	Set Divert Valve	Waste

#	RT [min]	Area	Int. Type	I	S/N	Chromatogram	Max. m/z	FWHM [min]
n.a.	4.4	n.a.	Single spectrum	n.a.	n.a.	n.a.	292.0350	n.a.

+MS, 4.4min #261

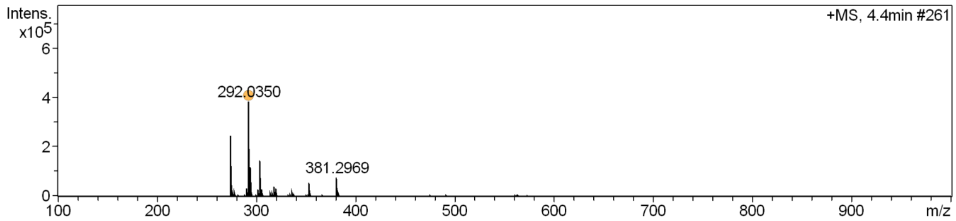

Meas. m/z	#	Ion Formula	m/z	err [ppm]	mSigma	# Sigma	Score	rdb	e⁻ Conf	N-Rule
292.0350	1	C12H12ClNNaO4	292.0347	-1.1	12.7	2	100.00	6.5	even	ok

Figure S27. HRMS (ESI) report of **5d**

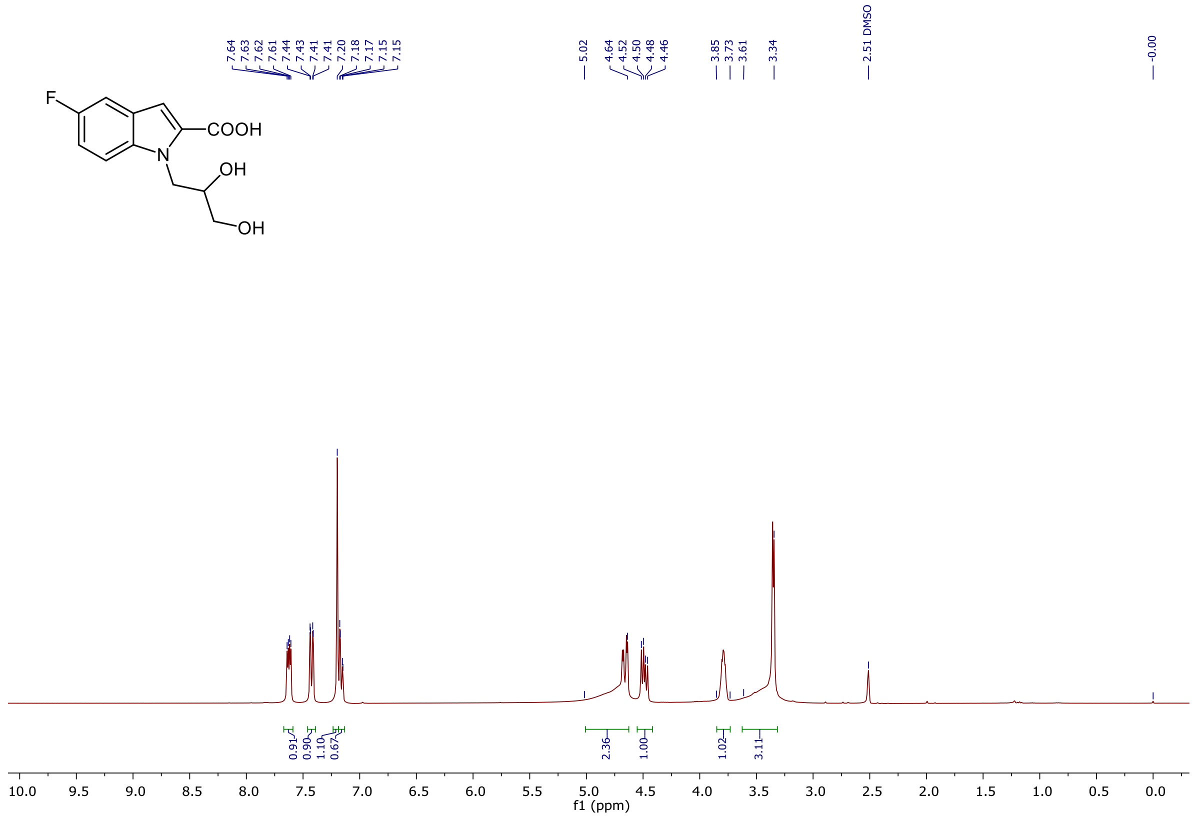

Figure S28. ^{1}H NMR (400 MHz, DMSO-d_6) spectrum of **5e**

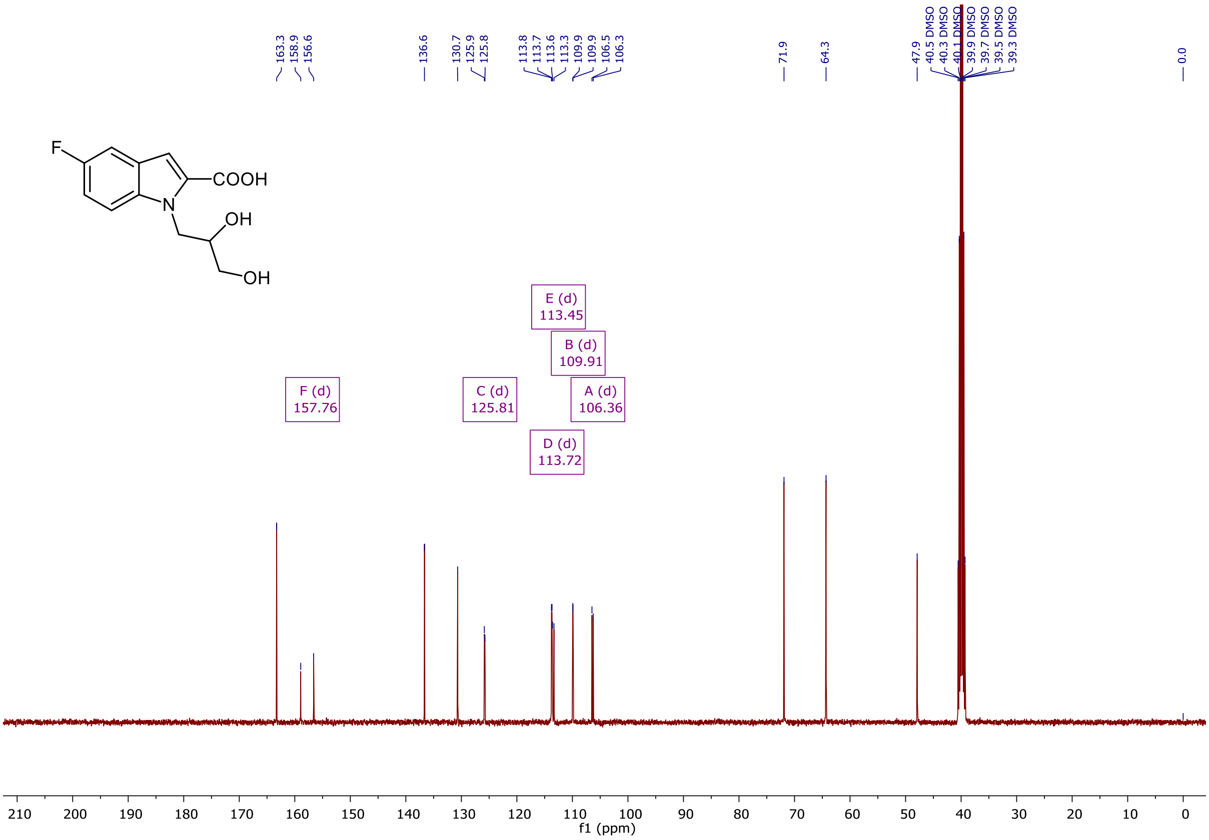

Figure S29. ^{13}C NMR (101 MHz, DMSO-d_6) spectrum of **5e**

Compound Spectrum SmartFormula Report

Analysis Info

Analysis Name	D:\Data\IZ-262.d	Acquisition Date	5/7/2025 2:07:31 AM
Method	DirectInfusion_TuneLow_pos.m	Operator	hplc
Sample Name	IZ-262	Instrument	micrOTOF-Q III 8228888.20448
Comment	AB		

Acquisition Parameter

Source Type	ESI	Ion Polarity	Positive	Set Nebulizer	0.4 Bar
Focus	Not active	Set Capillary	4500 V	Set Dry Heater	180 °C
Scan Begin	50 m/z	Set End Plate Offset	-500 V	Set Dry Gas	4.0 l/min
Scan End	1000 m/z	Set Collision Cell RF	140.0 Vpp	Set Divert Valve	Waste

#	RT [min]	Area	Int. Type	I	S/N	Chromatogram	Max. m/z	FWHM [min]
n.a.	6.7	n.a.	Single spectrum	n.a.	n.a.	n.a.	276.0646	n.a.

+MS, 6.7min #402

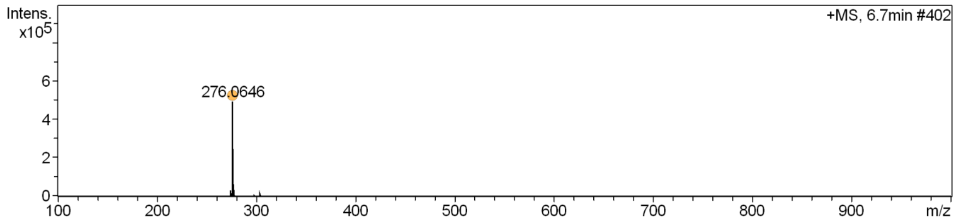

Meas. m/z	#	Ion Formula	m/z	err [ppm]	mSigma	# Sigma	Score	rdb	e⁻ Conf	N-Rule
276.0646	1	C12H12FNNaO4	276.0643	1.2	0.2	1	100.00	6.5	even	ok

Figure S30. HRMS (ESI) report of **5e**

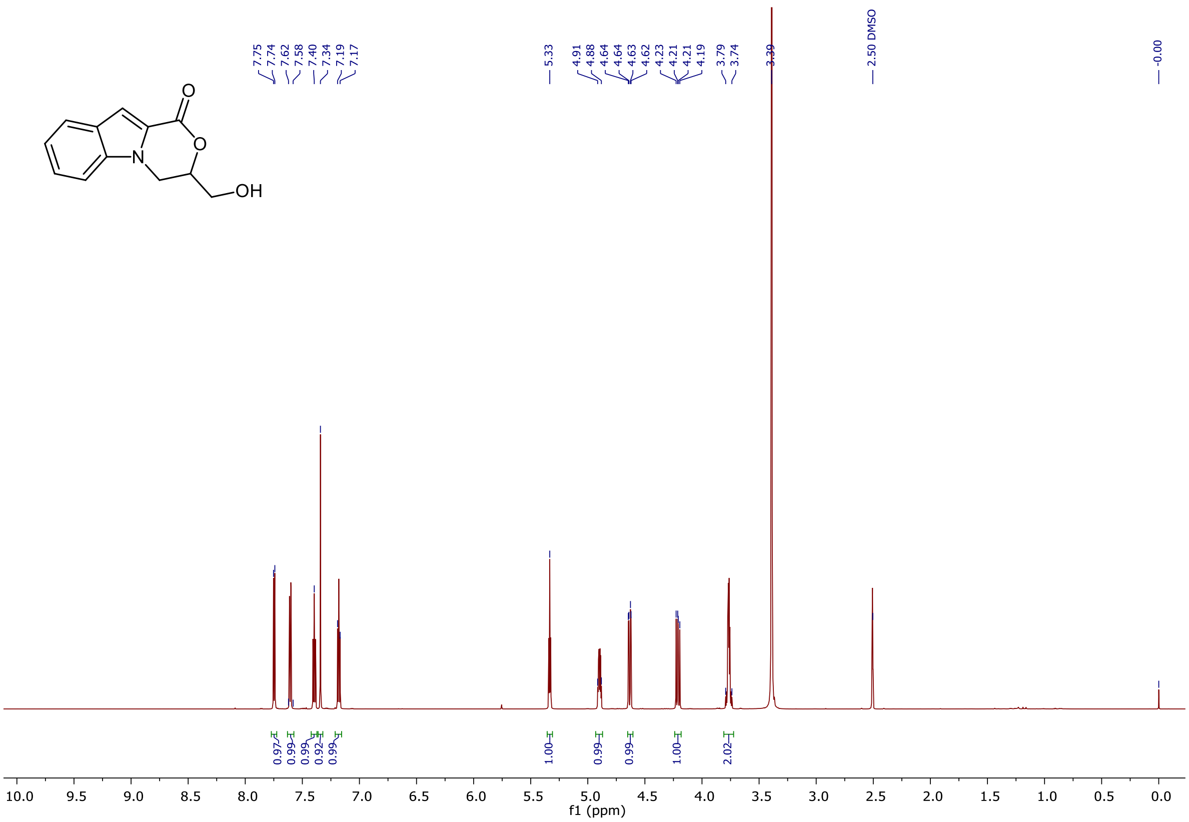

Figure S31. ^{1}H NMR (700 MHz, DMSO-d_6) spectrum of **6a**

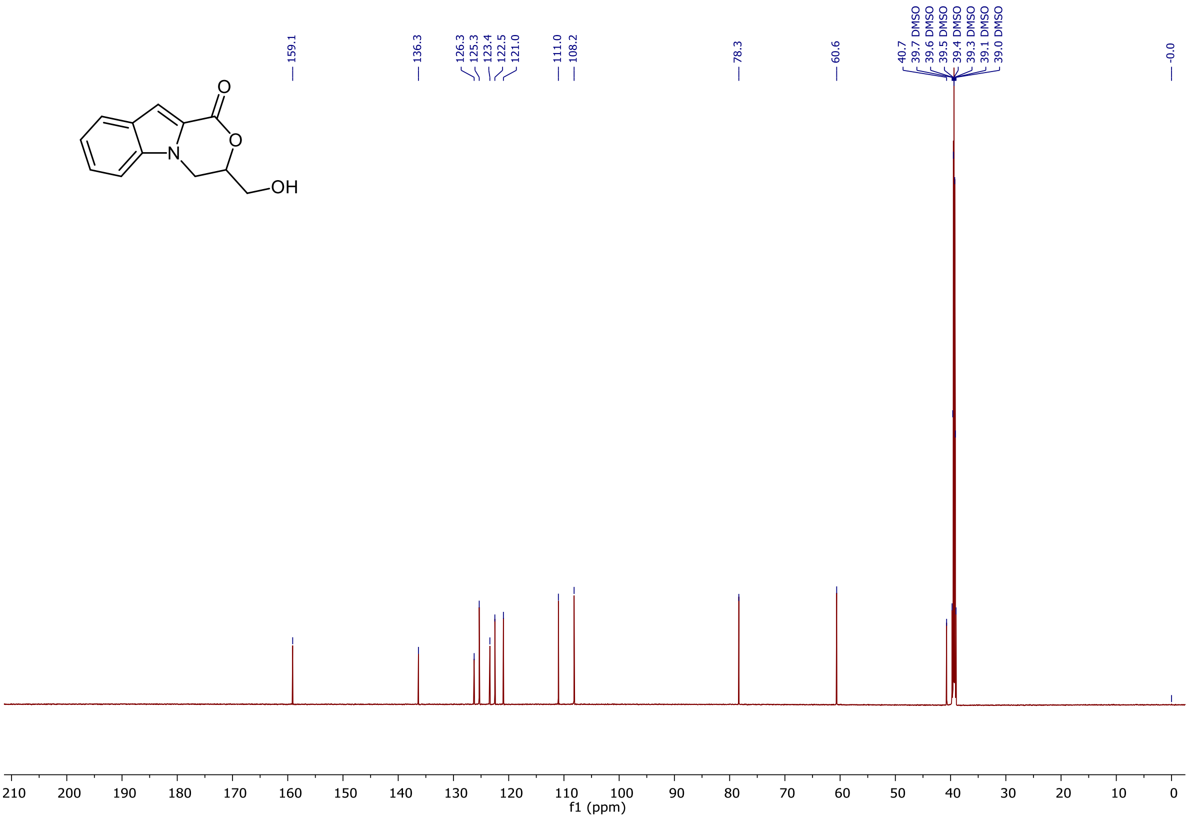

Figure S32. ^{13}C NMR (176 MHz, DMSO-d_6) spectrum of **6a**

Compound Spectrum SmartFormula Report

Analysis Info

Analysis Name	D:\Data\IZ-214.d	Acquisition Date	12/18/2024 9:06:46 AM
Method	DirectInfusion_TuneLow_pos.m	Operator	hplc
Sample Name	IZ-214	Instrument	micrOTOF-Q III 8228888.20448
Comment	SB		

Acquisition Parameter

Source Type	ESI	Ion Polarity	Positive	Set Nebulizer	0.4 Bar
Focus	Not active	Set Capillary	4500 V	Set Dry Heater	180 °C
Scan Begin	50 m/z	Set End Plate Offset	-500 V	Set Dry Gas	4.0 l/min
Scan End	1000 m/z	Set Collision Cell RF	140.0 Vpp	Set Divert Valve	Waste

#	RT [min]	Area	Int. Type	I	S/N	Chromatogram	Max. m/z	FWHM [min]
n.a.	2.7	n.a.	Single spectrum	n.a.	n.a.	n.a.	304.2625	n.a.
n.a.	4.8	n.a.	Single spectrum	n.a.	n.a.	n.a.	240.0631	n.a.

+MS, 4.8min #285

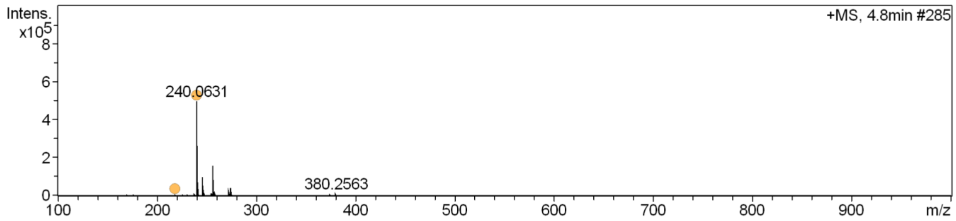

Meas. m/z	#	Ion Formula	m/z	err [ppm]	mSigma	# Sigma	Score	rdb	e⁻ Conf	N-Rule
218.0815	1	C12H12NO3	218.0812	1.7	8.5	1	100.00	7.5	even	ok
240.0631	1	C12H11NNaO3	240.0631	0.0	4.5	1	100.00	7.5	even	ok

Figure S33. HRMS (ESI) report of **6a**

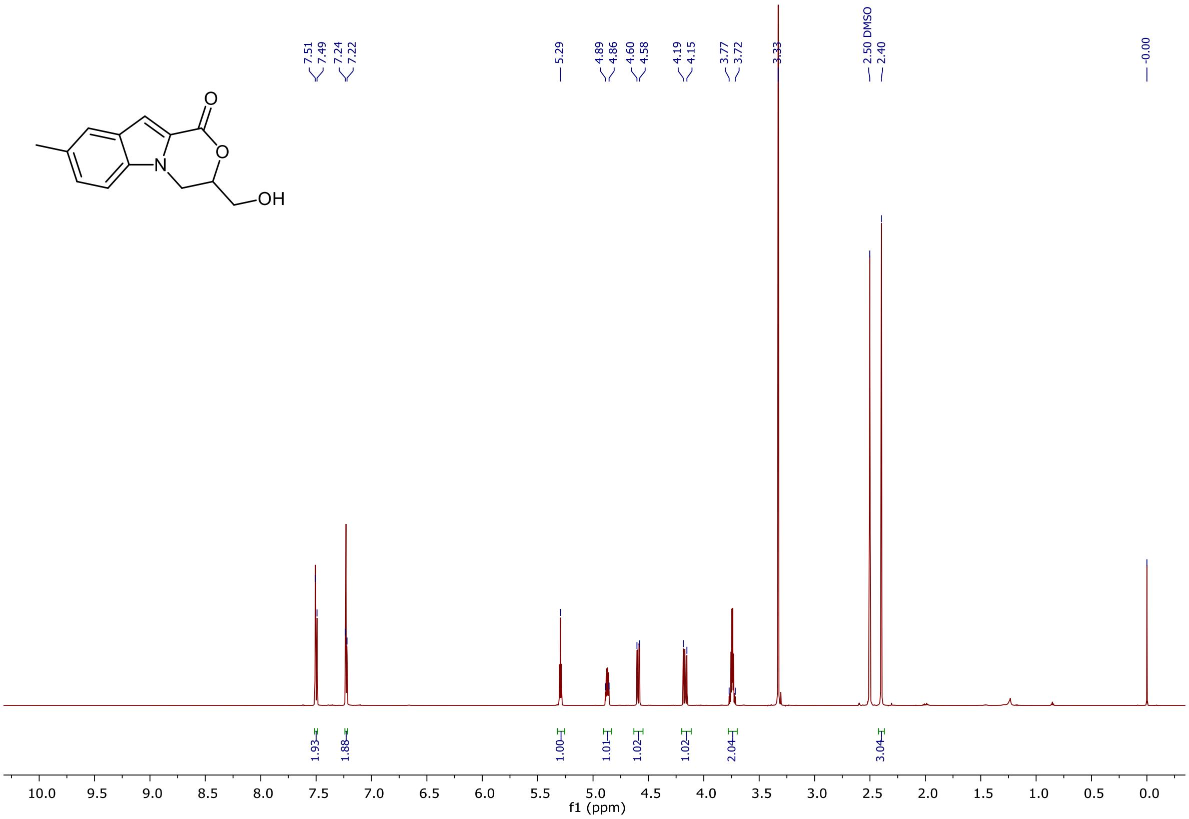

Figure S34. ^{1}H NMR (700 MHz, DMSO-d_6) spectrum of **6b**

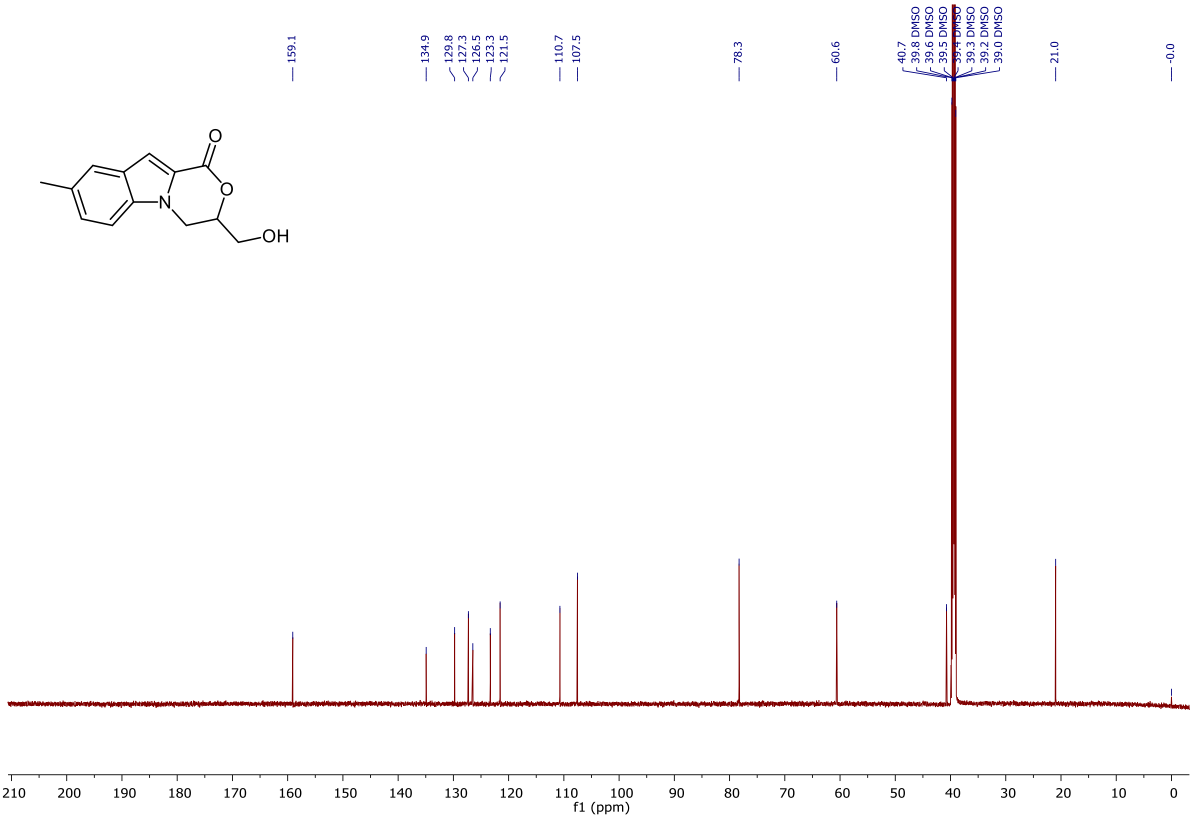

Figure S35. ^{13}C NMR (176 MHz, DMSO-d_6) spectrum of **6b**

Compound Spectrum SmartFormula Report

Analysis Info

Analysis Name	D:\Data\IZ-248.d	Acquisition Date	3/18/2025 2:27:22 PM
Method	DirectInfusion_TuneLow_pos.m	Operator	hplc
Sample Name	IZ-248	Instrument	micrOTOF-Q III 8228888.20448
Comment	AB		

Acquisition Parameter

Source Type	ESI	Ion Polarity	Positive	Set Nebulizer	0.4 Bar
Focus	Not active	Set Capillary	4500 V	Set Dry Heater	180 °C
Scan Begin	50 m/z	Set End Plate Offset	-500 V	Set Dry Gas	4.0 l/min
Scan End	1000 m/z	Set Collision Cell RF	140.0 Vpp	Set Divert Valve	Waste

#	RT [min]	Area	Int. Type	I	S/N	Chromatogram	Max. m/z	FWHM [min]
n.a.	6.5	n.a.	Single spectrum	n.a.	n.a.	n.a.	254.0789	n.a.

+MS, 6.5min #388

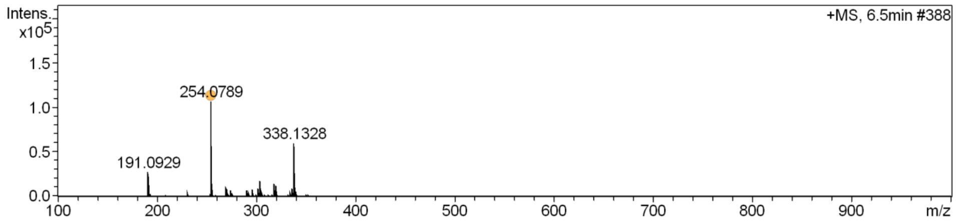

Meas. m/z	#	Ion Formula	m/z	err [ppm]	mSigma	# Sigma	Score	rdb	e^- Conf	N-Rule
254.0789	1	C13H13NNaO3	254.0788	-0.7	9.1	1	100.00	7.5	even	ok

Figure S36. HRMS (ESI) report of **6b**

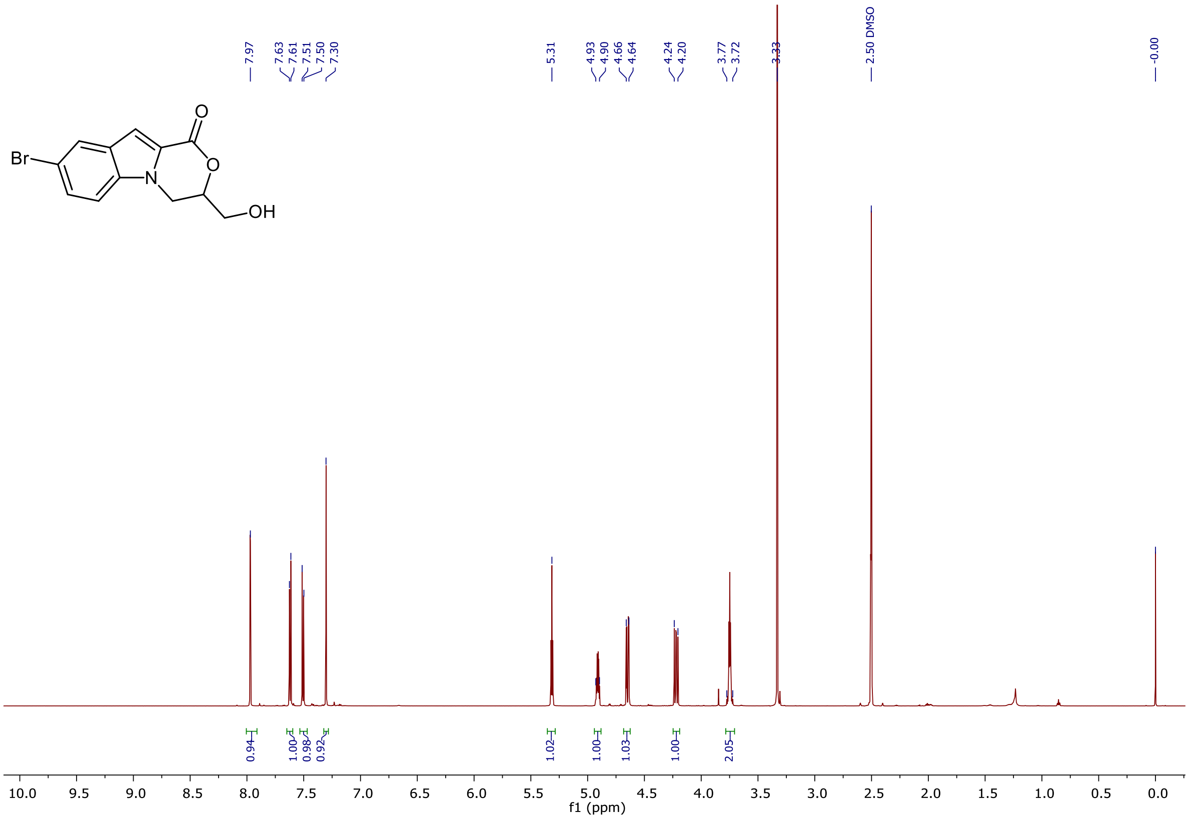

Figure S37. ^{1}H NMR (700 MHz, DMSO-d_6) spectrum of **6c**

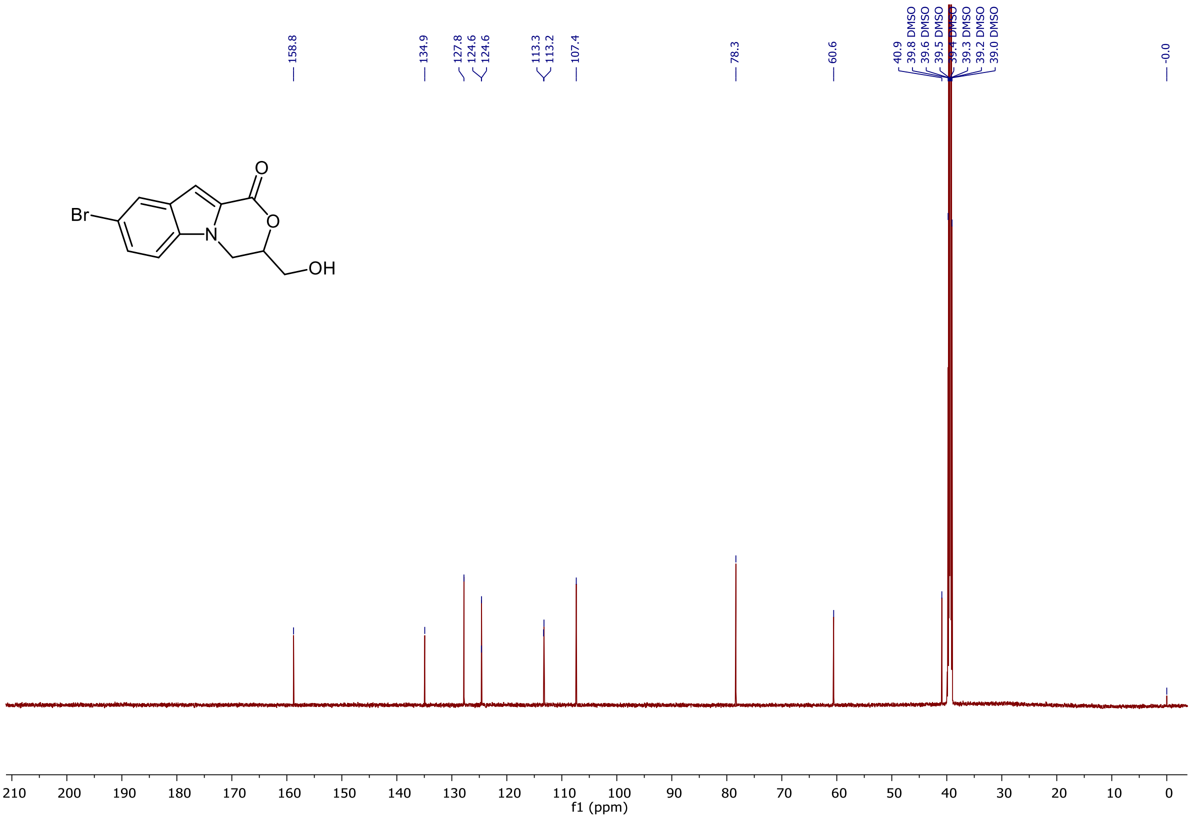

Figure S38. ^{13}C NMR (176 MHz, DMSO-d_6) spectrum of **6c**

Compound Spectrum SmartFormula Report

Analysis Info

Analysis Name	D:\Data\IZ-252.d	Acquisition Date	3/17/2025 6:48:18 PM
Method	DirectInfusion_TuneLow_pos.m	Operator	hplc
Sample Name	IZ-252	Instrument	micrOTOF-Q III 8228888.20448
Comment	AB		

Acquisition Parameter

Source Type	ESI	Ion Polarity	Positive	Set Nebulizer	0.4 Bar
Focus	Not active	Set Capillary	4500 V	Set Dry Heater	180 °C
Scan Begin	50 m/z	Set End Plate Offset	-500 V	Set Dry Gas	4.0 l/min
Scan End	1000 m/z	Set Collision Cell RF	140.0 Vpp	Set Divert Valve	Waste

#	RT [min]	Area	Int. Type	I	S/N	Chromatogram	Max. m/z	FWHM [min]
n.a.	1.9	n.a.	Single spectrum	n.a.	n.a.	n.a.	317.9734	n.a.

+MS, 1.9min #111

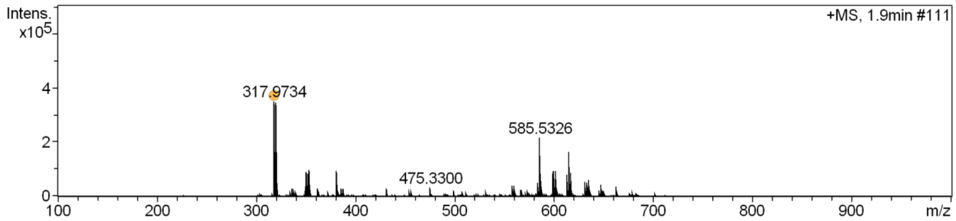

Meas. m/z	#	Ion Formula	m/z	err [ppm]	mSigma	# Sigma	Score	rdb	e⁻ Conf	N-Rule
317.9734	1	C12H10BrNNaO3	317.9736	0.8	5.1	1	100.00	7.5	even	ok

Figure S39. HRMS (ESI) report of **6c**

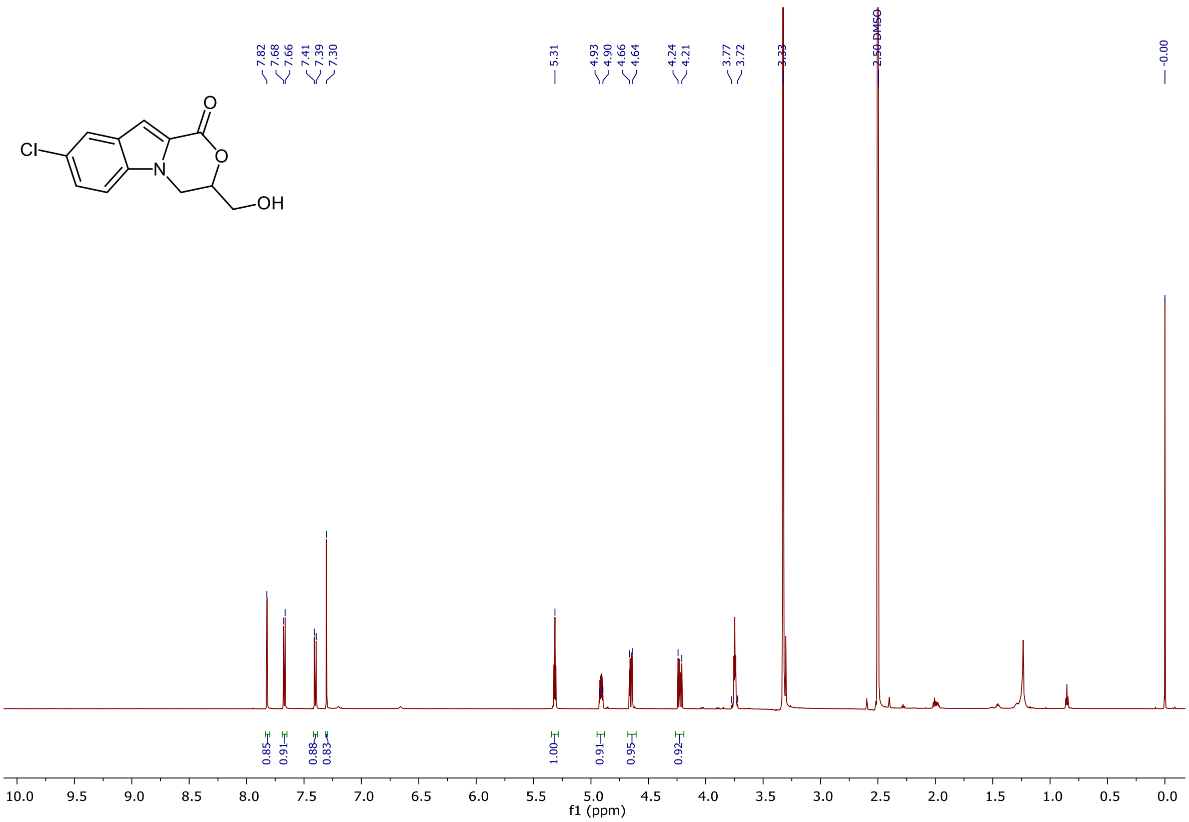

Figure S40. ^{1}H NMR (700 MHz, DMSO-d_6) spectrum of **6d**

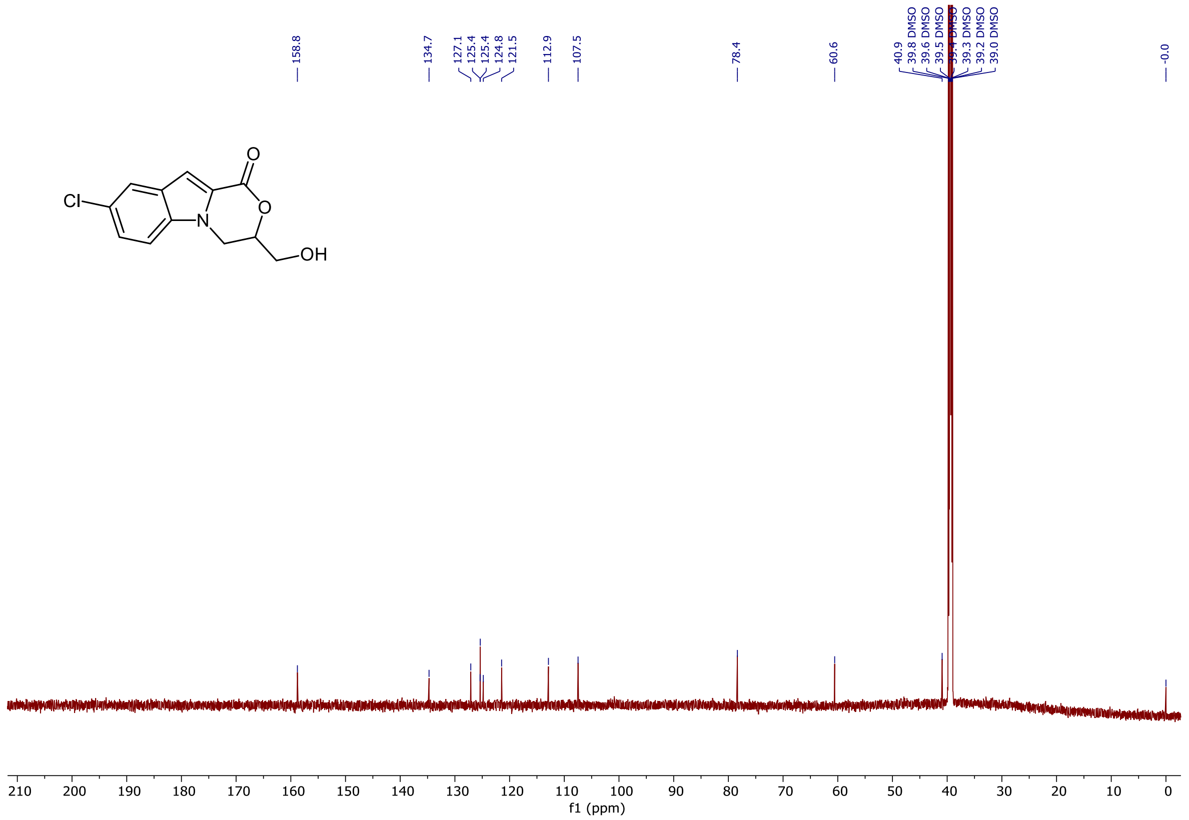

Figure S41. ^{13}C NMR (176 MHz, DMSO-d_6) spectrum of **6d**

Compound Spectrum SmartFormula Report

Analysis Info

Analysis Name	D:\Data\IZ-261.d	Acquisition Date	3/18/2025 3:34:20 PM
Method	DirectInfusion_TuneLow_pos.m	Operator	hplc
Sample Name	IZ-261	Instrument	micrOTOF-Q III 8228888.20448
Comment	AB		

Acquisition Parameter

Source Type	ESI	Ion Polarity	Positive	Set Nebulizer	0.4 Bar
Focus	Not active	Set Capillary	4500 V	Set Dry Heater	180 °C
Scan Begin	50 m/z	Set End Plate Offset	-500 V	Set Dry Gas	4.0 l/min
Scan End	1000 m/z	Set Collision Cell RF	140.0 Vpp	Set Divert Valve	Waste

#	RT [min]	Area	Int. Type	I	S/N	Chromatogram	Max. m/z	FWHM [min]
n.a.	17.6	n.a.	Single spectrum	n.a.	n.a.	n.a.	304.2635	n.a.

+MS, 17.6min #1057

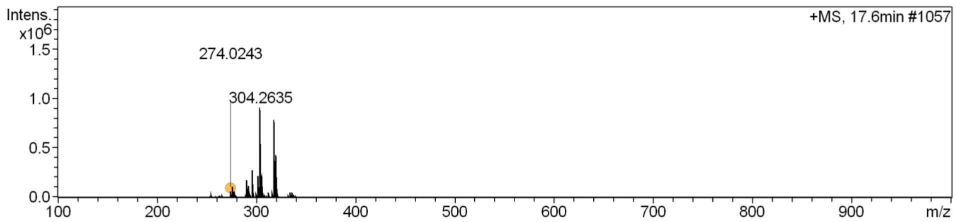

Meas. m/z	#	Ion Formula	m/z	err [ppm]	mSigma	# Sigma	Score	rdb	e⁻ Conf	N-Rule
274.0243	1	C12H10ClNNaO3	274.0241	0.6	22.8	1	100.00	7.5	even	ok

Figure S42. HRMS (ESI) report of **6d**

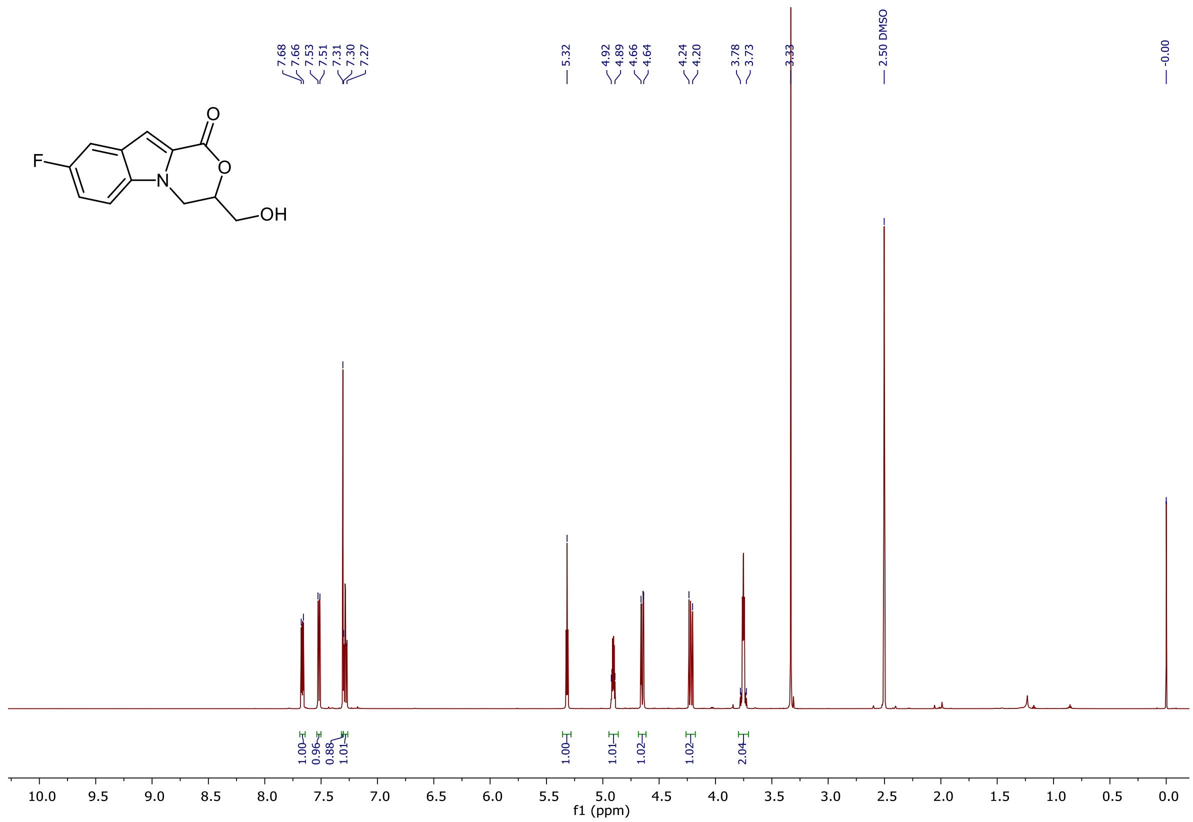

Figure S43. ^{1}H NMR (700 MHz, DMSO-d_6) spectrum of **6e**

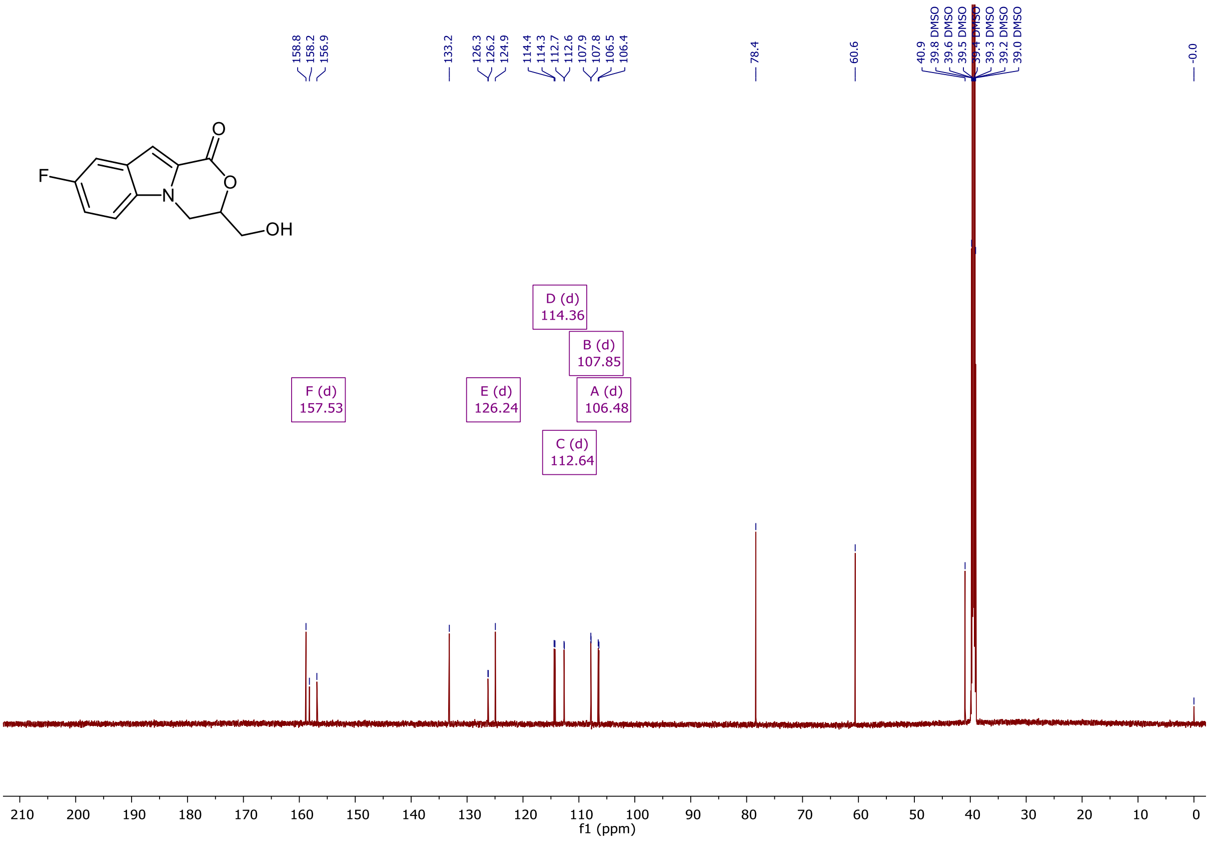

Figure S44. ^{13}C NMR (176 MHz, DMSO-d_6) spectrum of **6e**

Compound Spectrum SmartFormula Report

Analysis Info

Analysis Name	D:\Data\IZ-266.d	Acquisition Date	3/18/2025 1:30:37 PM
Method	DirectInfusion_TuneLow_pos.m	Operator	hplc
Sample Name	IZ-266	Instrument	micrOTOF-Q III 8228888.20448
Comment	AB		

Acquisition Parameter

Source Type	ESI	Ion Polarity	Positive	Set Nebulizer	0.4 Bar
Focus	Not active	Set Capillary	4500 V	Set Dry Heater	180 °C
Scan Begin	50 m/z	Set End Plate Offset	-500 V	Set Dry Gas	4.0 l/min
Scan End	1000 m/z	Set Collision Cell RF	140.0 Vpp	Set Divert Valve	Waste

#	RT [min]	Area	Int. Type	I	S/N	Chromatogram	Max. m/z	FWHM [min]
n.a.	15.1	n.a.	Single spectrum	n.a.	n.a.	n.a.	258.0539	n.a.

+MS, 15.1min #908

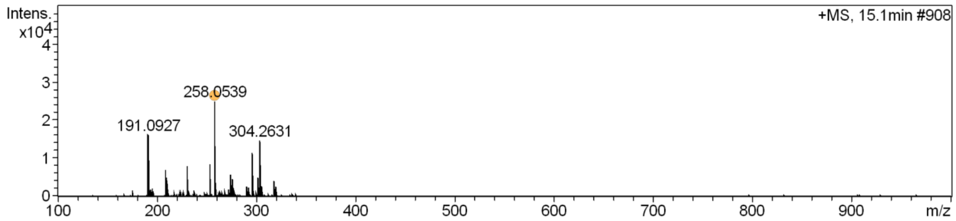

Meas. m/z	#	Ion Formula	m/z	err [ppm]	mSigma	# Sigma	Score	rdb	e⁻ Conf	N-Rule
258.0539	1	C12H10FNNaO3	258.0537	-0.8	0.7	1	100.00	7.5	even	ok

Figure S45. HRMS (ESI) report of **6e**

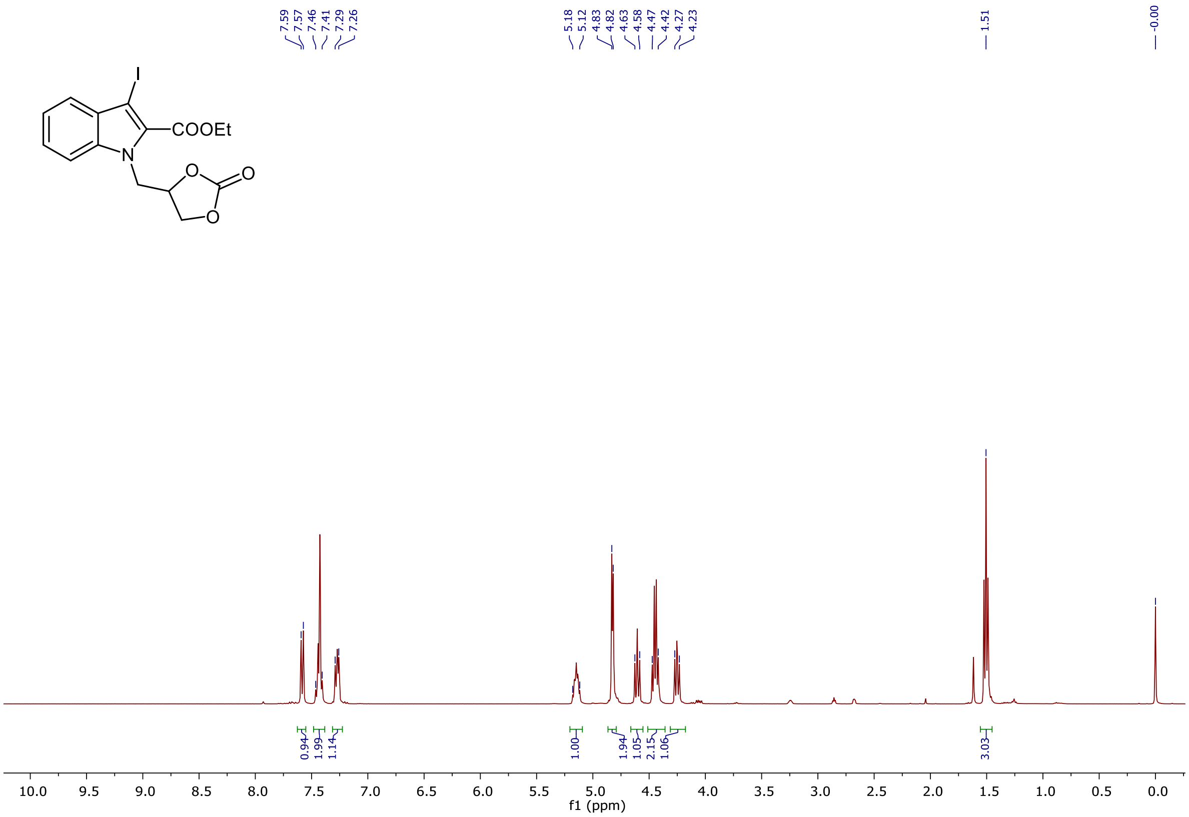

Figure S46. ^{1}H NMR (400 MHz, $CDCl_3$) spectrum of **7a**

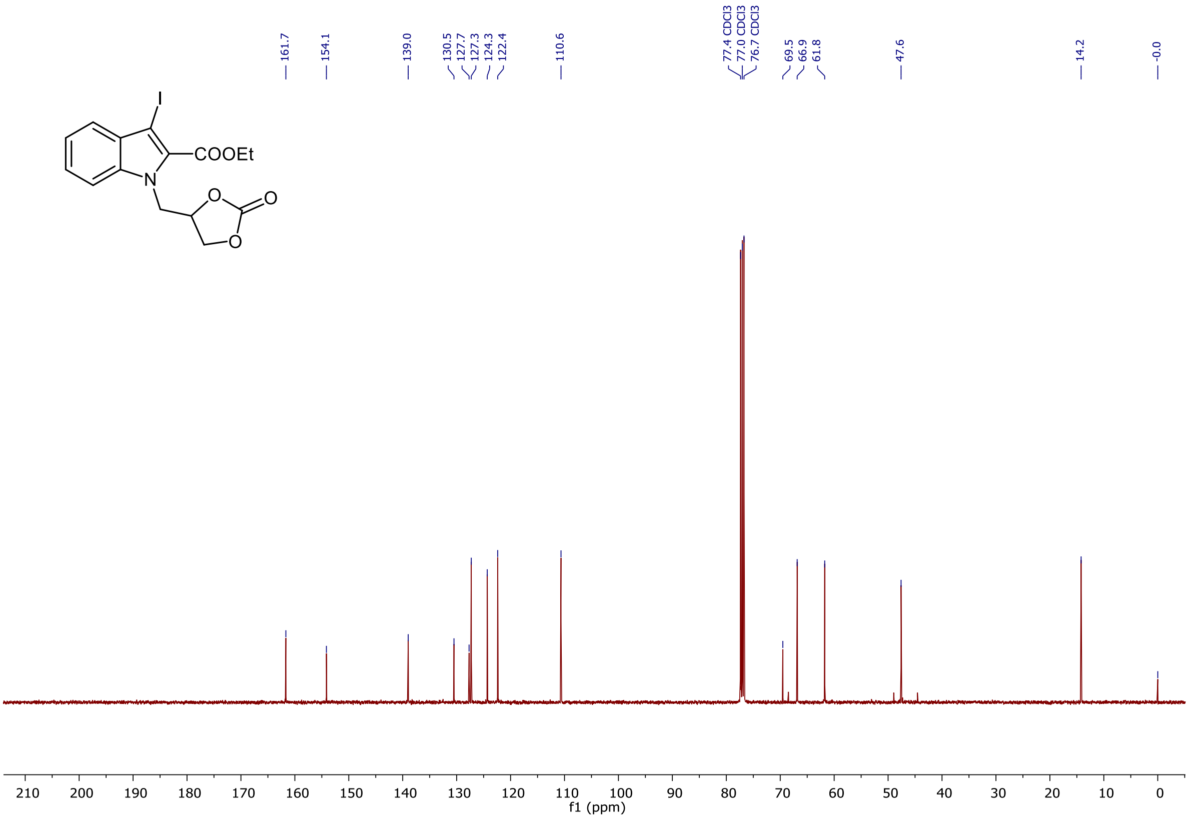

Figure S47. ^{13}C NMR (101 MHz, $CDCl_3$) spectrum of **7a**

Compound Spectrum SmartFormula Report

Analysis Info

Analysis Name	D:\Data\IZ-277.d	Acquisition Date	5/7/2025 1:11:55 AM
Method	DirectInfusion_TuneLow_pos.m	Operator	hplc
Sample Name	IZ-277	Instrument	micrOTOF-Q III 8228888.20448
Comment	AB		

Acquisition Parameter

Source Type	ESI	Ion Polarity	Positive	Set Nebulizer	0.4 Bar
Focus	Not active	Set Capillary	4500 V	Set Dry Heater	180 °C
Scan Begin	50 m/z	Set End Plate Offset	-500 V	Set Dry Gas	4.0 l/min
Scan End	1000 m/z	Set Collision Cell RF	140.0 Vpp	Set Divert Valve	Waste

#	RT [min]	Area	Int. Type	I	S/N	Chromatogram	Max. m/z	FWHM [min]
n.a.	5.7	n.a.	Single spectrum	n.a.	n.a.	n.a.	437.9805	n.a.

+MS, 5.7min #340

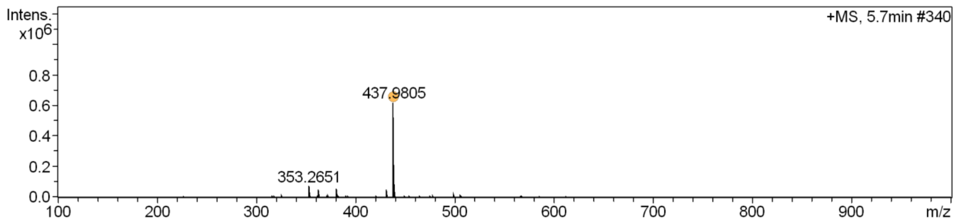

Meas. m/z	#	Ion Formula	m/z	err [ppm]	mSigma	# Sigma	Score	rdb	e^- Conf	N-Rule
437.9805	1	C15H14INNaO5	437.9809	-0.9	4.5	1	100.00	8.5	even	ok

Figure S48. HRMS (ESI) report of **7a**

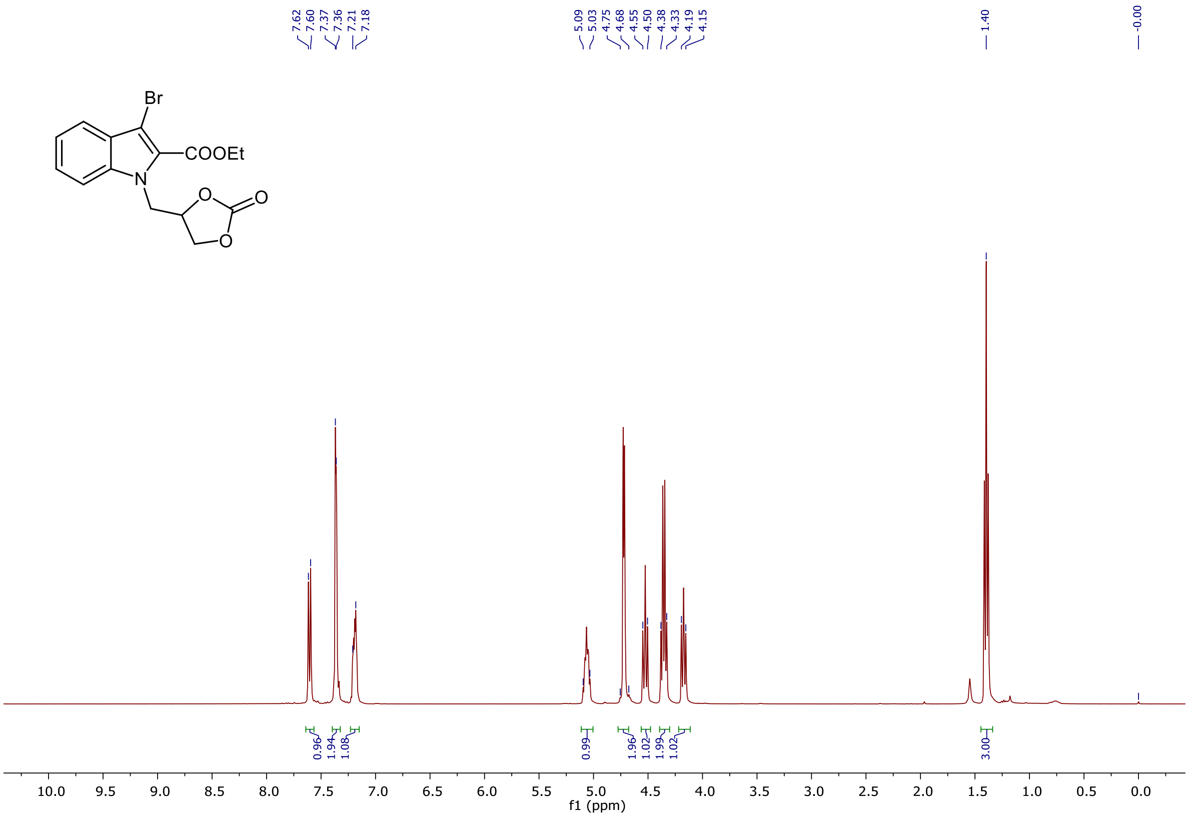

Figure S49. ^{1}H NMR (400 MHz, $CDCl_3$) spectrum of **7b**

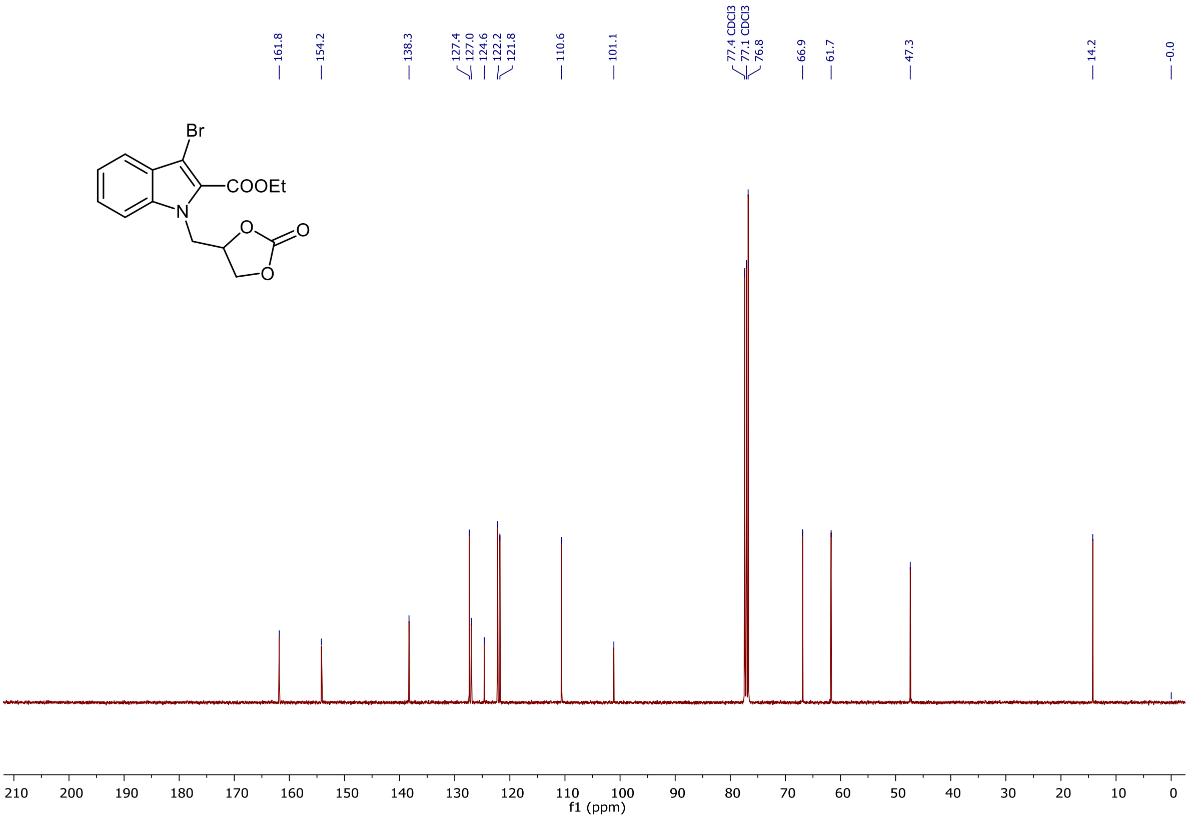

Figure S50. ^{13}C NMR (101 MHz, $CDCl_3$) spectrum of **7b**

Compound Spectrum SmartFormula Report

Analysis Info

Analysis Name	D:\Data\IZ-313.d	Acquisition Date	5/7/2025 4:12:07 AM
Method	DirectInfusion_TuneLow_pos.m	Operator	hplc
Sample Name	IZ-313	Instrument	micrOTOF-Q III 8228888.20448
Comment	AB		

Acquisition Parameter

Source Type	ESI	Ion Polarity	Positive	Set Nebulizer	0.4 Bar
Focus	Not active	Set Capillary	4500 V	Set Dry Heater	180 °C
Scan Begin	50 m/z	Set End Plate Offset	-500 V	Set Dry Gas	4.0 l/min
Scan End	1000 m/z	Set Collision Cell RF	140.0 Vpp	Set Divert Valve	Waste

#	RT [min]	Area	Int. Type	I	S/N	Chromatogram	Max. m/z	FWHM [min]
n.a.	5.1	n.a.	Single spectrum	n.a.	n.a.	n.a.	389.9945	n.a.

+MS, 5.1min #303

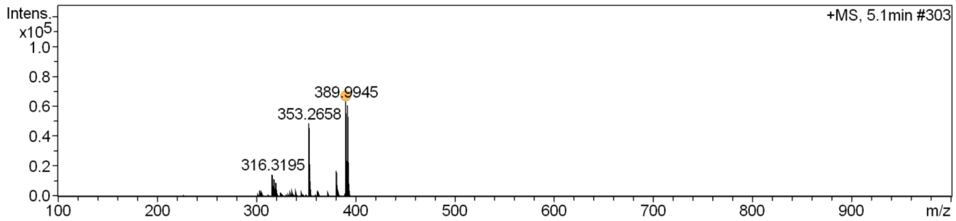

Meas. m/z	#	Ion Formula	m/z	err [ppm]	mSigma	# Sigma	Score	rdb	e⁻ Conf	N-Rule
389.9945	1	C15H14BrNNaO5	389.9948	0.7	21.1	2	100.00	8.5	even	ok

Figure S51. HRMS (ESI) report of **7b**

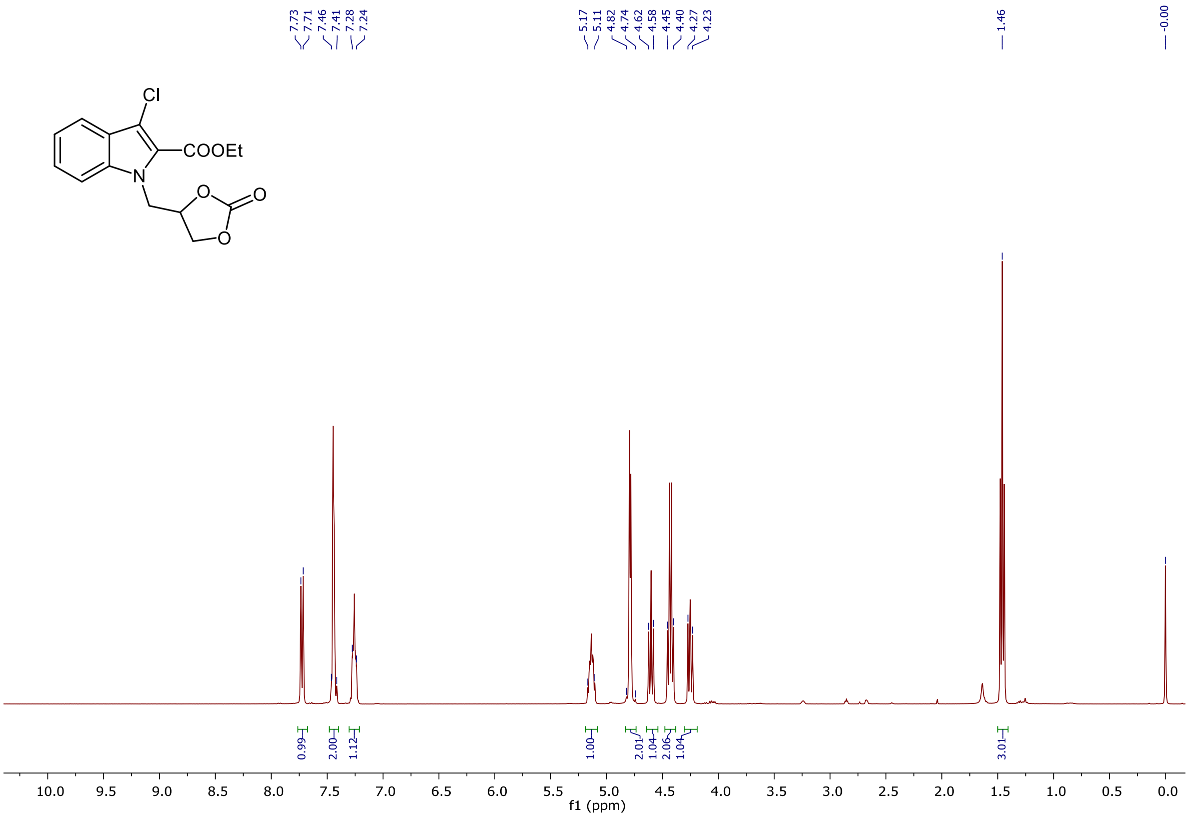

Figure S52. ^{1}H NMR (400 MHz, $CDCl_3$) spectrum of **7c**

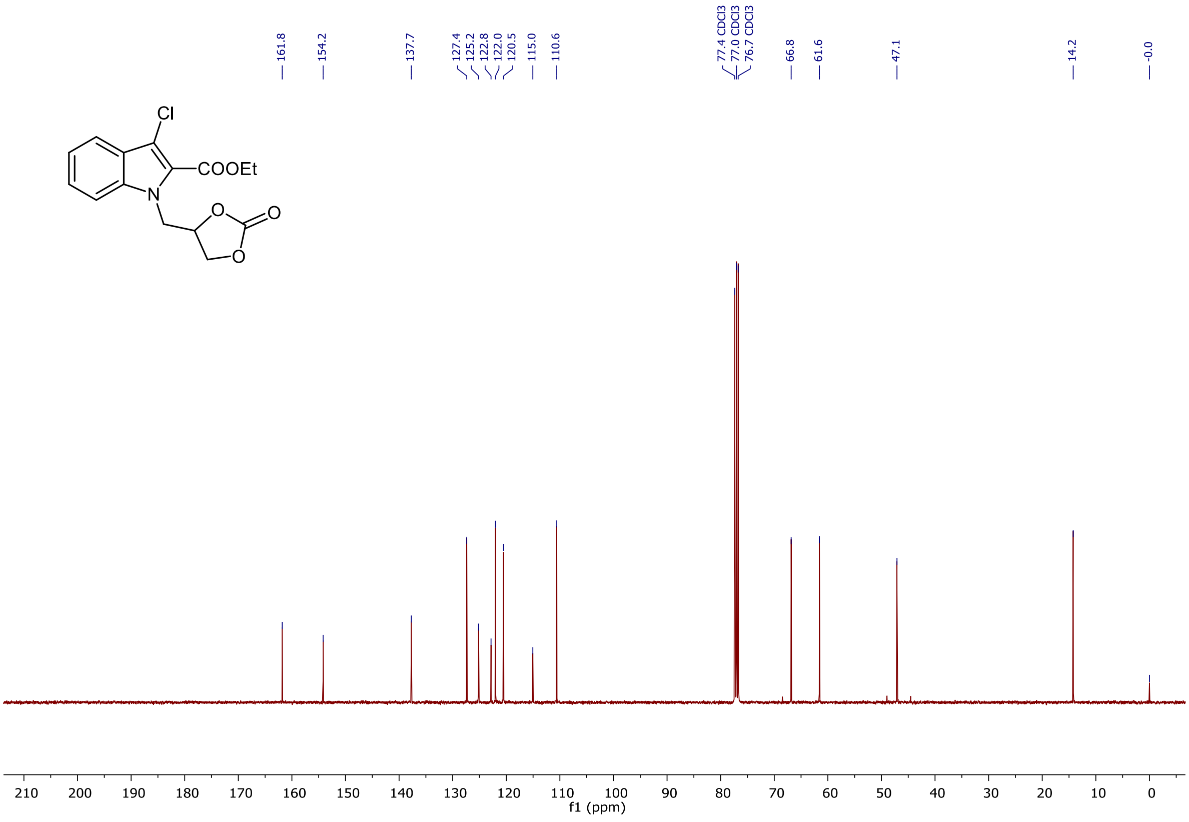

Figure S53. ^{13}C NMR (101 MHz, $CDCl_3$) spectrum of **7c**

Compound Spectrum SmartFormula Report

Analysis Info

Analysis Name	D:\Data\IZ-314.d	Acquisition Date	3/17/2025 7:19:05 PM
Method	DirectInfusion_TuneLow_pos.m	Operator	hplc
Sample Name	IZ-314	Instrument	micrOTOF-Q III 8228888.20448
Comment	AB		

Acquisition Parameter

Source Type	ESI	Ion Polarity	Positive	Set Nebulizer	0.4 Bar
Focus	Not active	Set Capillary	4500 V	Set Dry Heater	180 °C
Scan Begin	50 m/z	Set End Plate Offset	-500 V	Set Dry Gas	4.0 l/min
Scan End	1000 m/z	Set Collision Cell RF	140.0 Vpp	Set Divert Valve	Waste

#	RT [min]	Area	Int. Type	I	S/N	Chromatogram	Max. m/z	FWHM [min]
n.a.	7.0	n.a.	Single spectrum	n.a.	n.a.	n.a.	346.0458	n.a.

+MS, 7.0min #417

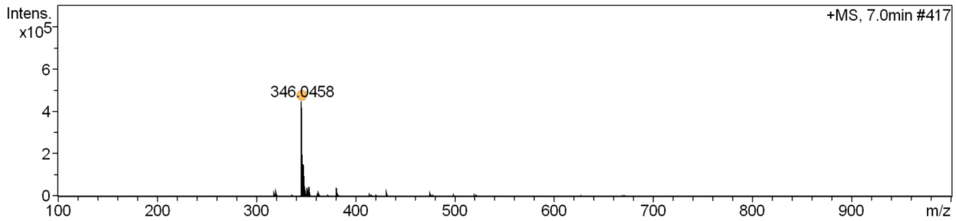

Meas. m/z	#	Ion Formula	m/z	err [ppm]	mSigma	# Sigma	Score	rdb	e⁻ Conf	N-Rule
346.0458	1	C15H14ClNNaO5	346.0453	-1.4	2.6	1	100.00	8.5	even	ok

Figure S54. HRMS (ESI) report of **7c**

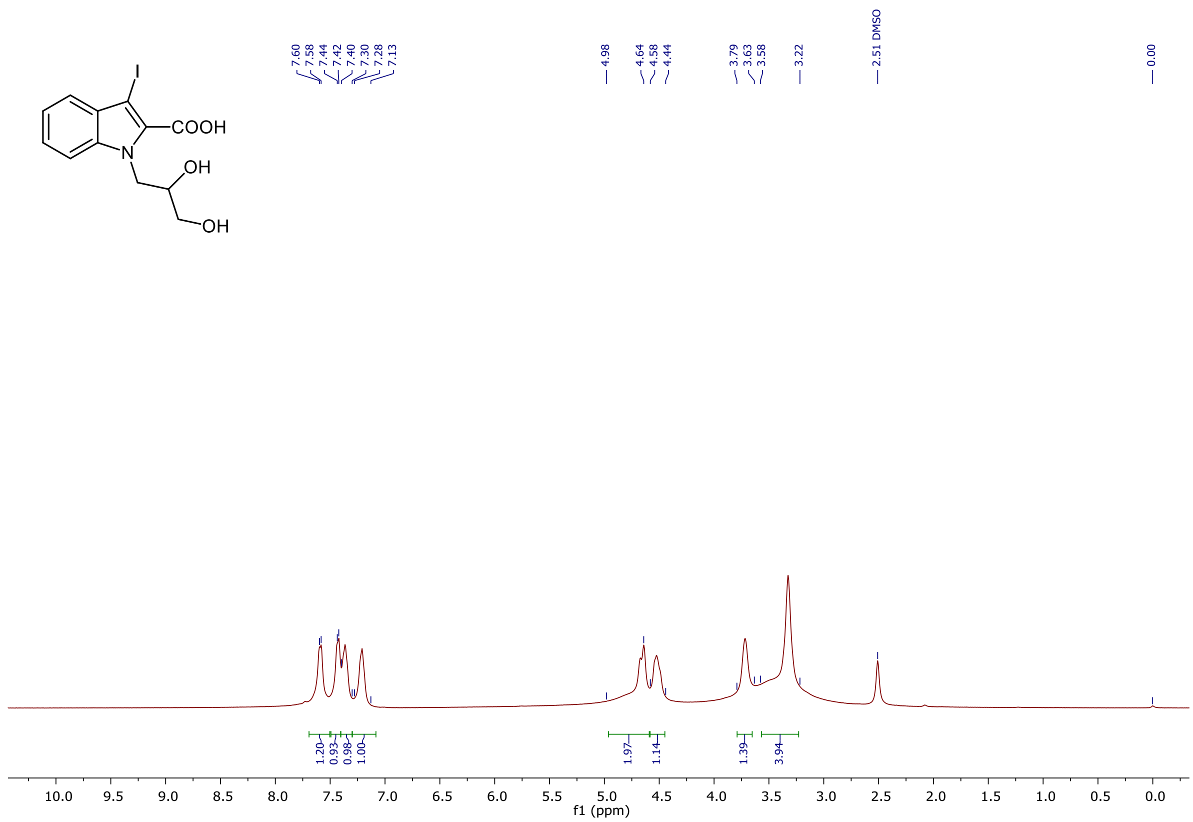

Figure S55. ^{1}H NMR (400 MHz, DMSO-d_6) spectrum of **8a**

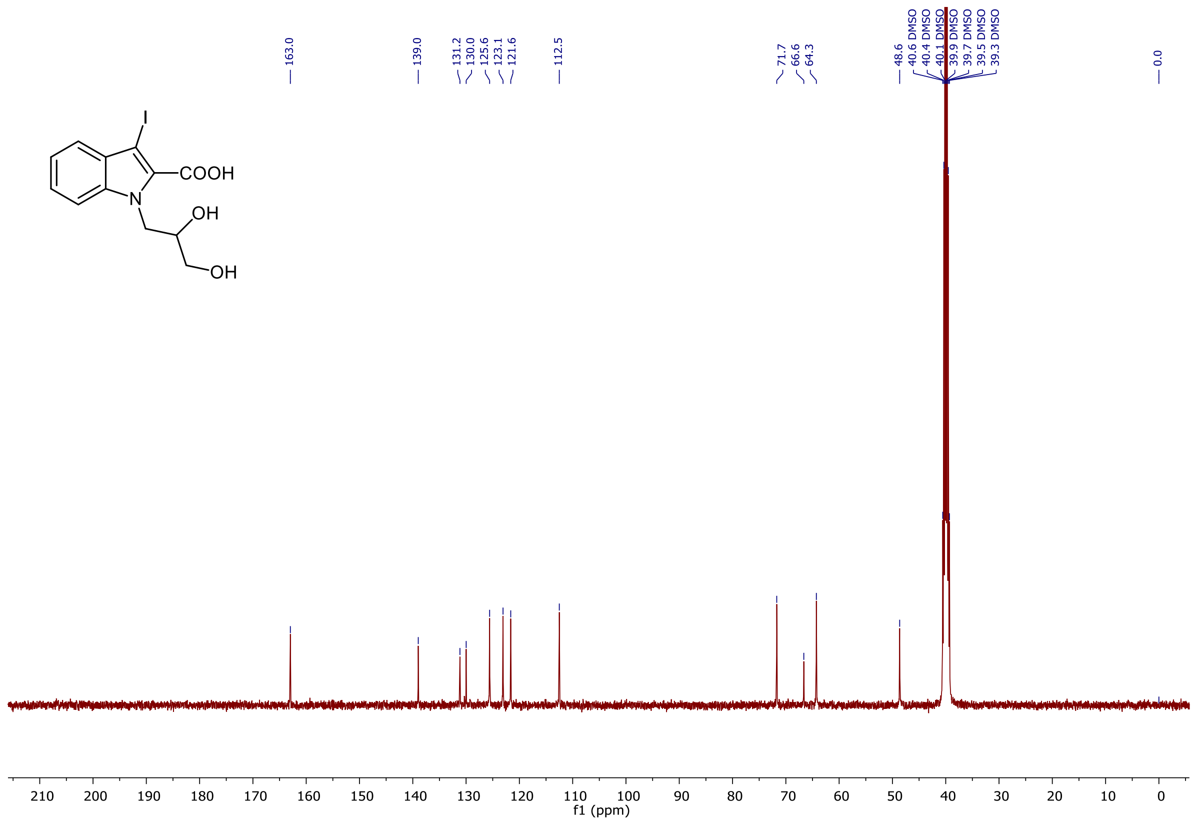

Figure S56. ^{13}C NMR (101 MHz, DMSO-d_6) spectrum of **8a**

Compound Spectrum SmartFormula Report

Analysis Info

Analysis Name	D:\Data\IZ-315.d	Acquisition Date	12/18/2024 10:02:46 AM
Method	DirectInfusion_TuneLow_pos.m	Operator	hplc
Sample Name	IZ-315	Instrument	micrOTOF-Q III 8228888.20448
Comment	SB		

Acquisition Parameter

Source Type	ESI	Ion Polarity	Positive	Set Nebulizer	0.4 Bar
Focus	Not active	Set Capillary	4500 V	Set Dry Heater	180 °C
Scan Begin	50 m/z	Set End Plate Offset	-500 V	Set Dry Gas	4.0 l/min
Scan End	1000 m/z	Set Collision Cell RF	140.0 Vpp	Set Divert Valve	Waste

#	RT [min]	Area	Int. Type	I	S/N	Chromatogram	Max. m/z	FWHM [min]
n.a.	0.0	n.a.	Single spectrum	n.a.	n.a.	n.a.	304.2630	n.a.
n.a.	2.6	n.a.	Single spectrum	n.a.	n.a.	n.a.	383.9703	n.a.

+MS, 2.6min #158

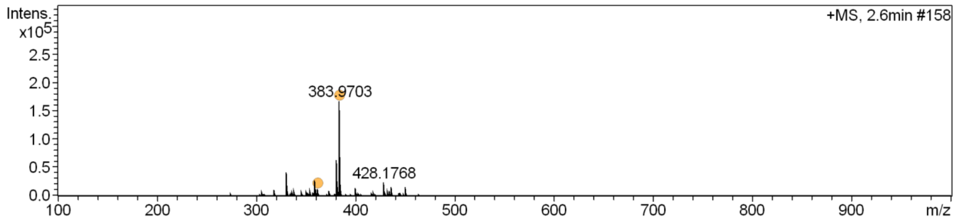

Meas. m/z	#	Ion Formula	m/z	err [ppm]	mSigma	# Sigma	Score	rdb	e⁻ Conf	N-Rule
361.9891	1	C12H13INO4	361.9884	1.9	88.1	1	100.00	6.5	even	ok
383.9703	1	C12H12INNaO4	383.9703	0.1	2.7	1	100.00	6.5	even	ok

Figure S57. HRMS (ESI) report of **8a**

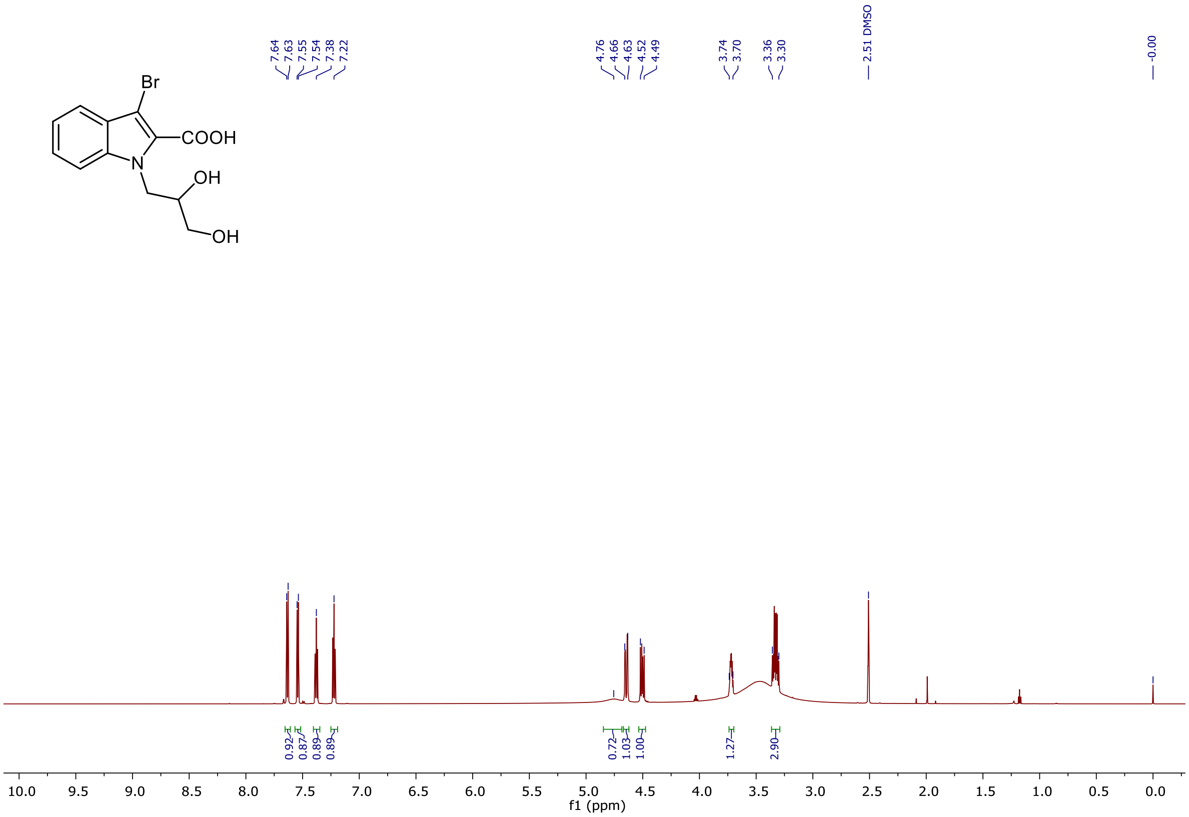

Figure S58. ^{1}H NMR (700 MHz, DMSO-d_6) spectrum of **8b**

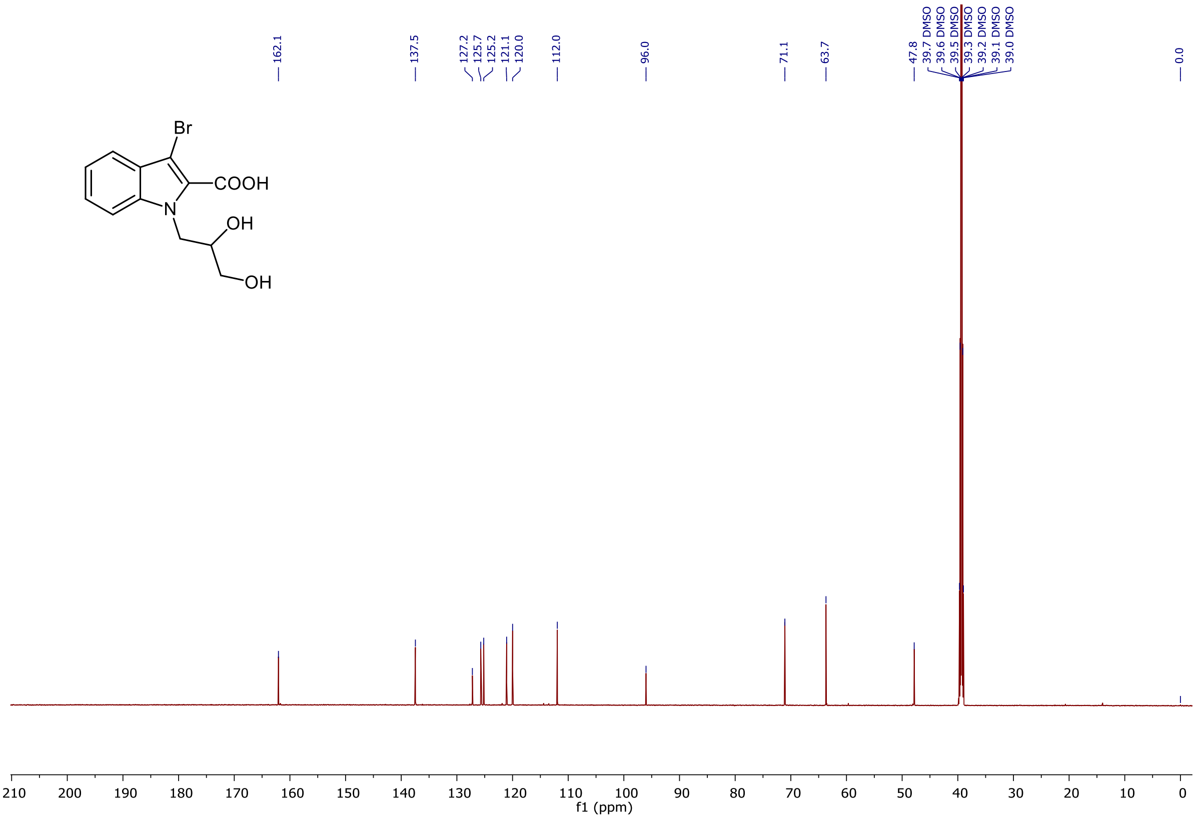

Figure S59. ^{13}C NMR (176 MHz, DMSO-d_6) spectrum of **8b**

Compound Spectrum SmartFormula Report

Analysis Info

Analysis Name	D:\Data\IZ-316.d	Acquisition Date	12/18/2024 10:21:36 AM
Method	DirectInfusion_TuneLow_pos.m	Operator	hplc
Sample Name	IZ-316	Instrument	micrOTOF-Q III 8228888.20448
Comment	SB		

Acquisition Parameter

Source Type	ESI	Ion Polarity	Positive	Set Nebulizer	0.4 Bar
Focus	Not active	Set Capillary	4500 V	Set Dry Heater	180 °C
Scan Begin	50 m/z	Set End Plate Offset	-500 V	Set Dry Gas	4.0 l/min
Scan End	1000 m/z	Set Collision Cell RF	140.0 Vpp	Set Divert Valve	Waste

#	RT [min]	Area	Int. Type	I	S/N	Chromatogram	Max. m/z	FWHM [min]
n.a.	0.1	n.a.	Single spectrum	n.a.	n.a.	n.a.	304.2626	n.a.
n.a.	10.1	n.a.	Single spectrum	n.a.	n.a.	n.a.	335.9842	n.a.

+MS, 10.1min #606

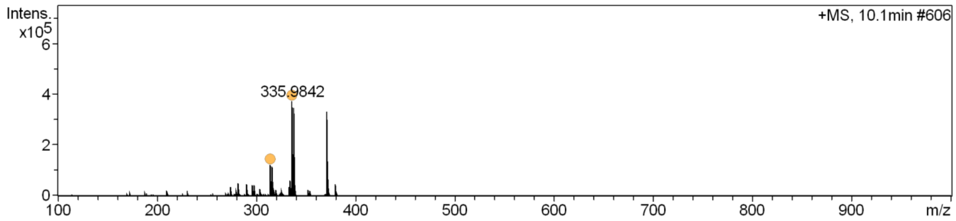

Meas. m/z	#	Ion Formula	m/z	err [ppm]	mSigma	# Sigma	Score	rdb	e⁻ Conf	N-Rule
314.0016	1	C12H13BrNO4	314.0022	1.9	18.3	1	100.00	6.5	even	ok
	1	C12H13BrNO4	314.0022	1.9	18.3	1	100.00	6.5	even	ok
335.9842	1	C12H12BrNNaO4	335.9842	0.0	29.1	1	100.00	6.5	even	ok

Figure S60. HRMS (ESI) report of **8b**

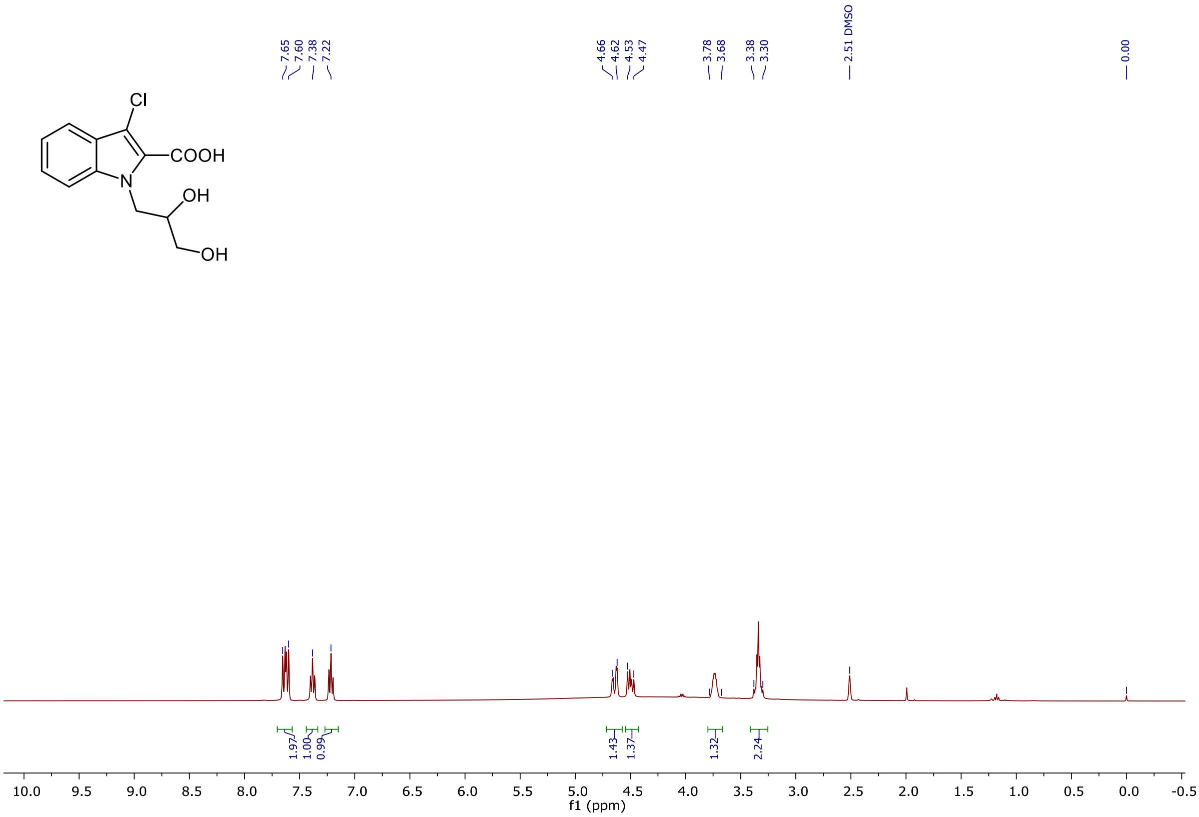

Figure S61. ^{1}H NMR (400 MHz, DMSO-d_6) spectrum of **8c**

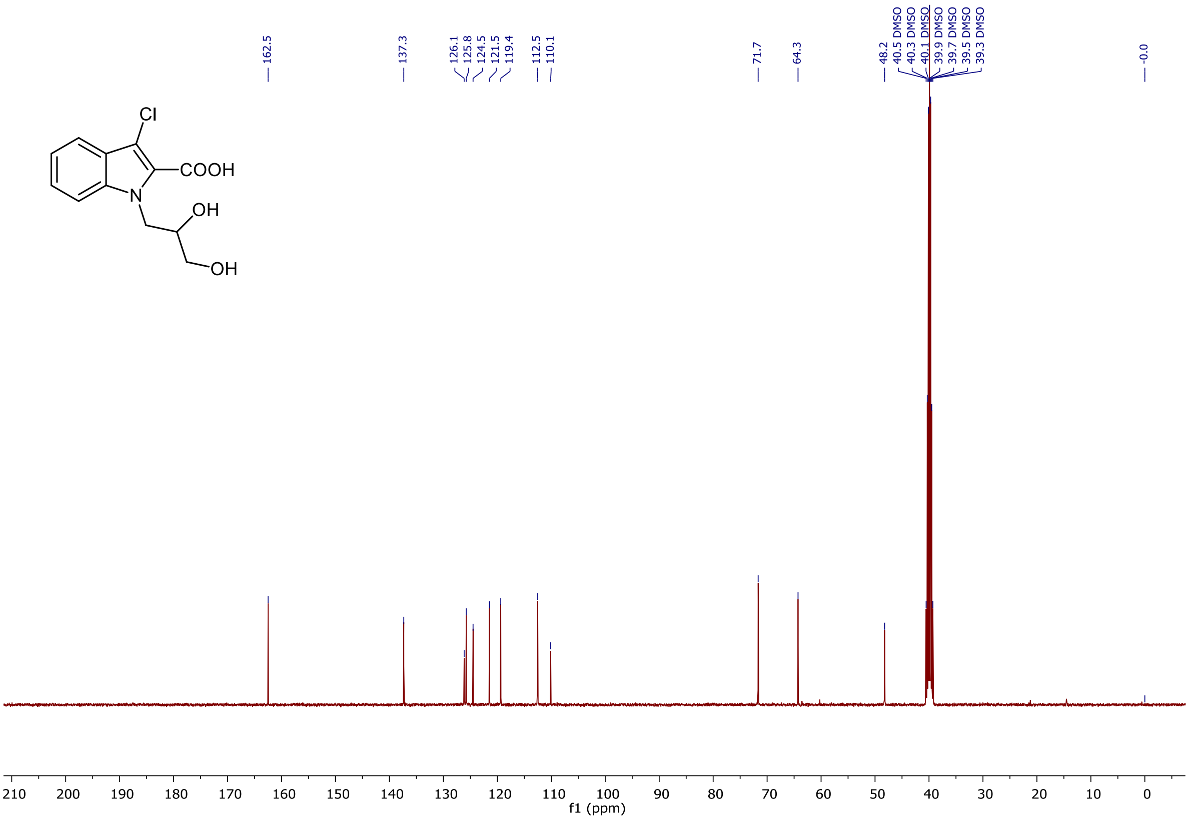

Figure S62. ^{13}C NMR (101 MHz, DMSO-d_6) spectrum of **8c**

Compound Spectrum SmartFormula Report

Analysis Info

Analysis Name	D:\Data\IZ-319.d	Acquisition Date	5/7/2025 4:24:13 AM
Method	DirectInfusion_TuneLow_pos.m	Operator	hplc
Sample Name	IZ-319	Instrument	micrOTOF-Q III 8228888.20448
Comment	AB		

Acquisition Parameter

Source Type	ESI	Ion Polarity	Positive	Set Nebulizer	0.4 Bar
Focus	Not active	Set Capillary	4500 V	Set Dry Heater	180 °C
Scan Begin	50 m/z	Set End Plate Offset	-500 V	Set Dry Gas	4.0 l/min
Scan End	1000 m/z	Set Collision Cell RF	140.0 Vpp	Set Divert Valve	Waste

#	RT [min]	Area	Int. Type	I	S/N	Chromatogram	Max. m/z	FWHM [min]
n.a.	6.9	n.a.	Single spectrum	n.a.	n.a.	n.a.	292.0345	n.a.

+MS, 6.9min #411

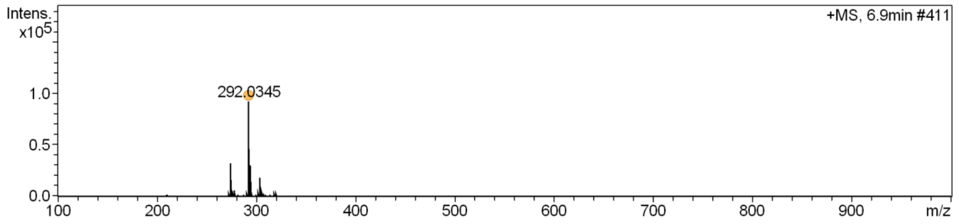

Meas. m/z	#	Ion Formula	m/z	err [ppm]	mSigma	# Sigma	Score	rdb	e⁻ Conf	N-Rule
292.0345	1	C12H12ClNNaO4	292.0347	0.7	4.5	1	100.00	6.5	even	ok

Figure S63. HRMS (ESI) report of **8c**

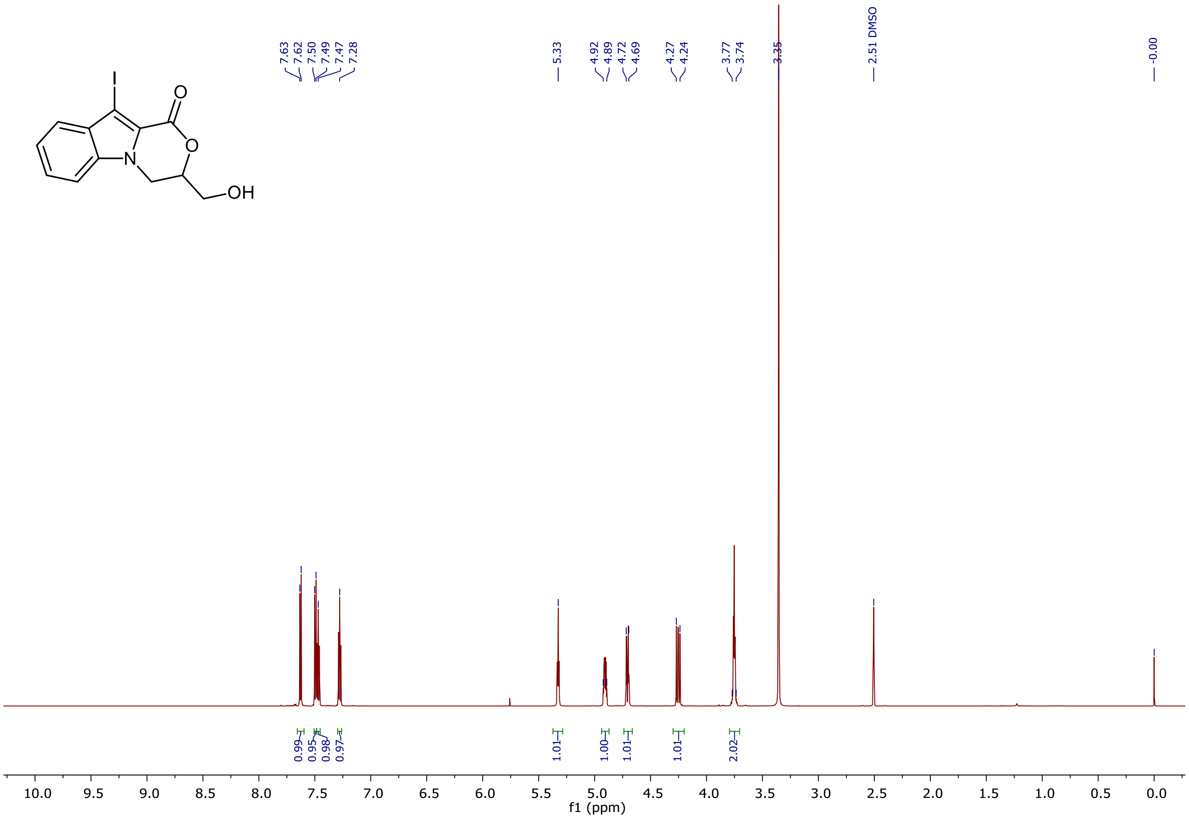

Figure S64. ^{1}H NMR (700 MHz, DMSO-d_6) spectrum of **9a**

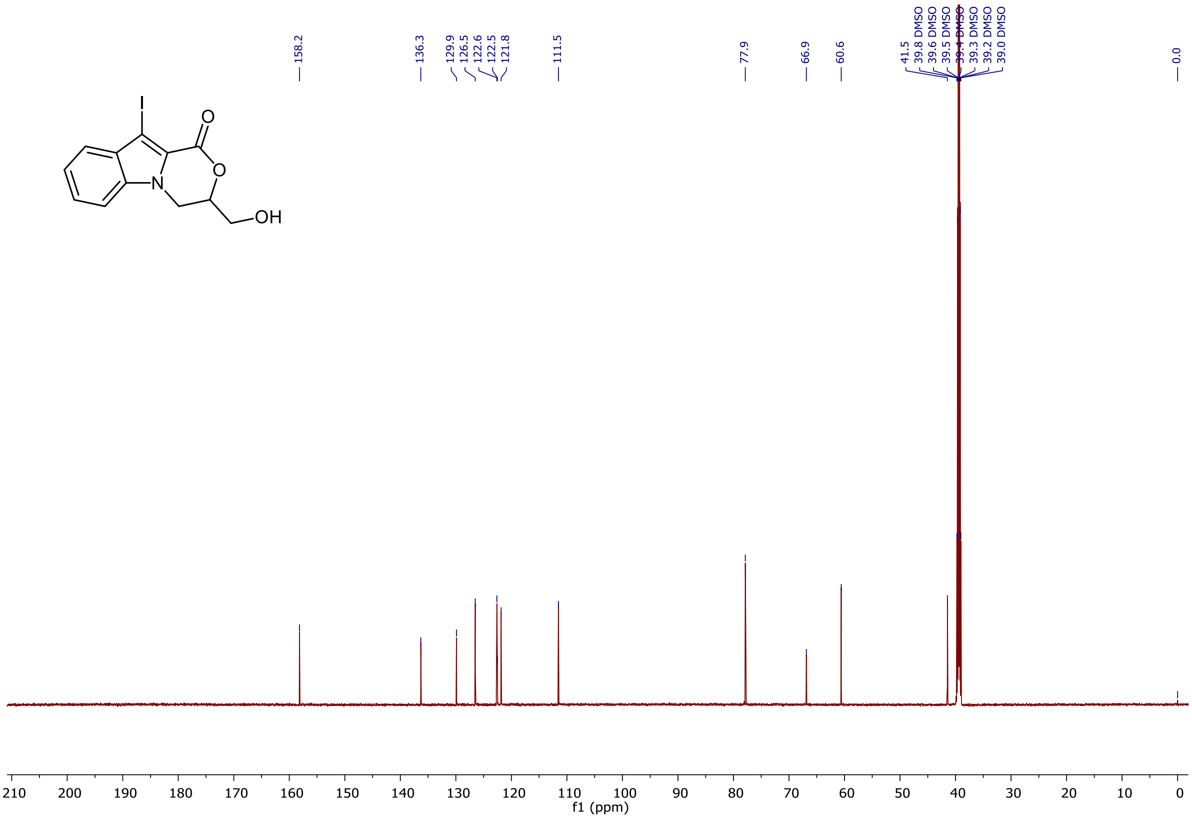

Figure S65. ^{13}C NMR (176 MHz, DMSO-d_6) spectrum of **9a**

Compound Spectrum SmartFormula Report

Analysis Info

Analysis Name	D:\Data\IZ-317.d	Acquisition Date	5/7/2025 3:15:55 AM
Method	DirectInfusion_TuneLow_pos.m	Operator	hplc
Sample Name	IZ-317	Instrument	micrOTOF-Q III 8228888.20448
Comment	AB		

Acquisition Parameter

Source Type	ESI	Ion Polarity	Positive	Set Nebulizer	0.4 Bar
Focus	Not active	Set Capillary	4500 V	Set Dry Heater	180 °C
Scan Begin	50 m/z	Set End Plate Offset	-500 V	Set Dry Gas	4.0 l/min
Scan End	1000 m/z	Set Collision Cell RF	140.0 Vpp	Set Divert Valve	Waste

#	RT [min]	Area	Int. Type	I	S/N	Chromatogram	Max. m/z	FWHM [min]
n.a.	3.6	n.a.	Single spectrum	n.a.	n.a.	n.a.	365.9598	n.a.

+MS, 3.6min #217

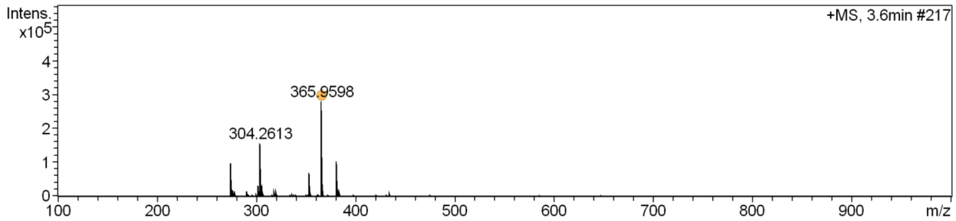

Meas. m/z	#	Ion Formula	m/z	err [ppm]	mSigma	# Sigma	Score	rdb	e⁻ Conf	N-Rule
365.9598	1	C12H10INNaO3	365.9598	0.0	2.2	1	100.00	7.5	even	ok

Figure S66. HRMS (ESI) report of **9a**

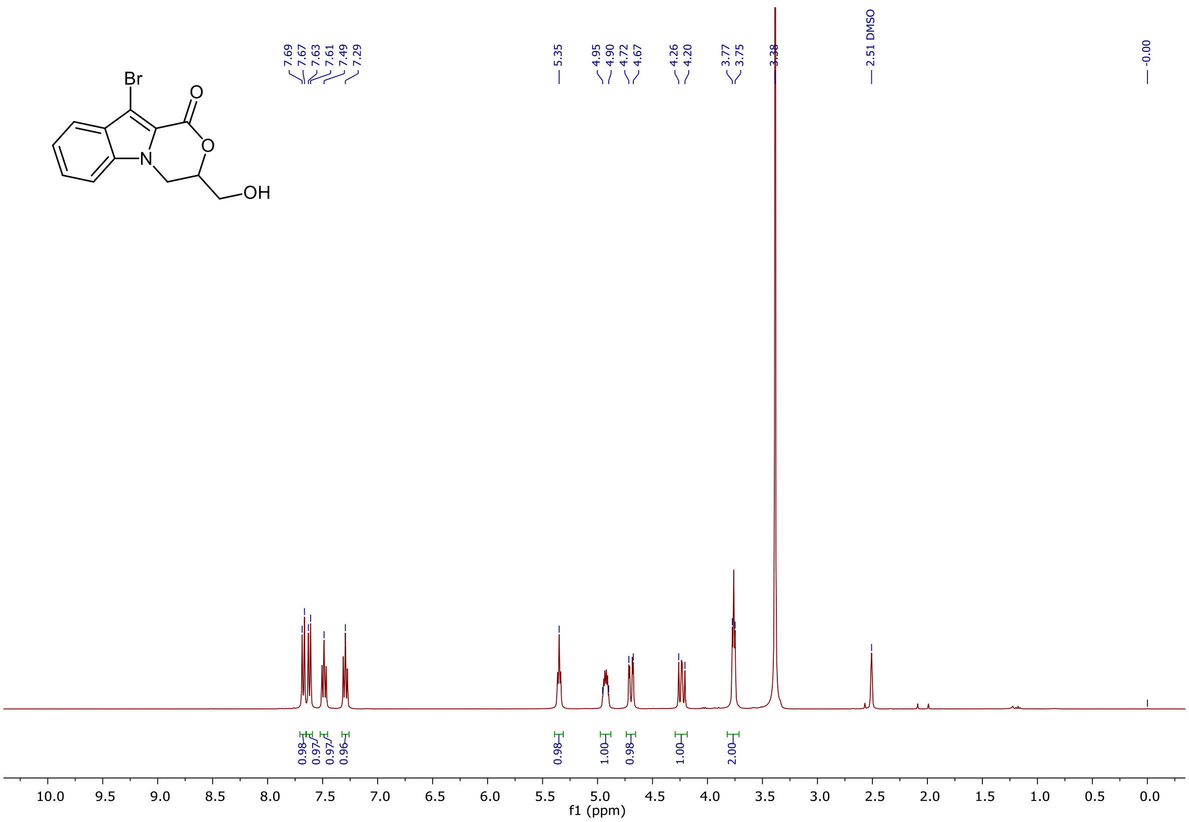

Figure S67. ^{1}H NMR (400 MHz, DMSO-d_6) spectrum of **9b**

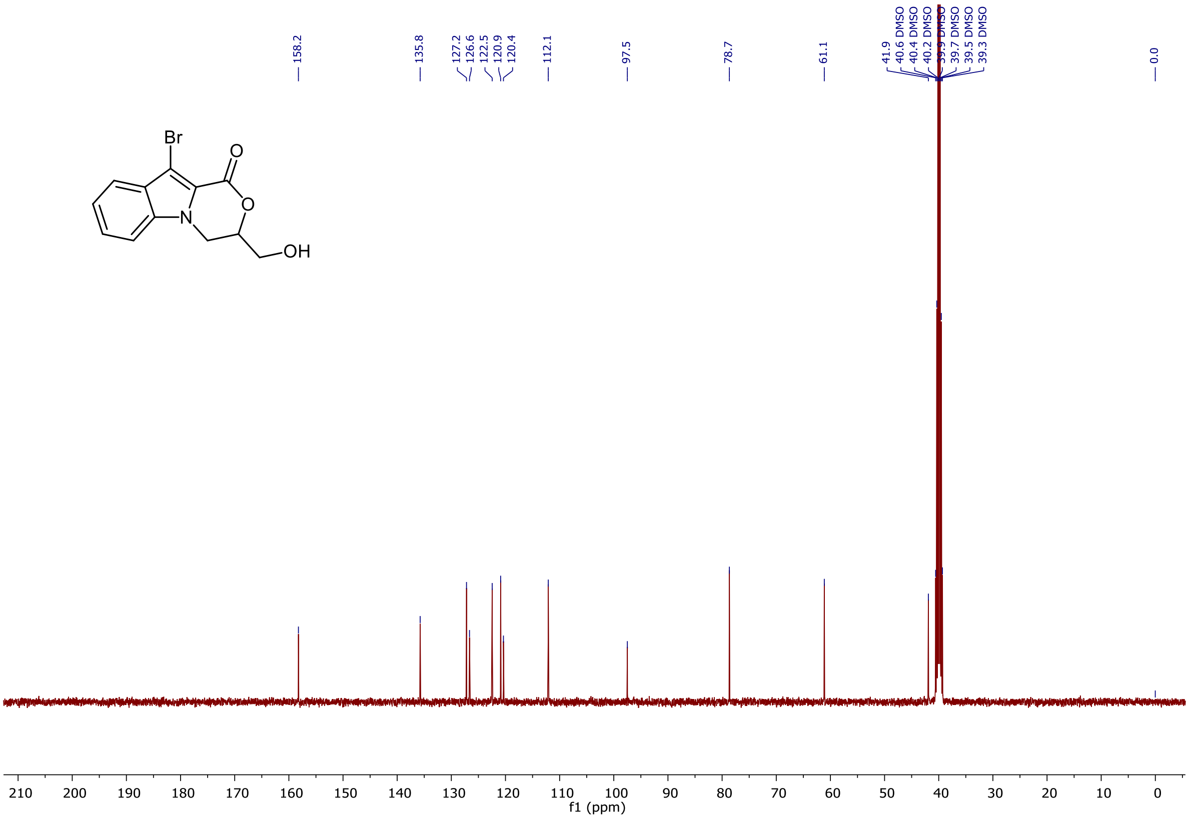

Figure S68. ^{13}C NMR (101 MHz, DMSO-d_6) spectrum of **9b**

Compound Spectrum SmartFormula Report

Analysis Info

Analysis Name	D:\Data\IZ-318.d	Acquisition Date	3/17/2025 7:37:18 PM
Method	DirectInfusion_TuneLow_pos.m	Operator	hplc
Sample Name	IZ-318	Instrument	micrOTOF-Q III 8228888.20448
Comment	AB		

Acquisition Parameter

Source Type	ESI	Ion Polarity	Positive	Set Nebulizer	0.4 Bar
Focus	Not active	Set Capillary	4500 V	Set Dry Heater	180 °C
Scan Begin	50 m/z	Set End Plate Offset	-500 V	Set Dry Gas	4.0 l/min
Scan End	1000 m/z	Set Collision Cell RF	140.0 Vpp	Set Divert Valve	Waste

#	RT [min]	Area	Int. Type	I	S/N	Chromatogram	Max. m/z	FWHM [min]
n.a.	5.6	n.a.	Single spectrum	n.a.	n.a.	n.a.	317.9739	n.a.

+MS, 5.6min #334

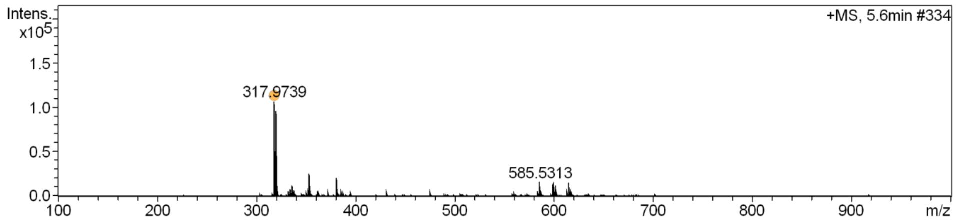

Meas. m/z	#	Ion Formula	m/z	err [ppm]	mSigma	# Sigma	Score	rdb	e^- Conf	N-Rule
317.9739	1	C12H10BrNNaO3	317.9736	0.8	39.9	2	100.00	7.5	even	ok

Figure S69. HRMS (ESI) report of **9b**

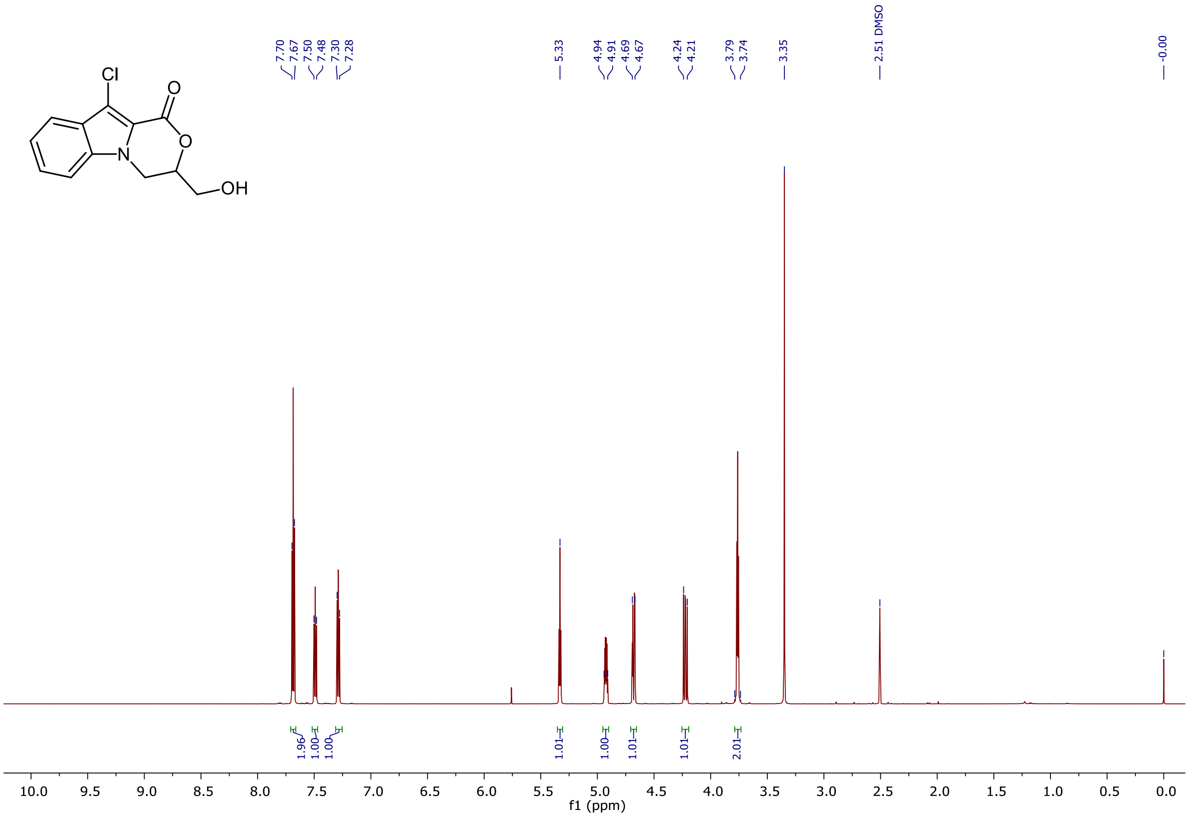

Figure S70. ^{1}H NMR (700 MHz, DMSO-d_6) spectrum of **9c**

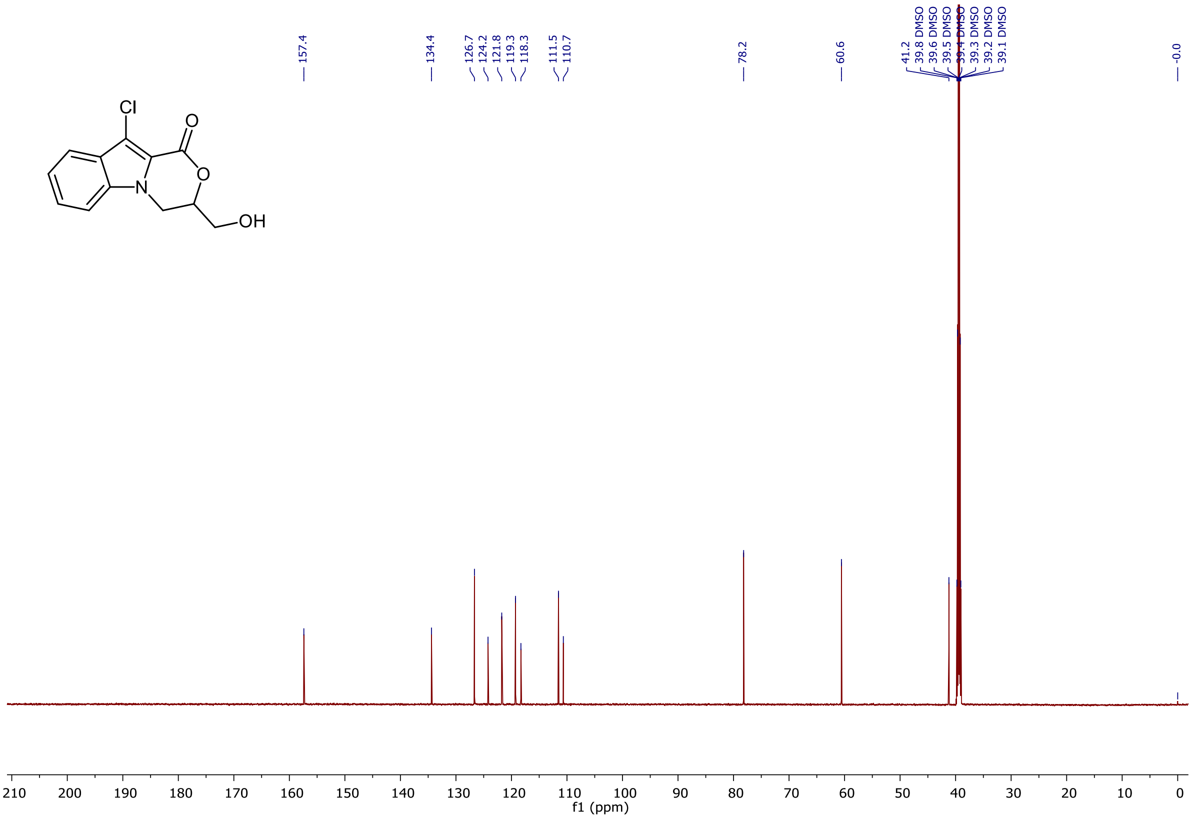

Figure S71. ^{13}C NMR (176 MHz, DMSO-d_6) spectrum of **9c**

Compound Spectrum SmartFormula Report

Analysis Info

Analysis Name	D:\Data\IZ-320.d	Acquisition Date	5/7/2025 1:28:22 AM
Method	DirectInfusion_TuneLow_pos.m	Operator	hplc
Sample Name	IZ-320	Instrument	micrOTOF-Q III 8228888.20448
Comment	AB		

Acquisition Parameter

Source Type	ESI	Ion Polarity	Positive	Set Nebulizer	0.4 Bar
Focus	Not active	Set Capillary	4500 V	Set Dry Heater	180 °C
Scan Begin	50 m/z	Set End Plate Offset	-500 V	Set Dry Gas	4.0 l/min
Scan End	1000 m/z	Set Collision Cell RF	140.0 Vpp	Set Divert Valve	Waste

#	RT [min]	Area	Int. Type	I	S/N	Chromatogram	Max. m/z	FWHM [min]
n.a.	5.7	n.a.	Single spectrum	n.a.	n.a.	n.a.	274.0239	n.a.

+MS, 5.7min #342

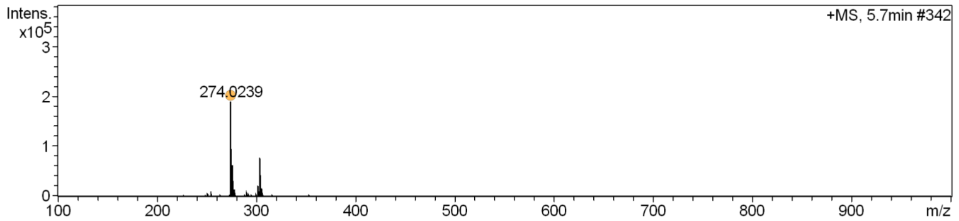

Meas. m/z	#	Ion Formula	m/z	err [ppm]	mSigma	# Sigma	Score	rdb	e⁻ Conf	N-Rule
274.0239	1	C12H10ClNNaO3	274.0241	0.9	8.2	2	100.00	7.5	even	ok

Figure S72. HRMS (ESI) report of **9c**

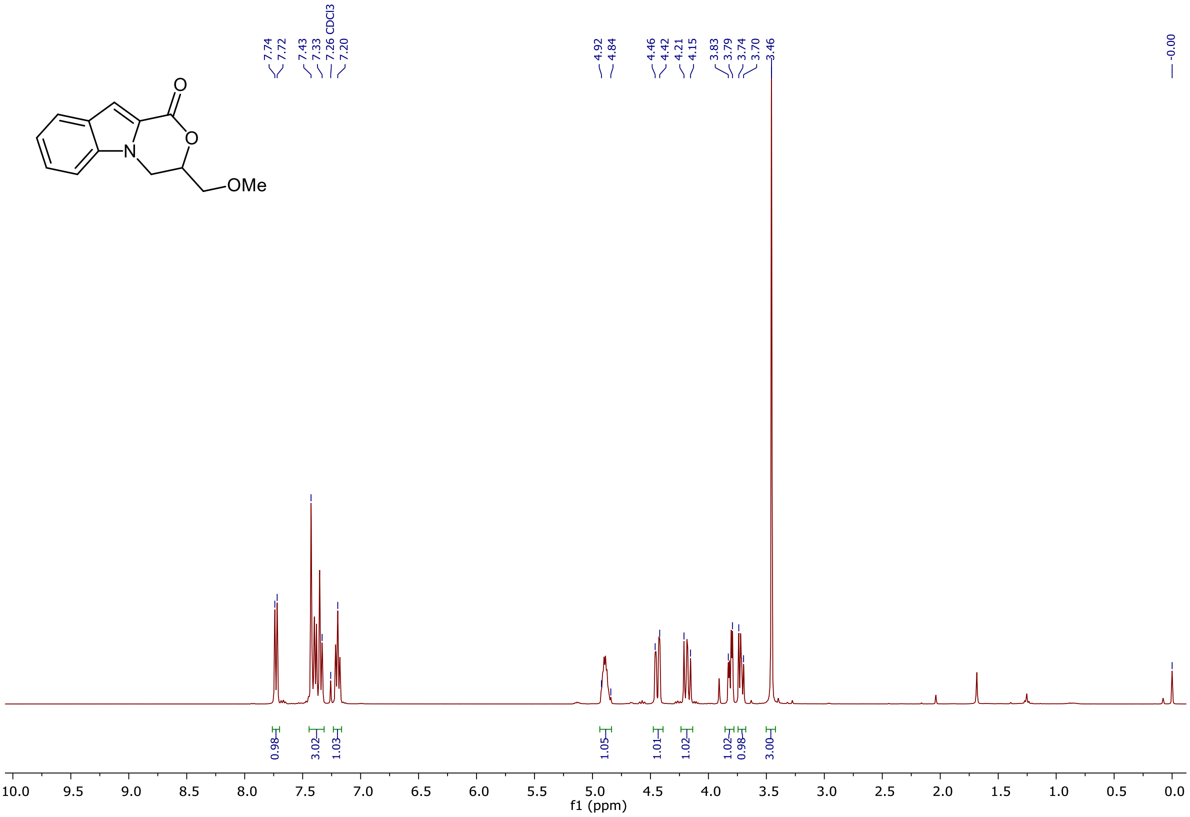

Figure S73. ^{1}H NMR (400 MHz, $CDCl_3$) spectrum of **10a**

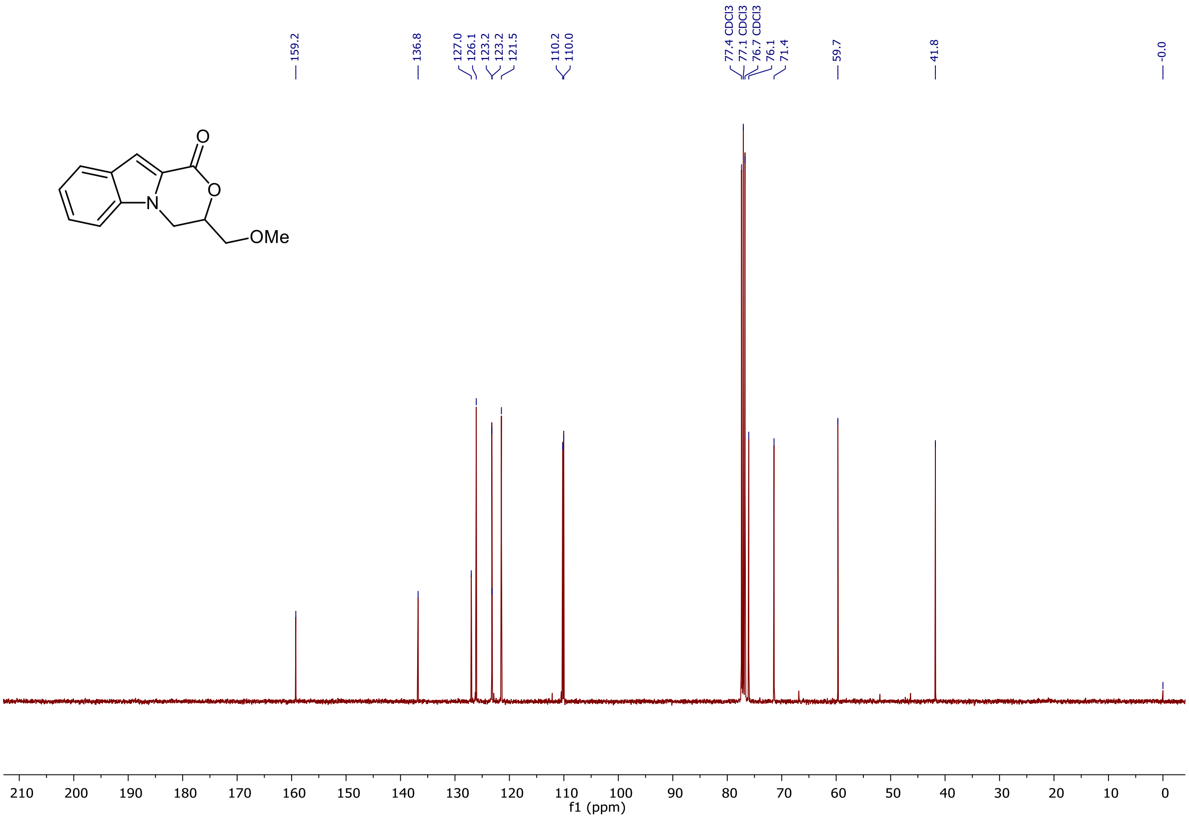

Figure S74. ^{13}C NMR (101 MHz, $CDCl_3$) spectrum of **10a**

Compound Spectrum SmartFormula Report

**Analysis Info**			
Analysis Name	D:\Data\IZ-254.d	Acquisition Date	5/7/2025 12:58:51 AM
Method	DirectInfusion_TuneLow_pos.m	Operator	hplc
Sample Name	IZ-254	Instrument	micrOTOF-Q III 8228888.20448
Comment	AB		

Acquisition Parameter

Source Type	ESI	Ion Polarity	Positive	Set Nebulizer	0.4 Bar
Focus	Not active	Set Capillary	4500 V	Set Dry Heater	180 °C
Scan Begin	50 m/z	Set End Plate Offset	-500 V	Set Dry Gas	4.0 l/min
Scan End	1000 m/z	Set Collision Cell RF	140.0 Vpp	Set Divert Valve	Waste

#	RT [min]	Area	Int. Type	I	S/N	Chromatogram	Max. m/z	FWHM [min]
n.a.	3.5	n.a.	Single spectrum	n.a.	n.a.	n.a.	254.0790	n.a.

+MS, 3.5min #210

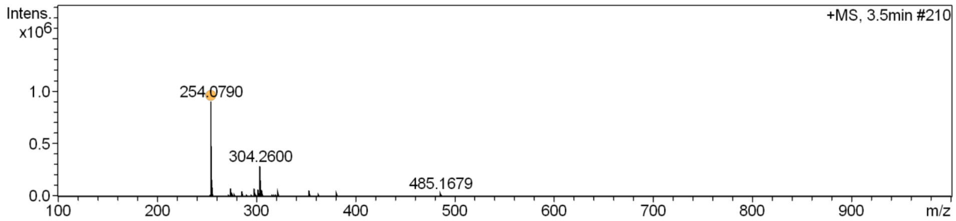

Meas. m/z	#	Ion Formula	m/z	err [ppm]	mSigma	# Sigma	Score	rdb	e⁻ Conf	N-Rule
254.0790	1	C13H13NNaO3	254.0788	-1.0	16.8	1	100.00	7.5	even	ok

Figure S75. HRMS (ESI) report of **10a**

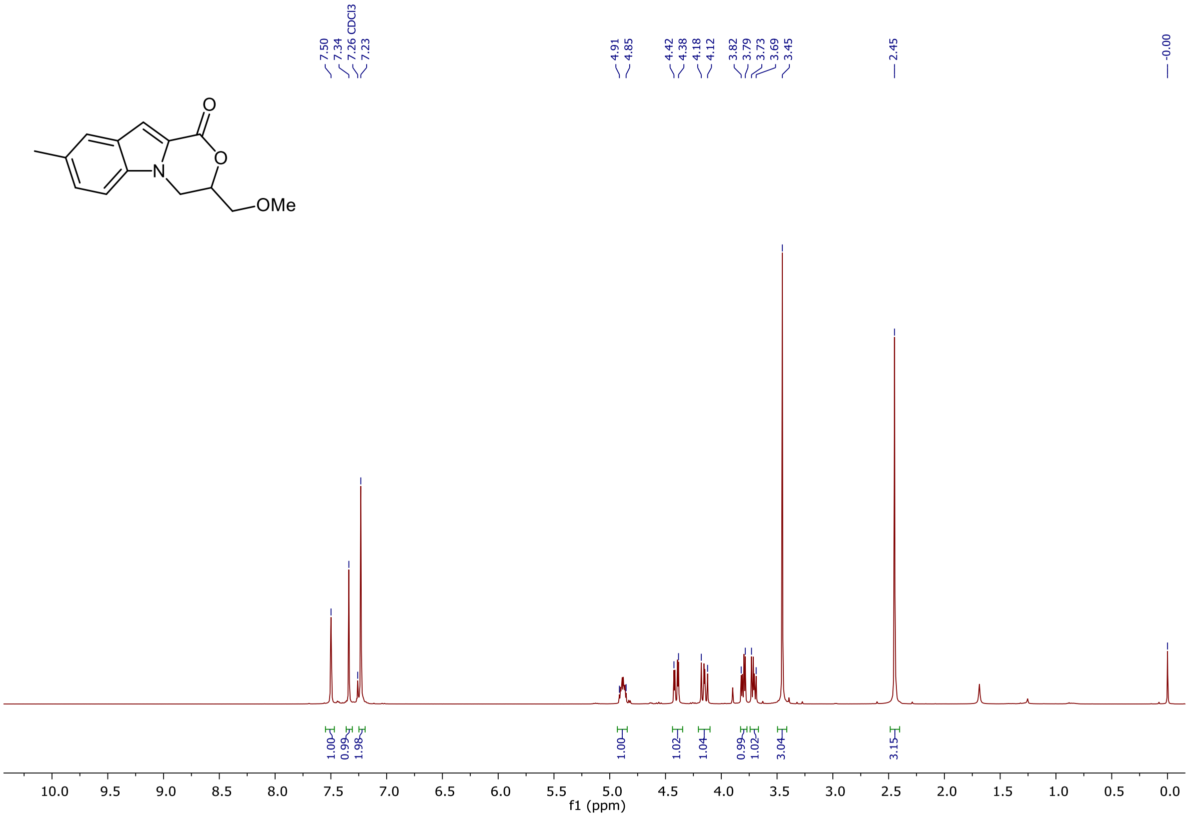

Figure S76. ^{1}H NMR (400 MHz, $CDCl_3$) spectrum of **10b**

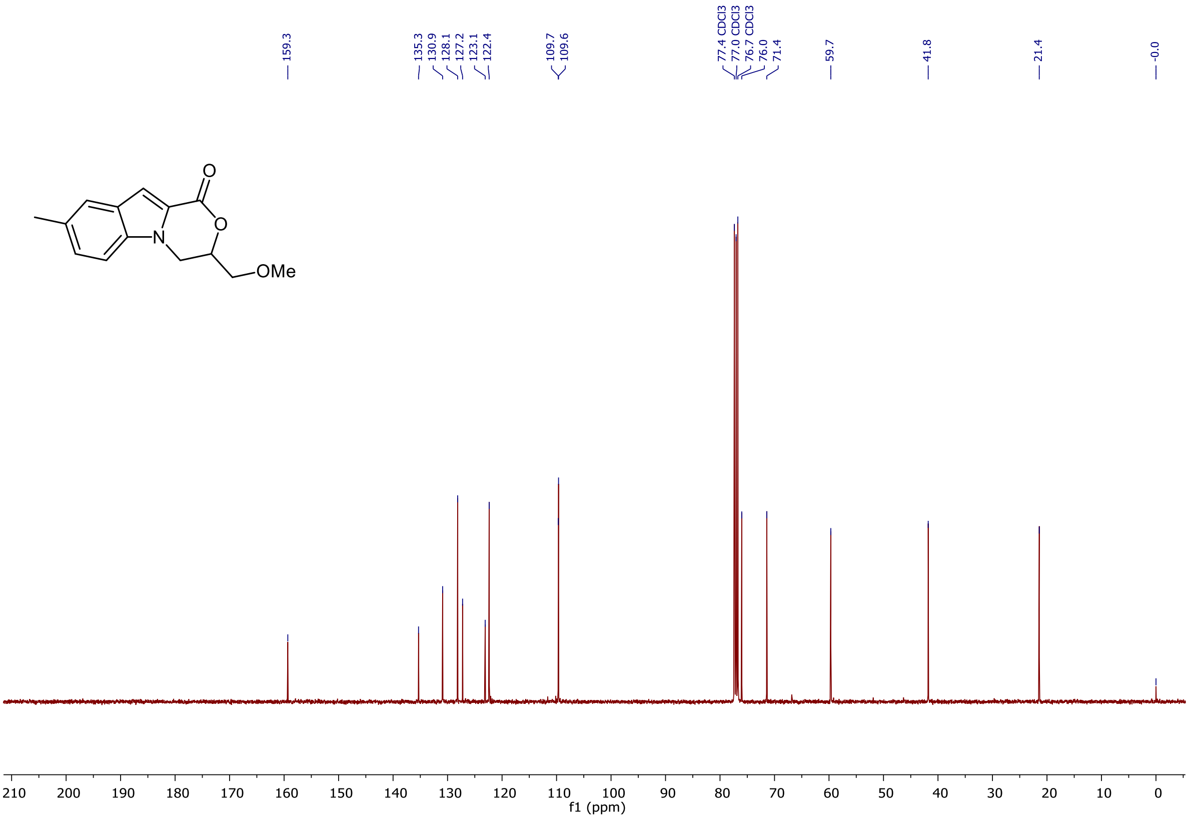

Figure S77. ^{13}C NMR (101 MHz, $CDCl_3$) spectrum of **10b**

Compound Spectrum SmartFormula Report

Analysis Info

Analysis Name	D:\Data\IZ-263.d	Acquisition Date	3/17/2025 7:53:56 PM
Method	DirectInfusion_TuneLow_pos.m	Operator	hplc
Sample Name	IZ-263	Instrument	micrOTOF-Q III 8228888.20448
Comment	AB		

Acquisition Parameter

Source Type	ESI	Ion Polarity	Positive	Set Nebulizer	0.4 Bar
Focus	Not active	Set Capillary	4500 V	Set Dry Heater	180 °C
Scan Begin	50 m/z	Set End Plate Offset	-500 V	Set Dry Gas	4.0 l/min
Scan End	1000 m/z	Set Collision Cell RF	140.0 Vpp	Set Divert Valve	Waste

#	RT [min]	Area	Int. Type	I	S/N	Chromatogram	Max. m/z	FWHM [min]
n.a.	7.6	n.a.	Single spectrum	n.a.	n.a.	n.a.	268.0941	n.a.

+MS, 7.6min #458

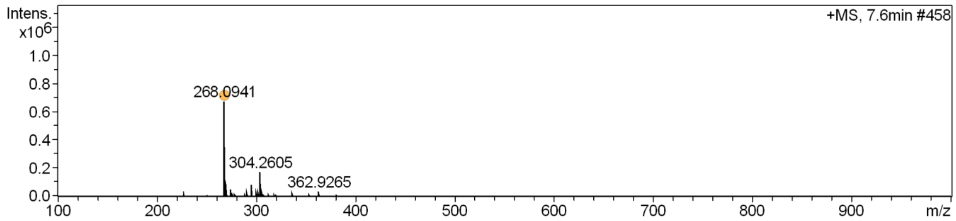

Meas. m/z	#	Ion Formula	m/z	err [ppm]	mSigma	# Sigma	Score	rdb	e⁻ Conf	N-Rule
268.0941	1	C14H15NNaO3	268.0944	1.1	7.7	1	100.00	7.5	even	ok

Figure S78. HRMS (ESI) report of **10b**

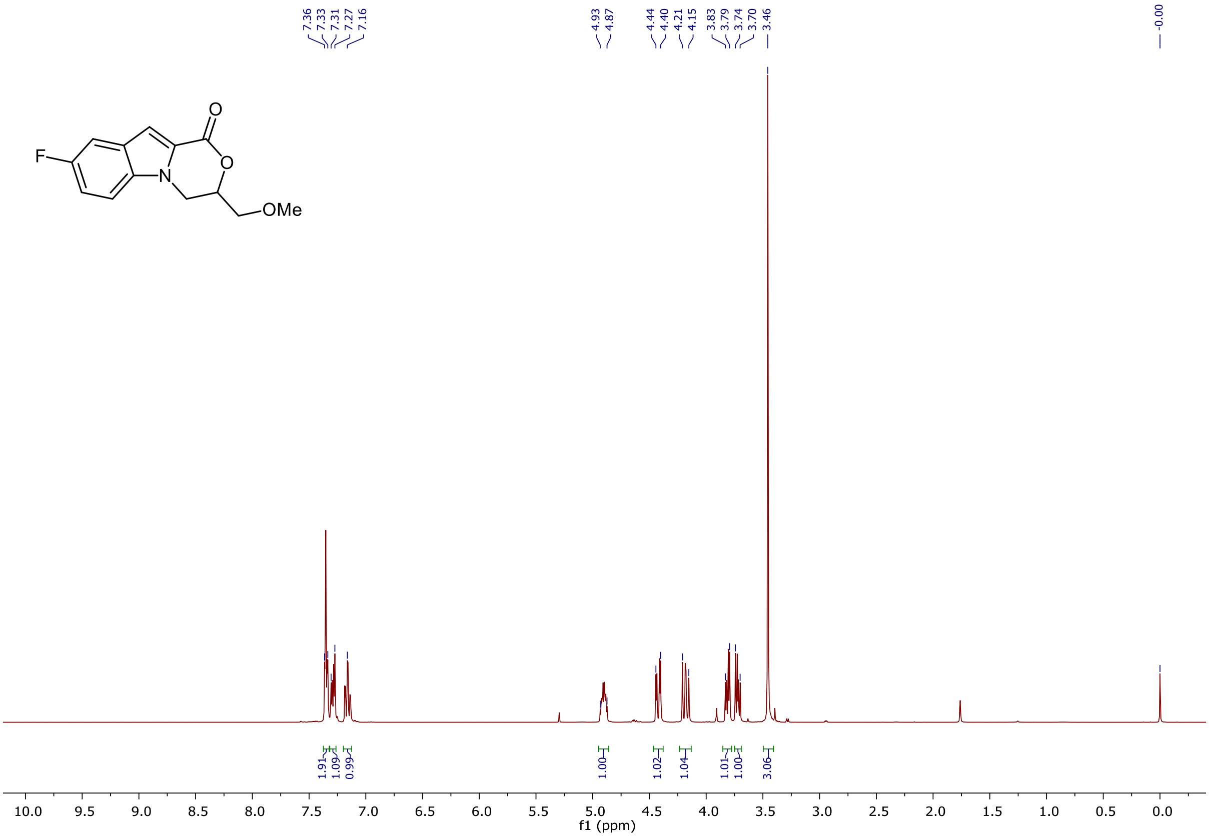

Figure S79. ^{1}H NMR (400 MHz, $CDCl_3$) spectrum of **10c**

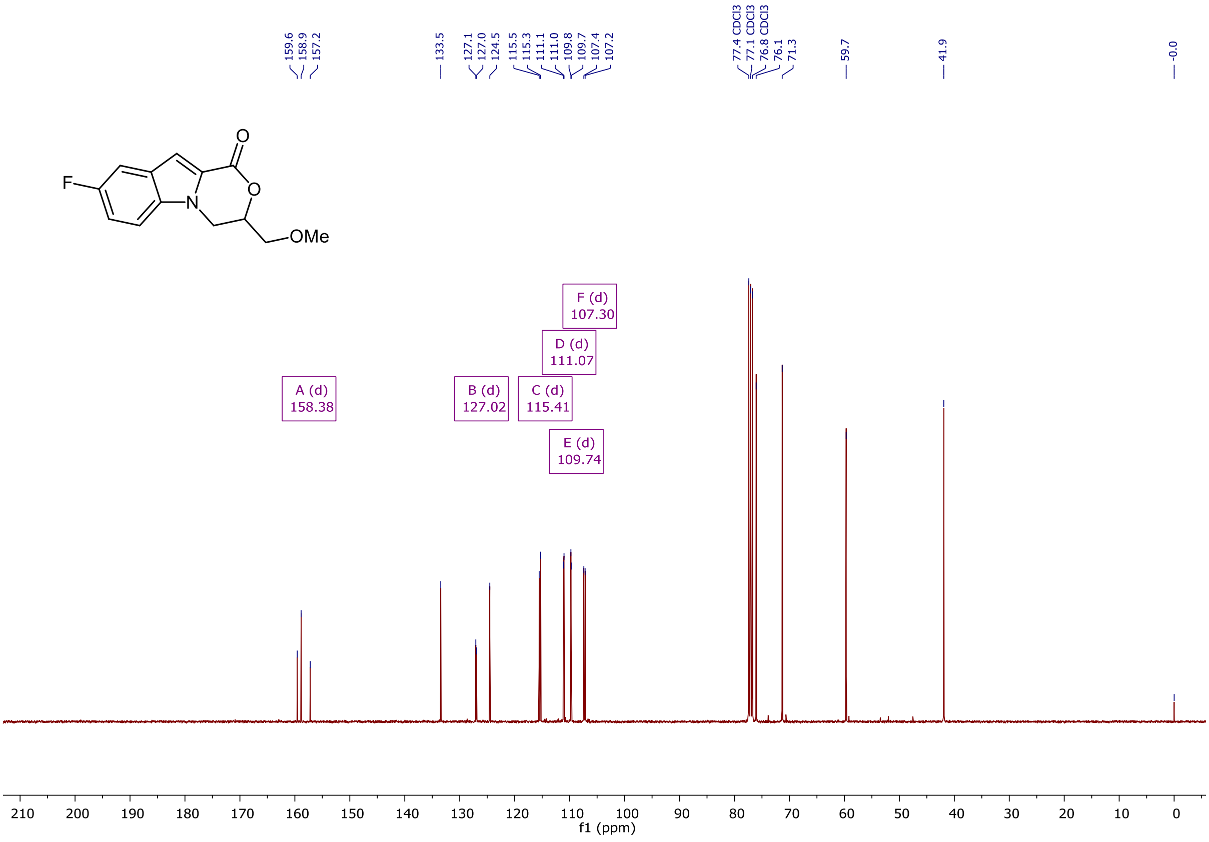

Figure S80. ^{13}C NMR (101 MHz, $CDCl_3$) spectrum of **10c**

Compound Spectrum SmartFormula Report

Analysis Info

Analysis Name	D:\Data\IZ-270.d	Acquisition Date	5/7/2025 3:44:40 AM
Method	DirectInfusion_TuneLow_pos.m	Operator	hplc
Sample Name	IZ-270	Instrument	micrOTOF-Q III 8228888.20448
Comment	AB		

Acquisition Parameter

Source Type	ESI	Ion Polarity	Positive	Set Nebulizer	0.4 Bar
Focus	Not active	Set Capillary	4500 V	Set Dry Heater	180 °C
Scan Begin	50 m/z	Set End Plate Offset	-500 V	Set Dry Gas	4.0 l/min
Scan End	1000 m/z	Set Collision Cell RF	140.0 Vpp	Set Divert Valve	Waste

#	RT [min]	Area	Int. Type	I	S/N	Chromatogram	Max. m/z	FWHM [min]
n.a.	6.6	n.a.	Single spectrum	n.a.	n.a.	n.a.	272.0694	n.a.

+MS, 6.6min #395

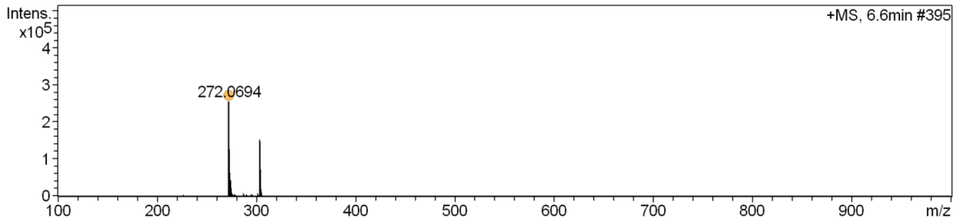

Meas. m/z	#	Ion Formula	m/z	err [ppm]	mSigma	# Sigma	Score	rdb	e⁻ Conf	N-Rule
272.0694	1	C13H12FNNaO3	272.0693	-0.2	58.1	2	100.00	7.5	even	ok

Figure S81. HRMS (ESI) report of **10c**

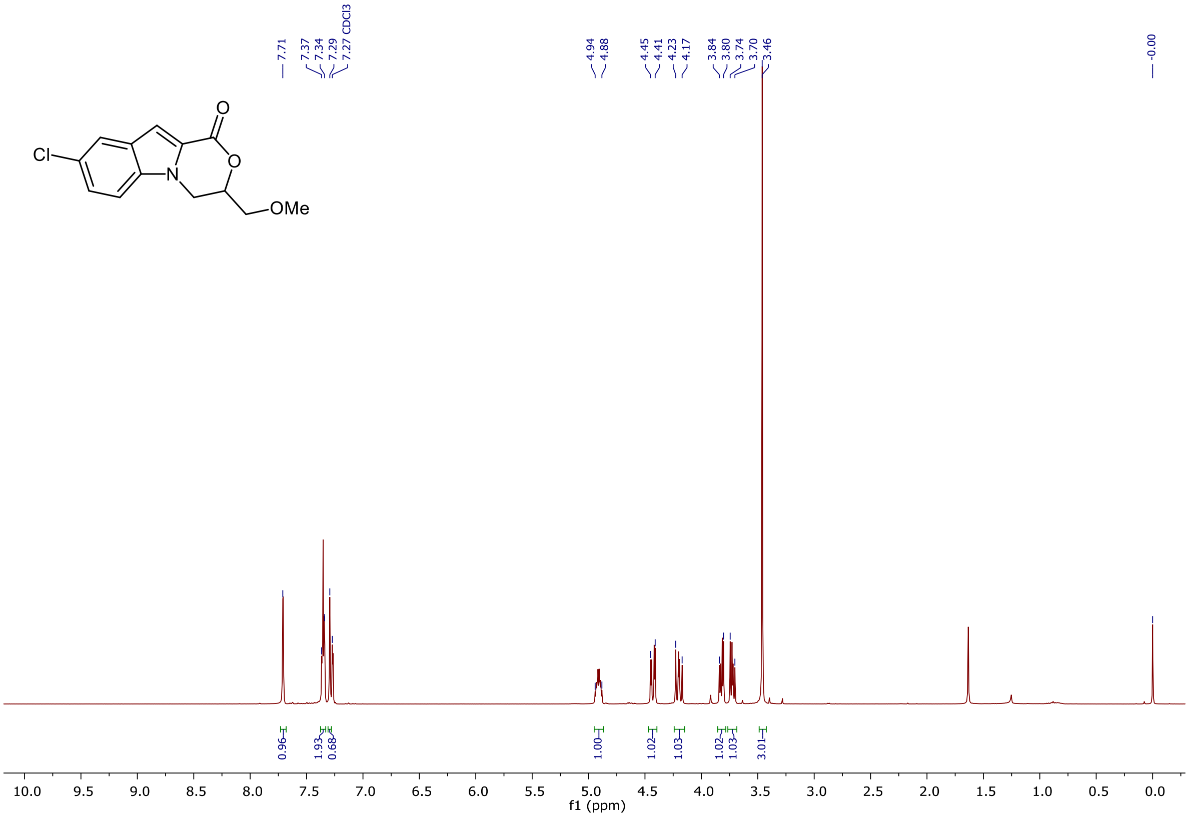

Figure S82. ^{1}H NMR (400 MHz, $CDCl_3$) spectrum of **10d**

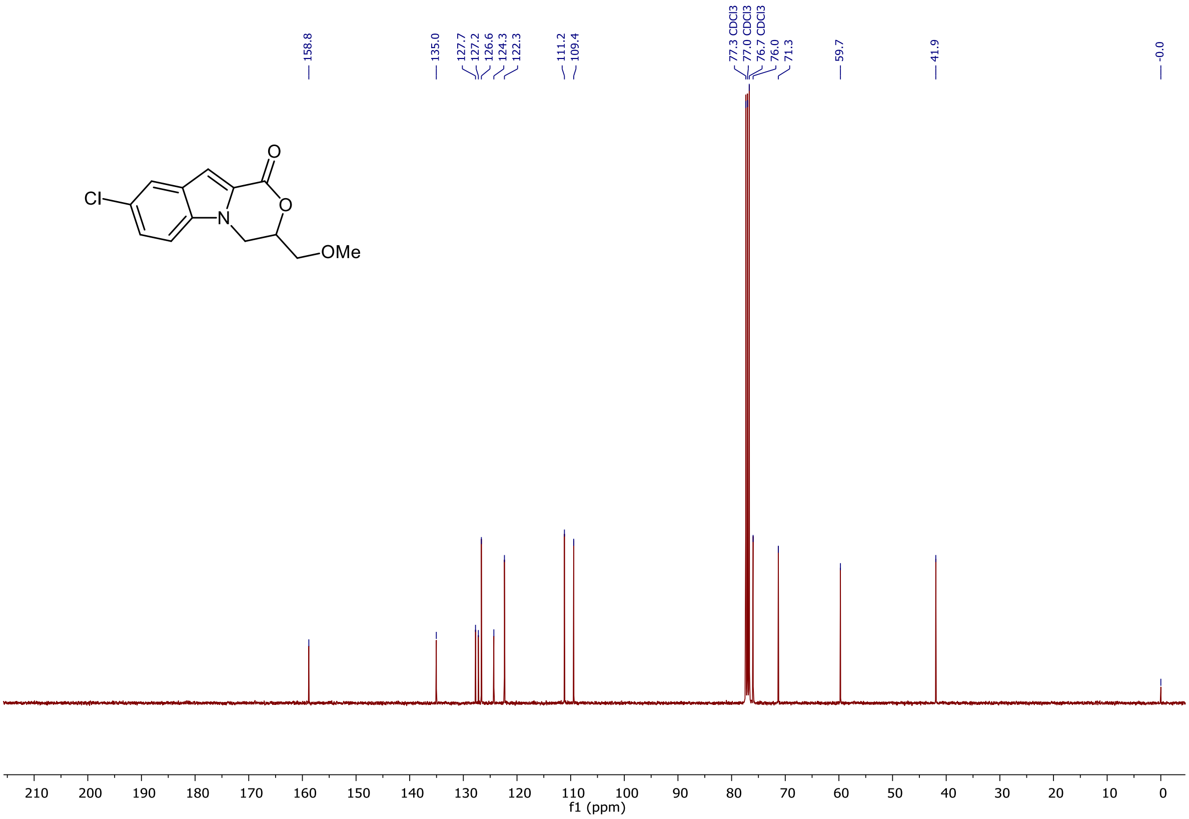

Figure S83. ^{13}C NMR (101 MHz, $CDCl_3$) spectrum of **10d**

Compound Spectrum SmartFormula Report

Analysis Info

Analysis Name	D:\Data\IZ-267.d	Acquisition Date	5/7/2025 3:30:01 AM
Method	DirectInfusion_TuneLow_pos.m	Operator	hplc
Sample Name	IZ-267	Instrument	micrOTOF-Q III 8228888.20448
Comment	AB		

Acquisition Parameter

Source Type	ESI	Ion Polarity	Positive	Set Nebulizer	0.4 Bar
Focus	Not active	Set Capillary	4500 V	Set Dry Heater	180 °C
Scan Begin	50 m/z	Set End Plate Offset	-500 V	Set Dry Gas	4.0 l/min
Scan End	1000 m/z	Set Collision Cell RF	140.0 Vpp	Set Divert Valve	Waste

#	RT [min]	Area	Int. Type	I	S/N	Chromatogram	Max. m/z	FWHM [min]
n.a.	4.8	n.a.	Single spectrum	n.a.	n.a.	n.a.	288.0399	n.a.

+MS, 4.8min #286

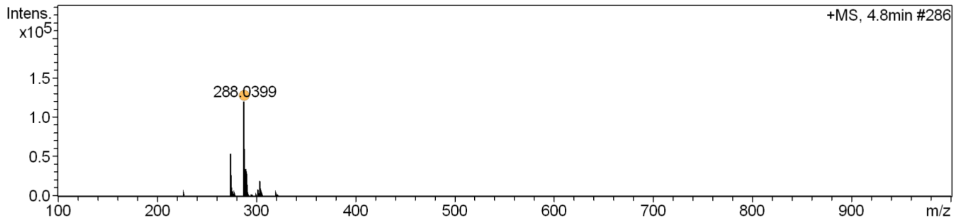

Meas. m/z	#	Ion Formula	m/z	err [ppm]	mSigma	# Sigma	Score	rdb	e⁻ Conf	N-Rule
288.0399	1	C13H12ClNNaO3	288.0398	-0.4	20.9	1	100.00	7.5	even	ok

Figure S84. HRMS (ESI) report of **10d**

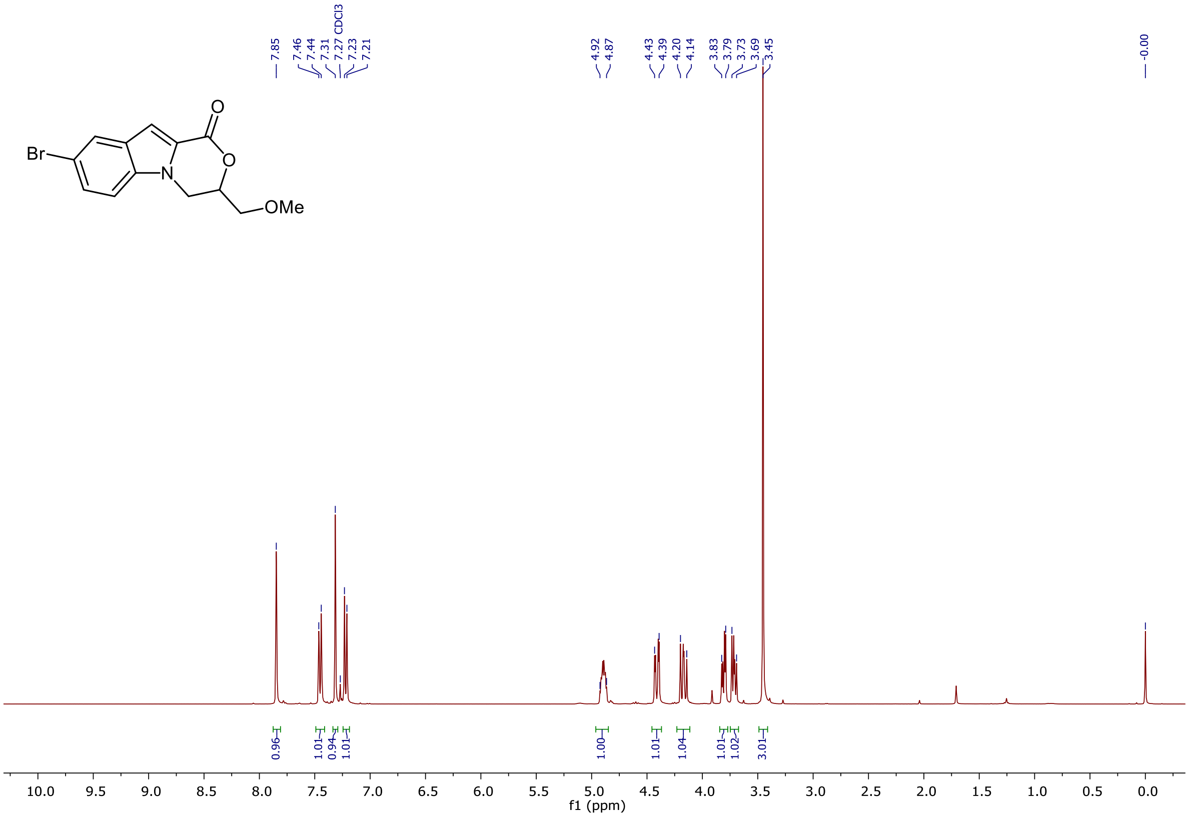

Figure S85. ^{1}H NMR (400 MHz, $CDCl_3$) spectrum of **10e**

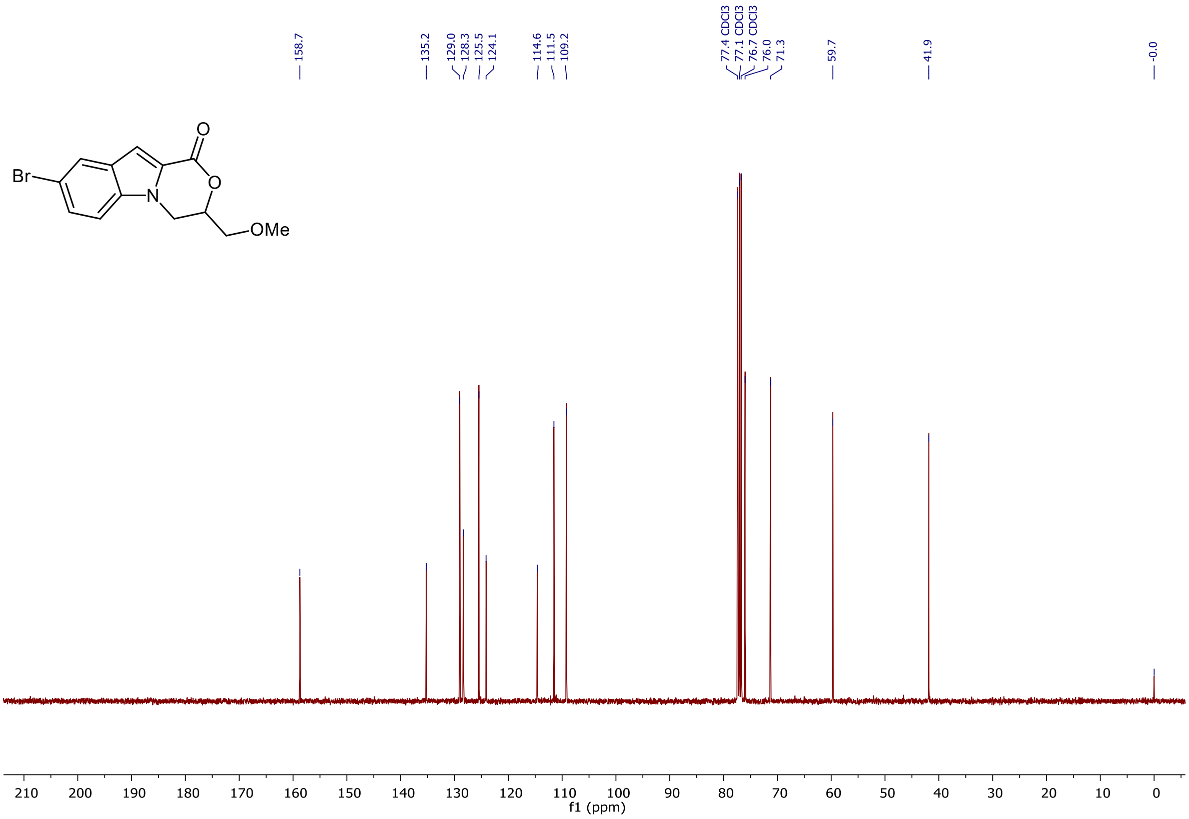

Figure S86. ^{13}C NMR (101 MHz, $CDCl_3$) spectrum of **10e**

Compound Spectrum SmartFormula Report

Analysis Info

Analysis Name	D:\Data\IZ-256.d	Acquisition Date	3/17/2025 8:14:58 PM
Method	DirectInfusion_TuneLow_pos.m	Operator	hplc
Sample Name	IZ-256	Instrument	micrOTOF-Q III 8228888.20448
Comment	AB		

Acquisition Parameter

Source Type	ESI	Ion Polarity	Positive	Set Nebulizer	0.4 Bar
Focus	Not active	Set Capillary	4500 V	Set Dry Heater	180 °C
Scan Begin	50 m/z	Set End Plate Offset	-500 V	Set Dry Gas	4.0 l/min
Scan End	1000 m/z	Set Collision Cell RF	140.0 Vpp	Set Divert Valve	Waste

#	RT [min]	Area	Int. Type	I	S/N	Chromatogram	Max. m/z	FWHM [min]
n.a.	8.8	n.a.	Single spectrum	n.a.	n.a.	n.a.	333.9874	n.a.

+MS, 8.8min #528

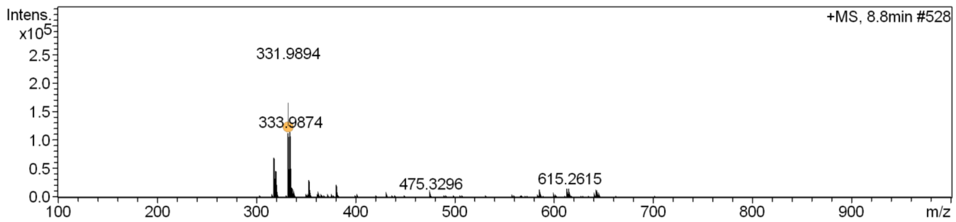

Meas. m/z	#	Ion Formula	m/z	err [ppm]	mSigma	# Sigma	Score	rdb	e⁻ Conf	N-Rule
331.9894	1	C13H12BrNNaO3	331.9893	0.3	10.6	1	100.00	7.5	even	ok

Figure S87. HRMS (ESI) report of **10e**

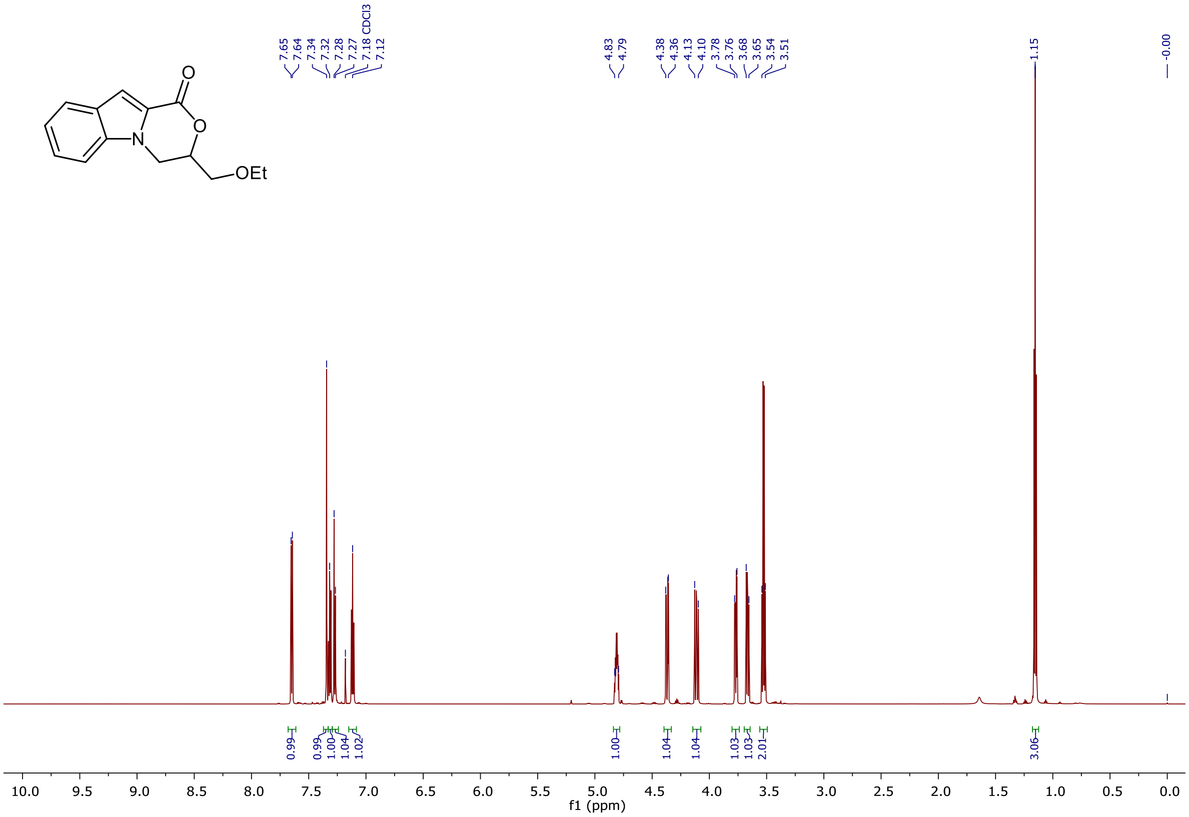

Figure S88. ^{1}H NMR (700 MHz, $CDCl_3$) spectrum of **10f**

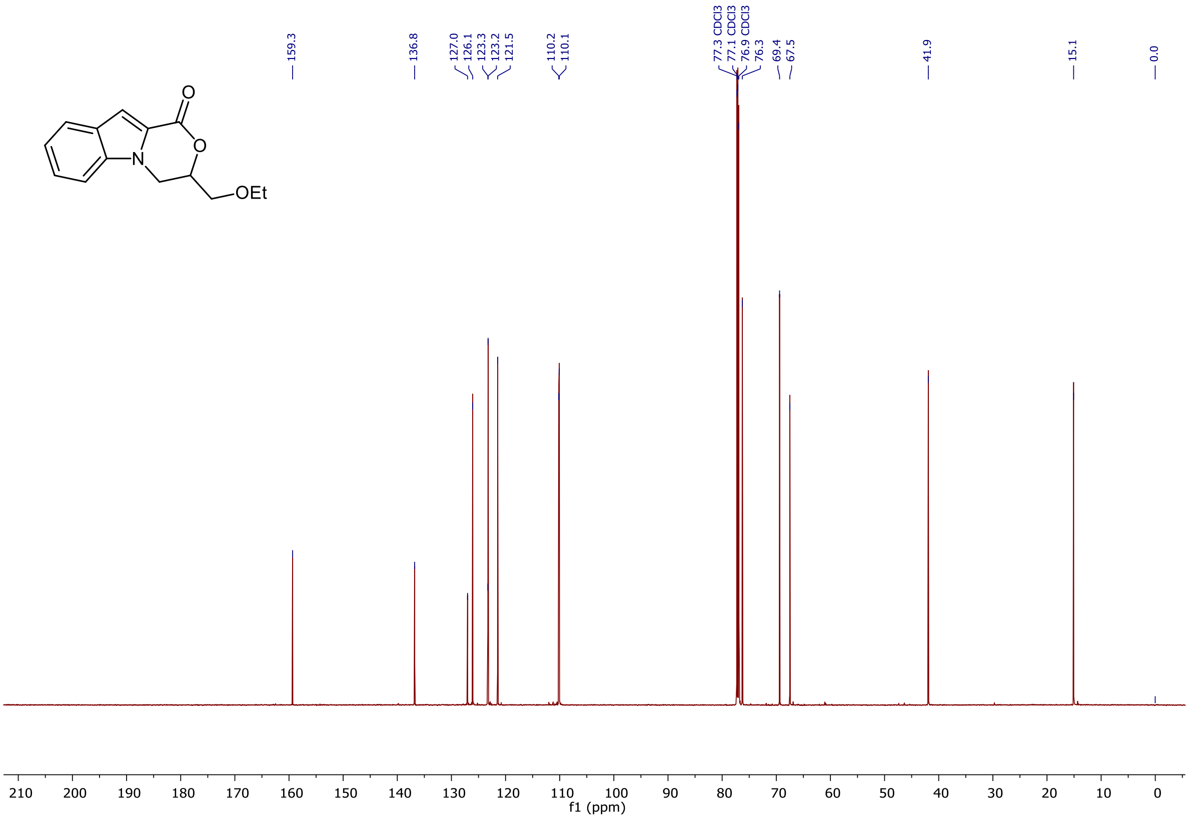

Figure S89. ^{13}C NMR (176 MHz, $CDCl_3$) spectrum of **10f**

Compound Spectrum SmartFormula Report

Analysis Info

Analysis Name	D:\Data\IZ-352.d	Acquisition Date	12/18/2024 1:38:50 PM
Method	DirectInfusion_TuneLow_pos.m	Operator	hplc
Sample Name	IZ-352	Instrument	micrOTOF-Q III 8228888.20448
Comment	SB		

Acquisition Parameter

Source Type	ESI	Ion Polarity	Positive	Set Nebulizer	0.4 Bar
Focus	Not active	Set Capillary	4500 V	Set Dry Heater	180 °C
Scan Begin	50 m/z	Set End Plate Offset	-500 V	Set Dry Gas	4.0 l/min
Scan End	1000 m/z	Set Collision Cell RF	140.0 Vpp	Set Divert Valve	Waste

#	RT [min]	Area	Int. Type	I	S/N	Chromatogram	Max. m/z	FWHM [min]
n.a.	0.3	n.a.	Single spectrum	n.a.	n.a.	n.a.	304.2629	n.a.
n.a.	3.9	n.a.	Single spectrum	n.a.	n.a.	n.a.	268.0944	n.a.

+MS, 3.9min #231

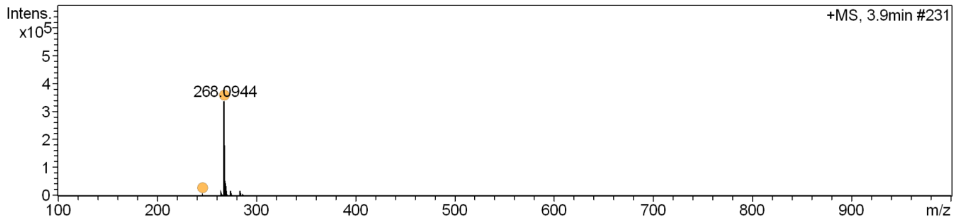

Meas. m/z	#	Ion Formula	m/z	err [ppm]	mSigma	# Sigma	Score	rdb	e⁻ Conf	N-Rule
246.1120	1	C14H16NO3	246.1125	-2.1	11.4	1	100.00	7.5	even	ok
268.0944	1	C14H15NNaO3	268.0944	-0.2	3.6	1	100.00	7.5	even	ok

Figure S90. HRMS (ESI) report of **10f**

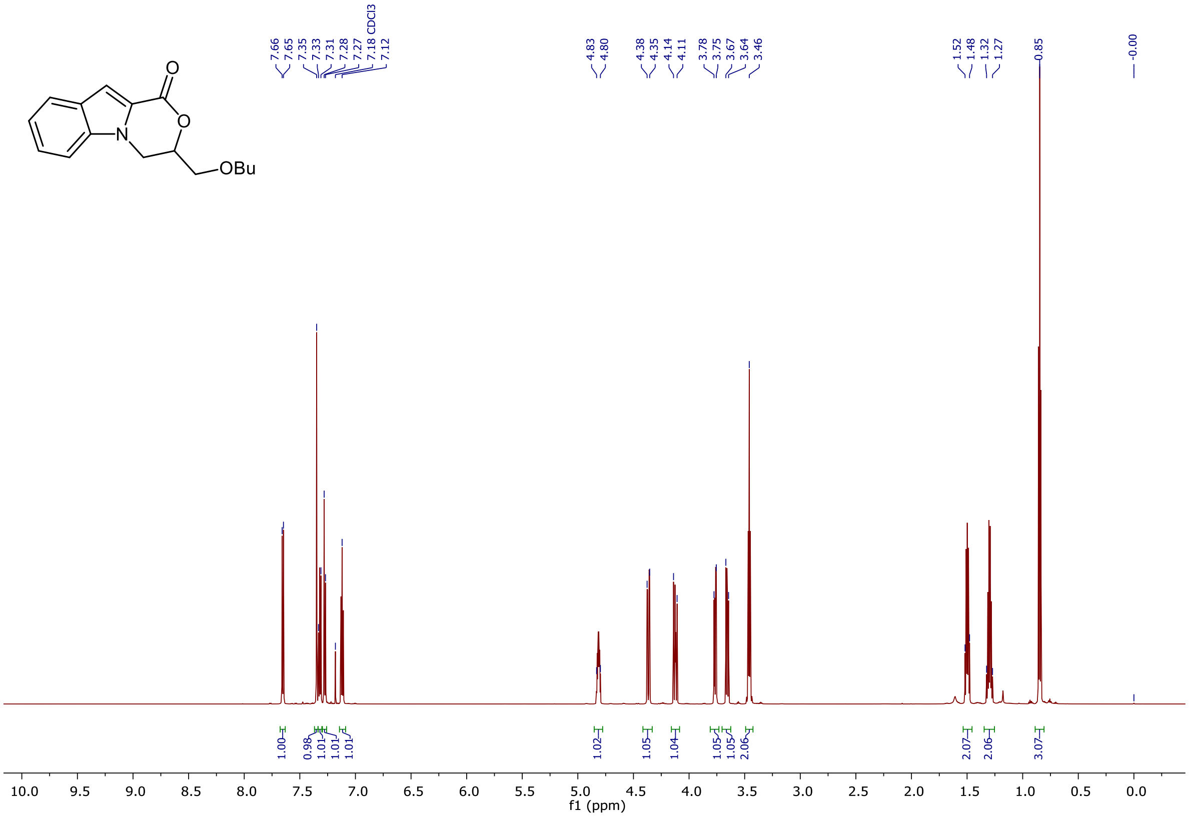

Figure S91. ^{1}H NMR (700 MHz, $CDCl_3$) spectrum of **10g**

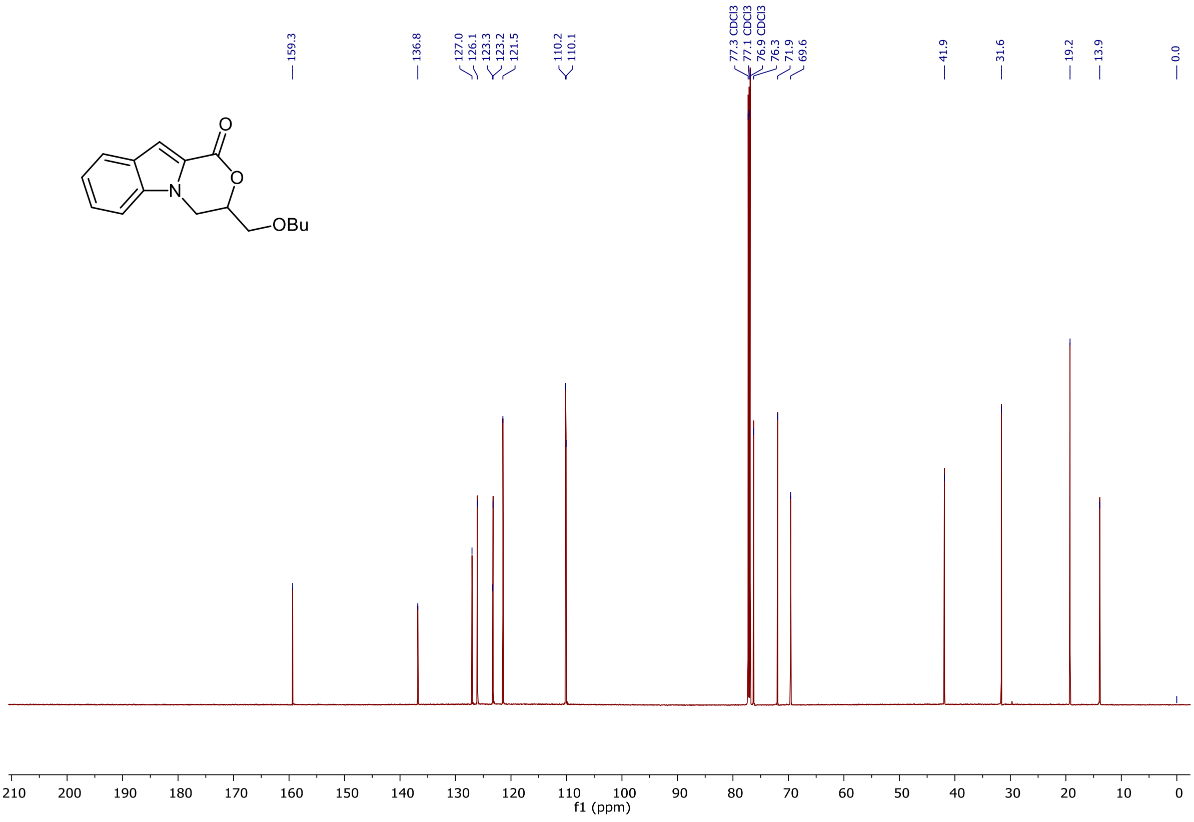

Figure S92. ^{13}C NMR (176 MHz, $CDCl_3$) spectrum of **10g**

Compound Spectrum SmartFormula Report

Analysis Info

Analysis Name	D:\Data\IZ-353.d	Acquisition Date	12/18/2024 1:56:10 PM
Method	DirectInfusion_TuneLow_pos.m	Operator	hplc
Sample Name	IZ-353	Instrument	micrOTOF-Q III 8228888.20448
Comment	SB		

Acquisition Parameter

Source Type	ESI	Ion Polarity	Positive	Set Nebulizer	0.4 Bar
Focus	Not active	Set Capillary	4500 V	Set Dry Heater	180 °C
Scan Begin	50 m/z	Set End Plate Offset	-500 V	Set Dry Gas	4.0 l/min
Scan End	1000 m/z	Set Collision Cell RF	140.0 Vpp	Set Divert Valve	Waste

#	RT [min]	Area	Int. Type	I	S/N	Chromatogram	Max. m/z	FWHM [min]
n.a.	0.1	n.a.	Single spectrum	n.a.	n.a.	n.a.	304.2628	n.a.
n.a.	3.6	n.a.	Single spectrum	n.a.	n.a.	n.a.	296.1257	n.a.

+MS, 3.6min #215

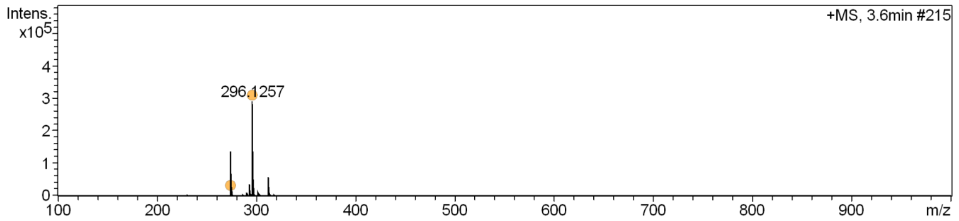

Meas. m/z	#	Ion Formula	m/z	err [ppm]	mSigma	# Sigma	Score	rdb	e⁻ Conf	N-Rule
274.1433	1	C16H20NO3	274.1438	1.8	16.3	1	100.00	7.5	even	ok
296.1257	1	C16H19NNaO3	296.1257	0.1	3.6	1	100.00	7.5	even	ok

Figure S93. HRMS (ESI) report of **10g**

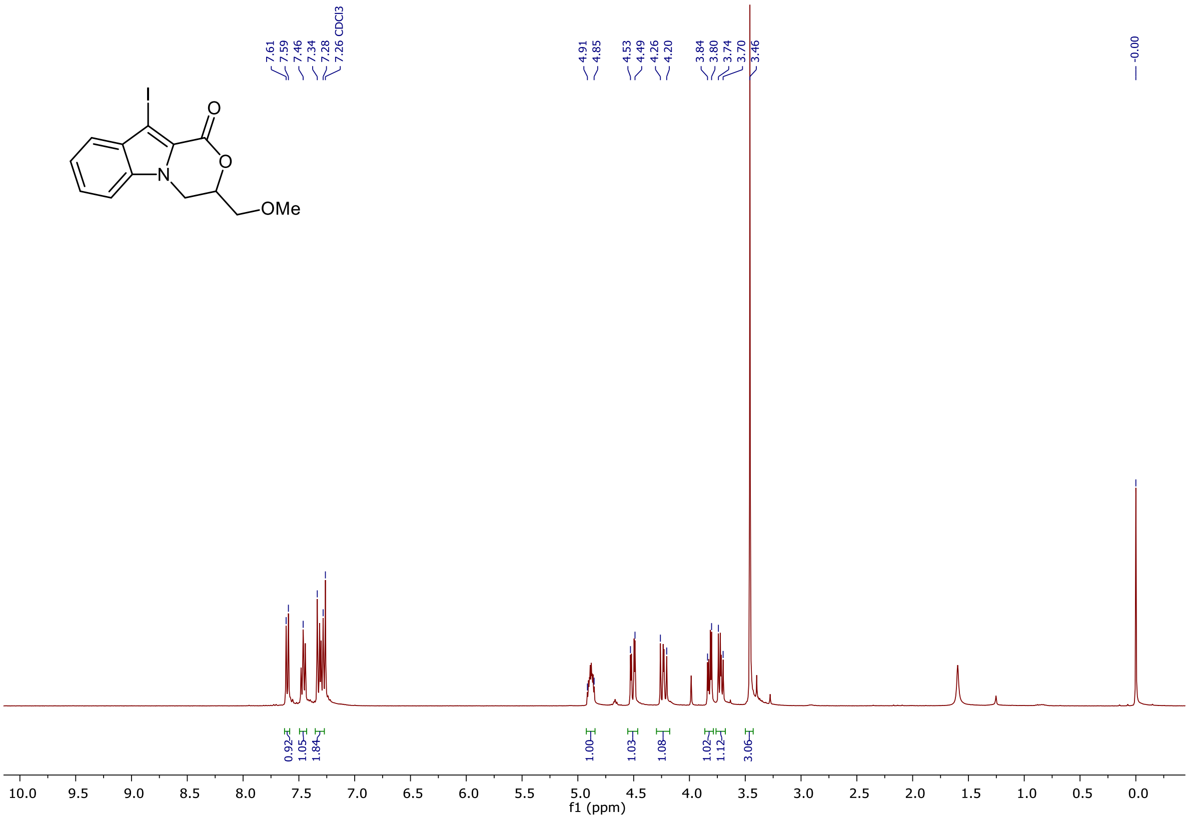

Figure S94. ^{1}H NMR (400 MHz, $CDCl_3$) spectrum of **11**

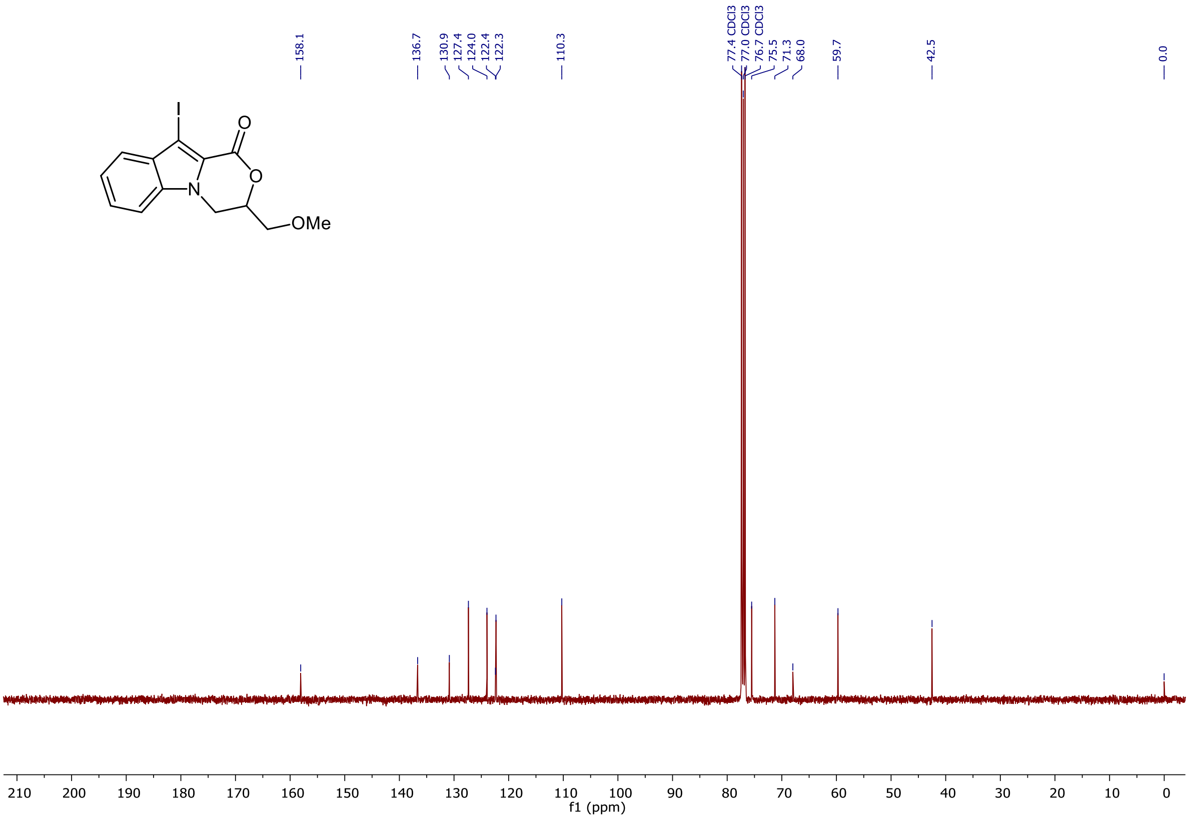

Figure S95. ^{13}C NMR (101 MHz, $CDCl_3$) spectrum of **11**

Compound Spectrum SmartFormula Report

Analysis Info

Analysis Name	D:\Data\IZ-323.d	Acquisition Date	3/18/2025 5:56:51 PM
Method	DirectInfusion_TuneLow_pos.m	Operator	hplc
Sample Name	IZ-323	Instrument	micrOTOF-Q III 8228888.20448
Comment	AB		

Acquisition Parameter

Source Type	ESI	Ion Polarity	Positive	Set Nebulizer	0.4 Bar
Focus	Not active	Set Capillary	4500 V	Set Dry Heater	180 °C
Scan Begin	50 m/z	Set End Plate Offset	-500 V	Set Dry Gas	4.0 l/min
Scan End	1000 m/z	Set Collision Cell RF	140.0 Vpp	Set Divert Valve	Waste

#	RT [min]	Area	Int. Type	I	S/N	Chromatogram	Max. m/z	FWHM [min]
n.a.	4.0	n.a.	Single spectrum	n.a.	n.a.	n.a.	379.9750	n.a.

+MS, 4.0min #237

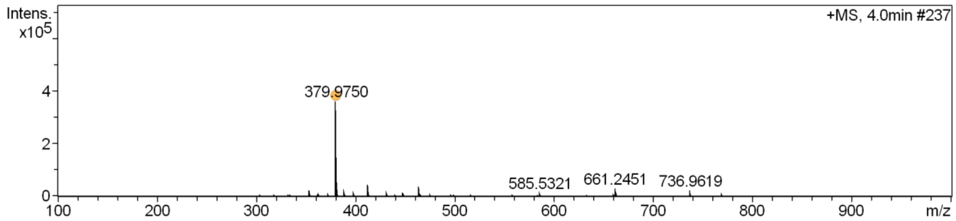

Meas. m/z	#	Ion Formula	m/z	err [ppm]	mSigma	# Sigma	Score	rdb	e⁻ Conf	N-Rule
379.9750	1	C13H12INNaO3	379.9754	-1.1	6.2	1	100.00	7.5	even	ok

Figure S96. HRMS (ESI) report of **11**

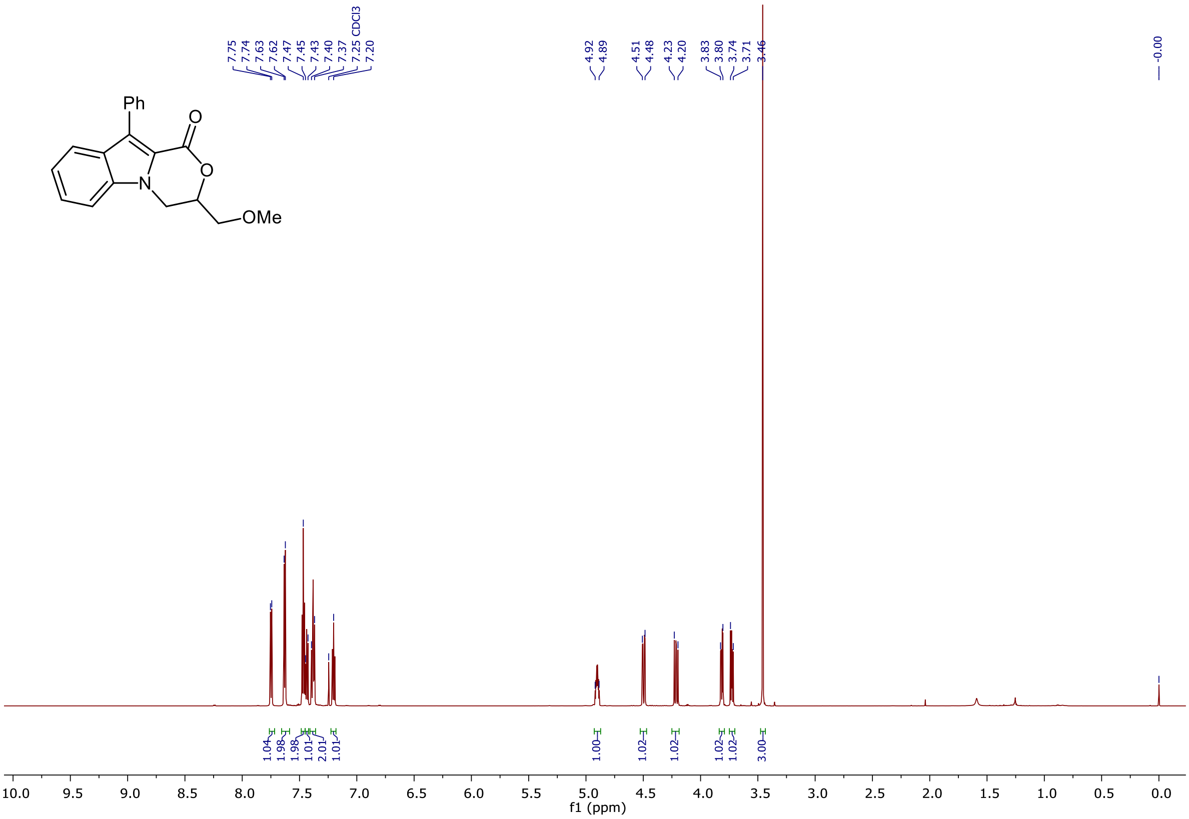

Figure S97. ^{1}H NMR (700 MHz, $CDCl_3$) spectrum of **12a**

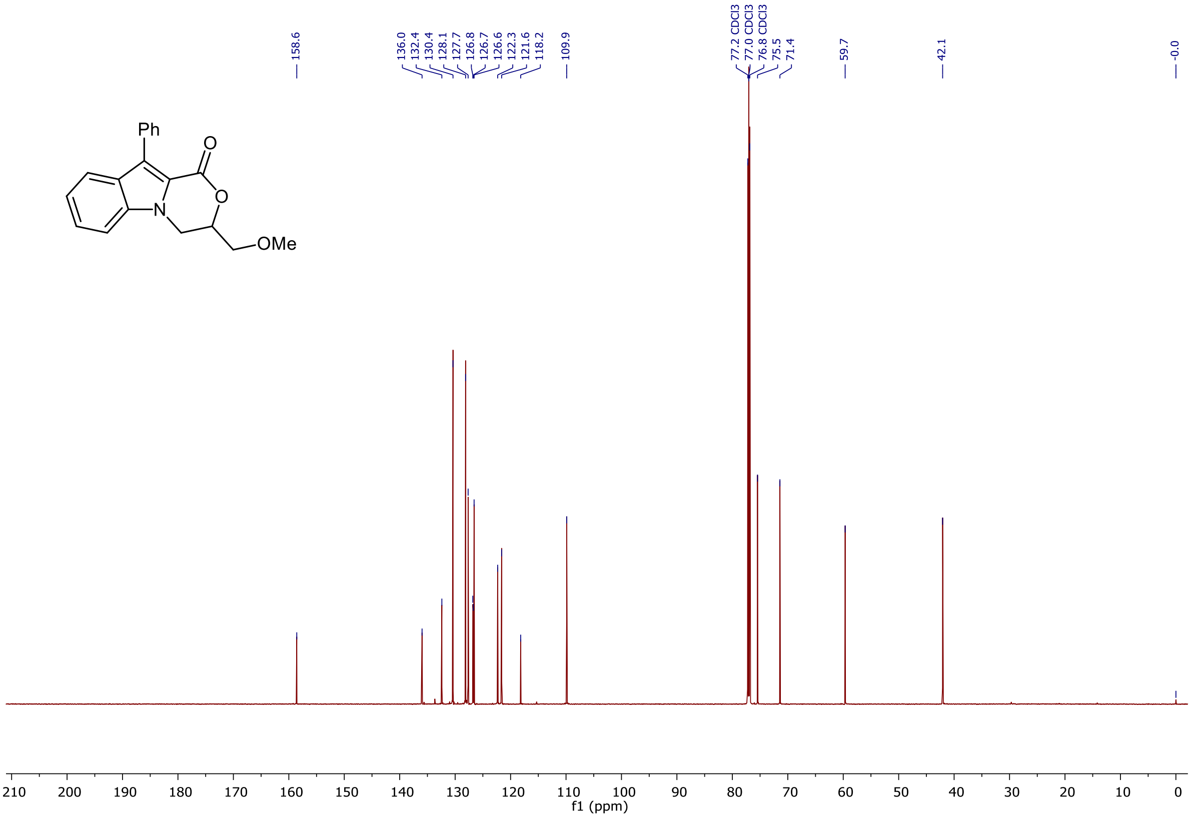

Figure S98. ^{13}C NMR (176 MHz, $CDCl_3$) spectrum of **12a**

Compound Spectrum SmartFormula Report

Analysis Info

Analysis Name	D:\Data\IZ-358.d	Acquisition Date	3/18/2025 6:15:24 PM
Method	DirectInfusion_TuneLow_pos.m	Operator	hplc
Sample Name	IZ-358	Instrument	micrOTOF-Q III 8228888.20448
Comment	AB		

Acquisition Parameter

Source Type	ESI	Ion Polarity	Positive	Set Nebulizer	0.4 Bar
Focus	Not active	Set Capillary	4500 V	Set Dry Heater	180 °C
Scan Begin	50 m/z	Set End Plate Offset	-500 V	Set Dry Gas	4.0 l/min
Scan End	1000 m/z	Set Collision Cell RF	140.0 Vpp	Set Divert Valve	Waste

#	RT [min]	Area	Int. Type	I	S/N	Chromatogram	Max. m/z	FWHM [min]
n.a.	4.7	n.a.	Single spectrum	n.a.	n.a.	n.a.	330.1100	n.a.

+MS, 4.7min #283

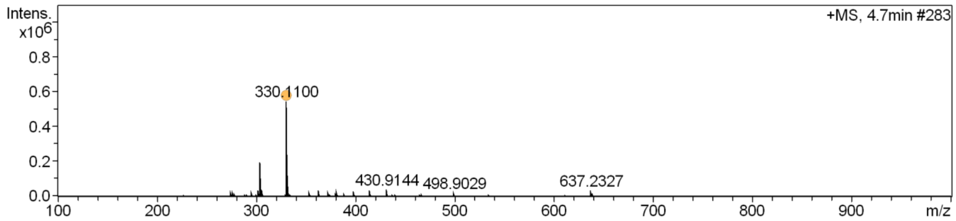

Meas. m/z	#	Ion Formula	m/z	err [ppm]	mSigma	# Sigma	Score	rdb	e⁻ Conf	N-Rule
330.1100	1	C19H17NNaO3	330.1101	-0.3	9.5	1	100.00	11.5	even	ok

Figure S99. HRMS (ESI) report of **12a**

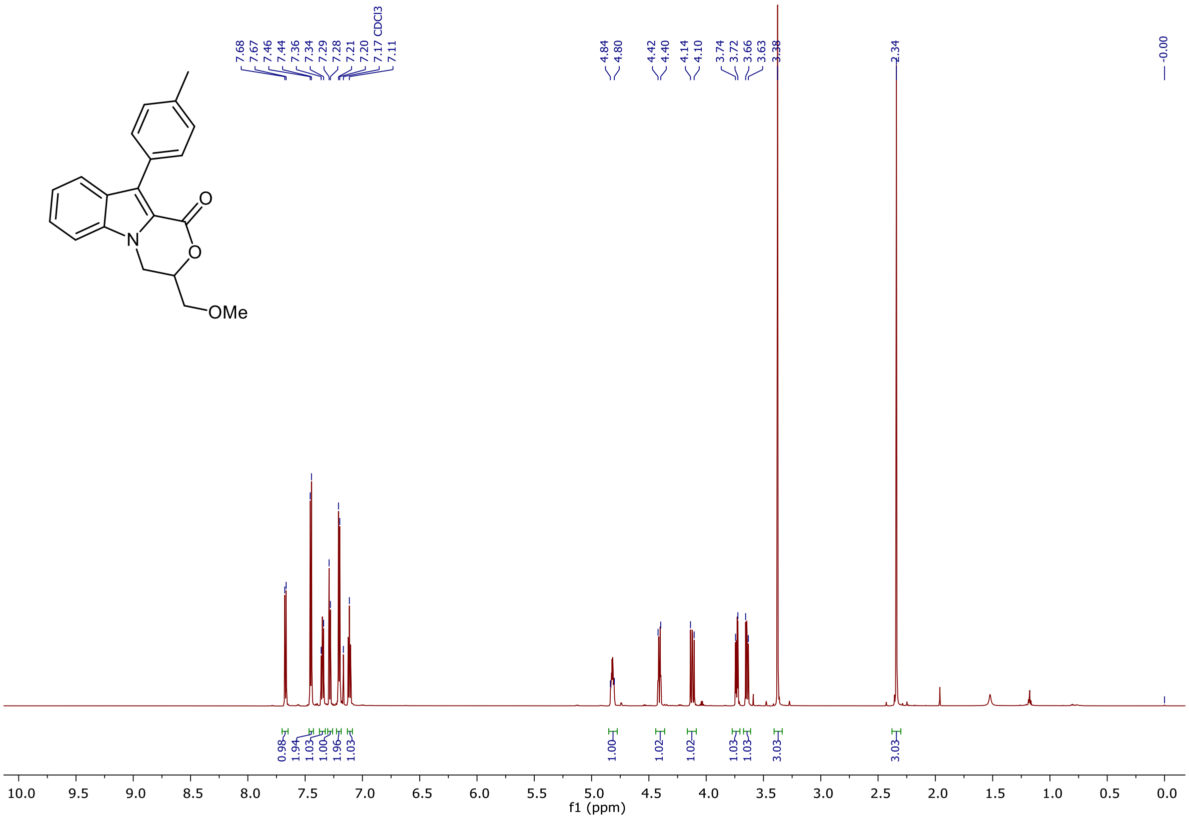

Figure S100. ^{1}H NMR (700 MHz, $CDCl_3$) spectrum of **12b**

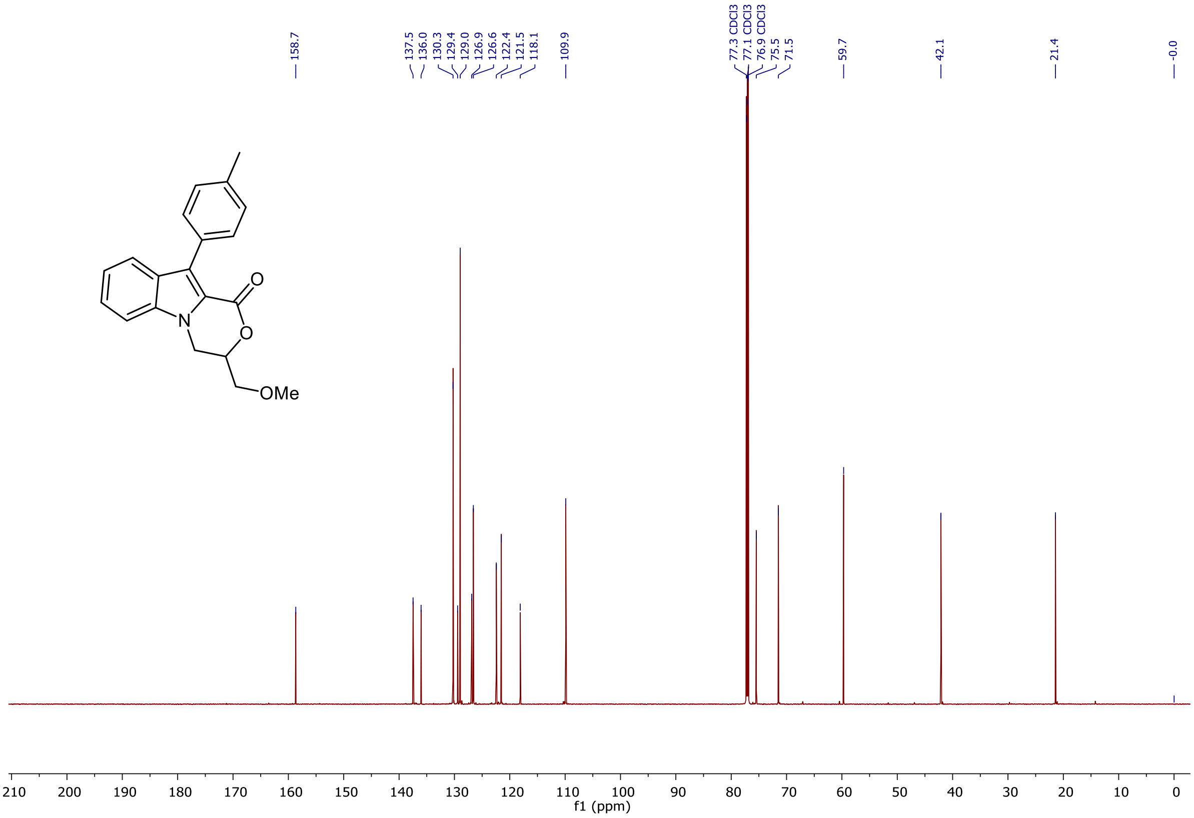

Figure S101. ^{13}C NMR (176 MHz, $CDCl_3$) spectrum of **12b**

Compound Spectrum SmartFormula Report

Analysis Info

Analysis Name	D:\Data\IZ-360.d	Acquisition Date	3/18/2025 6:48:20 PM
Method	DirectInfusion_TuneLow_pos.m	Operator	hplc
Sample Name	IZ-360	Instrument	micrOTOF-Q III 8228888.20448
Comment	AB		

Acquisition Parameter

Source Type	ESI	Ion Polarity	Positive	Set Nebulizer	0.4 Bar
Focus	Not active	Set Capillary	4500 V	Set Dry Heater	180 °C
Scan Begin	50 m/z	Set End Plate Offset	-500 V	Set Dry Gas	4.0 l/min
Scan End	1000 m/z	Set Collision Cell RF	140.0 Vpp	Set Divert Valve	Waste

#	RT [min]	Area	Int. Type	I	S/N	Chromatogram	Max. m/z	FWHM [min]
n.a.	4.4	n.a.	Single spectrum	n.a.	n.a.	n.a.	344.1262	n.a.

+MS, 4.4min #264

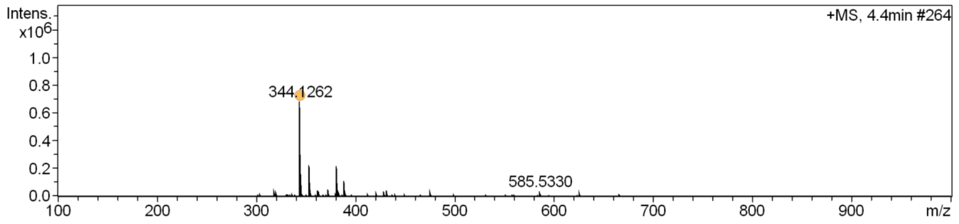

Meas. m/z	#	Ion Formula	m/z	err [ppm]	mSigma	# Sigma	Score	rdb	e⁻ Conf	N-Rule
344.1262	1	C20H19NNaO3	344.1257	-1.5	13.7	1	100.00	11.5	even	ok

Figure S102. HRMS (ESI) report of **12b**

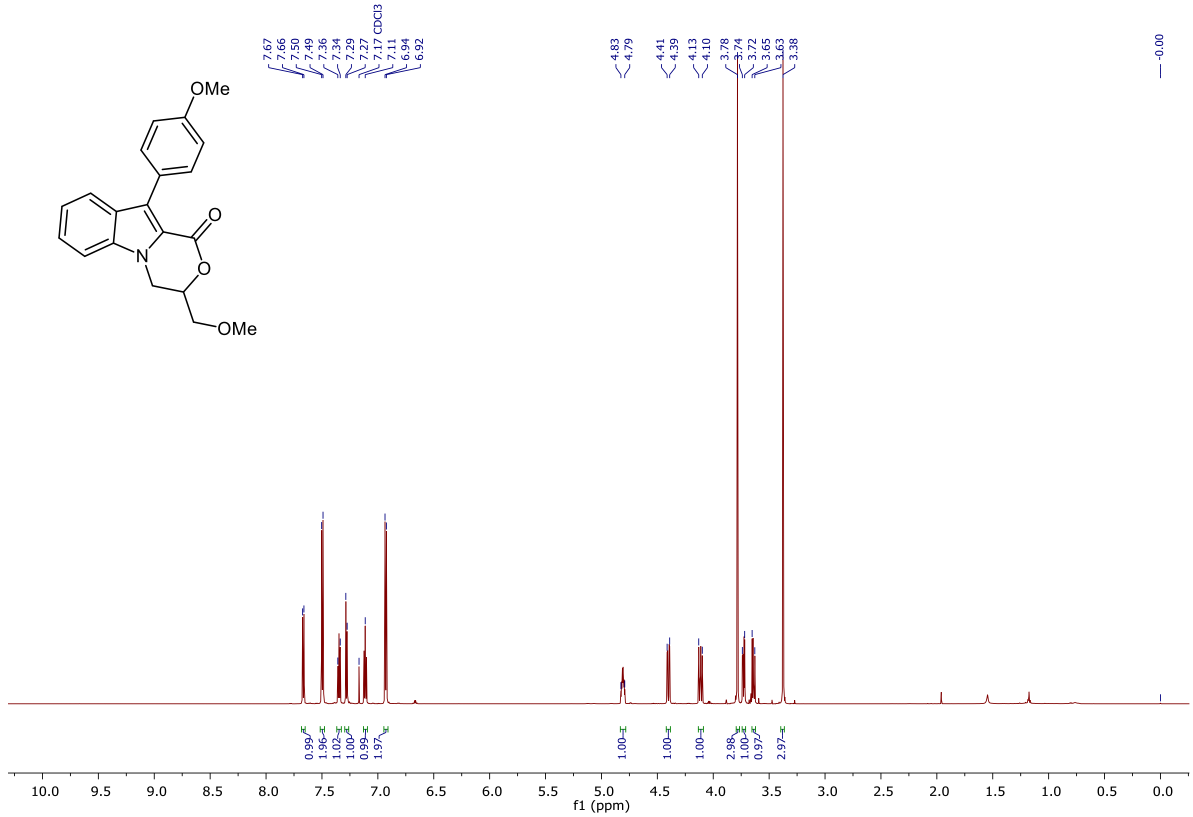

Figure S103. ^{1}H NMR (700 MHz, $CDCl_3$) spectrum of **12c**

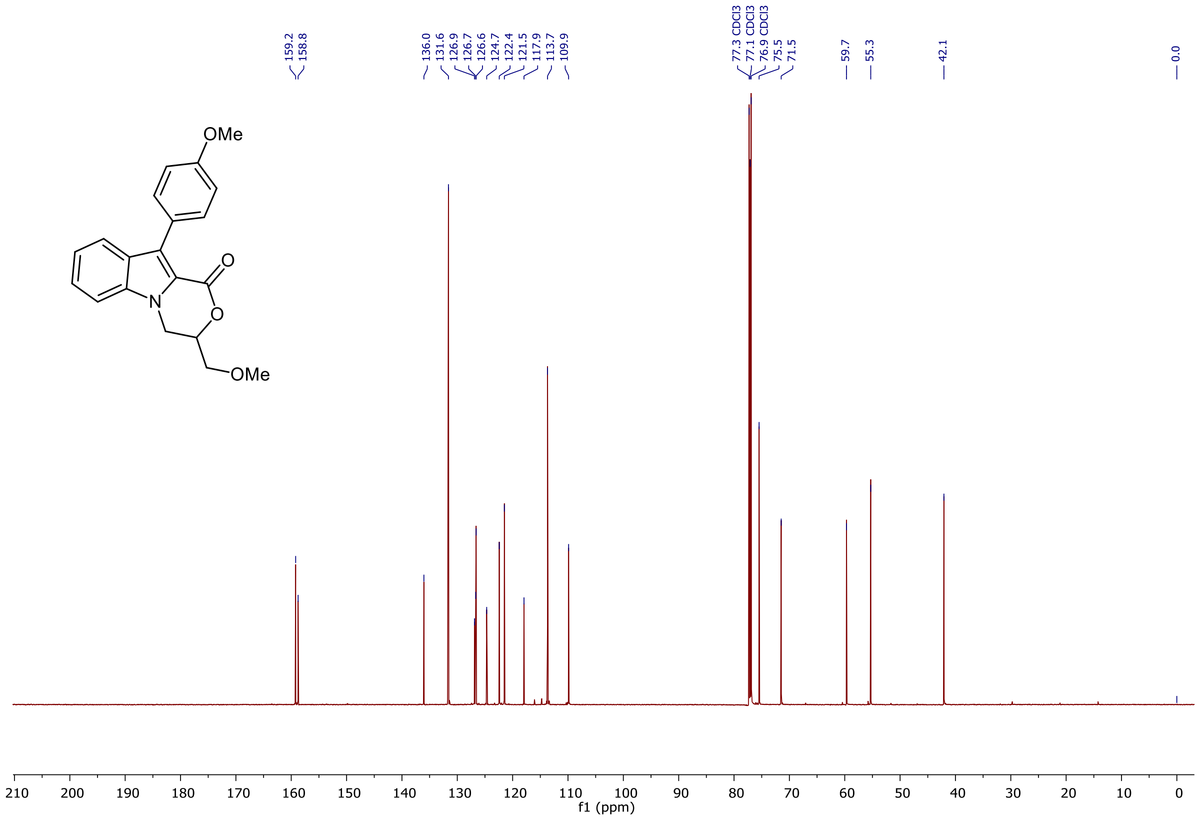

Figure S104. ^{13}C NMR (176 MHz, $CDCl_3$) spectrum of **12c**

Compound Spectrum SmartFormula Report

Analysis Info

Analysis Name	D:\Data\IZ-359.d	Acquisition Date	3/18/2025 6:33:42 PM
Method	DirectInfusion_TuneLow_pos.m	Operator	hplc
Sample Name	IZ-359	Instrument	micrOTOF-Q III 8228888.20448
Comment	AB		

Acquisition Parameter

Source Type	ESI	Ion Polarity	Positive	Set Nebulizer	0.4 Bar
Focus	Not active	Set Capillary	4500 V	Set Dry Heater	180 °C
Scan Begin	50 m/z	Set End Plate Offset	-500 V	Set Dry Gas	4.0 l/min
Scan End	1000 m/z	Set Collision Cell RF	140.0 Vpp	Set Divert Valve	Waste

#	RT [min]	Area	Int. Type	I	S/N	Chromatogram	Max. m/z	FWHM [min]
n.a.	5.6	n.a.	Single spectrum	n.a.	n.a.	n.a.	360.1209	n.a.

+MS, 5.6min #334

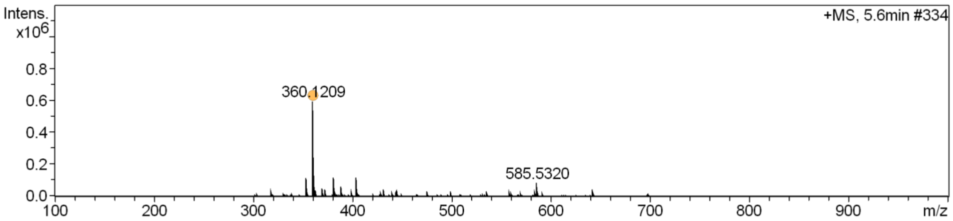

Meas. m/z	#	Ion Formula	m/z	err [ppm]	mSigma	# Sigma	Score	rdb	e^- Conf	N-Rule
360.1209	1	C20H19NNaO4	360.1206	0.7	8.4	2	100.00	11.5	even	ok

Figure S105. HRMS (ESI) report of **12c**

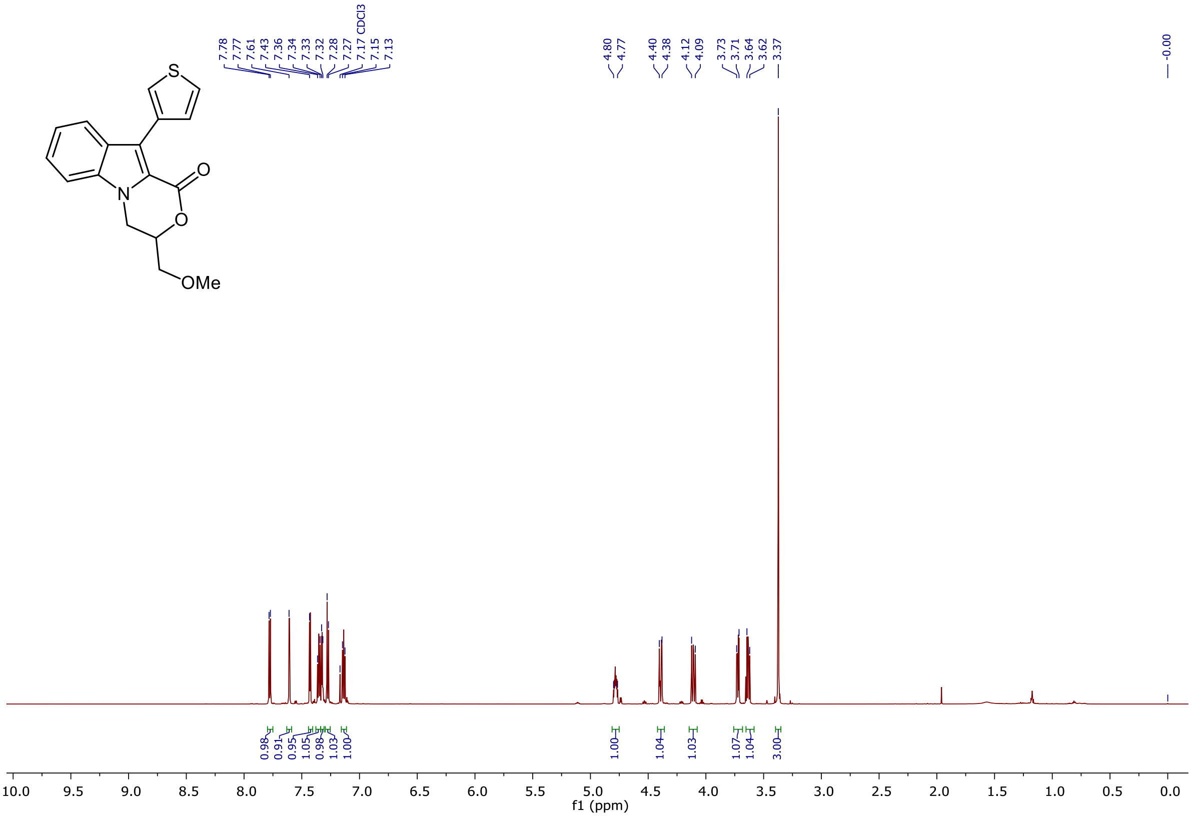

Figure S106. ^{1}H NMR (700 MHz, $CDCl_3$) spectrum of **12d**

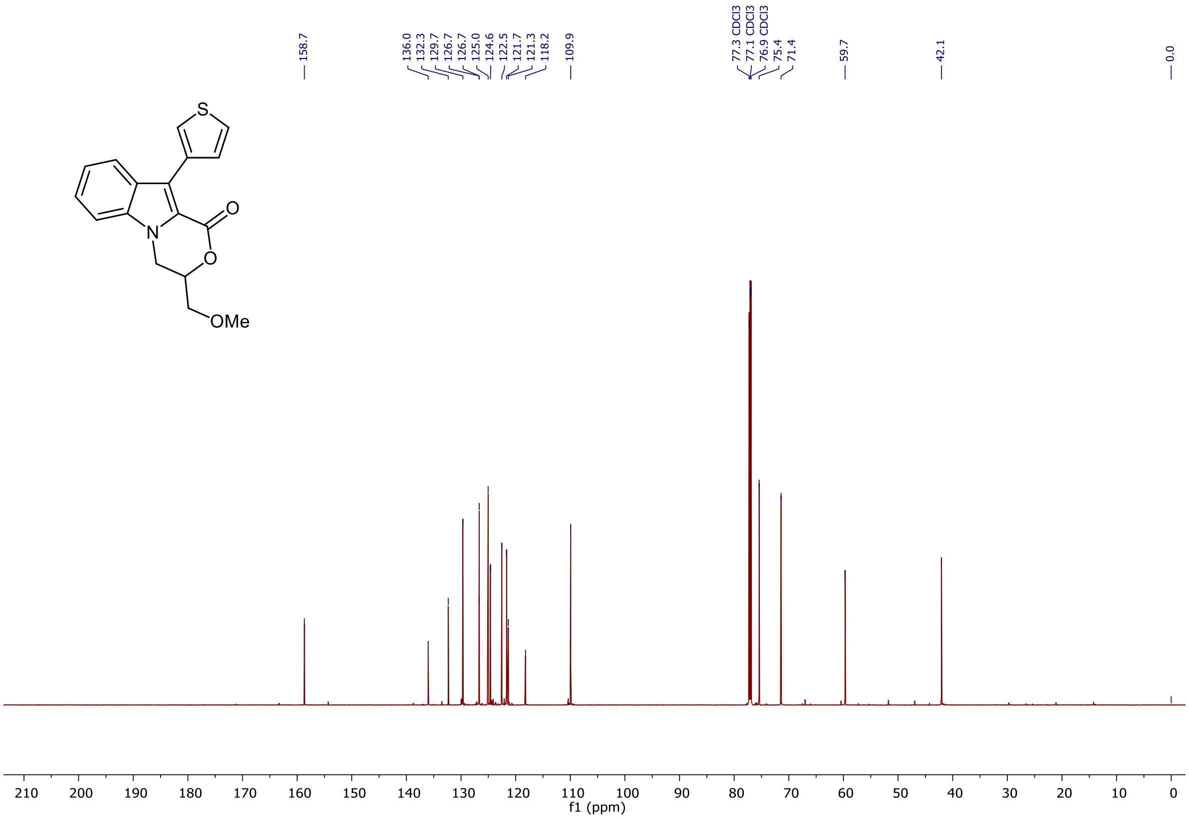

Figure S107. ^{13}C NMR (176 MHz, $CDCl_3$) spectrum of **12d**

Compound Spectrum SmartFormula Report

Analysis Info

Analysis Name	D:\Data\IZ-361.d	Acquisition Date	3/18/2025 7:04:09 PM
Method	DirectInfusion_TuneLow_pos.m	Operator	hplc
Sample Name	IZ-361	Instrument	micrOTOF-Q III 8228888.20448
Comment	AB		

Acquisition Parameter

Source Type	ESI	Ion Polarity	Positive	Set Nebulizer	0.4 Bar
Focus	Not active	Set Capillary	4500 V	Set Dry Heater	180 °C
Scan Begin	50 m/z	Set End Plate Offset	-500 V	Set Dry Gas	4.0 l/min
Scan End	1000 m/z	Set Collision Cell RF	140.0 Vpp	Set Divert Valve	Waste

#	RT [min]	Area	Int. Type	I	S/N	Chromatogram	Max. m/z	FWHM [min]
n.a.	3.5	n.a.	Single spectrum	n.a.	n.a.	n.a.	336.0663	n.a.

+MS, 3.5min #209

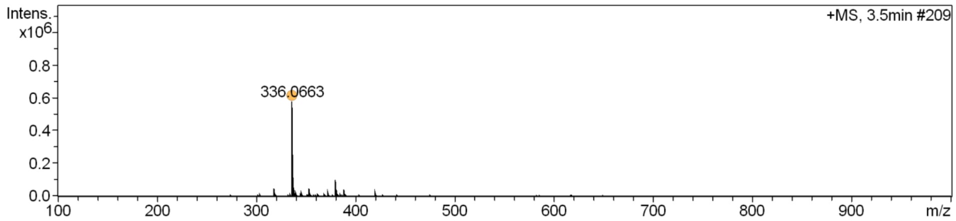

Meas. m/z	#	Ion Formula	m/z	err [ppm]	mSigma	# Sigma	Score	rdb	e⁻ Conf	N-Rule
336.0663	1	C17H15NNaO3S	336.0665	0.5	9.9	1	100.00	10.5	even	ok

Figure S108. HRMS (ESI) report of **12d**

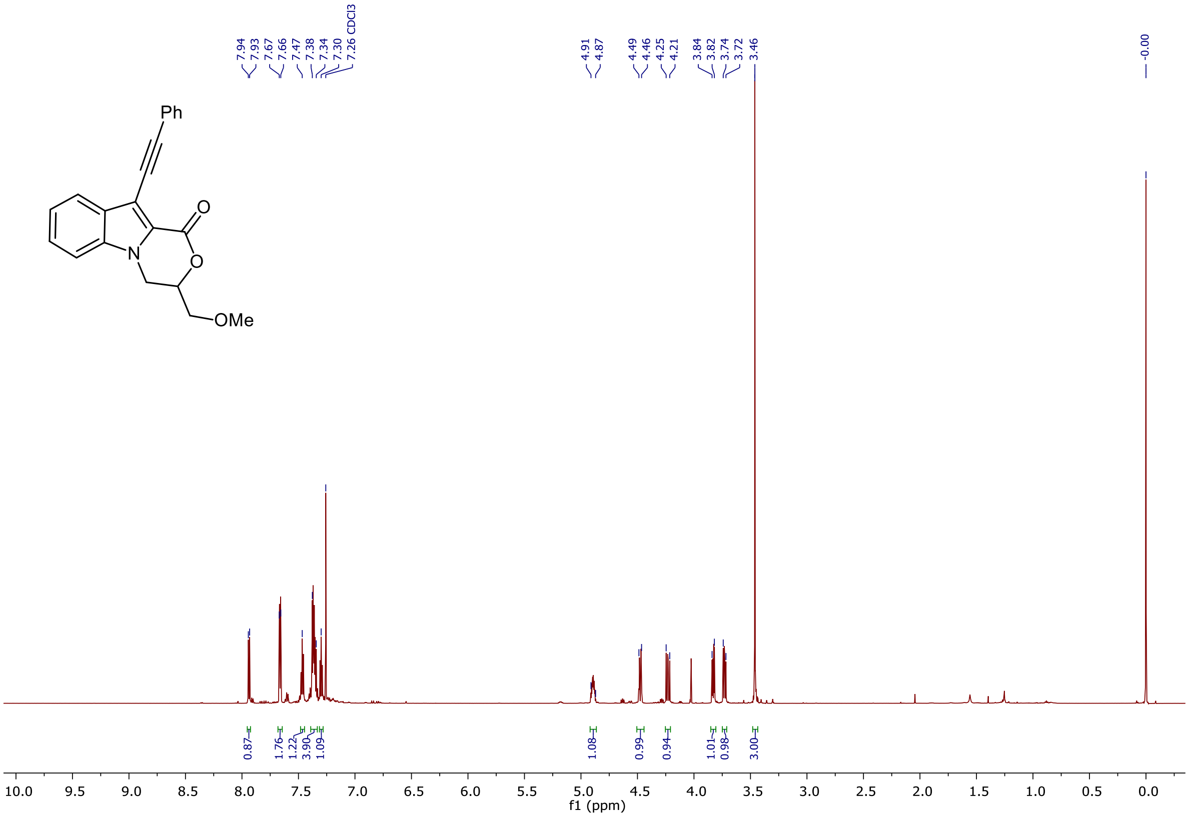

Figure S109. ^{1}H NMR (700 MHz, $CDCl_3$) spectrum of **13**

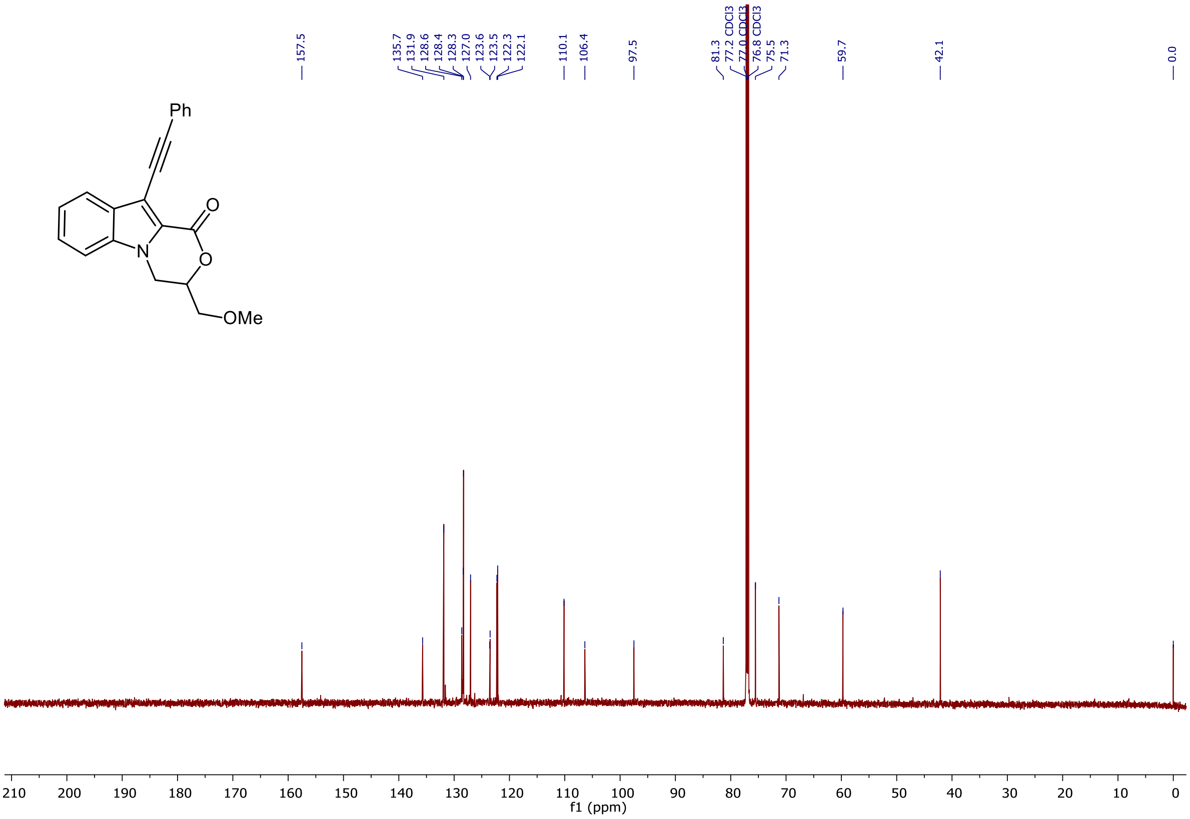

Figure S110. ^{13}C NMR (176 MHz, $CDCl_3$) spectrum of **13**

Compound Spectrum SmartFormula Report

Analysis Info

Analysis Name	D:\Data\IZ-368.d	Acquisition Date	3/19/2025 6:11:04 PM
Method	DirectInfusion_TuneLow_pos.m	Operator	hplc
Sample Name	IZ-368	Instrument	micrOTOF-Q III 8228888.20448
Comment	AB		

Acquisition Parameter

Source Type	ESI	Ion Polarity	Positive	Set Nebulizer	0.4 Bar
Focus	Not active	Set Capillary	4500 V	Set Dry Heater	180 °C
Scan Begin	50 m/z	Set End Plate Offset	-500 V	Set Dry Gas	4.0 l/min
Scan End	1000 m/z	Set Collision Cell RF	140.0 Vpp	Set Divert Valve	Waste

#	RT [min]	Area	Int. Type	I	S/N	Chromatogram	Max. m/z	FWHM [min]
n.a.	3.8	n.a.	Single spectrum	n.a.	n.a.	n.a.	354.1097	n.a.

+MS, 3.8min #228

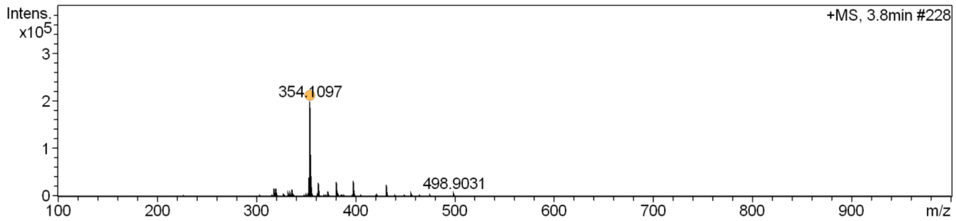

Meas. m/z	#	Ion Formula	m/z	err [ppm]	mSigma	# Sigma	Score	rdb	e⁻ Conf	N-Rule
354.1097	1	C21H17NNaO3	354.1101	-1.0	2.6	1	100.00	13.5	even	ok

Figure S111. HRMS (ESI) report of **13**

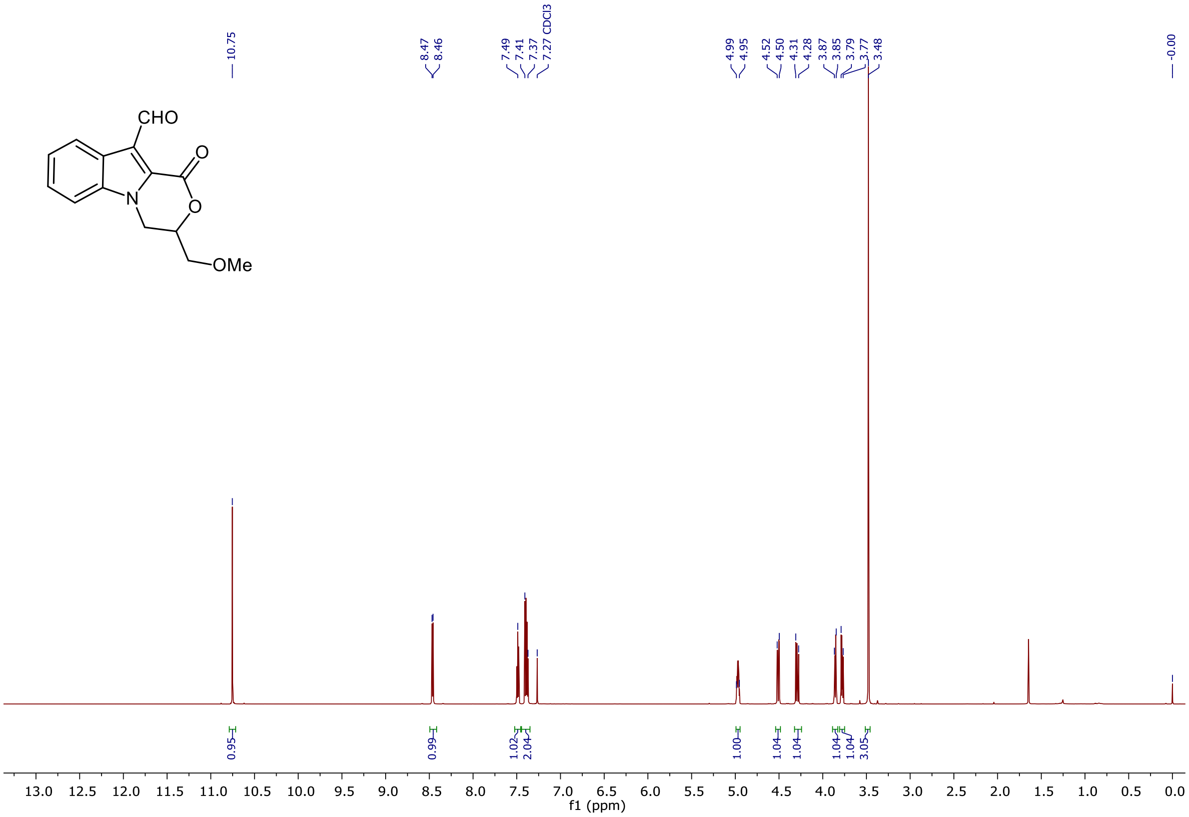

Figure S112. ^{1}H NMR (700 MHz, $CDCl_3$) spectrum of **14**

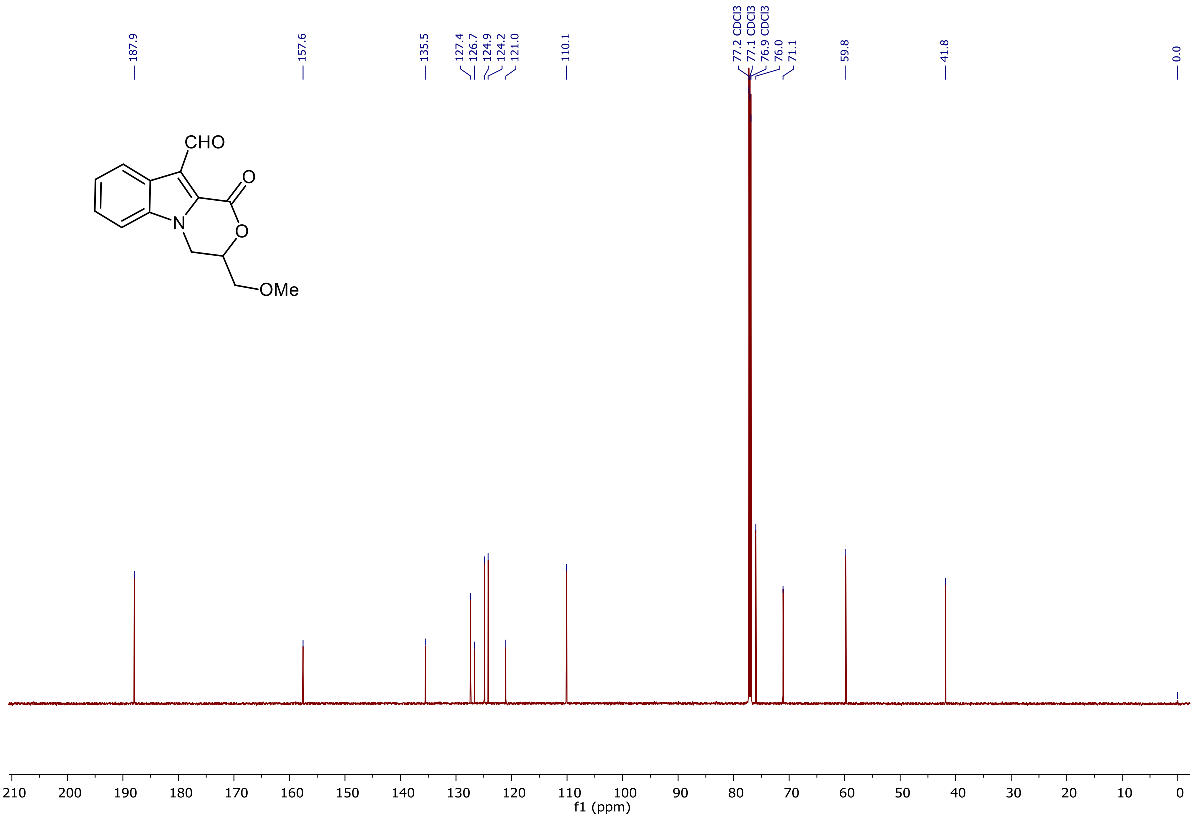

Figure S113. ^{13}C NMR (176 MHz, $CDCl_3$) spectrum of **14**

Compound Spectrum SmartFormula Report

Analysis Info

Analysis Name	D:\Data\IZ-362-new.d	Acquisition Date	3/18/2025 7:23:40 PM
Method	DirectInfusion_TuneLow_pos.m	Operator	hplc
Sample Name	IZ-362-new	Instrument	micrOTOF-Q III 8228888.20448
Comment	AB		

Acquisition Parameter

Source Type	ESI	Ion Polarity	Positive	Set Nebulizer	0.4 Bar
Focus	Not active	Set Capillary	4500 V	Set Dry Heater	180 °C
Scan Begin	50 m/z	Set End Plate Offset	-500 V	Set Dry Gas	4.0 l/min
Scan End	1000 m/z	Set Collision Cell RF	140.0 Vpp	Set Divert Valve	Waste

#	RT [min]	Area	Int. Type	I	S/N	Chromatogram	Max. m/z	FWHM [min]
n.a.	3.0	n.a.	Single spectrum	n.a.	n.a.	n.a.	282.0734	n.a.

+MS, 3.0min #182

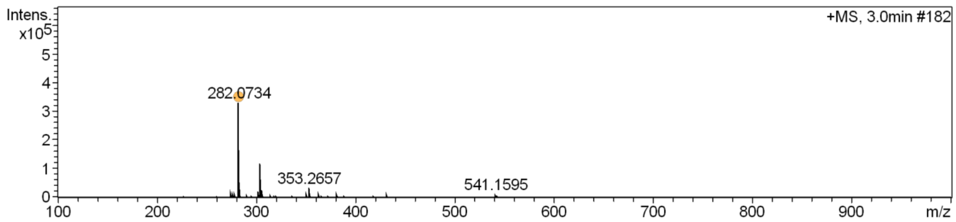

Meas. m/z	#	Ion Formula	m/z	err [ppm]	mSigma	# Sigma	Score	rdb	e⁻ Conf	N-Rule
282.0734	1	C14H13NNaO4	282.0737	0.9	1.8	1	100.00	8.5	even	ok

Figure S114. HRMS (ESI) report of **14**

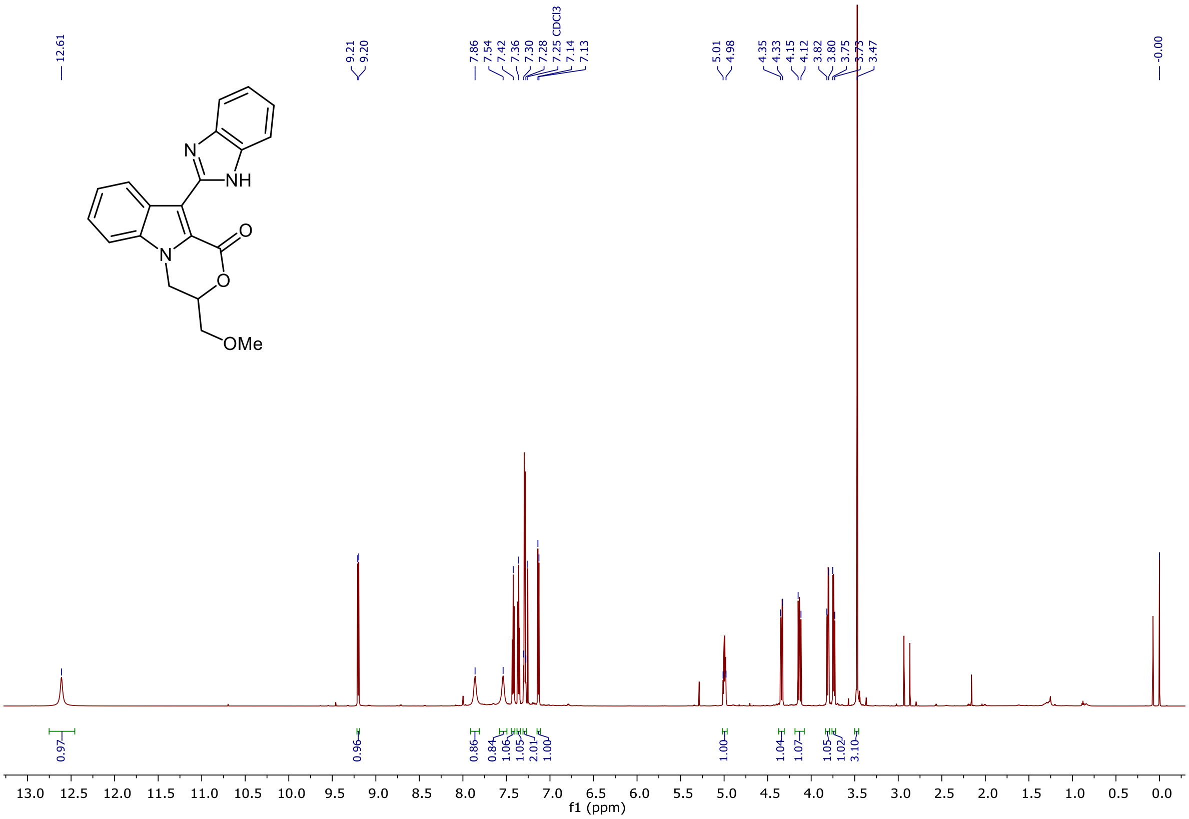

Figure S115. ^{1}H NMR (700 MHz, $CDCl_3$) spectrum of **15a**

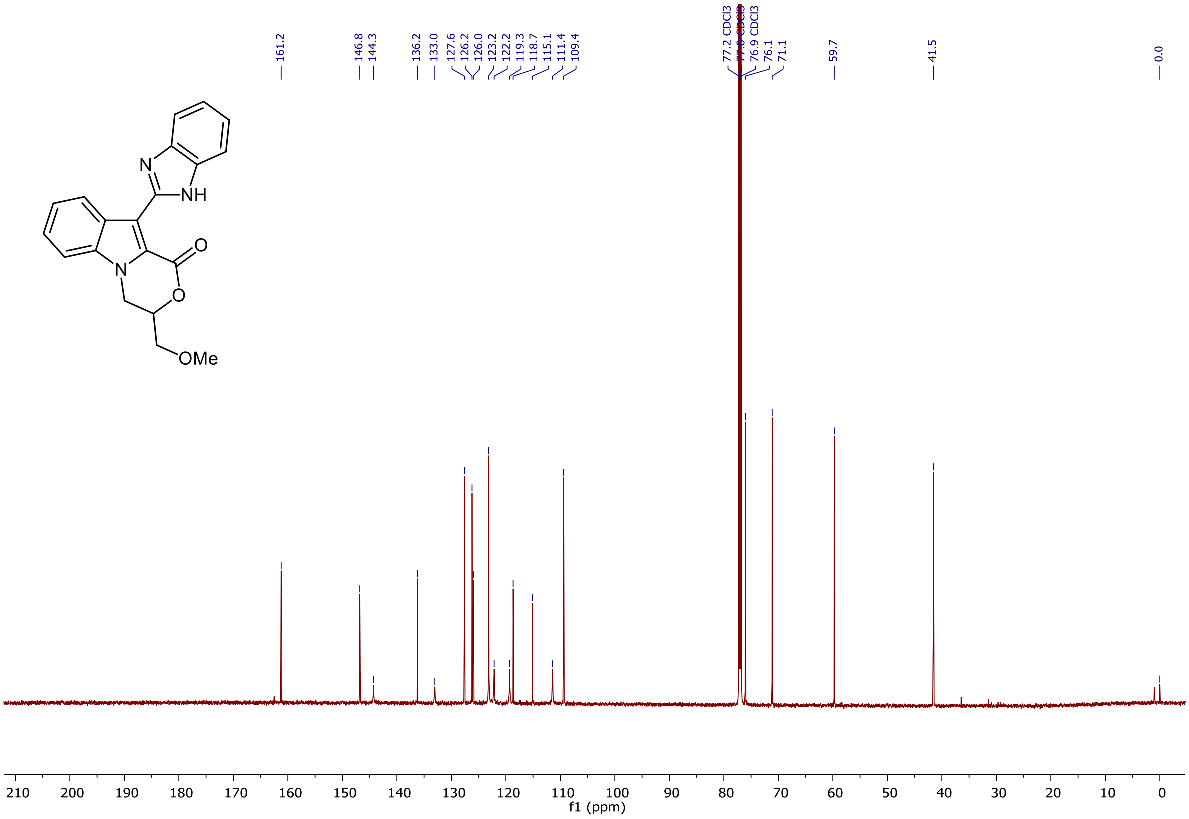

Figure S116. ^{13}C NMR (176 MHz, $CDCl_3$) spectrum of **15a**

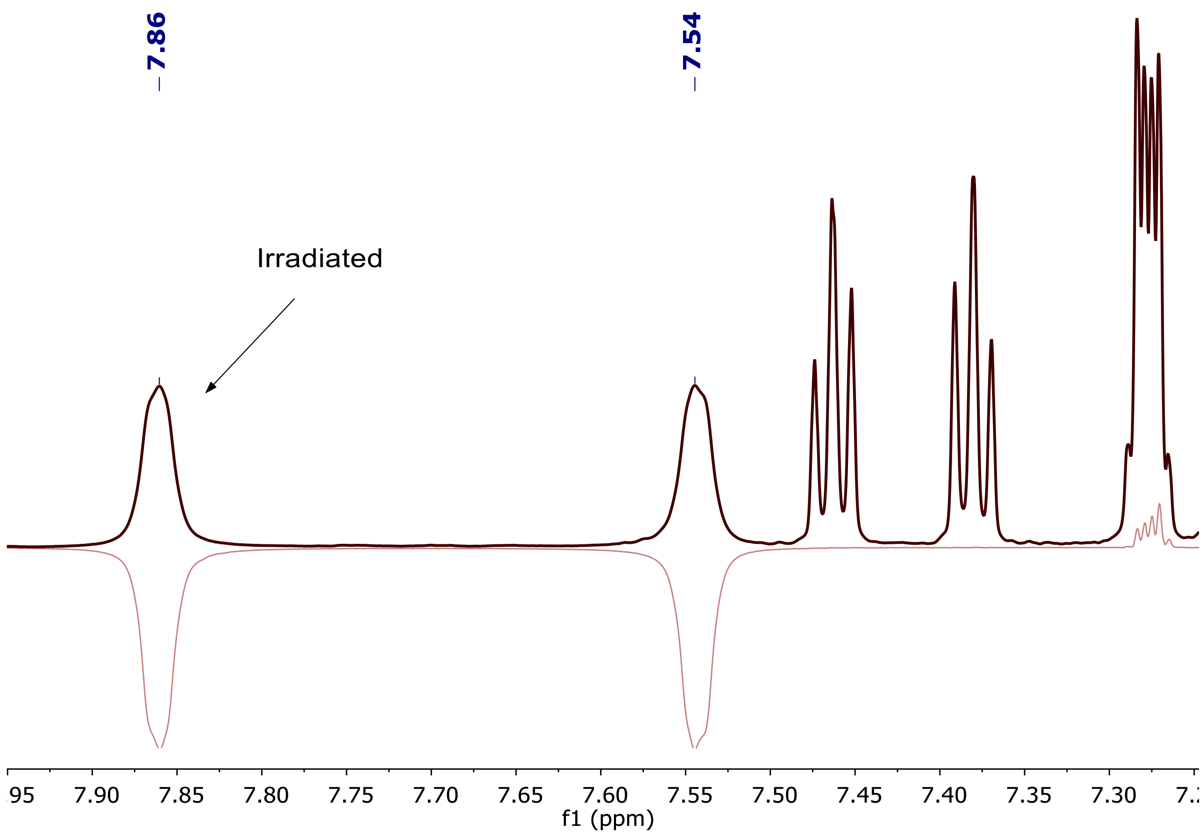

Figure S117. Superimposed ^{1}H NMR and 1D gradient NOE spectra of tautomer **15a** with a selective irradiation of signal at δ 7.86 ppm

Compound Spectrum SmartFormula Report

Analysis Info

Analysis Name	D:\Data\IZ-367.d	Acquisition Date	3/19/2025 5:54:32 PM
Method	DirectInfusion_TuneLow_pos.m	Operator	hplc
Sample Name	IZ-367	Instrument	micrOTOF-Q III 8228888.20448
Comment	AB		

Acquisition Parameter

Source Type	ESI	Ion Polarity	Positive	Set Nebulizer	0.4 Bar
Focus	Not active	Set Capillary	4500 V	Set Dry Heater	180 °C
Scan Begin	50 m/z	Set End Plate Offset	-500 V	Set Dry Gas	4.0 l/min
Scan End	1000 m/z	Set Collision Cell RF	140.0 Vpp	Set Divert Valve	Waste

#	RT [min]	Area	Int. Type	I	S/N	Chromatogram	Max. m/z	FWHM [min]
n.a.	3.2	n.a.	Single spectrum	n.a.	n.a.	n.a.	348.1345	n.a.

+MS, 3.2min #193

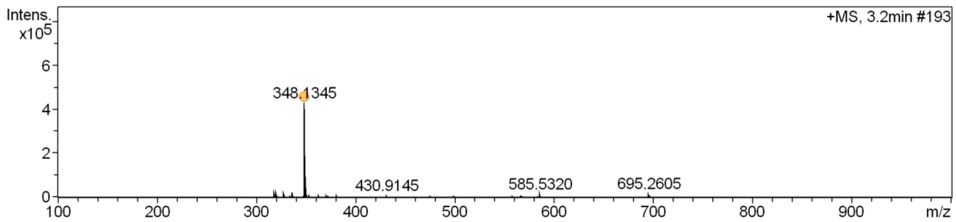

Meas. m/z	#	Ion Formula	m/z	err [ppm]	mSigma	# Sigma	Score	rdb	e⁻ Conf	N-Rule
348.1345	1	C20H18N3O3	348.1343	-0.7	1.6	1	100.00	13.5	even	ok

Figure S118. HRMS (ESI) report of **15a**

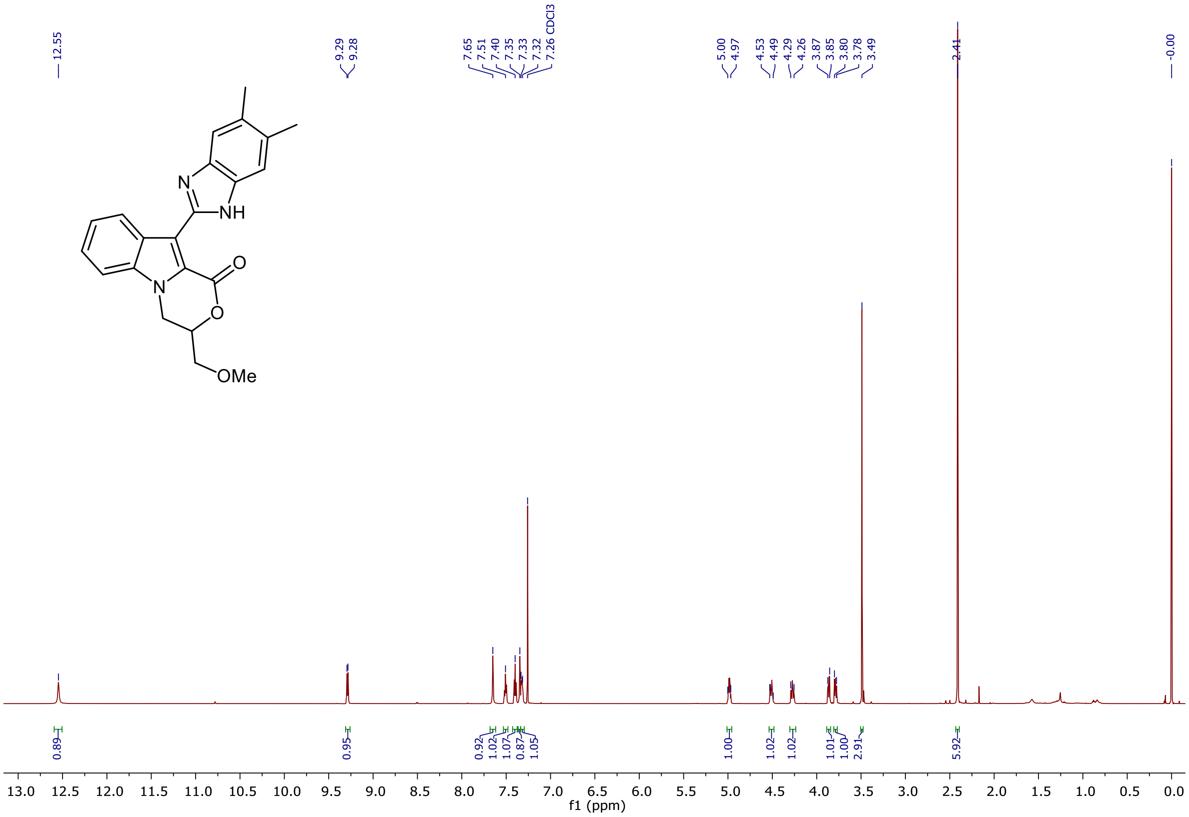

Figure S119. ^{1}H NMR (700 MHz, $CDCl_3$) spectrum of **15b**

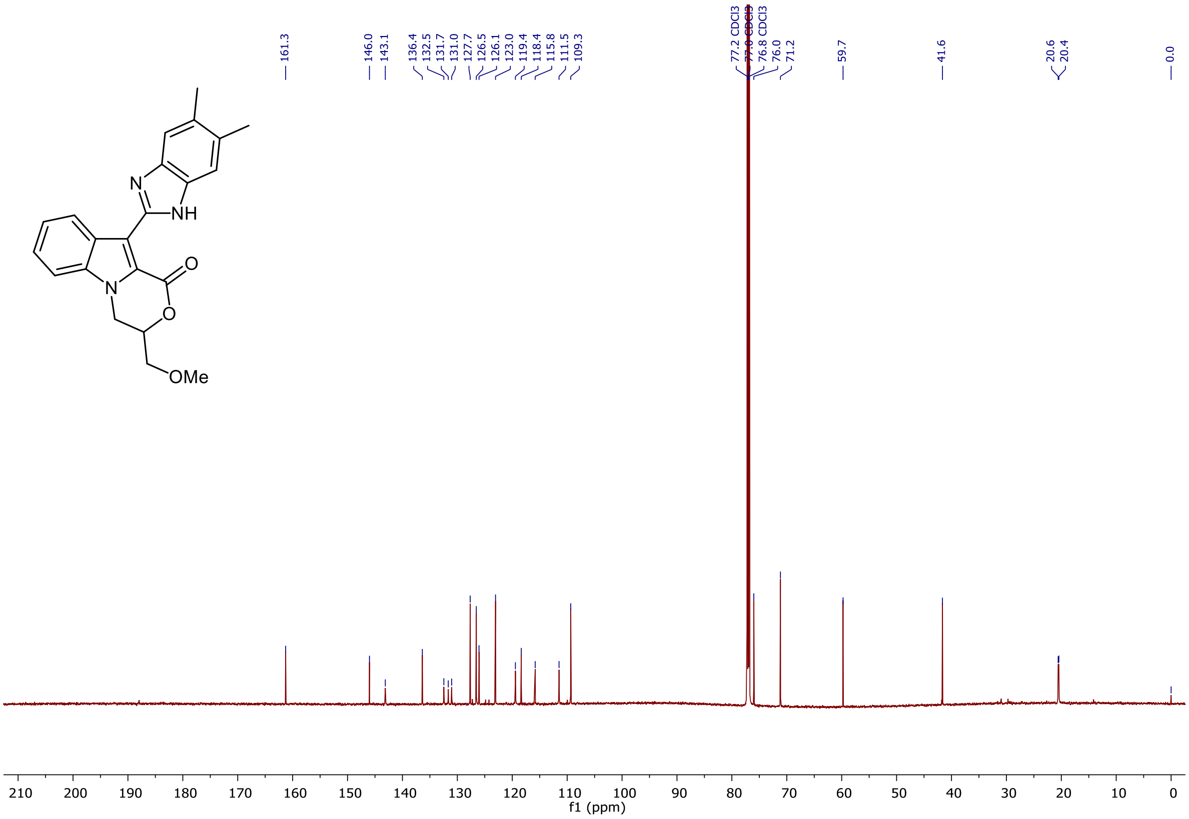

Figure S120. ^{13}C NMR (176 MHz, $CDCl_3$) spectrum of **15b**

Compound Spectrum SmartFormula Report

Analysis Info

Analysis Name	D:\Data\IZ-372-2_po_BMR.d	Acquisition Date	3/19/2025 6:24:03 PM
Method	DirectInfusion_TuneLow_pos.m	Operator	hplc
Sample Name	IZ-372-2_po_BMR	Instrument	micrOTOF-Q III 8228888.20448
Comment	AB		

Acquisition Parameter

Source Type	ESI	Ion Polarity	Positive	Set Nebulizer	0.4 Bar
Focus	Not active	Set Capillary	4500 V	Set Dry Heater	180 °C
Scan Begin	50 m/z	Set End Plate Offset	-500 V	Set Dry Gas	4.0 l/min
Scan End	1000 m/z	Set Collision Cell RF	140.0 Vpp	Set Divert Valve	Waste

#	RT [min]	Area	Int. Type	I	S/N	Chromatogram	Max. m/z	FWHM [min]
n.a.	4.9	n.a.	Single spectrum	n.a.	n.a.	n.a.	376.1653	n.a.

+MS, 4.9min #295

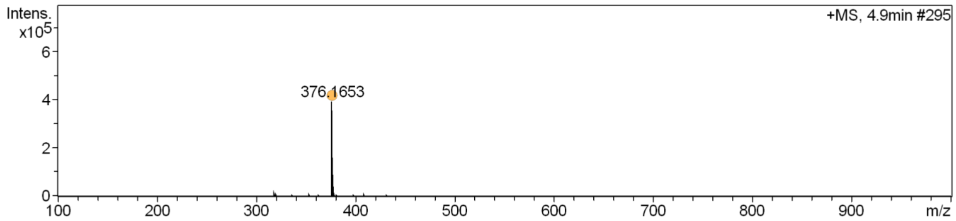

Meas. m/z	#	Ion Formula	m/z	err [ppm]	mSigma	# Sigma	Score	rdb	e⁻ Conf	N-Rule
376.1653	1	C22H22N3O3	376.1656	-0.6	0.7	1	100.00	13.5	even	ok

Figure S121. HRMS (ESI) report of **15b**

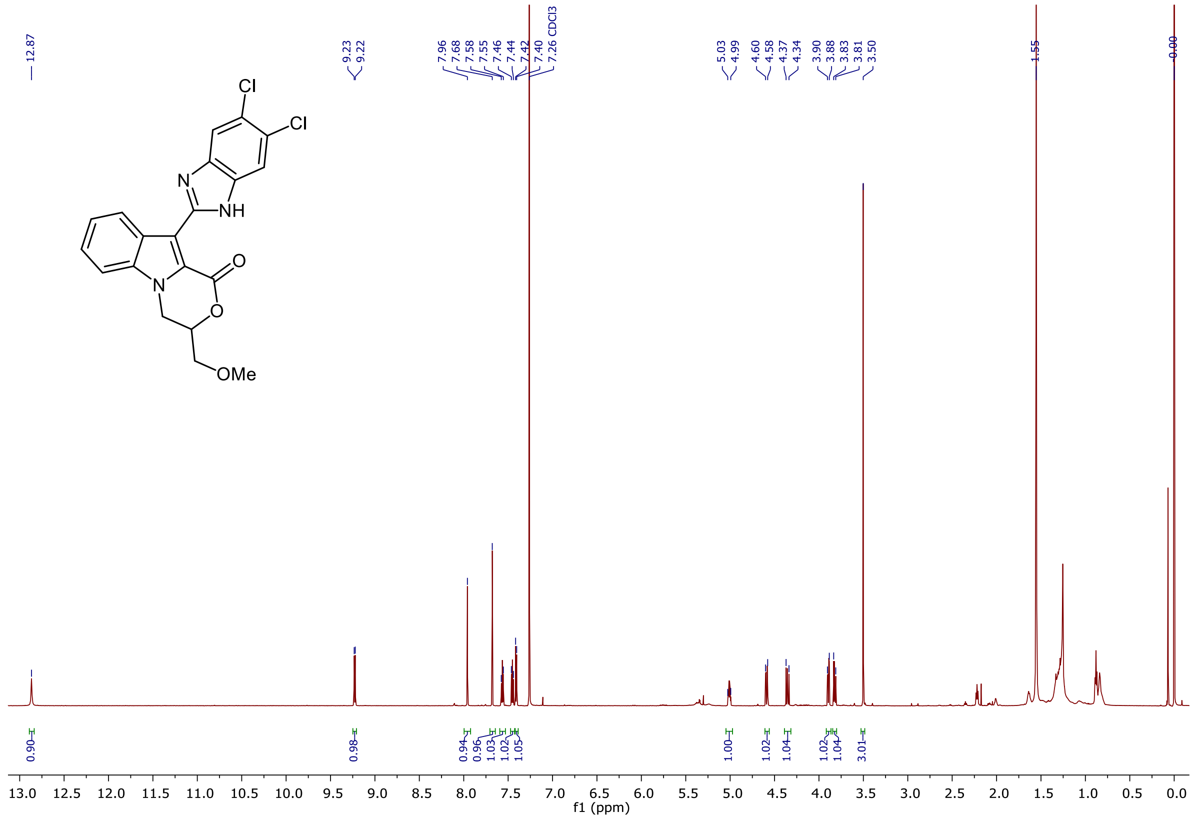

Figure S122. ^{1}H NMR (700 MHz, $CDCl_3$) spectrum of **15c**

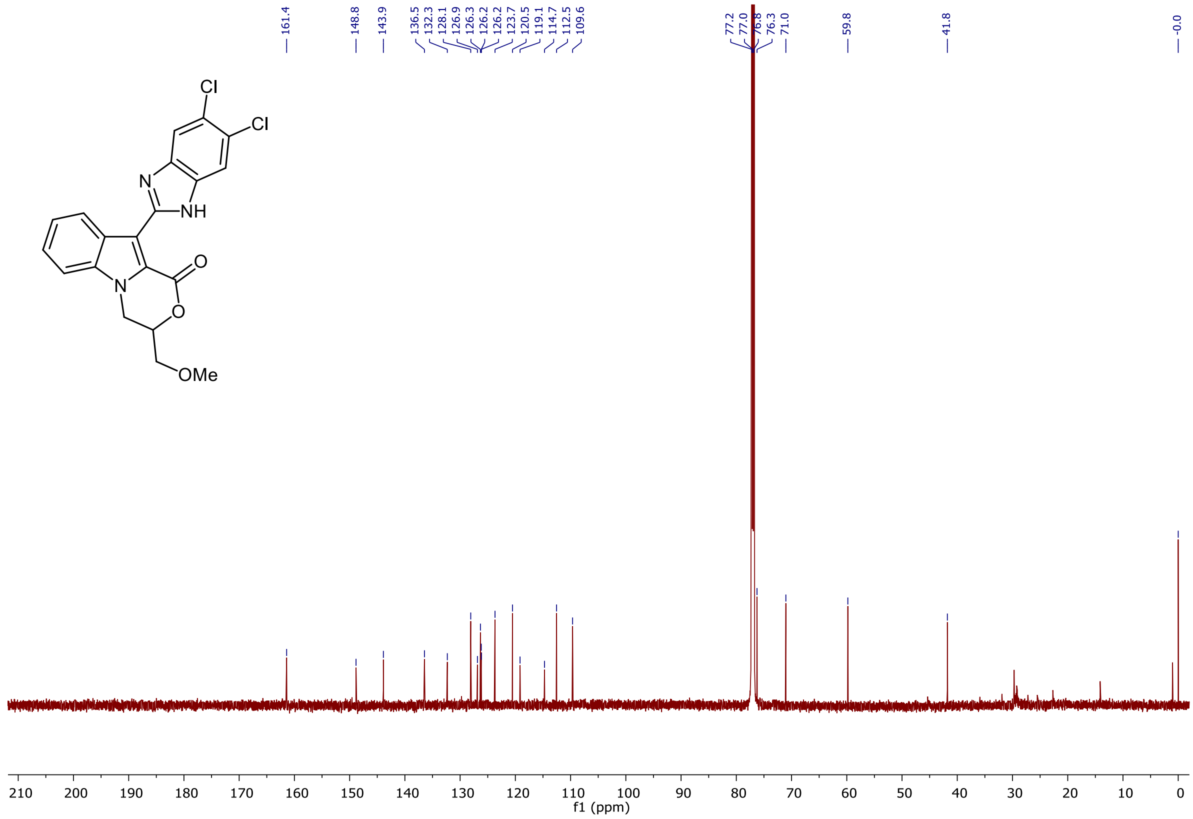

Figure S123. ^{13}C NMR (176 MHz, $CDCl_3$) spectrum of **15c**

Compound Spectrum SmartFormula Report

Analysis Info

Analysis Name	D:\Data\IZ-374_chcl3_meoh.d	Acquisition Date	3/19/2025 7:35:28 PM
Method	DirectInfusion_TuneLow_pos.m	Operator	hplc
Sample Name	IZ-374_chcl3_meoh	Instrument	micrOTOF-Q III 8228888.20448
Comment	AB		

Acquisition Parameter

Source Type	ESI	Ion Polarity	Positive	Set Nebulizer	0.4 Bar
Focus	Not active	Set Capillary	4500 V	Set Dry Heater	180 °C
Scan Begin	50 m/z	Set End Plate Offset	-500 V	Set Dry Gas	4.0 l/min
Scan End	1000 m/z	Set Collision Cell RF	140.0 Vpp	Set Divert Valve	Waste

#	RT [min]	Area	Int. Type	I	S/N	Chromatogram	Max. m/z	FWHM [min]
n.a.	17.0	n.a.	Single spectrum	n.a.	n.a.	n.a.	496.3618	n.a.

+MS, 17.0min #1020

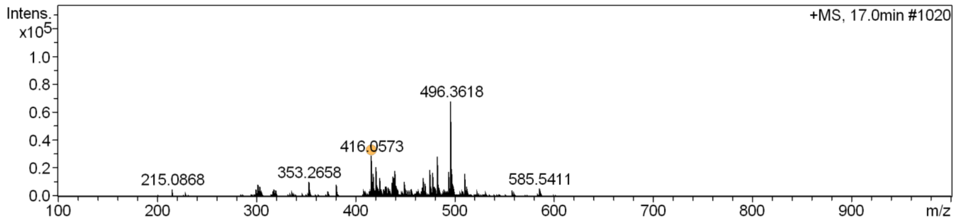

Meas. m/z	#	Ion Formula	m/z	err [ppm]	mSigma	# Sigma	Score	rdb	e⁻ Conf	N-Rule
416.0573	1	C20H16Cl2N3O3	416.0563	-2.3	52.1	1	100.00	13.5	even	ok

Figure S124. HRMS (ESI) report of **15c**

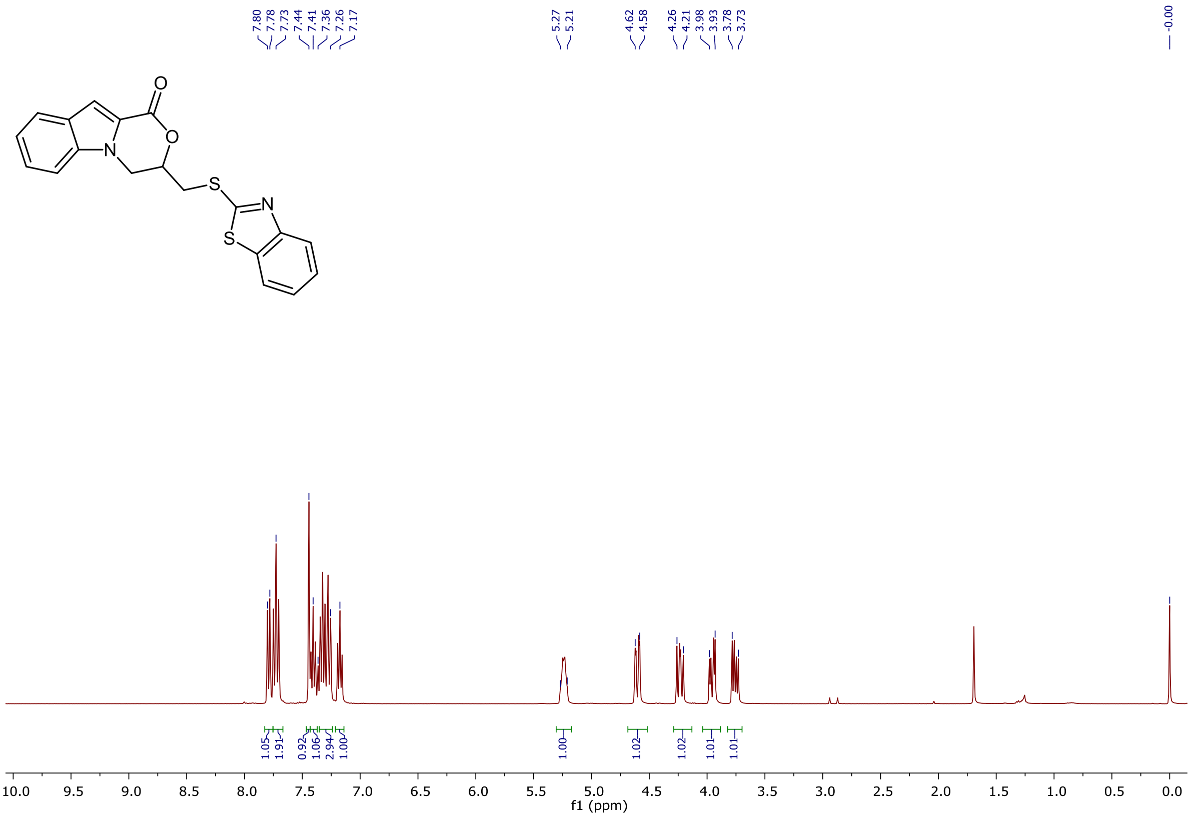

Figure S125. ^1H NMR (400 MHz, $CDCl_3$) spectrum of **16a**

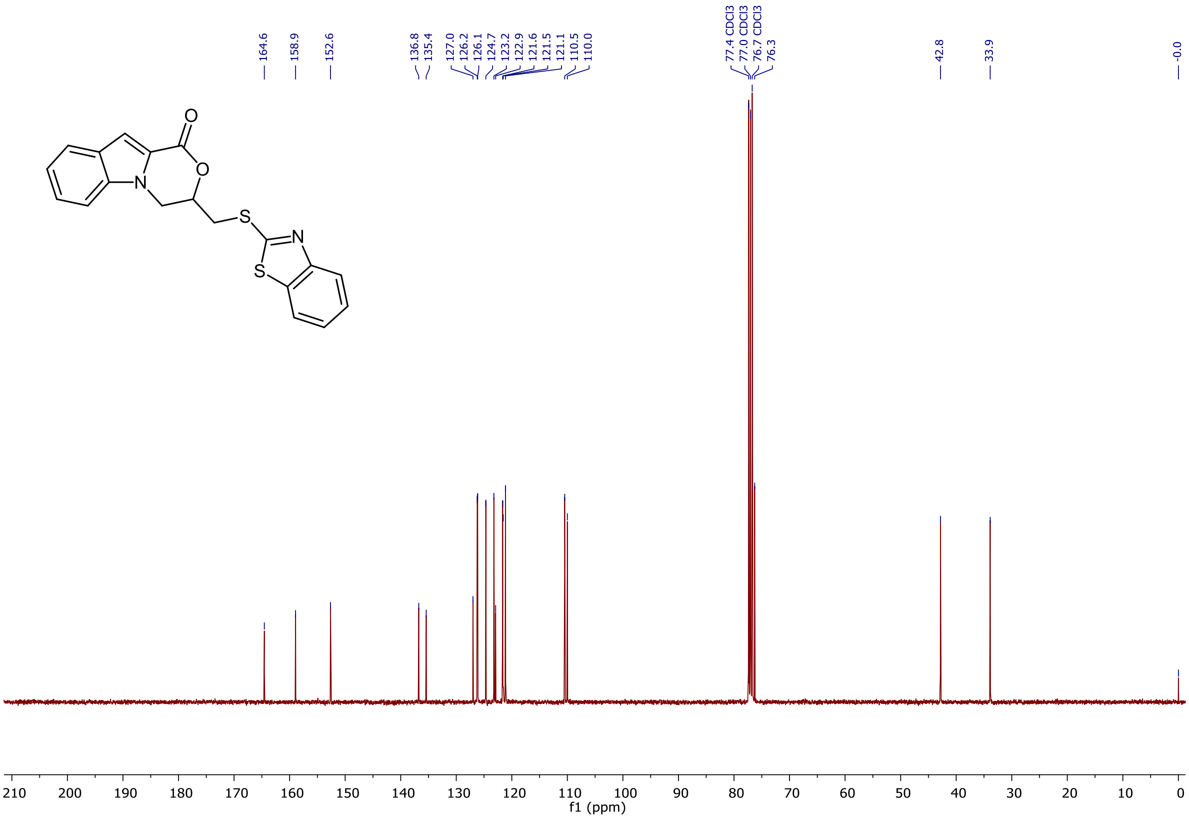

Figure S126. ^{13}C NMR (101 MHz, $CDCl_3$) spectrum of **16a**

Compound Spectrum SmartFormula Report

Analysis Info

Analysis Name	D:\Data\IZ-335.d	Acquisition Date	12/18/2024 11:00:56 AM
Method	DirectInfusion_TuneLow_pos.m	Operator	hplc
Sample Name	IZ-335	Instrument	micrOTOF-Q III 8228888.20448
Comment	SB		

Acquisition Parameter

Source Type	ESI	Ion Polarity	Positive	Set Nebulizer	0.4 Bar
Focus	Not active	Set Capillary	4500 V	Set Dry Heater	180 °C
Scan Begin	50 m/z	Set End Plate Offset	-500 V	Set Dry Gas	4.0 l/min
Scan End	1000 m/z	Set Collision Cell RF	140.0 Vpp	Set Divert Valve	Waste

#	RT [min]	Area	Int. Type	I	S/N	Chromatogram	Max. m/z	FWHM [min]
n.a.	3.3	n.a.	Single spectrum	n.a.	n.a.	n.a.	304.2626	n.a.
n.a.	5.0	n.a.	Single spectrum	n.a.	n.a.	n.a.	389.0389	n.a.

+MS, 5.0min #297

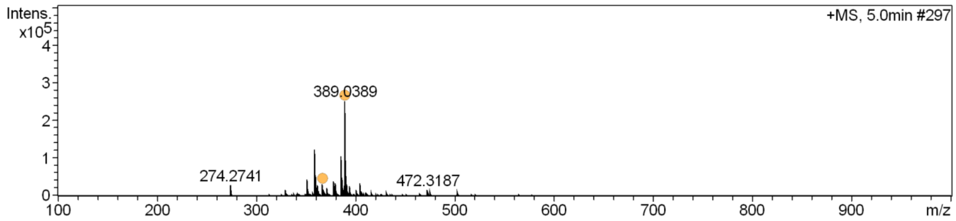

Meas. m/z	#	Ion Formula	m/z	err [ppm]	mSigma	# Sigma	Score	rdb	e⁻ Conf	N-Rule
367.0564	1	C19H15N2O2S2	367.0569	1.4	28.2	1	100.00	13.5	even	ok
389.0389	1	C19H14N2NaO2S2	389.0389	0.0	7.0	1	100.00	13.5	even	ok

Figure S127. HRMS (ESI) report of **16a**

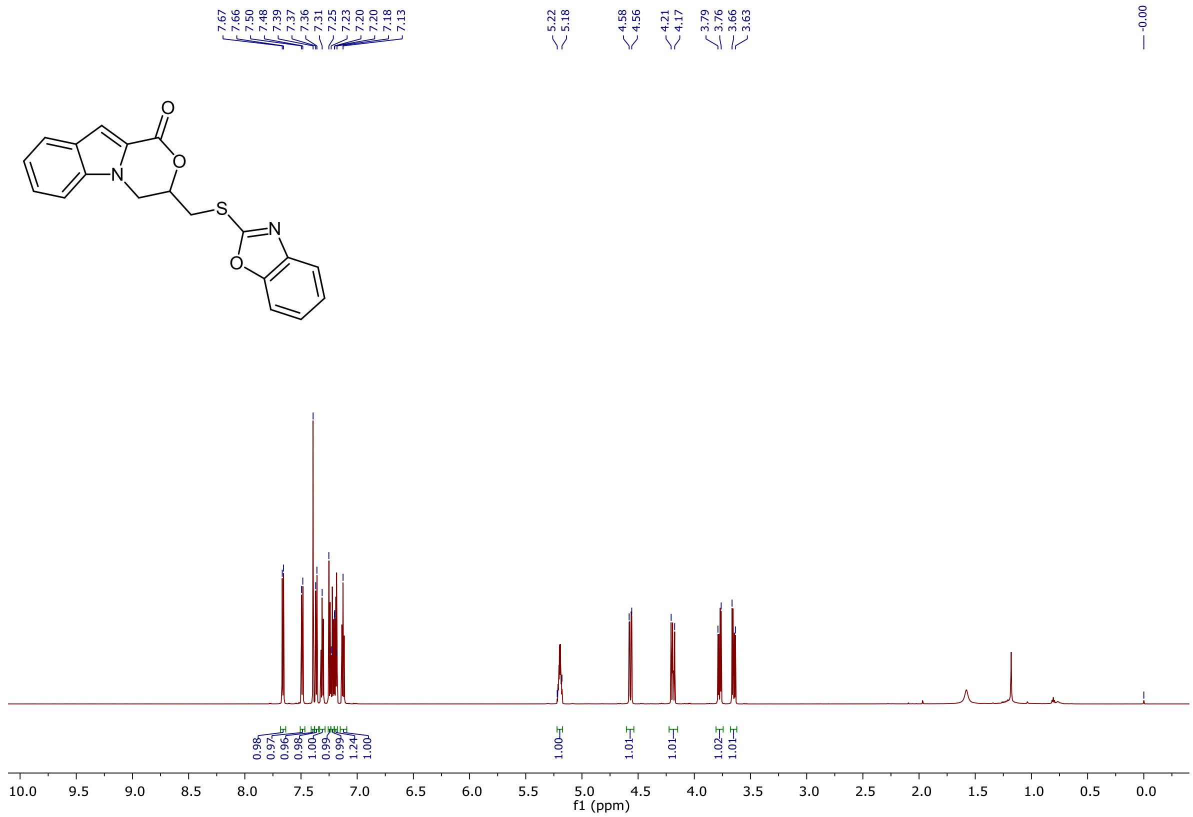

Figure S128. ^{1}H NMR (700 MHz, $CDCl_3$) spectrum of **16b**

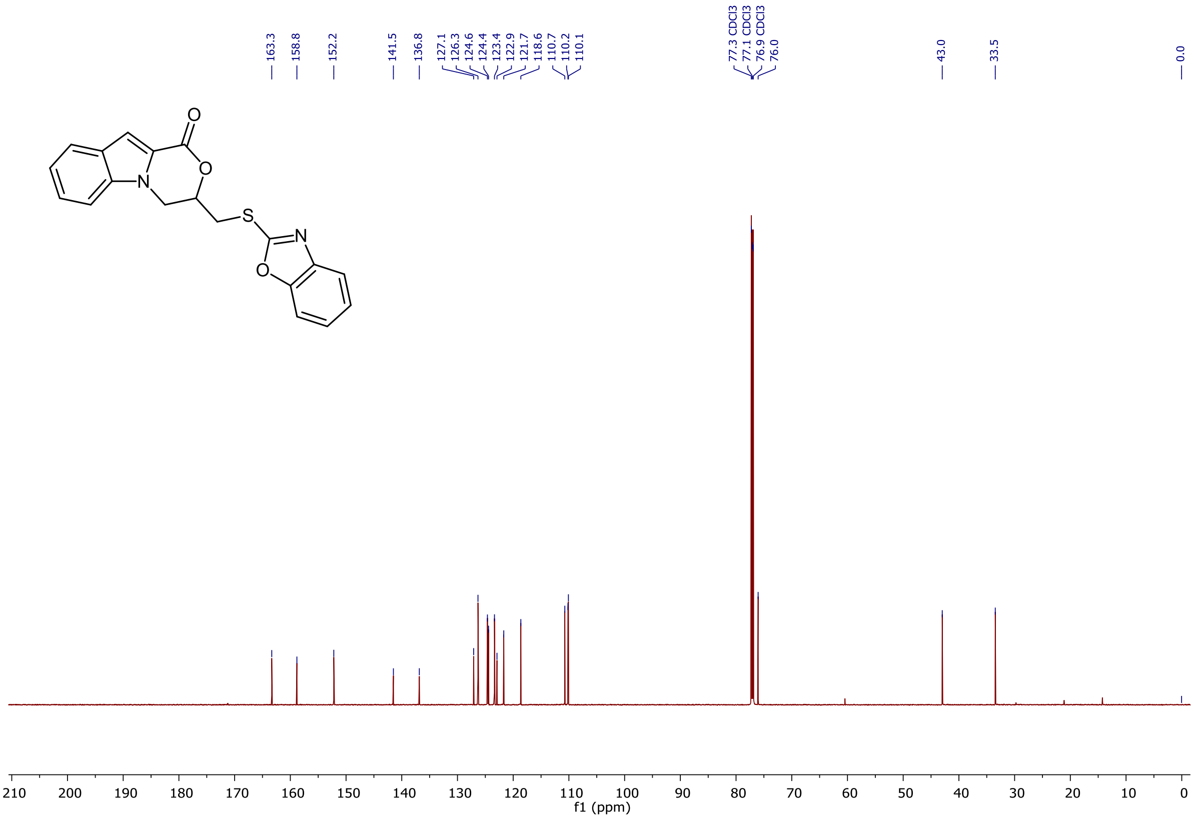

Figure S129. ^{13}C NMR (176 MHz, $CDCl_3$) spectrum of **16b**

Compound Spectrum SmartFormula Report

Analysis Info

Analysis Name	D:\Data\IZ-339.d	Acquisition Date	12/18/2024 12:37:59 PM
Method	DirectInfusion_TuneLow_pos.m	Operator	hplc
Sample Name	IZ-339	Instrument	micrOTOF-Q III 8228888.20448
Comment	SB		

Acquisition Parameter

Source Type	ESI	Ion Polarity	Positive	Set Nebulizer	0.4 Bar
Focus	Not active	Set Capillary	4500 V	Set Dry Heater	180 °C
Scan Begin	50 m/z	Set End Plate Offset	-500 V	Set Dry Gas	4.0 l/min
Scan End	1000 m/z	Set Collision Cell RF	140.0 Vpp	Set Divert Valve	Waste

#	RT [min]	Area	Int. Type	I	S/N	Chromatogram	Max. m/z	FWHM [min]
n.a.	0.6	n.a.	Single spectrum	n.a.	n.a.	n.a.	304.2631	n.a.
n.a.	5.1	n.a.	Single spectrum	n.a.	n.a.	n.a.	373.0617	n.a.

+MS, 5.1min #307

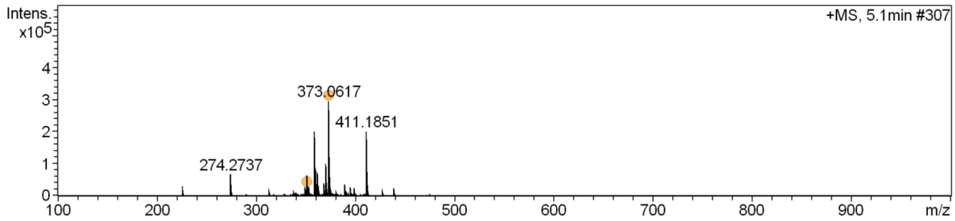

Meas. m/z	#	Ion Formula	m/z	err [ppm]	mSigma	# Sigma	Score	rdb	e⁻ Conf	N-Rule
351.0797	1	C19H15N2O3S	351.0798	-0.2	68.1	1	100.00	13.5	even	ok
373.0617	1	C19H14N2NaO3S	373.0617	-0.2	5.8	1	100.00	13.5	even	ok

Figure S130. HRMS (ESI) report of **16b**

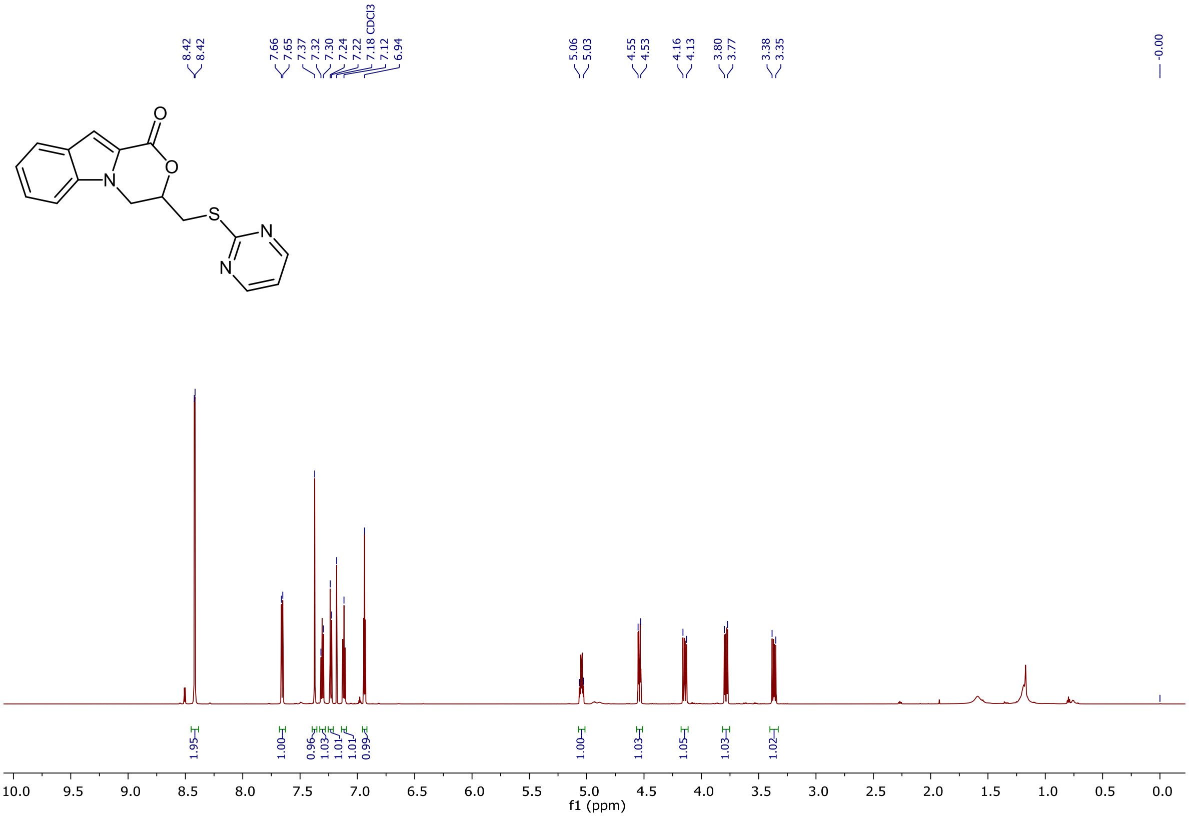

Figure S131. ^{1}H NMR (700 MHz, $CDCl_3$) spectrum of **16c**

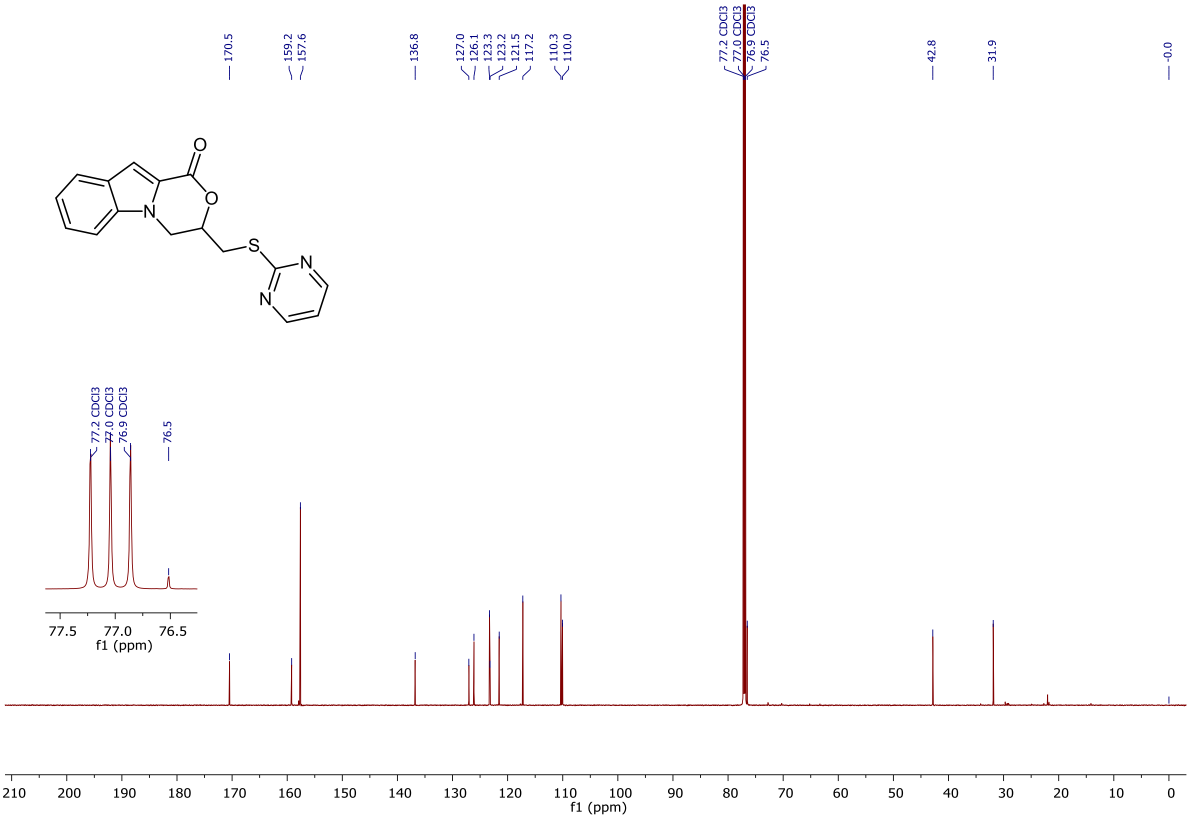

Figure S132. ^{13}C NMR (176 MHz, $CDCl_3$) spectrum of **16c**

Compound Spectrum SmartFormula Report

Analysis Info

Analysis Name	D:\Data\IZ-337.d	Acquisition Date	3/18/2025 5:35:47 PM
Method	DirectInfusion_TuneLow_pos.m	Operator	hplc
Sample Name	IZ-337	Instrument	micrOTOF-Q III 8228888.20448
Comment	AB		

Acquisition Parameter

Source Type	ESI	Ion Polarity	Positive	Set Nebulizer	0.4 Bar
Focus	Not active	Set Capillary	4500 V	Set Dry Heater	180 °C
Scan Begin	50 m/z	Set End Plate Offset	-500 V	Set Dry Gas	4.0 l/min
Scan End	1000 m/z	Set Collision Cell RF	140.0 Vpp	Set Divert Valve	Waste

#	RT [min]	Area	Int. Type	I	S/N	Chromatogram	Max. m/z	FWHM [min]
n.a.	3.5	n.a.	Single spectrum	n.a.	n.a.	n.a.	334.0624	n.a.

+MS, 3.5min #207

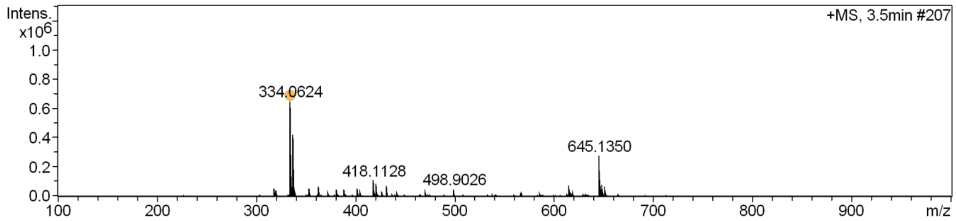

Meas. m/z	#	Ion Formula	m/z	err [ppm]	mSigma	# Sigma	Score	rdb	e⁻ Conf	N-Rule
334.0624	1	C16H13N3NaO2S	334.0621	-0.9	13.7	2	100.00	11.5	even	ok

Figure S133. HRMS (ESI) report of **16c**

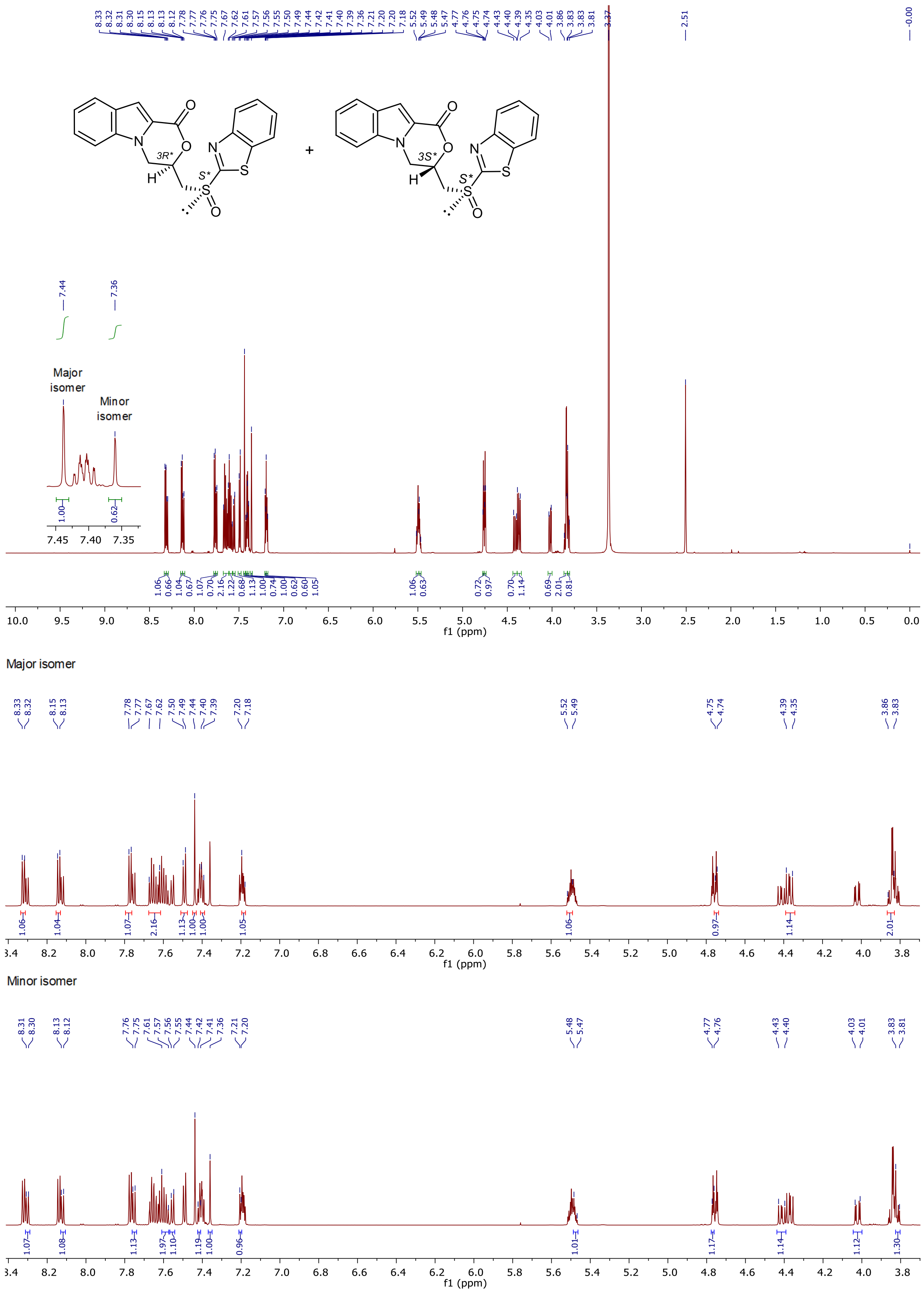

Figure S134. ^{1}H NMR (700 MHz, DMSO-d_6) spectrum of **17**

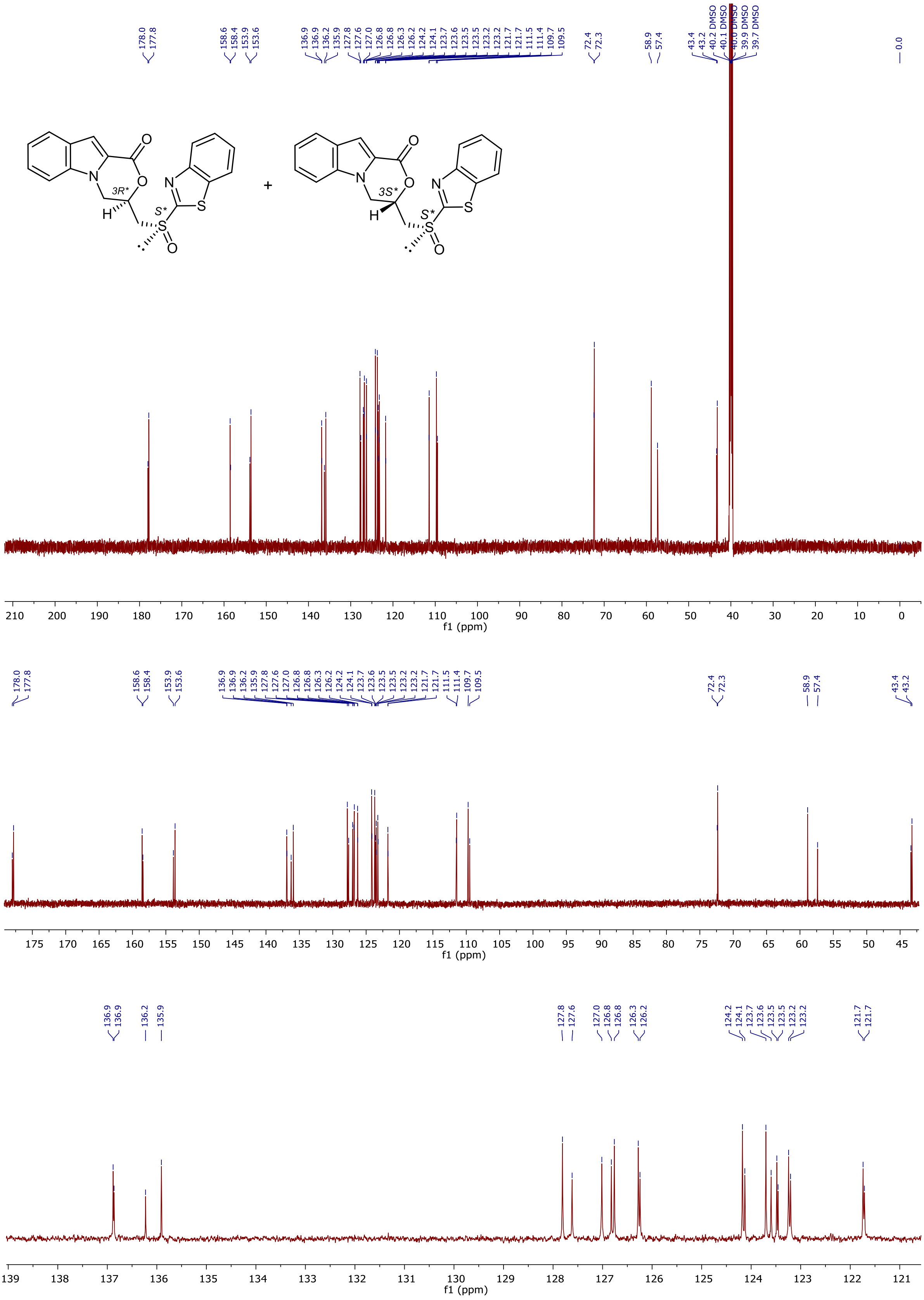

Figure S135. ^{13}C NMR (176 MHz, DMSO-d_6) spectrum of **17**

Compound Spectrum SmartFormula Report

Analysis Info

Analysis Name	D:\Data\IZ-344_1P.d	Acquisition Date	5/7/2025 4:38:28 AM
Method	DirectInfusion_TuneLow_pos.m	Operator	hplc
Sample Name	IZ-344_1P	Instrument	micrOTOF-Q III 8228888.20448
Comment	AB		

Acquisition Parameter

Source Type	ESI	Ion Polarity	Positive	Set Nebulizer	0.4 Bar
Focus	Not active	Set Capillary	4500 V	Set Dry Heater	180 °C
Scan Begin	50 m/z	Set End Plate Offset	-500 V	Set Dry Gas	4.0 l/min
Scan End	1000 m/z	Set Collision Cell RF	140.0 Vpp	Set Divert Valve	Waste

#	RT [min]	Area	Int. Type	I	S/N	Chromatogram	Max. m/z	FWHM [min]
n.a.	7.2	n.a.	Single spectrum	n.a.	n.a.	n.a.	405.0336	n.a.

+MS, 7.2min #433

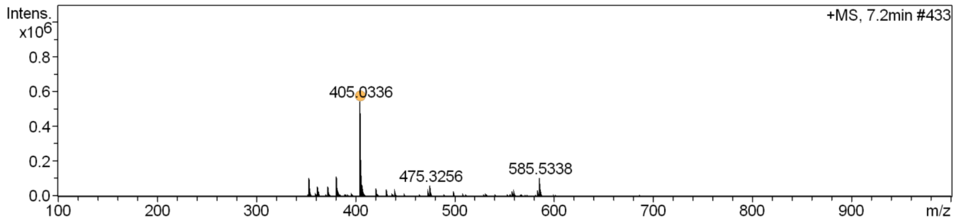

Meas. m/z	#	Ion Formula	m/z	err [ppm]	mSigma	# Sigma	Score	rdb	e⁻ Conf	N-Rule
405.0336	1	C19H14N2NaO3S2	405.0338	0.6	9.3	1	100.00	13.5	even	ok

Figure S136. HRMS (ESI) report of **17**

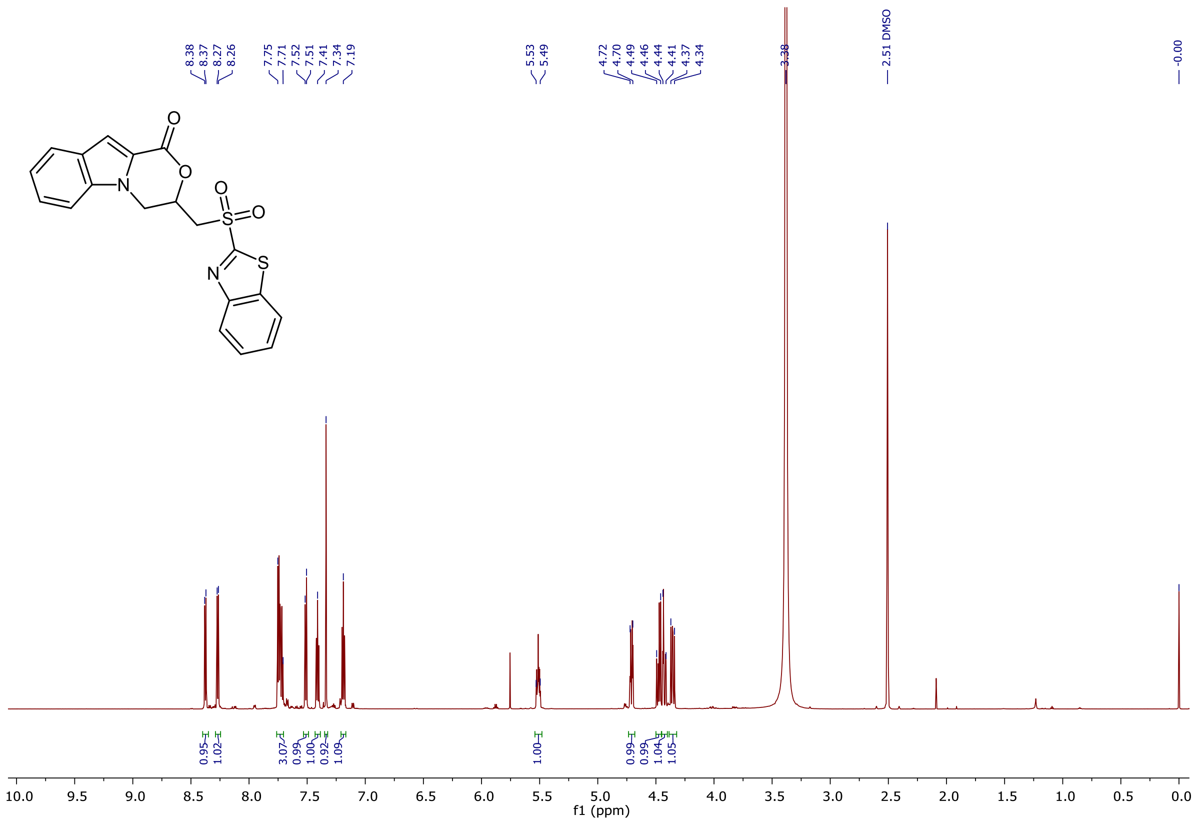

Figure S137. ^{1}H NMR (700 MHz, DMSO-d_6) spectrum of **18**

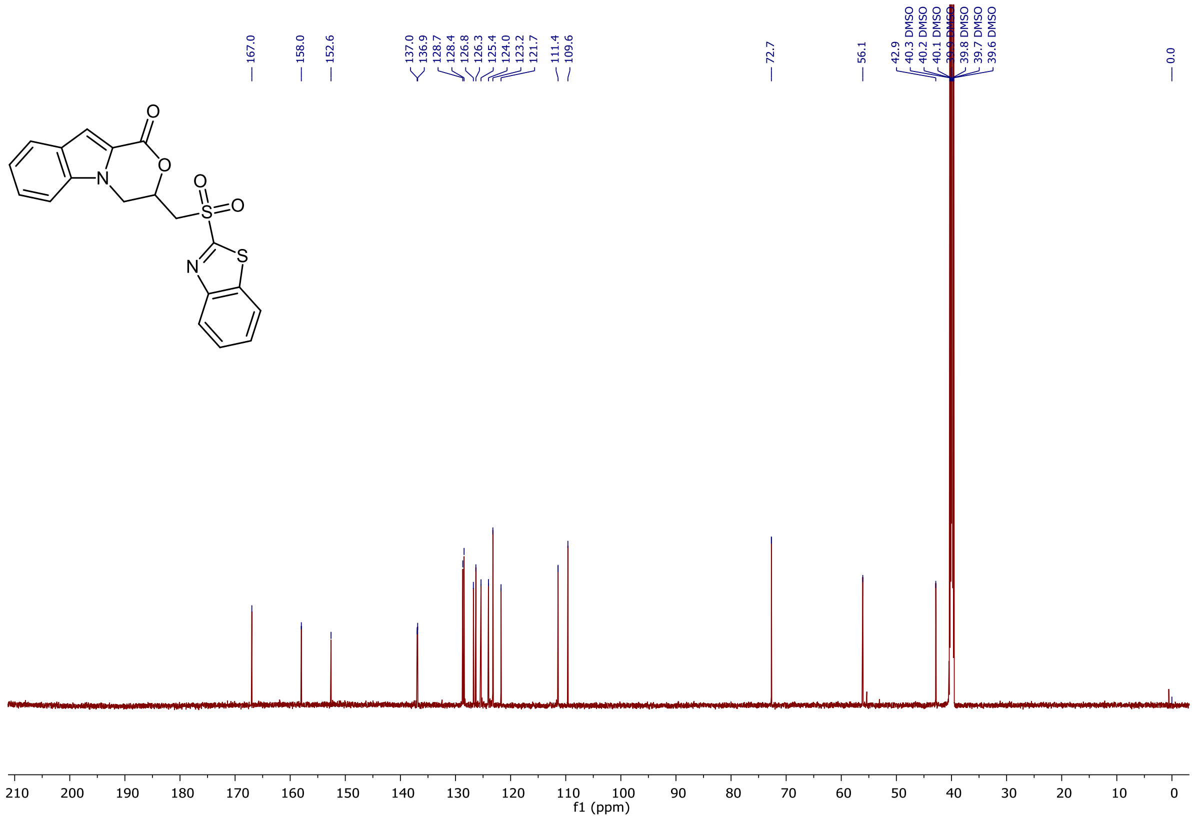

Figure S138. ^{13}C NMR (176 MHz, DMSO-d_6) spectrum of **18**

Compound Spectrum SmartFormula Report

Analysis Info

Analysis Name	D:\Data\IZ-341.d	Acquisition Date	10/18/2024 4:36:24 PM
Method	DirectInfusion_TuneLow_pos.m	Operator	hplc
Sample Name	IZ-341	Instrument	micrOTOF-Q III 8228888.20448
Comment	AB		

Acquisition Parameter

Source Type	ESI	Ion Polarity	Positive	Set Nebulizer	0.4 Bar
Focus	Not active	Set Capillary	4500 V	Set Dry Heater	180 °C
Scan Begin	50 m/z	Set End Plate Offset	-500 V	Set Dry Gas	4.0 l/min
Scan End	1000 m/z	Set Collision Cell RF	140.0 Vpp	Set Divert Valve	Waste

#	RT [min]	Area	Int. Type	I	S/N	Chromatogram	Max. m/z	FWHM [min]
n.a.	6.2	n.a.	Single spectrum	n.a.	n.a.	n.a.	421.0285	n.a.

+MS, 6.2min #371

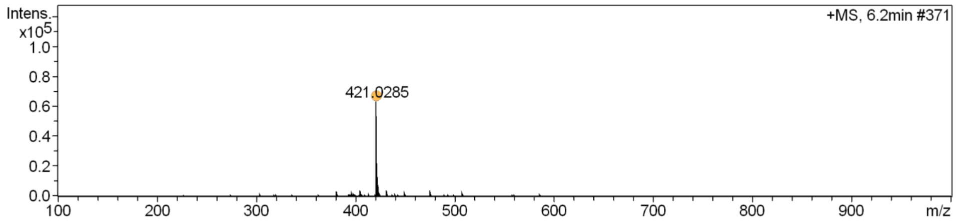

Meas. m/z	#	Ion Formula	m/z	err [ppm]	mSigma	# Sigma	Score	rdb	e⁻ Conf	N-Rule
421.0285	1	C19H14N2NaO4S2	421.0287	0.6	8.7	1	100.00	13.5	even	ok

Figure S139. HRMS (ESI) report of **18**

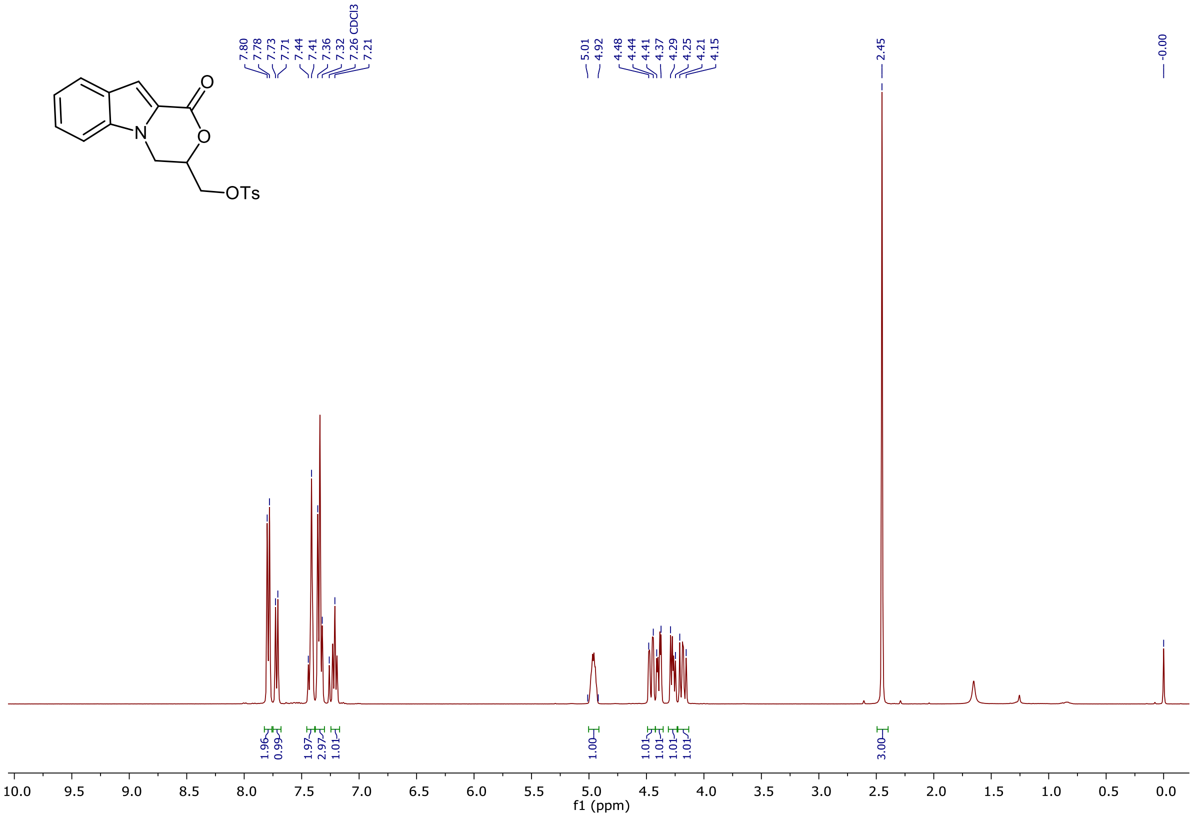

Figure S140. ^{1}H NMR (400 MHz, $CDCl_3$) spectrum of **19**

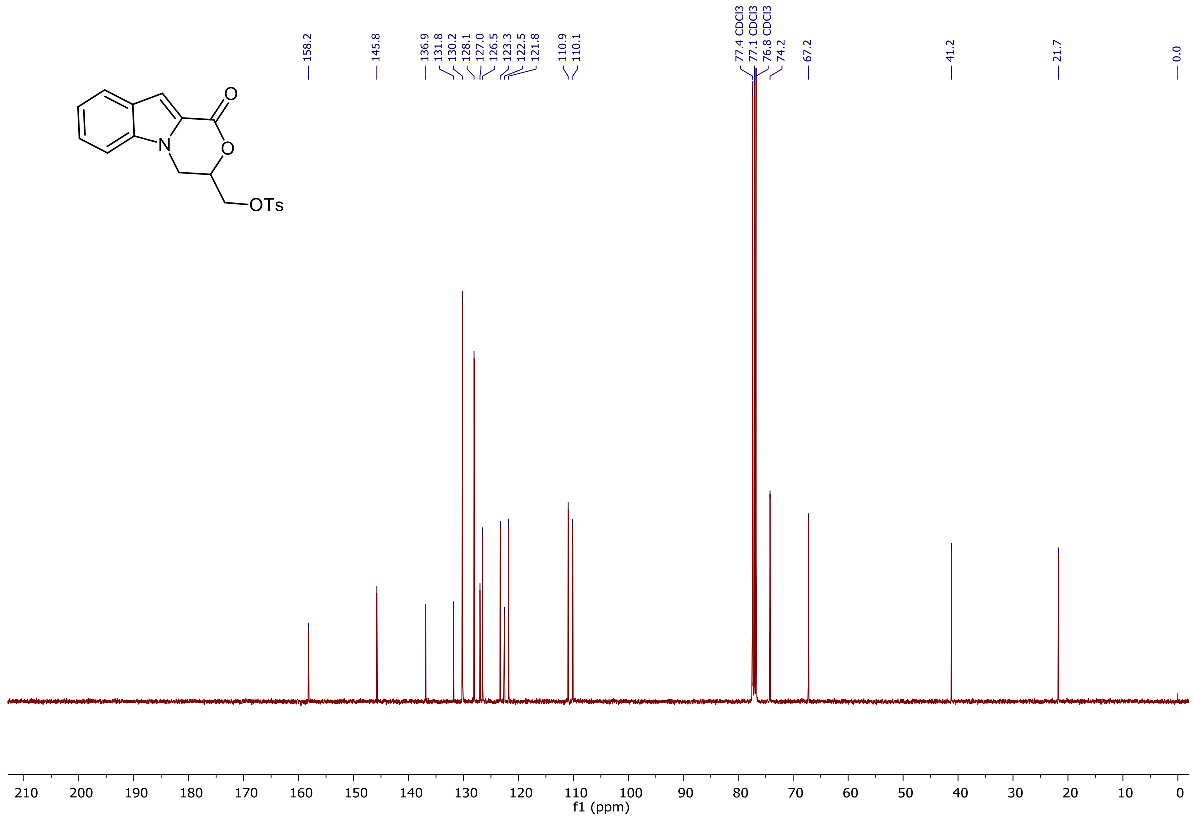

Figure S141. ^{13}C NMR (101 MHz, $CDCl_3$) spectrum of **19**

Compound Spectrum SmartFormula Report

Analysis Info

Analysis Name	D:\Data\IZ-333.d	Acquisition Date	12/18/2024 10:45:21 AM
Method	DirectInfusion_TuneLow_pos.m	Operator	hplc
Sample Name	IZ-333	Instrument	micrOTOF-Q III 8228888.20448
Comment	SB		

Acquisition Parameter

Source Type	ESI	Ion Polarity	Positive	Set Nebulizer	0.4 Bar
Focus	Not active	Set Capillary	4500 V	Set Dry Heater	180 °C
Scan Begin	50 m/z	Set End Plate Offset	-500 V	Set Dry Gas	4.0 l/min
Scan End	1000 m/z	Set Collision Cell RF	140.0 Vpp	Set Divert Valve	Waste

#	RT [min]	Area	Int. Type	I	S/N	Chromatogram	Max. m/z	FWHM [min]
n.a.	0.1	n.a.	Single spectrum	n.a.	n.a.	n.a.	304.2622	n.a.
n.a.	4.3	n.a.	Single spectrum	n.a.	n.a.	n.a.	394.0720	n.a.

+MS, 4.3min #257

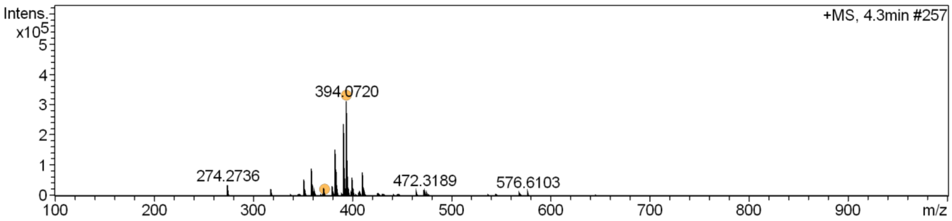

Meas. m/z	#	Ion Formula	m/z	err [ppm]	mSigma	# Sigma	Score	rdb	e⁻ Conf	N-Rule
372.0893	1	C19H18NO5S	372.0900	2.0	140.8	1	100.00	11.5	even	ok
394.0720	1	C19H17NNaO5S	394.0720	0.2	6.7	1	100.00	11.5	even	ok

Figure S142. HRMS (ESI) report of **19**

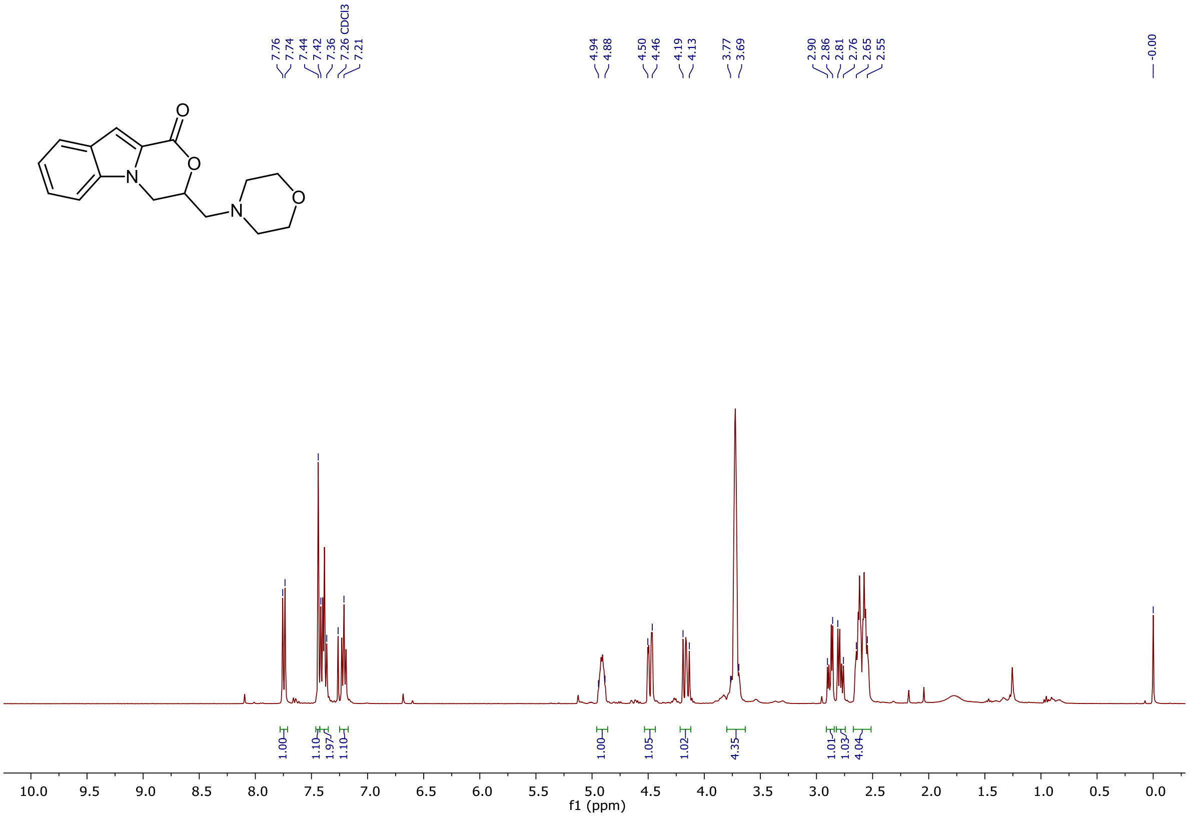

Figure S143. ^{1}H NMR (400 MHz, $CDCl_3$) spectrum of **20a**

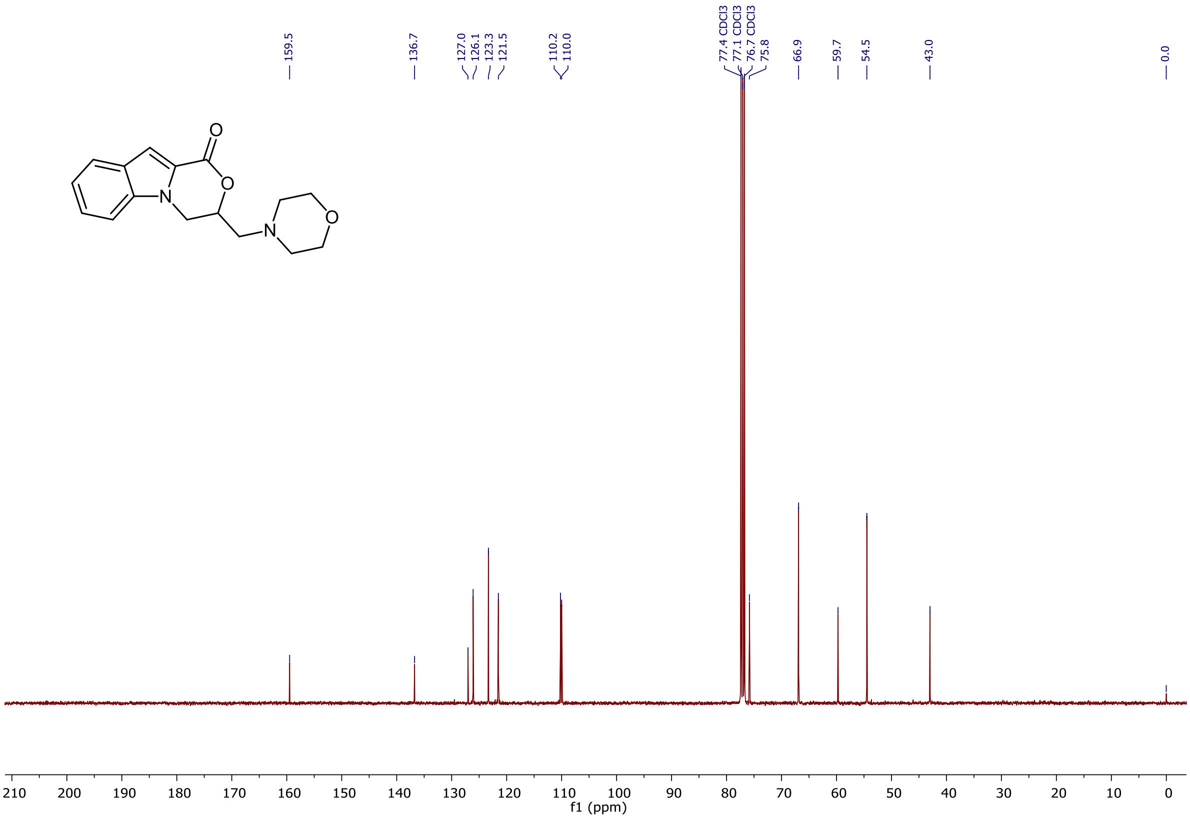

Figure S144. ^{13}C NMR (101 MHz, $CDCl_3$) spectrum of **20a**

Compound Spectrum SmartFormula Report

Analysis Info

Analysis Name	D:\Data\IZ-351.d	Acquisition Date	12/18/2024 1:10:36 PM
Method	DirectInfusion_TuneLow_pos.m	Operator	hplc
Sample Name	IZ-351	Instrument	micrOTOF-Q III 8228888.20448
Comment	SB		

Acquisition Parameter

Source Type	ESI	Ion Polarity	Positive	Set Nebulizer	0.4 Bar
Focus	Not active	Set Capillary	4500 V	Set Dry Heater	180 °C
Scan Begin	50 m/z	Set End Plate Offset	-500 V	Set Dry Gas	4.0 l/min
Scan End	1000 m/z	Set Collision Cell RF	140.0 Vpp	Set Divert Valve	Waste

#	RT [min]	Area	Int. Type	I	S/N	Chromatogram	Max. m/z	FWHM [min]
n.a.	0.7	n.a.	Single spectrum	n.a.	n.a.	n.a.	304.2628	n.a.
n.a.	6.9	n.a.	Single spectrum	n.a.	n.a.	n.a.	309.1210	n.a.

+MS, 6.9min #412

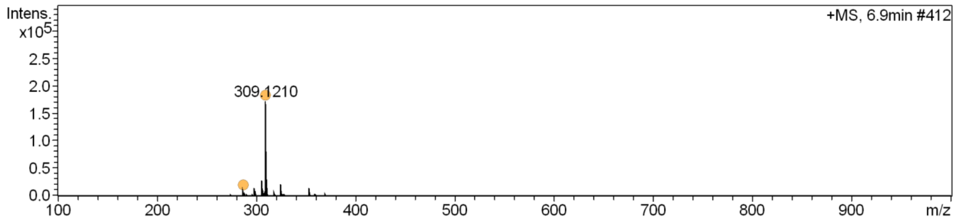

Meas. m/z	#	Ion Formula	m/z	err [ppm]	mSigma	# Sigma	Score	rdb	e⁻ Conf	N-Rule
287.1390	1	C16H19N2O3	287.1390	-0.0	167.9	1	100.00	8.5	even	ok
309.1210	1	C16H18N2NaO3	309.1210	0.2	3.9	1	100.00	8.5	even	ok

Figure S145. HRMS (ESI) report of **20a**

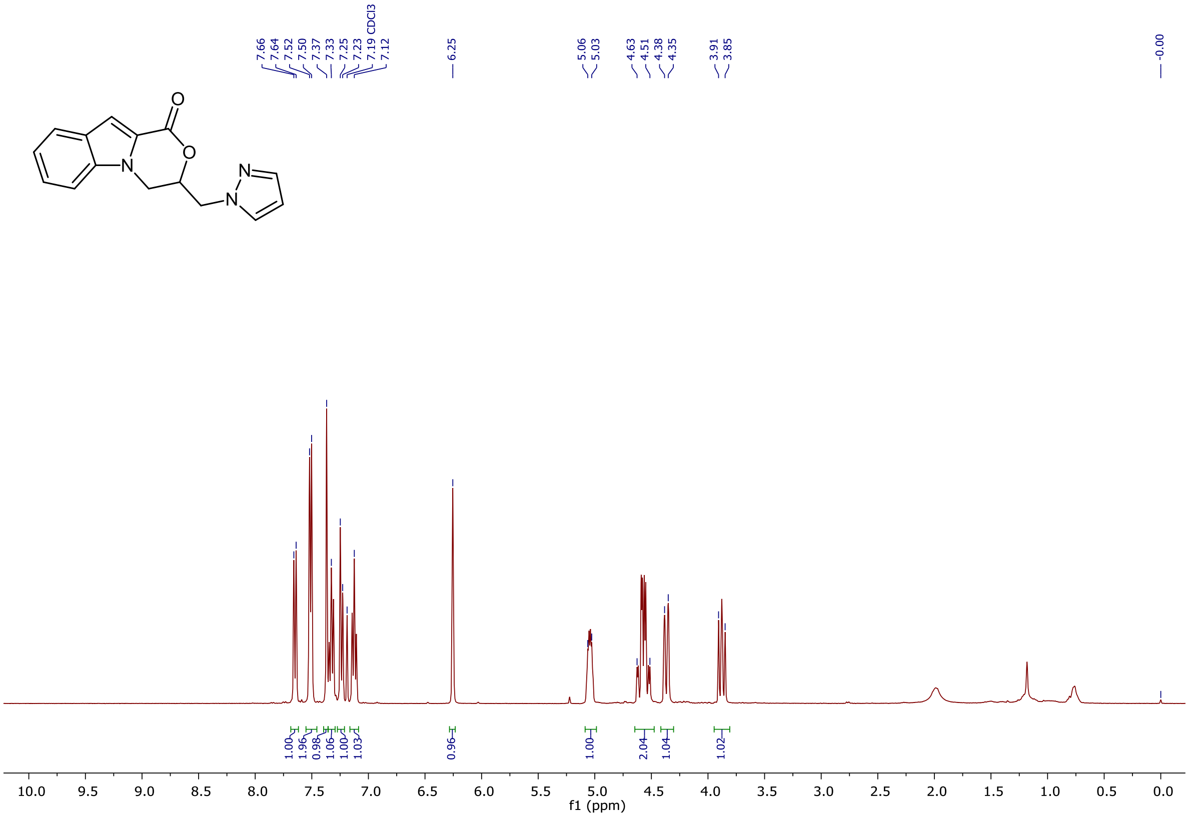

Figure S146. ^{1}H NMR (400 MHz, $CDCl_3$) spectrum of **20b**

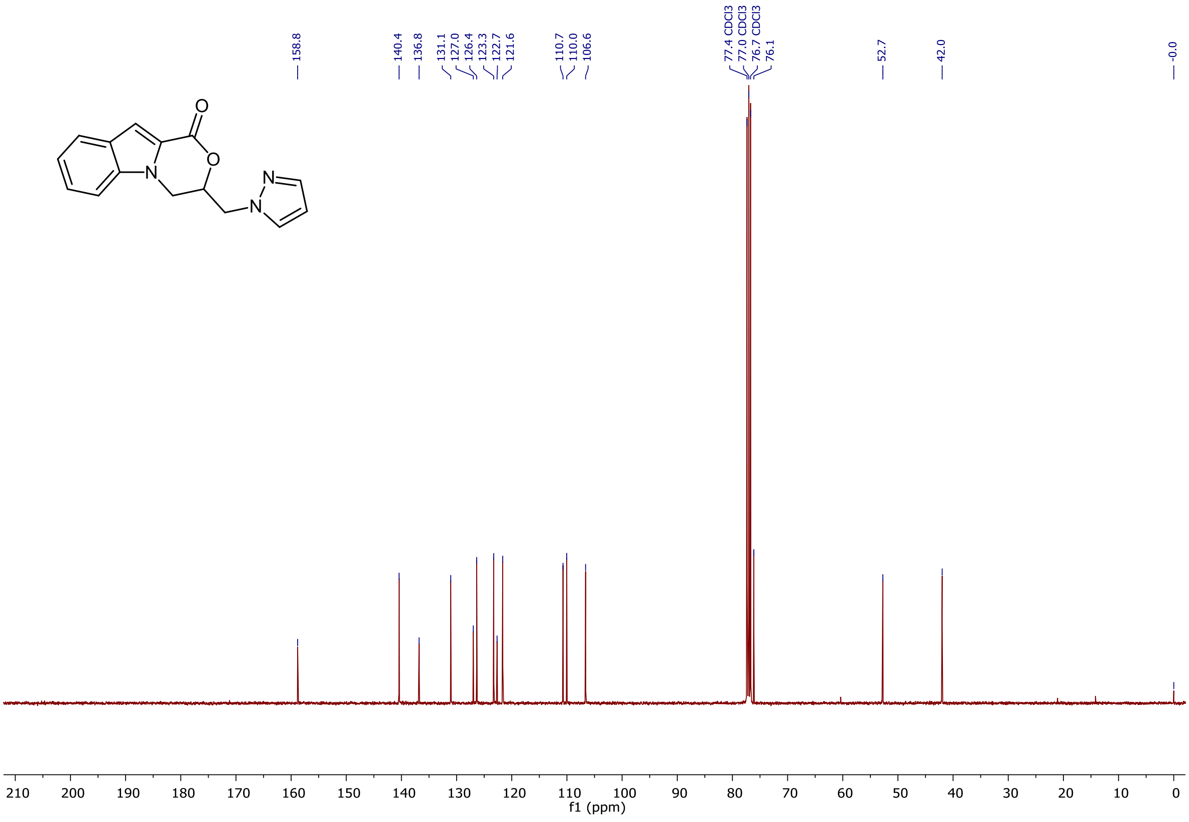

Figure S147. ^{13}C NMR (101 MHz, $CDCl_3$) spectrum of **20b**

Compound Spectrum SmartFormula Report

Analysis Info

Analysis Name	D:\Data\IZ-348.d	Acquisition Date	12/18/2024 12:55:49 PM
Method	DirectInfusion_TuneLow_pos.m	Operator	hplc
Sample Name	IZ-348	Instrument	micrOTOF-Q III 8228888.20448
Comment	SB		

Acquisition Parameter

Source Type	ESI	Ion Polarity	Positive	Set Nebulizer	0.4 Bar
Focus	Not active	Set Capillary	4500 V	Set Dry Heater	180 °C
Scan Begin	50 m/z	Set End Plate Offset	-500 V	Set Dry Gas	4.0 l/min
Scan End	1000 m/z	Set Collision Cell RF	140.0 Vpp	Set Divert Valve	Waste

#	RT [min]	Area	Int. Type	I	S/N	Chromatogram	Max. m/z	FWHM [min]
n.a.	2.2	n.a.	Single spectrum	n.a.	n.a.	n.a.	304.2631	n.a.
n.a.	4.7	n.a.	Single spectrum	n.a.	n.a.	n.a.	290.0900	n.a.

+MS, 4.7min #284

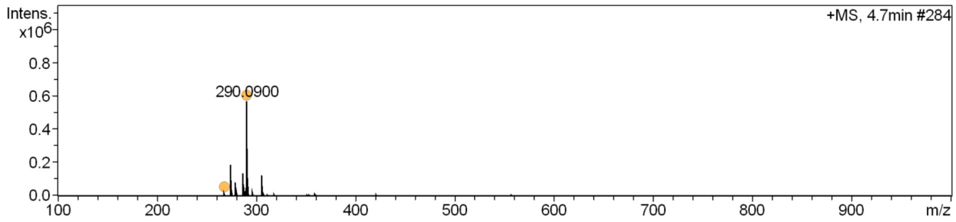

Meas. m/z	#	Ion Formula	m/z	err [ppm]	mSigma	# Sigma	Score	rdb	e^- Conf	N-Rule
268.1081	1	C15H14N3O2	268.1081	-0.0	7.7	1	100.00	10.5	even	ok
290.0900	1	C15H13N3NaO2	290.0900	-0.0	9.7	1	100.00	10.5	even	ok

Figure S148. HRMS (ESI) report of **20b**

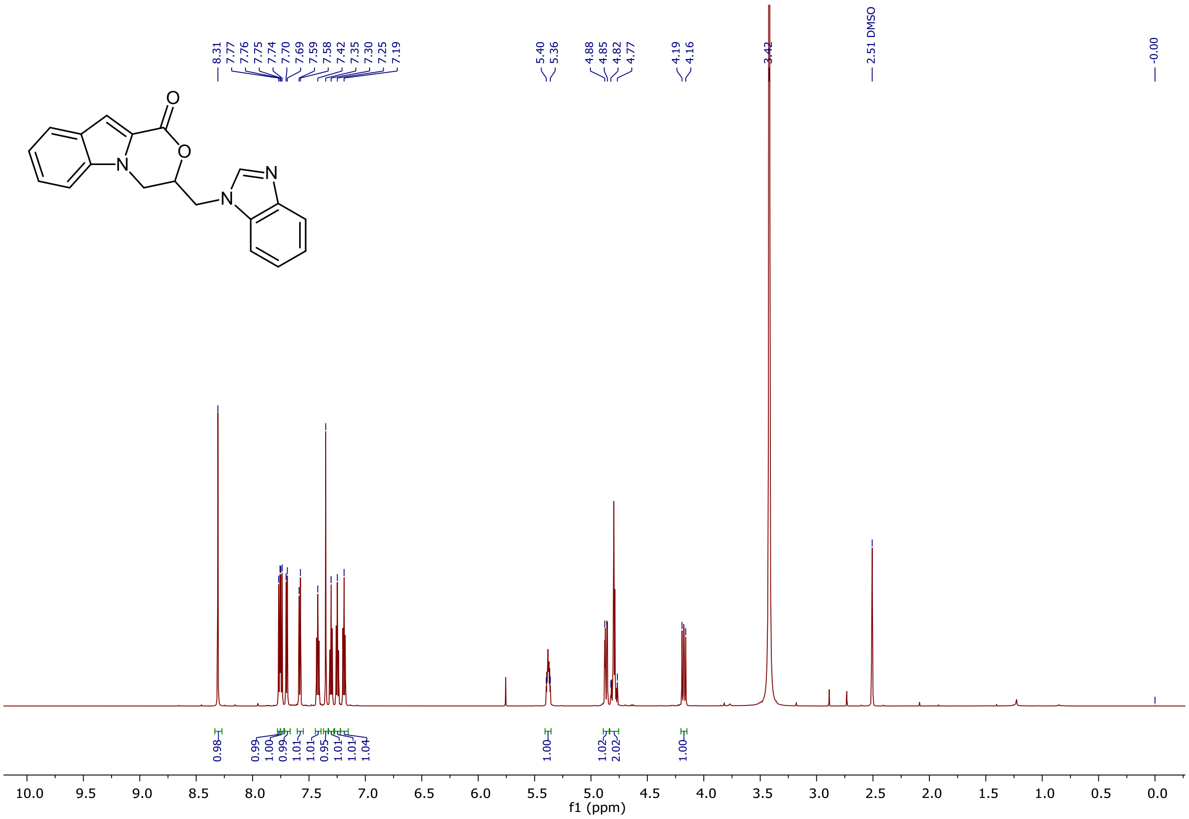

Figure S149. ^{1}H NMR (700 MHz, DMSO-d_6) spectrum of **20c**

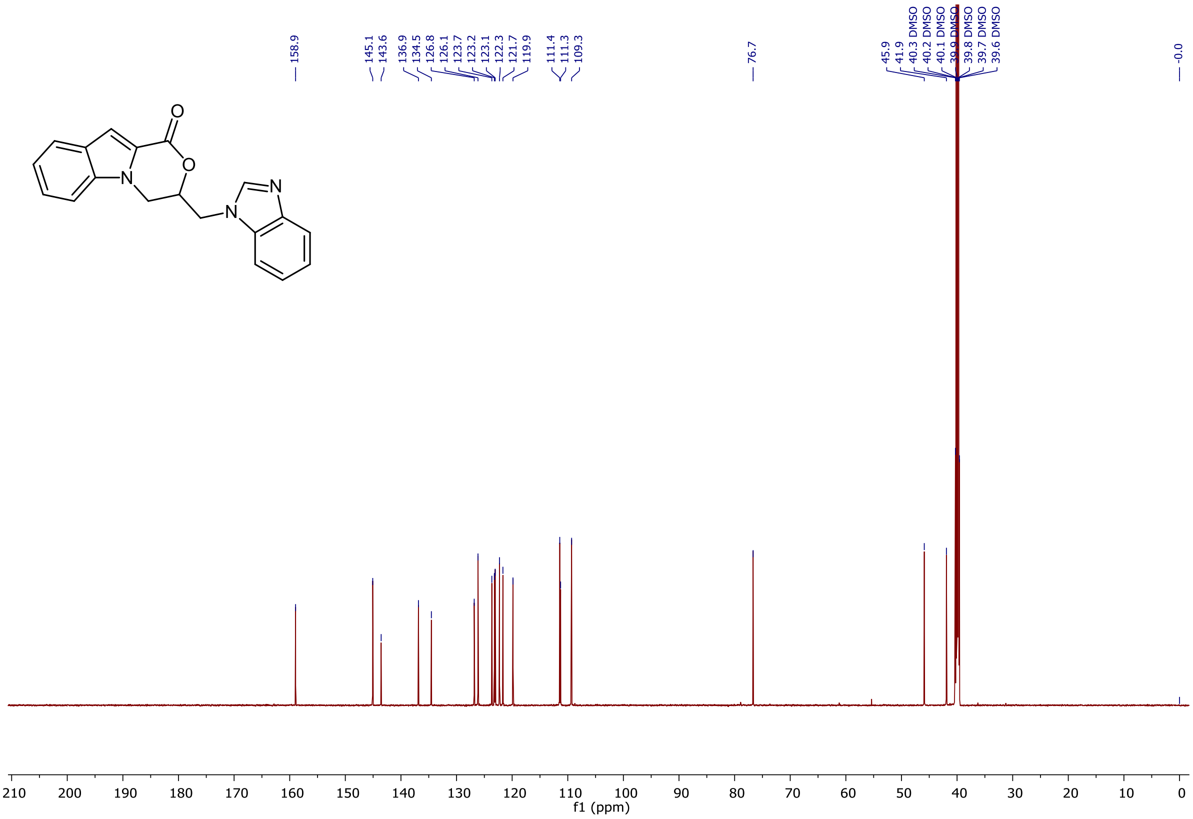

Figure S150. ^{13}C NMR (176 MHz, DMSO-d_6) spectrum of **20c**

Compound Spectrum SmartFormula Report

Analysis Info

Analysis Name	D:\Data\IZ-354.d	Acquisition Date	12/18/2024 2:11:00 PM
Method	DirectInfusion_TuneLow_pos.m	Operator	hplc
Sample Name	IZ-354	Instrument	micrOTOF-Q III 8228888.20448
Comment	SB		

Acquisition Parameter

Source Type	ESI	Ion Polarity	Positive	Set Nebulizer	0.4 Bar
Focus	Not active	Set Capillary	4500 V	Set Dry Heater	180 °C
Scan Begin	50 m/z	Set End Plate Offset	-500 V	Set Dry Gas	4.0 l/min
Scan End	1000 m/z	Set Collision Cell RF	140.0 Vpp	Set Divert Valve	Waste

#	RT [min]	Area	Int. Type	I	S/N	Chromatogram	Max. m/z	FWHM [min]
n.a.	0.1	n.a.	Single spectrum	n.a.	n.a.	n.a.	304.2628	n.a.
n.a.	5.9	n.a.	Single spectrum	n.a.	n.a.	n.a.	318.1237	n.a.

+MS, 5.9min #354

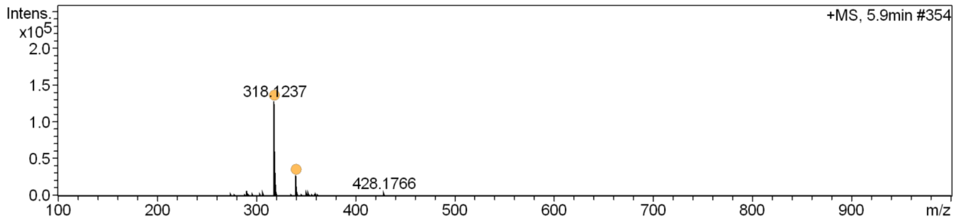

Meas. m/z	#	Ion Formula	m/z	err [ppm]	mSigma	# Sigma	Score	rdb	e⁻ Conf	N-Rule
318.1237	1	C19H16N3O2	318.1237	-0.0	22.7	1	100.00	13.5	even	ok
340.1052	1	C19H15N3NaO2	340.1056	-1.5	5.0	1	100.00	13.5	even	ok

Figure S151. HRMS (ESI) report of **20c**

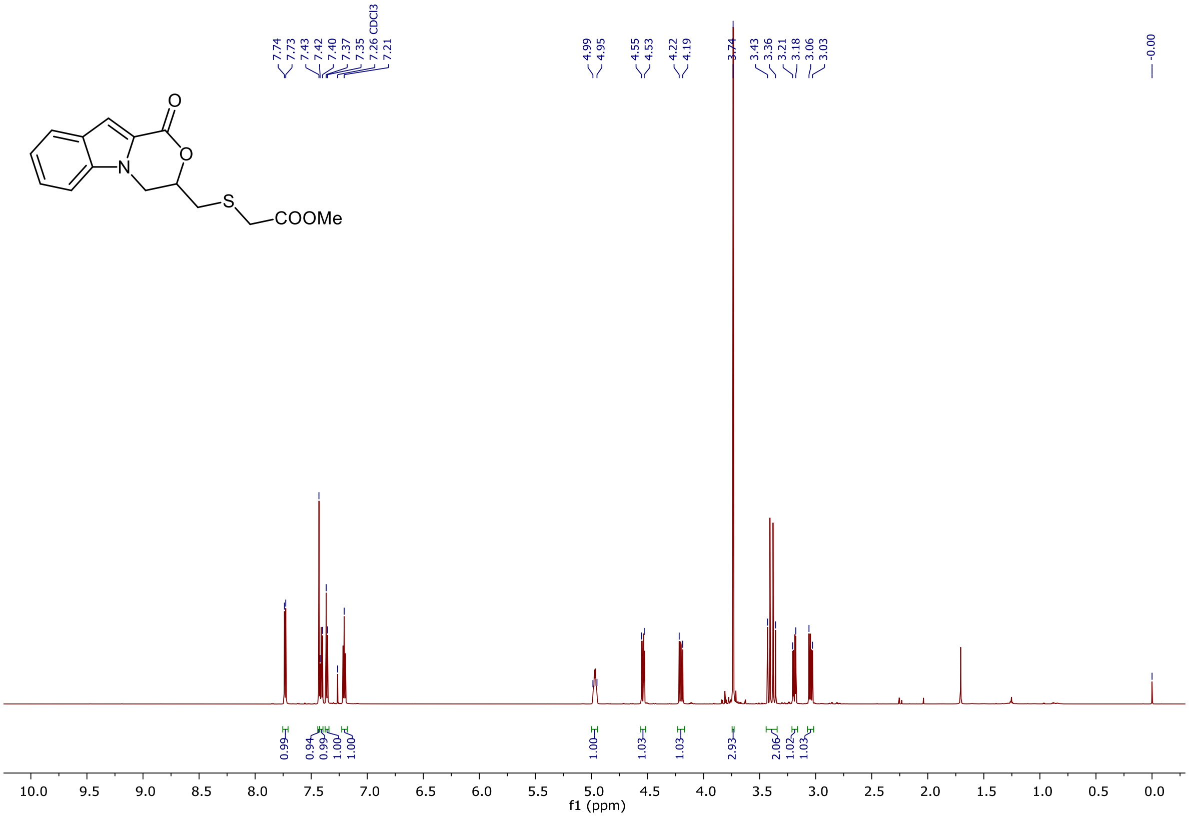

Figure S152. ^{1}H NMR (700 MHz, $CDCl_3$) spectrum of **21**

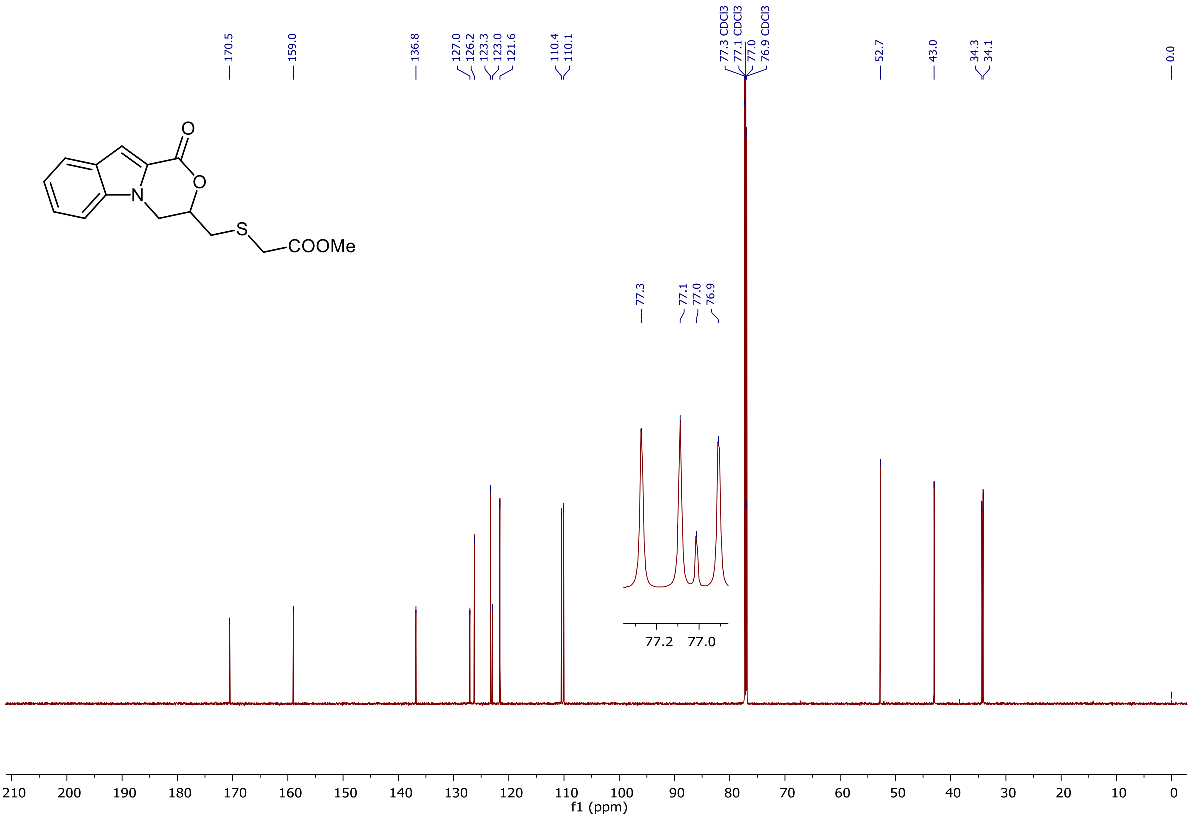

Figure S153. ^{13}C NMR (176 MHz, $CDCl_3$) spectrum of **21**

Compound Spectrum SmartFormula Report

Analysis Info

Analysis Name	D:\Data\IZ-365.d	Acquisition Date	3/19/2025 5:41:43 PM
Method	DirectInfusion_TuneLow_pos.m	Operator	hplc
Sample Name	IZ-365	Instrument	micrOTOF-Q III 8228888.20448
Comment	AB		

Acquisition Parameter

Source Type	ESI	Ion Polarity	Positive	Set Nebulizer	0.4 Bar
Focus	Not active	Set Capillary	4500 V	Set Dry Heater	180 °C
Scan Begin	50 m/z	Set End Plate Offset	-500 V	Set Dry Gas	4.0 l/min
Scan End	1000 m/z	Set Collision Cell RF	140.0 Vpp	Set Divert Valve	Waste

#	RT [min]	Area	Int. Type	I	S/N	Chromatogram	Max. m/z	FWHM [min]
n.a.	3.0	n.a.	Single spectrum	n.a.	n.a.	n.a.	328.0616	n.a.

+MS, 3.0min #180

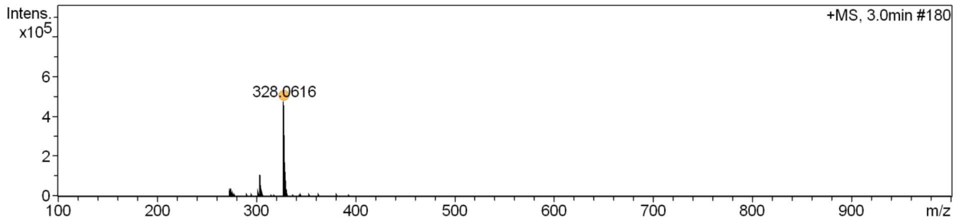

Meas. m/z	#	Ion Formula	m/z	err [ppm]	mSigma	# Sigma	Score	rdb	e⁻ Conf	N-Rule
328.0616	1	C15H15NNaO4S	328.0614	-0.7	3.8	1	100.00	8.5	even	ok

Figure S154. HRMS (ESI) report of **21**

X-Ray analysis of compound 6a:

Single crystals of $C_{12}H_{11}NO_3$ [**6a**] were investigated on a Rigaku, XtaLAB Synergy, Dualflex, HyPix diffractometer. The crystal was kept at 160.0(1) K during data collection. Using Olex2 [1], the structure was solved with the SIR2011 [2] structure solution program using Direct Methods and refined with the olex2.refine [3] refinement package using Gauss-Newton minimisation.

Table S1. Experimental parameters and CCDC-2428119

**Sample**	**Machine**	**Source**	**Temp.** [K]	**CCDC**
**6a**	Rigaku, XtaLAB Synergy, Dualflex, HyPix	μ(Cu Kα) = 0.889 mm^{-1}	160.0(1)	2428119

Table S2. Sample and crystal data of **6a**

**Chemical formula**	$C_{12}H_{11}NO_3$	**Crystal system**	Orthorhombic	
**Formula weight [g/mol]**	217.23	**Space group**	*Pna*2_1	
**Temperature [K]**	160.0(1)	**Z**	4	
		**Volume [Å^3]**	976.72(4)	
**Radiation wavelength [Å]**	1.54184	**Unit cell dimensions [Å^3] and [°]**	30.9767(6)<br>6.3602(2)<br>4.9575(1)	90<br>90<br>90
**Crystal size [mm^3]**	0.14 × 0.06 × 0.03	**Absorption coefficient [mm^{-1}]**	0.889	
**Density (calculated) [g/cm^3]**	1.4771	**F(000) [e$^-$]**	456	

Table S3. Data collection and structure refinement of compound **6a**

**Index ranges**	$-39 \leq h \leq 29$<br>$-8 \leq k \leq 7$<br>$-6 \leq l \leq 6$	**2θ range for data collection [°]**	160	
**Reflection numbers**	7627	**Data / restraints / parameters**	2001/1/149	
**Goodness-of-fit on F₂**	1.064	**Final R indices**	All data	R_1 = 0.0434<br>wR_2 = 0.1145
**Largest diff. peak and hole [e Å^{-3}]**	0.36/-0.19		I > 2σ(I)	R_1 = 0.0421<br>wR_2 = 0.1132

Table S4. Fractional Atomic Coordinates (×10^4) and Equivalent Isotropic Displacement Parameters (Å^2×10^3) for **6a**. U_{eq} is defined as 1/3 of of the trace of the orthogonalised U_{IJ} tensor.

**Atom**	***x***	***y***	***z***	***U*(eq)**
C1	3399.3(6)	3981(3)	1413(4)	26.6(4)
O2	3078.5(4)	2592(2)	922(3)	30.7(3)
C3	2974.4(6)	1014(3)	2999(4)	30.7(4)
C4	3373.6(6)	-100(3)	3939(4)	32.1(4)
N5	3681.7(5)	1496(2)	4773(3)	25.5(3)
C5A	4015.5(6)	1330(3)	6565(4)	26.2(4)

C6	4122.9(6)	-340(3)	8284(4)	29.7(4)
C7	4476.6(6)	-50(3)	9942(5)	32.8(4)
C8	4717.9(6)	1824(4)	9900(4)	34.3(4)
C9	4611.6(6)	3465(3)	8209(4)	32.7(4)
C9A	4252.6(6)	3234(3)	6476(4)	26.7(4)
C10	4052.9(6)	4538(3)	4516(4)	28.8(4)
C10A	3707.7(6)	3430(3)	3513(4)	26.0(4)
O11	3415.7(4)	5561(2)	45(3)	31.1(3)
C12	2653.0(6)	-462(3)	1655(5)	32.0(4)
O13	2257.6(5)	570(2)	1079(3)	37.0(4)

Table S5. Anisotropic Displacement Parameters ($Å^2×10^3$) for **6a**. The Anisotropic displacement factor exponent takes the form: $-2\pi^2[h^2a^{*2}U_{11}+2hka^*b^*U_{12}+\ldots]$.

Atom	*U*11	*U*22	*U*33	*U*12	*U*13	*U*23
C1	27.9(8)	23.6(8)	28.3(9)	-0.5(6)	2.7(7)	0.2(8)
O2	32.0(6)	27.4(7)	32.6(7)	-4.9(5)	-4.1(5)	5.3(6)
C3	33.4(9)	28.5(9)	30.2(10)	-2.1(7)	2.3(8)	1.8(8)
C4	32.4(9)	27.8(9)	36.0(10)	-6.8(7)	-3.6(8)	4.0(8)
N5	27.7(7)	21.4(7)	27.5(8)	-1.7(6)	0.1(6)	0.9(6)
C5A	25.7(8)	26.5(9)	26.3(9)	-0.1(6)	3.7(7)	-2.1(8)
C6	30.4(9)	29.4(9)	29.2(9)	0.5(7)	2.3(7)	2.3(9)
C7	32.2(9)	37.1(10)	29.0(8)	3.7(8)	-0.3(8)	1.2(9)
C8	29.3(8)	41.2(11)	32.3(9)	2.2(8)	-3.3(8)	-2.4(9)
C9	28.7(9)	37.8(10)	31.7(11)	-5.6(7)	0.7(8)	-3.5(9)
C9A	27.3(8)	29.0(8)	23.9(9)	-1.0(6)	3.0(7)	-2.3(8)
C10	31.1(9)	25.0(9)	30.3(10)	-3.6(7)	1.3(8)	-0.0(7)
C10A	27.5(8)	23.1(8)	27.4(9)	0.5(6)	3.5(7)	0.7(7)
O11	34.6(7)	25.6(6)	33.0(7)	-2.5(5)	-1.3(6)	5.3(6)
C12	31.6(9)	26.7(9)	37.7(11)	-4.0(7)	-3.3(8)	2.6(8)
O13	31.8(7)	40.4(8)	38.8(8)	-0.5(6)	3.3(7)	8.1(7)

Table S6. Bond Lengths for **6a**.

Atom	Atom	Length/Å	Atom	Atom	Length/Å
C1	O2	1.352(2)	C5A	C6	1.402(3)
C1	C10A	1.456(3)	C5A	C9A	1.417(3)
C1	O11	1.214(2)	C6	C7	1.382(3)
O2	C3	1.473(2)	C7	C8	1.407(3)
C3	C4	1.499(3)	C8	C9	1.379(3)
C3	C12	1.522(3)	C9	C9A	1.413(3)
C4	N5	1.453(2)	C9A	C10	1.420(3)
N5	C5A	1.367(2)	C10	C10A	1.374(3)
N5	C10A	1.382(2)	C12	O13	1.418(2)

Table S7. Bond Angles for **6a**.

Atom	Atom	Atom	Angle/°	Atom	Atom	Atom	Angle/°
C10A	C1	O2	116.99(16)	C9A	C5A	C6	122.91(17)
O11	C1	O2	118.13(17)	C7	C6	C5A	116.68(18)
O11	C1	C10A	124.86(17)	C8	C7	C6	121.67(19)
C3	O2	C1	118.69(15)	C9	C8	C7	121.55(19)
C4	C3	O2	111.00(15)	C9A	C9	C8	118.63(19)
C12	C3	O2	104.90(16)	C9	C9A	C5A	118.56(18)

C12	C3	C4	112.58(17)	C10	C9A	C5A	107.16(16)
N5	C4	C3	107.47(16)	C10	C9A	C9	134.28(18)
C5A	N5	C4	128.90(16)	C10A	C10	C9A	106.67(17)
C10A	N5	C4	122.10(16)	N5	C10A	C1	119.93(16)
C10A	N5	C5A	108.53(15)	C10	C10A	C1	130.25(18)
C6	C5A	N5	129.25(17)	C10	C10A	N5	109.80(17)
C9A	C5A	N5	107.83(16)	O13	C12	C3	111.57(16)

Table S8. Hydrogen Atom Coordinates (Å×104) and Isotropic Displacement Parameters (Å2×103) for **6a**.

**Atom**	***x***	***y***	***z***	***U*(iso)**
H3	2833.1(6)	1723(3)	4568(4)	36.8(5)
H4a	3304.5(6)	-1040(3)	5470(4)	38.5(5)
H4b	3495.6(6)	-959(3)	2459(4)	38.5(5)
H6	3960.6(6)	-1607(3)	8308(4)	35.6(5)
H7	4558.9(6)	-1145(3)	11141(5)	39.3(5)
H8	4959.7(6)	1963(4)	11065(4)	41.1(5)
H9	4776.3(6)	4725(3)	8208(4)	39.3(5)
H10	4140.9(6)	5911(3)	3998(4)	34.6(5)
H12a	2778.8(6)	-1006(3)	-43(5)	38.4(5)
H12b	2597.2(6)	-1674(3)	2858(5)	38.4(5)
H13	2073(10)	550(50)	2490(80)	55(9)

X-Ray analysis of compound 18:

Single crystals of $C_{19}H_{14}N_2O_4S_2$ [**18**] were investigated on a Rigaku, XtaLAB Synergy, Dualflex, HyPix diffractometer. The crystal was kept at 160.0(3) K during data collection. Using Olex2 [1], the structure was solved with the SIR2014 [2] structure solution program using Direct Methods and refined with the olex2.refine [3] refinement package using Levenberg-Marquardt minimisation.

Table S9. Experimental parameters and CCDC-2428115

Sample	Machine	Source	Temp. [K]	CCDC
**18**	Rigaku, XtaLAB Synergy, Dualflex, HyPix	μ(Cu Kα) = 3.082 mm^{-1}	160.0(3)	2428115

Table S10. Sample and crystal data of **18**

**Chemical formula**	$C_{19}H_{14}N_2O_4S_2$	**Crystal system**	triclinic	
**Formula weight [g/mol]**	398.466	**Space group**	$P\bar{1}$	
**Temperature [K]**	160.0(3)	**Z**	2	
		**Volume [Å³]**	857.97(5)	
**Radiation wavelength [Å]**	1.54184	**Unit cell dimensions [Å³] and [°]**	6.8591(1)<br>11.0368(4)<br>11.7919(4)	93.795(3)<br>92.677(2)<br>105.124(2)
**Crystal size [mm³]**	0.14 × 0.09 × 0.03	**Absorption coefficient [mm^{-1}]**	3.082	
**Density (calculated) [g/cm³]**	1.542	**F(000) [e^-]**	412	

Table S11. Data collection and structure refinement of compound **18**

**Index ranges**	$-6 \leq h \leq 8$<br>$-13 \leq k \leq 13$<br>$-15 \leq l \leq 14$	**2θ range for data collection [°]**	160	
**Reflection numbers**	12166	**Data / restraints / parameters**	3655/0/248	
**Goodness-of-fit on F2**	1.038	**Final R indices**	All data	R_1 = 0.0704, wR_2 = 0.1933
**Largest diff. peak and hole [e Å⁻³]**	0.90/-0.54		I > 2σ(I)	R_1 = 0.0682, wR_2 = 0.1924

Table S12. Fractional Atomic Coordinates (×10⁴) and Equivalent Isotropic Displacement Parameters (Å²×10³) for **18**. U_{eq} is defined as 1/3 of of the trace of the orthogonalised U_{IJ} tensor.

**Atom**	***x***	***y***	***z***	***U*(eq)**
C1	6949(6)	7997(4)	5598(4)	27.4(8)
O2	5245(4)	7361(3)	6068(3)	28.3(6)
C3	3389(6)	6907(4)	5329(4)	28.3(8)
C4	2925(6)	7950(4)	4684(4)	30.7(9)
N5	4712(5)	8508(3)	4104(3)	28.2(7)
C5A	4856(6)	9191(4)	3163(4)	28.8(9)
C6	3333(7)	9450(5)	2460(4)	36.0(10)

C7	3976(8)	10214(5)	1594(4)	42.4(11)
C8	6010(8)	10695(5)	1414(4)	38.9(11)
C9	7508(7)	10437(4)	2094(4)	35.1(10)
C9A	6931(7)	9667(4)	2995(4)	29.3(9)
C10	8049(6)	9255(4)	3878(4)	28.9(9)
C10A	6645(6)	8557(4)	4534(4)	26.9(8)
O11	8574(4)	8112(3)	6088(3)	33.1(7)
C12	1767(6)	6385(4)	6124(4)	27.8(8)
S13	2047.9(14)	4957.1(9)	6649.7(8)	24.2(3)
O14	217(4)	4378(3)	7165(3)	32.4(7)
O15	2727(5)	4246(3)	5760(3)	32.4(7)
C16	4039(6)	5431(4)	7717(3)	25.7(8)
S17	6331.7(14)	5084.7(10)	7518.6(8)	27.7(3)
C18	7247(6)	5944(4)	8811(3)	24.6(8)
C19	9173(6)	6190(4)	9360(4)	30.9(9)
C20	9519(6)	6899(5)	10388(4)	33.8(10)
C21	8001(7)	7360(4)	10876(4)	35.3(10)
C22	6095(6)	7118(4)	10334(4)	30.0(9)
C23	5715(6)	6405(4)	9284(3)	25.3(8)
N24	3893(5)	6104(3)	8635(3)	26.1(7)

Table S13. Table 3 Anisotropic Displacement Parameters (Å^2×10^3) for **18**. The Anisotropic displacement factor exponent takes the form: $-2\pi^2[h^2a^{*2}U_{11}+2hka^*b^*U_{12}+\ldots]$.

**Atom**	***U*11**	***U*22**	***U*33**	***U*12**	***U*13**	***U*23**
C1	24(2)	21.0(18)	38(2)	6.8(15)	1.6(16)	-0.4(16)
O2	17.6(13)	31.6(15)	34.2(15)	4.4(11)	0.2(11)	2.7(12)
C3	22.3(19)	30(2)	31(2)	5.9(16)	-0.7(16)	-2.2(17)
C4	23(2)	35(2)	35(2)	8.8(17)	1.8(16)	1.3(17)
N5	23.5(17)	31.3(18)	30.0(18)	8.7(14)	1.5(13)	-1.1(14)
C5A	27(2)	32(2)	29(2)	12.4(17)	1.8(16)	-3.2(16)
C6	28(2)	46(3)	35(2)	13.2(19)	-2.3(18)	0.7(19)
C7	52(3)	49(3)	31(2)	24(2)	-3(2)	1(2)
C8	51(3)	38(2)	29(2)	15(2)	3(2)	1.4(18)
C9	38(2)	30(2)	37(2)	8.8(18)	4.7(19)	0.4(18)
C9A	31(2)	26(2)	33(2)	11.3(16)	4.5(17)	-2.4(16)
C10	26(2)	25.3(19)	35(2)	9.0(16)	1.3(16)	-0.9(16)
C10A	23.6(19)	24.5(19)	34(2)	9.4(15)	0.3(16)	-2.7(16)
O11	21.0(14)	35.8(16)	42.3(17)	7.4(12)	-2.7(12)	4.9(13)
C12	18.1(18)	30(2)	37(2)	9.5(15)	0.7(16)	2.2(17)
S13	17.9(5)	26.0(5)	28.9(5)	7.1(3)	0.5(3)	-1.3(4)
O14	18.9(14)	36.4(16)	40.1(17)	4.1(12)	1.3(12)	4.7(13)
O15	28.9(15)	35.5(16)	33.8(16)	13.5(12)	-0.9(12)	-7.9(13)
C16	23.7(19)	27.3(19)	28.0(19)	9.0(15)	6.1(15)	4.5(15)
S17	18.7(5)	38.3(6)	28.7(5)	13.3(4)	2.0(3)	-2.6(4)
C18	23.4(19)	27.6(19)	24.5(18)	9.2(15)	3.5(15)	2.7(15)
C19	20.0(19)	41(2)	34(2)	12.9(17)	2.1(16)	3.2(18)
C20	21(2)	45(3)	36(2)	10.5(18)	-4.2(17)	1.2(19)
C21	36(2)	38(2)	32(2)	13.2(19)	-2.6(18)	-4.0(18)
C22	26(2)	36(2)	31(2)	15.5(17)	3.4(16)	0.5(17)
C23	22.4(19)	26.4(19)	29(2)	8.5(15)	2.2(15)	6.2(15)
N24	23.1(16)	27.1(17)	29.4(17)	9.3(13)	1.9(13)	1.0(13)

Table S14. Bond Lengths for **18**

**Atom**	**Atom**	**Length/Å**	**Atom**	**Atom**	**Length/Å**
C1	O2	1.360(5)	C10	C10A	1.372(6)

C1	C10A	1.464(6)	C12	S13	1.783(4)
C1	O11	1.204(5)	S13	O14	1.435(3)
O2	C3	1.462(5)	S13	O15	1.435(3)
C3	C4	1.513(6)	S13	C16	1.762(4)
C3	C12	1.511(6)	C16	S17	1.735(4)
C4	N5	1.445(5)	C16	N24	1.295(5)
N5	C5A	1.376(6)	S17	C18	1.739(4)
N5	C10A	1.383(5)	C18	C19	1.396(6)
C5A	C6	1.402(6)	C18	C23	1.403(5)
C5A	C9A	1.411(6)	C19	C20	1.375(6)
C6	C7	1.382(7)	C20	C21	1.403(6)
C7	C8	1.389(8)	C21	C22	1.380(6)
C8	C9	1.373(7)	C22	C23	1.399(6)
C9	C9A	1.411(6)	C23	N24	1.385(5)
C9A	C10	1.431(6)			

Table S15. Bond Angles for **18**

**Atom**	**Atom**	**Atom**	**Angle/°**	**Atom**	**Atom**	**Atom**	**Angle/°**
C10A	C1	O2	116.2(3)	C10	C10A	N5	110.0(4)
O11	C1	O2	119.4(4)	S13	C12	C3	111.6(3)
O11	C1	C10A	124.4(4)	O14	S13	C12	107.72(19)
C3	O2	C1	118.0(3)	O15	S13	C12	108.8(2)
C4	C3	O2	111.6(3)	O15	S13	O14	118.62(19)
C12	C3	O2	104.7(3)	C16	S13	C12	105.02(19)
C12	C3	C4	112.2(3)	C16	S13	O14	108.89(19)
N5	C4	C3	106.9(3)	C16	S13	O15	106.95(18)
C5A	N5	C4	128.3(4)	S17	C16	S13	120.2(2)
C10A	N5	C4	122.6(4)	N24	C16	S13	121.7(3)
C10A	N5	C5A	108.7(3)	N24	C16	S17	117.9(3)
C6	C5A	N5	130.1(4)	C18	S17	C16	87.90(19)
C9A	C5A	N5	107.6(4)	C19	C18	S17	128.8(3)
C9A	C5A	C6	122.3(4)	C23	C18	S17	109.7(3)
C7	C6	C5A	116.2(4)	C23	C18	C19	121.5(4)
C8	C7	C6	122.5(5)	C20	C19	C18	117.6(4)
C9	C8	C7	121.6(5)	C21	C20	C19	121.6(4)
C9A	C9	C8	118.2(4)	C22	C21	C20	120.9(4)
C9	C9A	C5A	119.2(4)	C23	C22	C21	118.4(4)
C10	C9A	C5A	107.5(4)	C22	C23	C18	120.0(4)
C10	C9A	C9	133.3(4)	N24	C23	C18	115.2(4)
C10A	C10	C9A	106.4(4)	N24	C23	C22	124.9(4)
N5	C10A	C1	120.4(4)	C23	N24	C16	109.4(3)
C10	C10A	C1	129.5(4)				

Table S16. Hydrogen Atom Coordinates (Å×10^4) and Isotropic Displacement Parameters (Å^2×10^3) for **18**

**Atom**	***x***	***y***	***z***	***U*(iso)**
H3	3490(70)	6170(40)	4700(40)	23(11)
H4a	1754(6)	7606(4)	4127(4)	36.8(11)
H4b	2599(6)	8590(4)	5217(4)	36.8(11)
H6	1938(7)	9118(5)	2572(4)	43.2(12)
H7	2990(8)	10417(5)	1103(4)	50.9(14)
H8	6374(8)	11216(5)	806(4)	46.7(13)
H9	8895(7)	10769(4)	1961(4)	42.2(12)
H10	9480(6)	9430(4)	3988(4)	34.7(10)

H12a	1839(6)	7015(4)	6773(4)	33.4(10)
H12b	420(6)	6225(4)	5714(4)	33.4(10)
H19	10207(6)	5879(4)	9035(4)	37.1(11)
H20	10819(6)	7082(5)	10778(4)	40.5(11)
H21	8287(7)	7844(4)	11589(4)	42.3(12)
H22	5068(6)	7429(4)	10667(4)	36.0(11)

References:

1. O.V. Dolomanov, L.J. Bourhis, R.J. Gildea, J.A.K. Howard, and H. Puschmann, *J. Appl. Cryst.*, 2009, **42**, 339-341.
2. M.C. Burla, R. Caliandro, M. Camalli, B. Carrozzini, G.L. Cascarano, L. De Caro, C. Giacovazzo, G. Polidori, D. Siliqi, and R. Spagna, *J. Appl. Cryst*., 2007, **40**, 609-613.
3. L.J. Bourhis, O.V. Dolomanov, R.J. Gildea, J.A.K. Howard and H. Puschmann, *Acta Cryst*., 2015, **A71**, 59-75.
